# Mechanochemical enzymatic resolution of *N*-benzylated-β^3^-amino esters

**DOI:** 10.3762/bjoc.13.167

**Published:** 2017-08-18

**Authors:** Mario Pérez-Venegas, Gloria Reyes-Rangel, Adrián Neri, Jaime Escalante, Eusebio Juaristi

**Affiliations:** 1 Departamento de Química, Centro de Investigación y de Estudios Avanzados, Avenida I.P.N. 2508, Ciudad de México, 07360, Mexico; 2 Centro de Investigaciones Químicas, Universidad Autónoma del Estado de Morelos, Av. Universidad 1001, Cuernavaca, Morelos, 62210, Mexico; 3 El Colegio Nacional, Luis Gonzáles Obregón 23, Centro Histórico, Ciudad de México, 06020, Mexico

**Keywords:** ball-milling, β^3^-amino acid, *Candida antarctica* lipase B, enzymatic resolution, mechanochemistry

## Abstract

The use of mechanochemistry to carry out enantioselective reactions has been explored in the last ten years with excellent results. Several chiral organocatalysts and even enzymes have proved to be resistant to milling conditions, which allows for rather efficient enantioselective transformations under ball-milling conditions. The present article reports the first example of a liquid-assisted grinding (LAG) mechanochemical enzymatic resolution of racemic β^3^-amino esters employing *Candida antarctica* lipase B (CALB) to afford highly valuable enantioenriched *N*-benzylated-β^3^-amino acids in good yields. Furthermore the present protocol is readily scalable.

## Introduction

β-Amino acids are rather interesting molecules that frequently exhibit exceptional biological properties [1–3]; for instance, some of them are efficient inhibitors of several enzymes [4–5]. Furthermore, β-amino acid residues can be used to protect peptides and proteins against the activity of proteolytic enzymes [6–7], or are precursors of numerous active compounds such as β-lactams [8–9]. Finally, β-amino acids are present in numerous natural products [10]. These properties have generated great interest in the development of synthetic methods for the preparation of β-amino acids, especially protocols leading to products with high enantiomeric excess (ee), which are required to test the pharmacological activity of each enantiomer [11–13]. In this regard, several methods for the asymmetric synthesis of β-amino acids have been documented [14–22] including strategies based on organocatalysis [23–26] and kinetic resolution using enzymes such as *Candida antarctica* lipase B, which was shown to be efficient in the resolution of racemic β-amino acids under various conditions [27–30].

Among recent developments in instrumentation for synthetic chemistry, mechanochemistry has proved a rather attractive and useful technique [31–37]. In particular, it has been demonstrated that mechanochemistry allows for the generation of products through catalysts that can be recovered and reused [38–44], so this converts mechanochemistry into a green technique, whose field of application is still very wide.

In this context, the use of a minimal amount of solvent (LAG) enable the development of convenient ball-milling protocols. In particular, LAG facilitates mechanochemical applications on a large scale [45–46].

**Scheme 1:**
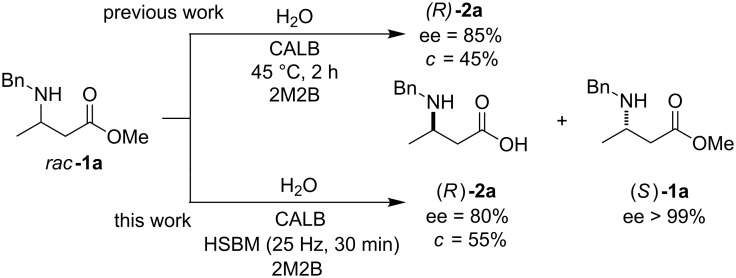
Enantioselective enzymatic hydrolysis of racemic β^3^-amino ester *rac***-1a** using CALB in solution [52] (top) and under HSBM conditions (button). 2M2B: 2-methyl-2-butanol.

Very recently, Hernández, Frings, and Bolm developed a method to carry out the kinetic resolution of secondary alcohols through selective acylation using *Candida antarctica* lipase B, under solvent-free ball-milling conditions [47–48]. Inspired by this ground-breaking report, which is in line with our continuous interest in developing new sustainable organocatalytic protocols [39,49–51], and taking advantage of previous experience with the enzymatic hydrolysis of a racemic mixture of *N*-protected-β^3^-amino acid methyl esters [52], we decided to examine the use of CALB enzyme under high-speed ball-milling (HSBM) conditions as a method to obtain enantiopure *N*-benzylated-β^3^-amino acids (Scheme 1).

## Results and Discussion

A racemic mixture of substrate *rac*-**1a** (82 mg, 1 equiv) was milled in an Agate jar (12 mm of diameter, 4.6 mL) with an Agate ball (6 mm of diameter, 480 mg weight) using water (3.6 μL, 0.5 equiv), 0.2 mL of 2-methyl-2-butanol (2M2B) as a LAG additive (η = 1.63) and 40 mg of CALB (Novozym 435, Novozymes, recombinant, expressed in Aspergillus niger, immobilized in acrylic resin, >10000 U/g) at 25 Hz during 30 min. Gratifyingly, 55% conversion to the enantioenriched (*R*)-*N*-benzylated-β^3^-amino acid (*R*)-**2a** was observed, recovering 51% of enantioenriched starting material. It could be established by chiral HPLC that the ee of the product amounted 80% (Table 1, entry 1). This assay demonstrated that enzymatic hydrolysis can indeed be carried out under HSBM conditions. A second reaction was carried out under the same conditions but in the absence of the enzyme, which did not proceed and the starting material was recovered in its totality. This result shows that the observed hydrolysis is induced by CALB and not by the milling process per se. Furthermore, it could be established that the CALB enzyme and *N*-benzylated-β^3^-amino esters are stable to the mechanical force caused by HSBM. We then focused our attention on the search of the best conditions for this enzymatic mechanochemical resolution.

**Table 1:** Search of the best parameters in the enzymatic enantioselective hydrolysis of *rac*-**1a** under ball milling.

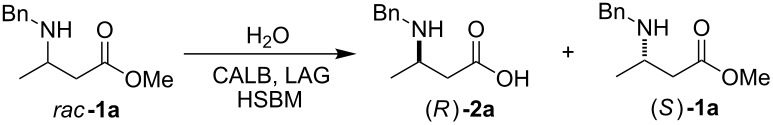							

entry^a^	LAG additive^b^	yield (%)^c^ (*S*)-**1a**/(*R*)-**2a**	time (h)	ee (*S*)-**1a** (%)^d^	ee (*R*)-**2a** (%)^d^	*c^e^* (%)	*E*^f^

1^g^	2M2B	51/49	0.5	99	80	55	46
2	2M2B	70/30	0.5	89	77	54	23
**3**	**2M2B**	**51/49**	**1**	**99**	**95**	**51**	**>200**
4	AcOEt	86/13	1	69	95	42	81
5	IPA	82/21	1	48	95	34	63
6	CH_3_CN	65/29	1	65	95	41	77
7	hexane	40/60	1	97	86	53	55
8	–	58/41	1	95	92	51	89
9^g^	–	58/42	1	93	86	52	45
10^h^	–	68/31	1	74	80	48	20

^a^Reactions were carried out with 0.5 equivalents of water and 15 Hz of frequency. ^b^0.2 mL of LAG additive was used. ^c^Determined after purification by flash chromatography. ^d^Determined by HPLC with chiral stationary phase. ^e^Calculated from *c* = ee_s_/(ee_s_ + ee_p_). ^f^*E* = ln[1 − *c*(1 + ee_p_)]/ln[1 − *c*(1 − ee_p_)]. ^g^25 Hz of frequency was used. ^h^0.25 equivalents of water were used.

First of all, we examined the effect of the milling frequency, 15 Hz (Table 1, entry 2). Both yield and ee decreased substantially in comparison with the initial approach carried out at 25 Hz (Table 1, entry 1). Nevertheless, when the reaction time was increased from 30 min to 1 h at 15 Hz (Table 1, entry 3) the yield of the *N*-benzylated-β^3^-amino acid reached 49%, and presented high ee (95%, *E* > 200). These data represent an improvement both in ee and yield compared with the data recorded in solution [52]. Motivated by this result, we investigated the effect of other LAG additives in the reaction (see Supporting Information File 1, Table S1, entries 4–10). When 2M2B was replaced with other LAG additives a lower yield was observed (Table 1, entries 4–6). Nevertheless, the enantioselectivity of the process is maintained (95% ee), except when hexane was used (Table 1, entry 7), where a higher yield was observed (60%) although with a lower enantiomeric excess (86% ee). In the absence of a LAG additive and using 0.25 equivalents of water (Table 1, entries 8–10) both yield and ee were lower.

Water plays an important role in the reaction controlling the activity of the enzyme; for example, the use of 0.5 equivalents of water yielded 49% of product **2a** (Table 1, entry 3). However, when 1 equivalent of water was employed the yield of the product increased to 92%. By contrast, when the reaction was carried out in the absence of water only traces of product were detected (see Supporting Information File 1 Table S1).

To determine the substrate scope, the conditions that led to the best results in the enzymatic resolution of substrate *rac*-**1a** (Table 1, entry 3) were employed with other racemic *N*-benzylated-β^3^-amino esters as substrates (Table 2). It can be appreciated that reaction yields decrease when longer aliphatic chains are present in the substrate (Table 2, entries 1–5), although the ee in products **2b–f** remained rather high (>90%). Notably, this aliphatic chain-length effect has been studied in other systems with similar results [53].

**Table 2:** Substrate scope for the enzymatic resolution of *N*-benzylated-β^3^-amino esters.

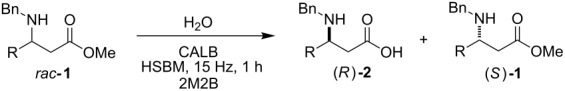										

entry^a^	*rac*	R	yield (%)^b^ (*S*)-**1**/(*R*)-**2**	ee^c^ (*S*)-**1** (%)	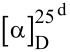	ee^c^ (*R*)-**2** (%)	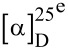	*c^f^* (%)	*E*^g^	absolute configuration^h^

1	**1b**	CH_3_-(CH_2_)-	51/49	91	4.5	97	−36.5	48	>200	*R*
2	**1c**	CH_3_-(CH_2_)_2_-	53/43	84	2.1	98	−45.2	46	>200	*R*
3	**1d**	CH_3_-(CH_2_)_3_-	68/29	23	2.0	94	−35.3	20	40	*R*
4	**1e**	CH_3_-(CH_2_)_4_-	74/24	57	0.2	94	−40.0	15	38	*R*
5	**1f**	CH_3_-(CH_2_)_5_-	79/18	13	0.8	91	−39.7	13	24	*R*
6^i^	**1g**	Ph	92/10	18	3.4	83	−35.0	18	13	*S*
7^i^	**1h**	4-MeO-Ph	89/10	1	−0.5	80	−31.7	1	9	*S*
8	**1i**	*t*-Bu	89/4	4	−0.6	94	12.8	4	34	*S*

^a^Reactions were carried out with 0.5 equivalents of water and 0.2 mL of 2M2B at 15 Hz during 1 h. ^b^Determined after purification by flash chromatography. ^c^Determined by HPLC with chiral stationary phase. ^d^*c* = 0.33 in CH_3_Cl. ^e^*c* = 0.33 in MeOH. ^f^Calculated from *c* = ee_s_/(ee_s_ + ee_p_). ^g^*E* = ln[1 − *c*(1 + *ee*_p_)]/ln[1 − *c*(1 − *ee*_p_)]. ^h^Assigned by chemical correlation and by HPLC with chiral stationary phase. ^i^0.75 equivalents of water were used.

The introduction of an aromatic ring (either unsubstituted or *para*-substituted) in the substrate resulted in diminished yields (Table 2, entries 6 and 7) but good ee (≥80%). With bulky groups, such as *tert*-butyl, the experimentally observed low yield was accompanied nevertheless by high ee (Table 2, entry 8). Other reaction conditions were tested aiming of increasing both yield and ee (see Supporting Information File 1); however, the best results continued to be obtained by using the conditions indicated in Table 1, entry 3.

**Figure 1:**
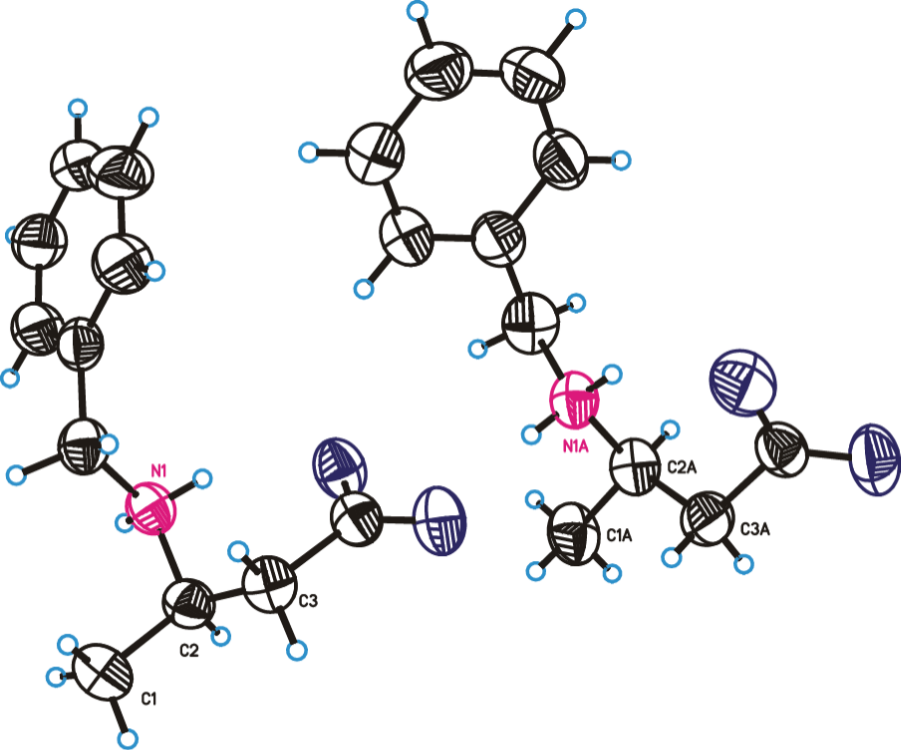
X-ray crystallographic structure of product (*R*)-**2a** (50% of probability ellipsoids). CCDC registry number 1552645.

To establish the absolute configuration of product **2a**, a sample was crystallized to give a suitable single-crystal for X-ray diffraction analysis. The resulting structure showed the *R* configuration (Flack parameter = 0.154) in the stereocenter delimited by the atoms marked as C1, N1 and C3 (Figure 1). The *R* configuration in hydrolyzed product **2a** was confirmed by comparison with literature data [52]. The configuration of products **2b**, **2g** to **2i** was also assigned by comparison with literature data [52,54–55]. Finally, in the case of products **2c**–**f**, comparison of the elution order for both enantiomers with the tendency found in **2a** and **2b** suggested that the configuration is the same in all of them (see Supporting Information File 1) [56–57].

The enzyme employed in these experiments was recovered by centrifugation of the reaction crude followed by drying under vacuum (90% of recovered enzyme; we will call it rCALB). This recovered material was reused to evaluate the enzyme recyclability after the mechanochemical protocol. When the reaction was carried out using the recovered enzyme the yield was not as good as the obtained with fresh catalyst (compare entries 1 and 2 in Table 3). This might suggest that the enzyme undergoes partial denaturation and/or partial destruction of the support, within each cycle (Table 3, entry 3). Interestingly, however, ee values of the isolated β-amino acid still resulted quite acceptable. On the other hand, no product was detected after the third cycle. To evaluate the denaturalization of the enzyme provoked by the milling process, a sample of fresh catalyst was milled for 1 h at 15 Hz under solvent-free conditions and in the presence of a LAG additive, finding that both reaction yield (38%) and ee (>90%) are higher (see Supporting Information File 1, Table S3, entries 5 and 6), compared with results from the hydrolysis using the catalyst recovered after the first cycle (Table 4, entry 2). The milling process carried out using the catalyst milled with 2M2B presents a slight decrease in ee compared with the resolution reaction using the milled enzyme under solvent-free conditions. This observation suggests that the LAG additive increases to some extent the degree of denaturation of the enzyme, reducing the enantiodiscrimination (ee = 91%) although maintaining significant catalytic activity (yield = 38%).

**Table 3:** Recycling capacity of immobilized CALB under HSBM conditions.

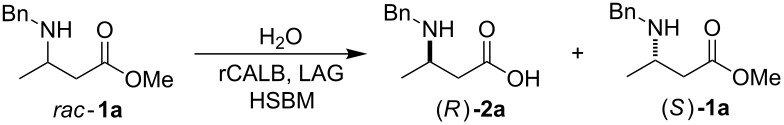						

entry^a^	recycling cycle	yield (%)^b^ (*S*)-**1a**/(*R*)-**2a**	ee^c^ (*S*)-**1a** (%)	ee^c^ (*R*)-**2a** (%)	*c^d^* (%)	*E*^e^

1	–	51/49	49	95	51	>200
2	1	65/37	35	88	59	22
3	2	80/20	6	80	51	10
4	3	100/0	0	–	–	–

^a^Reactions were carried out with 0.5 equivalents of water and 0.2 mL of 2M2B at 15 Hz during 1 h. ^b^Determined after purification by flash chromatography. ^c^Determined by HPLC with chiral stationary phase. ^d^Calculated from *c* = ee_s_/(ee_s_ + ee_p_). ^e^*E* = ln[1 − *c*(1 + ee_p_)]/ln[1 − *c*(1 − ee_p_)].

Finally, to test the scalability of the process, a set of reactions was carried out increasing the amount of substrate *rac*-**1a** under the optimized reaction parameters. (Table 4).

**Table 4:** Scaling-up of the enzymatic hydrolysis reaction under ball-milling using substrate *rac*-**1a**.

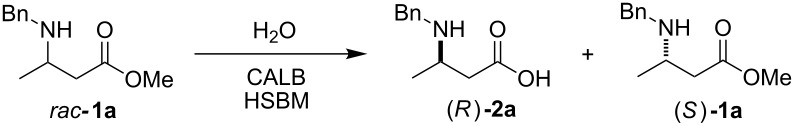						

entry^a^	catalyst/substrate (equiv) ^b^	yield (%)^c^ (*S*)-**1a**/(*R*)-**2a**	ee^d^ (*S*)-**1a** (%)	ee^d^ (*R*)-**2a** (%)	*c^e^* (%)	*E*^f^

1^g^	1/1	51/49	>99	95	51	>200
2	1/3	52/48	62	93	40	52
3	1/6	61/42	53	93	36	47
4	1/9	59/40	49	94	34	53

^a^Reactions were carried out with 0.5 equivalents of water at 15 Hz during 1 h. ^b^1 equivalent of enzyme = 40 mg; 1 equivalent of susbtrate = 82 mg. ^c^Determined after purification by flash chromatography. ^d^Determined by HPLC with chiral stationary phase. ^e^Calculated from *c* = ee_s_/(ee_s_ + ee_p_). ^f^*E* = ln[1 − *c*(1 + ee_p_)]/ln[1 − *c*(1 − ee_p_)]. ^g^0.2 mL of LAG additive were used.

Relative to the results obtained with 1 equivalent of *rac*-**1a** in the presence of LAG additive (Table 4, entry 1) a slight decrease in yield was observed when 3 equivalents of substrate (and no LAG additive) were used to carry out the reaction (Table 4, entry 2). Nevertheless, the hydrolysis still proceeds with excellent ee (93%). This result confirms that under solvent-free conditions a particular amount of enzyme can catalyze a larger amount of substrate, even up to nine equivalents, without loss of enantiodiscrimination (Table 4, entry 4). It appears that this high efficiency is a consequence of the highly-concentrated medium that is generated under solvent-free mechanochemical conditions, an effect that is not possible to reach in solution [52]. This effect also allows for an increase in the amount of product per cycle of the enzymatic reaction.

## Conclusion

The capacity of immobilized CALB to carry out the enzymatic hydrolytic resolution of *N*-benzylated-β^3^-amino esters under mechanochemical conditions was demonstrated. The reaction proceeds with an excellent yield (up to 49% of the theoretical 50% maximum) and high enantioselectivity (up to 98% ee). The method proved to be efficient in the resolution of racemic mixtures of β^3^-amino esters with aliphatic chains of different lengths, affording high ees of the resulting β-amino acids in spite of a decrease in yield in the case of long aliphatic chains. This efficiency of the enzymatic process is also observed in substrates with bulky aromatic groups (ee ≥ 80%). The reaction is best carried out in the presence of the LAG additive 2-methyl-2-butanol when the concentration of the substrate is low. The enzymatic process could be scaled-up to 9-fold affording the hydrolyzed product with high ee (≥93%) and an excellent yield (40% out of a 50% theoretical maximum). Finally, the enzyme catalyst could be recovered and reused several times affording the desired amino acids with good ee although with a decrease in conversion due to a partial denaturation process and partial destruction of the enzyme support.

## Supporting Information

File 1 Experimental section, NMR spectra, chromatograms and X-ray diffraction data.
