# Correction: Mechanochemical enzymatic resolution of *N*-benzylated-β^3^-amino esters

**DOI:** 10.3762/bjoc.13.210

**Published:** 2017-10-12

**Authors:** Mario Pérez-Venegas, Gloria Reyes-Rangel, Adrián Neri, Jaime Escalante, Eusebio Juaristi

**Affiliations:** 1 Departamento de Química, Centro de Investigación y de Estudios Avanzados, Avenida I.P.N. 2508, Ciudad de México, 07360, Mexico; 2 Centro de Investigaciones Químicas, Universidad Autónoma del Estado de Morelos, Av. Universidad 1001, Cuernavaca, Morelos, 62210, Mexico; 3 El Colegio Nacional, Luis Gonzáles Obregón 23, Centro Histórico, Ciudad de México, 06020, Mexico

**Keywords:** ball-milling, β^3^-amino acid, *Candida antarctica* lipase B, enzymatic resolution, mechanochemistry

The original published Tables 1–4 and Supporting Information File 1 contain some incorrect values. In this Correction article the whole corrected Tables are given. The positions of the correted values are described in detail in the paragraphs below.

In Table 1 in entry 5 the yield (column 3) has been corrected.

**Table 1:** Search of the best parameters in the enzymatic enantioselective hydrolysis of *rac*-**1a** under ball milling.

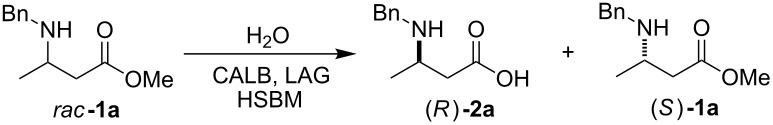							

entry^a^	LAG additive^b^	yield (%)^c^ (*S*)-**1a**/(*R*)-**2a**	time (h)	ee (*S*)-**1a** (%)^d^	ee (*R*)-**2a** (%)^d^	*c^e^* (%)	*E*^f^

1^g^	2M2B	51/49	0.5	99	80	55	46
2	2M2B	70/30	0.5	89	77	54	23
**3**	**2M2B**	**51/49**	**1**	**99**	**95**	**51**	**>200**
4	AcOEt	86/13	1	69	95	42	81
5	IPA	80/20	1	48	95	34	63
6	CH_3_CN	65/29	1	65	95	41	77
7	hexane	40/60	1	97	86	53	55
8	–	58/41	1	95	92	51	89
9^g^	–	58/42	1	93	86	52	45
10^h^	–	68/31	1	74	80	48	20

^a^Reactions were carried out with 0.5 equivalents of water and 15 Hz of frequency. ^b^0.2 mL of LAG additive was used. ^c^Determined after purification by flash chromatography. ^d^Determined by HPLC with chiral stationary phase. ^e^Calculated from *c* = ee_s_/(ee_s_ + ee_p_). ^f^*E* = ln[1 − *c*(1 + ee_p_)]/ln[1 − *c*(1 − ee_p_)]. ^g^25 Hz of frequency was used. ^h^0.25 equivalents of water were used.

In Table 2 in entry 4 the ee value of (*S*)-**1** (column 5), and in entry 6 the yield (column 4) have been corrected.

**Table 2:** Substrate scope for the enzymatic resolution of *N*-benzylated-β^3^-amino esters.

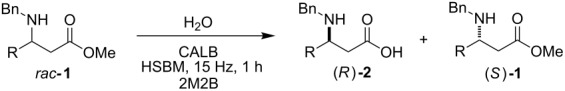										

entry^a^	*rac*	R	yield (%)^b^ (*S*)-**1**/(*R*)-**2**	ee^c^ (*S*)-**1**(%)	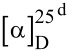	ee^c^ (*R*)-**2**(%)	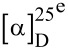	*c^f (^*%)	*E*^g^	absolute configuration^h^

1	**1b**	CH_3_-(CH_2_)-	51/49	91	4.5	97	−36.5	48	>200	*R*
2	**1c**	CH_3_-(CH_2_)_2_-	53/43	84	2.1	98	−45.2	46	>200	*R*
3	**1d**	CH_3_-(CH_2_)_3_-	68/29	23	2.0	94	−35.3	20	40	*R*
4	**1e**	CH_3_-(CH_2_)_4_-	74/24	16	0.2	94	−40.0	15	38	*R*
5	**1f**	CH_3_-(CH_2_)_5_-	79/18	13	0.8	91	−39.7	13	24	*R*
6^i^	**1g**	Ph	90/10	18	3.4	83	−35.0	18	13	*S*
7^i^	**1h**	4-MeO-Ph	89/10	1	−0.5	80	−31.7	1	9	*S*
8	**1i**	*t*-Bu	89/4	4	−0.6	94	12.8	4	34	*S*

^a^Reactions were carried out with 0.5 equivalents of water and 0.2 mL of 2M2B at 15 Hz during 1 h. ^b^Determined after purification by flash chromatography. ^c^Determined by HPLC with chiral stationary phase. ^d ^*c* = 0.33 in CH_3_Cl. ^e^*c* = 0.33 in MeOH. ^f^Calculated from *c* = ee_s_/(ee_s_ + ee_p_). ^g^*E* = ln[1 − *c*(1 + ee_p_)]/ln[1 − *c*(1 − ee_p_)]. ^h^Assigned by chemical correlation and by HPLC with chiral stationary phase. ^i^0.75 equivalents of water were used.

In Table 3 entry 1 the ee of (*S*)-**1a** (column 4), in entry 2 the yield (column 3) and the *c* value (column 6), and in entry 3 the *c* value (column 6) have been corrected.

**Table 3:** Recycling capacity of immobilized CALB under HSBM conditions.

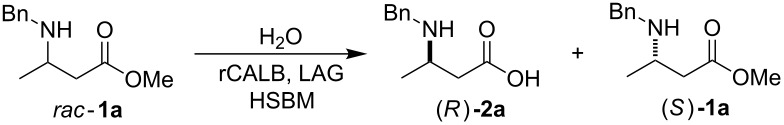						

entry^a^	Recycling cycle	yield (%)^b^ (*S*)-**1a**/(*R*)-**2a**	ee^c^ (*S*)-**1a** (%)	ee^c^ (*R*)-**2a** (%)	*c^d^* (%)	*E*^e^

1	–	51/49	99	95	51	>200
2	1	65/35	35	88	28	22
3	2	80/20	6	80	7	10
4	3	100/0	0	–	–	–

^a^Reactions were carried out with 0.5 equivalents of water and 0.2 mL of 2M2B at 15 Hz during 1 h. ^b^Determined after purification by flash chromatography. ^c^Determined by HPLC with chiral stationary phase. ^d^Calculated from *c* = ee_s_/(ee_s_ + ee_p_). ^e^*E* = ln[1 − *c*(1 + *ee*_p_)]/ln[1 − *c*(1 – *ee*_p_)].

In Table 4 in entry 3 the yield (column 3) has been corrected.

**Table 4:** Scaling-up of the enzymatic hydrolysis reaction under ball-milling using substrate *rac*-**1a**.

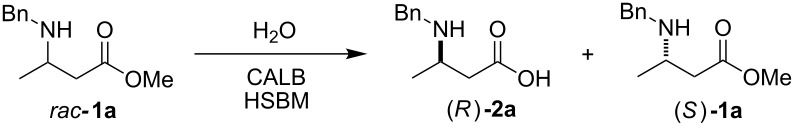						

entry^a^	catalyst/substrate (equiv) ^b^	yield (%)^c^ (*S*)-**1a**/(*R*)-**2a**	ee^d^ (*S*)-**1a** (%)	ee^d^ (*R*)-**2a** (%)	*c^e^* (%)	*E*^f^

1^g^	1/1	51/49	>99	95	51	>200
2	1/3	52/48	62	93	40	52
3	1/6	61/38	53	93	36	47
4	1/9	59/40	49	94	34	53

^a^Reactions were carried out with 0.5 equivalents of water at 15 Hz during 1 h. ^b^1 equivalent of enzyme = 40 mg; 1 equivalent of susbtrate = 82 mg. ^c^Determined after purification by flash chromatography. ^d^Determined by HPLC with chiral stationary phase. ^e^Calculated from *c* = ee_s_/(ee_s_ + ee_p_). ^f^*E* = ln[1 − *c*(1 + *ee*_p_)]/ln[1 − *c*(1 − *ee*_p_)]. ^g^0.2 mL of LAG were used.

A corrected version of Supporting Information File 1 is also part of this Correction. The new Supporting Information File 1 is the complete file with the corrections marked in yellow color.

## Supporting Information

File 1 Experimental section, NMR spectra, chromatograms and X-ray diffraction data.

