# Supplementary material for: Mechanochemical enzymatic resolution of N-benzylated-β3-amino esters
Source: Beilstein J Org Chem. 2017 Aug 18;13:1728–34. doi: 10.3762/bjoc.13.167 (PMC5564257; doi:10.3762/bjoc.13.167)
Supplement: File 1 — Experimental section, NMR spectra, chromatograms and X-ray diffraction data. [file Beilstein_J_Org_Chem-13-1728-s001.pdf]

# Supporting Information

**for**

# Mechanochemical enzymatic resolution of *N*-benzylated-β$^3$-amino esters

Mario Pérez-Venegas[1], Gloria Reyes-Rangel[1], Adrián Neri[2], Jaime Escalante*[,2] and Eusebio Juaristi.*[,1,3]

Address: [1]Departamento de Química, Centro de Investigación y de Estudios Avanzados, Avenida I.P.N. 2508, Ciudad de México, 07360, Mexico, [2]Centro de Investigaciones Químicas, Universidad Autónoma del Estado de Morelos, Av. Universidad 1001, Cuernavaca, Morelos, 62210, Mexico and [3]El Colegio Nacional, Luis Gonzáles Obregón 23, Centro Histórico, Ciudad de México, 06020, Mexico

Email: Jaime Escalante* - jaime@uaem.mx; Eusebio Juaristi* - juaristi@relaq.mx
*Corresponding author

**Experimental section, NMR spectra, chromatograms and X-ray diffraction data**

**Table of Contents**


1) **General methods** **S2**
2) **General method for the synthesis of *N*-benzylated-β$^3$-amino esters** **S2**
3) **General method for the synthesis of *N*-benzylated-β$^3$-amino acid** **S9**
4) **General method for the enzymatic resolution using CALB** **S13**
5) **$^1$H and $^{13}$C spectra for M*t*E-b to M*t*E-d, *rac*-1a to *rac*-1j and products 2a to 2j** **S18**
6) **Chromatograms for the determination of enantiomeric excess of raw material and hydrolysis products** **S43**
7) **HPLC conditions and retention times for the substrates *rac*-1a to *rac*-1j and hydrolysis products *rac*-2a to *rac*-2j** **S94**
8) **Determinations of the absolute configuration for the products 2a to 2j.** **S95**
9) **References** **S98**

## 1) General methods

NMR spectra were recorded on a BRUKER DP300 (300 MHz) and a Jeol Eclipse (400 MHz). High resolution mass spectra were recorded on a HPLC 1100 coupled to a MSD-TOF Agilent series HR-MSTOF model 1969 A. Chromatograms were acquired in a Dionex HPLC Ultimate 3000 with a UV/Visible detector, with diode array, at 210 and 254 nm. HSBM reactions were carry out in a Retsch, Mixer Mill (MM200). *N*-Benzylated-β$^3$-amino esters were synthetized according to the literature. All reagents for the synthesis were purchased from Sigma-Aldrich. Immobilized CALB was purchased from Novozymes, Novozym 453 (Immobilized on acrylic resine, U/g >100000)

## 2) General method for the synthesis of *N*-benzylated-β$^3$-amino esters.

The substrates *rac*-**1a** to *rac*-**1f** (Table 2 in article) for the enzymatic resolution were synthetized according to previously reported methodologies [1-3], the synthesis for *rac*-**1a** starts from methyl crotonate. For substrates *rac*-**1g** to *rac*-**1i** we used the methodology described by Escalante [4].

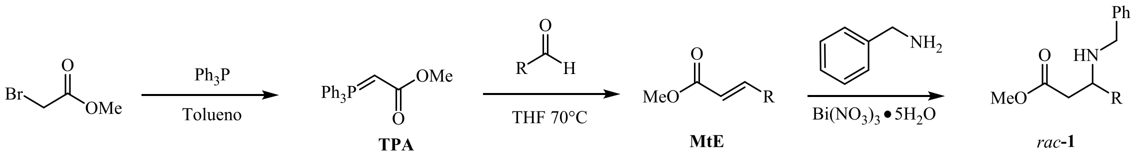


**Scheme S1:** Route for the synthesis of substrates rac-**1**.

Synthesis of methyl (triphenylphosphoranyldiene)acetate (**TPA**): Triphenylphosphine (7.86 g, 0.03 mmol) was placed in a round flask equipped with a magnetic bar and was dissolved in toluene (100 mL), methyl bromoacetate (5.01 g, 0.03 mmol) was added and the reaction was stirred overnight. The reaction product was filtered and the resulting solid was dissolved in water (200 mL), basified till pH 9 using KOH (2 M) and phenolphthalein as indicator and was extracted with $CH_2Cl_2$ (150 mL × 2), the organic fractions were collected, dried with anhydride

sodium sulfate and concentrated to obtain 9.3 g (93% yield) of a white solid (**TPA**). MS-TOF: calculated for $C_{21}H_{20}0_2P$ 335.1156, found 335.1197. $^1$H NMR ($CDCl_3$, 300MHz): δ= 7.53 (m, 15H), 3.51 (s, 3H). $^{13}$C{$^1$H} NMR ($CDCl_3$, 300MHz): δ 28.7, 30.5, 50.0, 128.8, 128.9, 132.1, 133.0, 133.1. $^{31}$P{$^1$H} NMR ($CDCl_3$, 300MHz): δ 18.9, 23.1, 30.3.

Representative example for the synthesis of methyl *trans*-2 pentenoate (**M*t*E-b**): In a round flask equipped with a magnetic bar **TPA** (10.02 g 0.03 mmol) was dissolved in anhydrous THF (150 mL), propionaldehyde (1.32 g, 0.03 mmol) was added to the solution and the reaction mixture was refluxed for 4 h. The reaction was filtered and evaporated for his purification. The product was purified by flash chromatography on silica gel (hexanes/ethyl acetate) to give 5.2 g of a colorless liquid (**M(*E*)E-b**) (86% yield).

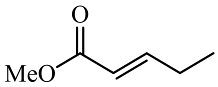


- Methyl *trans*-2 pentenoate (**M*t*E-b**) [5], from propionaldehyde, colorless liquid, 86% yield. $^1$H NMR ($CDCl_3$, 300MHz): δ 7.01 (dt, *J*=6.3 Hz, $J_{trans}$=15.6 Hz, 1H), 5.80 (d, *J*=15.6 Hz, 1H), 3.71 (s, 3H), 2.21 (quint, *J*=6.7 Hz, 2H), 1.05 (t, *J*=7.2 Hz, 3H), 5.28 ($CH_2Cl_2$). $^{13}$C{$^1$H} NMR ($CDCl_3$, 300MHz): δ 12.3, 25.5, 51.5, 120.1, 151.2, 167.4.

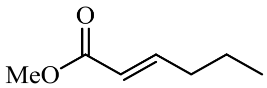


- Methyl *trans*-2 hexenoate (**M*t*E-c**) [6], from butyraldehyde, colorless liquid, 70% yield. $^1$H NMR ($CDCl_3$, 300MHz): δ 6.96 (dt, *J*=6.9 Hz, $J_{trans}$=15.6 Hz, 1H), 5.81 (d, *J*=15.6 Hz, 1H), 3.71 (s, 3H), 2.21 (q, *J*=6.8 Hz, 2H), 1.47 (m, *J*=7.5 Hz, 2H), 0.92 (t, *J*=7.3 Hz, 3H), 5.29 ($CH_2Cl_2$). $^{13}$C{$^1$H} NMR ($CDCl_3$, 300MHz): δ 13.7, 21.4, 34.6, 51.4, 121.1, 149.8, 167.6.

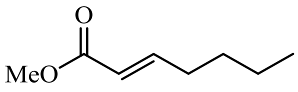

- Methyl *trans*-2 heptenoate (**M*t*E-d**) [7], from valeraldehyde, colorless liquid, 83% yield. $^{1}$H NMR ($CDCl_3$, 300MHz): δ 6.95 (dt, *J*=6.9 Hz, $J_{trans}$=15.6 Hz, 1H), 5.79 (d, *J*=15.6 Hz, 1H), 3.69 (s, 3H), 2.17 (q, *J*=6.9 Hz, 2H), 1.35 (m, 2H), 0.88 (t, *J*=7.2 Hz, 3H). $^{13}$C{$^{1}$H} NMR ($CDCl_3$, 300MHz): δ 13.7, 22.4, 30.2, 32.3, 51.6, 120.9, 149.7, 167.2.

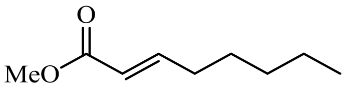

- Methyl *trans*-2 octenoate (**M*t*E-e**) [8], from hexanal, colorless liquid, 80% yield. $^{1}$H NMR ($CDCl_3$, 300MHz): δ 6.82 (dt, *J*=7.2 Hz, $J_{trans}$=15.6 Hz, 1H), 5.80 (d, *J*=15.6 Hz, 1H), 3.71 (s, 3H), 2.18 (q, *J*=7.2 Hz, 2H), 1.44 (quint, *J*=16.5 Hz, 2H), 1.29 (m, 4H), 0.87 (t, *J*=6.3 Hz, 3H). $^{13}$C{$^{1}$H} NMR ($CDCl_3$, 300MHz): δ 41.1, 22.6, 27.8, 31.4, 32.2, 120.8, 150.0, 167.4.

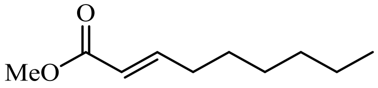

- Methyl *trans*-2 nonenoate (**M*t*E-f**) [9], from heptanal, colorless liquid, 75% yield. $^{1}$H NMR ($CDCl_3$, 300MHz): δ 6.96 (dt, *J*=6.9 Hz, $J_{trans}$=15.6 Hz, 1H), 5.85 (d, *J*=15.9 Hz, 1H), 3.71 (s, 3H), 2.18 (q, *J*=7.2 Hz, 2H), 1.43 (quint, *J*=5.1 Hz, 2H), 1.27 (m, 4H), 0.87 (t, *J*=6.6 Hz, 3H). $^{13}$C{$^{1}$H} NMR ($CDCl_3$, 300MHz): δ 14.2, 22.7, 28.1, 29.0, 31.7, 32.4, 51.6, 120.9, 150.1, 167.5.

Representative example for the synthesis of methyl 3-(benzylamino)butanoate (*rac*-**1a**): $Bi(NO_3)_3 \cdot 5H_2O$ (0.8 g) was added to a round flask containing methyl crotonate (3.18 mL, 0.03 mmol) equipped with a stir bar, the reaction was stirred by 5 min at 0 °C, after that benzylamine (3.27 mL, 0.03 mmol) was added. The reaction was stirred overnight, filtered, extracted with $CH_2Cl_2$, dried with anhydrous sodium sulfate and concentrated for his purification, obtaining 4 g (63% yield) of an amber liquid, *rac*-**1a**. Compounds *rac*-**1g** to *rac*-**1i** were synthetized as describe in literature [4].

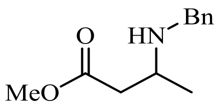


- *rac*-Methyl 3-(benzylamino)butanoate (*rac*-**1a**) [10], from methyl crotonate, amber liquid, 63% yield. MS-TOF: calculated for $C_{12}H_{18}N0_2$ 208.1293, found 208.1331. $^1H$ NMR ($CDCl_3$, 300MHz): δ 7.32 (d, *J*=4.2 Hz, 4H), 7.26 (m, 1H), 3.79 (q, *J*=12.3 Hz, 2H), 3.67 (s, 3H), 3.16 (m, *J*=6.3 Hz, 1H), 2.51 (dd, *J*= 6.9, 15.3, 1H), 2.39 (dd, *J*= 6, 15, 1H), 1.83 (br s, 1H), 1.43 (d, *J*=6.3 Hz, 3H). $^{13}C\{^1H\}$ NMR ($CDCl_3$, 300MHz): δ 20.58, 41.5, 49.8, 51.3, 51.7, 127.1, 128.3, 128.6, 140.4, 173.0.

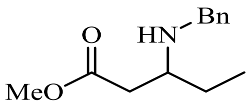


- *rac*-Methyl 3-(benzylamino)pentanoate (*rac*-**1b**) [11], from **M*t*E-b**, amber liquid, 67% yield. MS-TOF: calculated for $C_{13}H_{20}N0_2$ 222.1449, found 222.1489. $^1H$ NMR ($CDCl_3$, 300MHz): δ 7.32 (m, 5H), 3.78 (s, 2H), 3.67 (s, 3H), 2.97 (quint, J= 6 Hz, 1H), 2.46 (m, 2H), 1.53 (m, 2H) + (br, 1H), 0.92 (t, *J*= 7.2 Hz, 3H). $^{13}C\{^1H\}$ NMR ($CDCl_3$, 300MHz): δ 10.0, 26.9, 38.8, 51.0, 51.6, 55.6, 127.0, 128.3, 128.5, 140.7, 173.2.

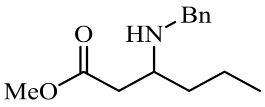

- *rac*-Methyl 3-(benzylamino)hexanoate (*rac*-**1c**) [12], from **M*t*E-c**, amber liquid, 57% yield. MS-TOF: calculated for $C_{14}H_{22}N0_2$ 236.1606, found 236.1647. $^1$H NMR ($CDCl_3$, 300MHz): δ 7.27 (m, 5H), 3.78 (s, 2H), 3.67 (s, 3H), 3.03 (quint, *J*= 6.3 Hz, 1H), 2.46 (d, *J*= 6.3 Hz, 2H), 1.43 (m, 4H) + (br, 1H), 0.91 (t, *J*= 6.9 Hz, 3H). $^{13}$C{$^1$H} NMR ($CDCl_3$, 300MHz): δ 14.3, 19.1, 36.7, 39.2, 51.1, 51.6, 54.1, 127.0, 128.2, 128.5, 140.7, 173.2.

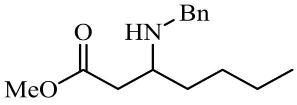

- *rac*-Methyl 3-(benzylamino)heptanoate (*rac*-**1d**), from **M*t*E-d**, amber liquid, 79% yield. MS-TOF: calculated for $C_{15}H_{24}N0_2$ 250.1762, found 250.1803. $^1$H NMR ($CDCl_3$, 300MHz): δ 7.28 (m, 5H), 3.78 (s, 2H), 3.67 (s, 3H), 3.01 (quint, *J*= 6.3 Hz, 1H), 2.46 (d, *J*= 6.0 Hz, 2H), 1.42 (m, 6H) + (br, 1H), 0.89 (t, *J*= 6.6 Hz, 3H). $^{13}$C{$^1$H} NMR ($CDCl_3$, 300MHz): δ 14.2, 22.9, 28.0, 34.2, 39.2, 51.1, 51.7, 54.4, 127.0, 128.3, 128.5, 140.7, 173.2.

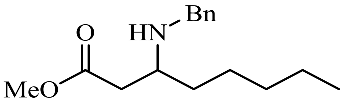

- *rac*-Methyl 3-(benzylamino)octanoate (*rac*-**1e**), from **M*t*E-e**, amber liquid, 60% yield. MS-TOF: calculated for $C_{16}H_{24}N0_2$ 264.1919, found 264.1956. $^1$H NMR ($CDCl_3$, 300MHz): δ 7.27 (m, 5H), 3.78 (s, 2H), 3.67 (s, 3H), 3.02 (quint, *J*= 6 Hz, 1H), 2.46 (d, *J*= 6 Hz, 2H), 1.46 (m, 8H) + (br, 1H), 0.89 (t, *J*= 6 Hz, 3H). $^{13}$C{$^1$H} NMR ($CDCl_3$, 300MHz): δ 14.1, 22.7, 25.5, 32.0, 34.4, 39.2, 51.1, 51.6, 54.3, 127.0, 128.2, 128.5, 140.7, 173.2.

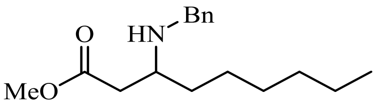

- *rac*-Methyl 3-(benzylamino)nonanoate (*rac*-**1f**), from **M*t*E-f**, amber liquid, 50% yield. MS-TOF: calculated for $C_{17}H_{28}N0_2$ 278.2075, found 278.1156. $^1$H NMR ($CDCl_3$, 300MHz): δ 7.26 (m, 5H), 3.78 (s, 2H), 3.67 (s, 3H), 3.01 (quint, *J*= 6 Hz, 1H), 2.46 (d, *J*= 6.3 Hz, 2H), 1.46 (m, 10H) + (br, 1H), 0.88 (t, *J*= 5.7 Hz, 3H). $^{13}$C{$^1$H} NMR ($CDCl_3$, 300MHz): δ 14.2, 22.7, 25.8, 29.5, 31.9, 34.5, 39.2, 51.1, 51.6, 54.4, 127.0, 128.3, 128.5, 140.7, 173.2.

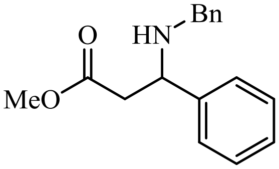

- *rac*-Methyl 3-(benzylamino)-3-phenylpropanoate (*rac*-**1g**) [4], amber liquid. MS-TOF: calculated for $C_{17}H_{20}N0_2$ 270.1494, found 270.1501. 79% yield. $^1$H NMR ($CDCl_3$, 400MHz): δ 7.39-7.25 (m, 10H), 4.15 (dd, *J*= 5.2, 8.6 Hz, 1H), 3.71-3.55 (m, 5H), 2.77 (dd, *J*= 8.8, 15.6 Hz, 1H), 2.55 (dd, *J*= 5.2, 15.6 Hz, 1H). $^{13}$C{$^1$H} NMR ($CDCl_3$, 400MHz): δ 42.8, 51.3, 58.8, 65.3, 126.9, 127.1, 127.5, 128.1, 128.3, 128.6, 140.2, 142.4, 172.2.

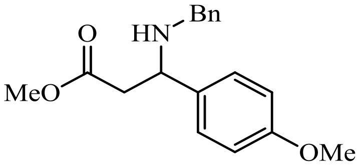

- *rac*-Methyl 3-(benzylamino)-3-(*p*-metoxy)phenylpropanoate (*rac*-**1h**) [13], amber liquid, 50% yield (0.03 mmol). $^1$H NMR ($CDCl_3$, 300MHz): δ 7.33-7.23 (m, 7H), 6.85 (d, *J*=9 Hz, 2H), 4.06 (q, *J*= 5.4 Hz, 1H), 3.81 (s, 3H), 3.67-3.50 (m, 5H), 2.76-2.57 (m, 2H), 1.97 (br s, 1H). $^{13}$C{$^1$H} NMR ($CDCl_3$, 300MHz): δ 43.1, 51.4, 51.8, 55.4, 58.2, 114.1, 127.0, 128.3, 128.5, 134.6, 140.4, 159.0, 172.6.

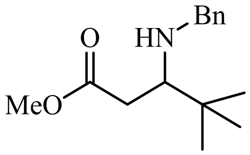

- *rac*-Methyl 3-(benzylamino)-4,4-dimethylpentanoate (*rac*-**1i**) [4], amber liquid. HRMS(ES$^+$): calculated for $C_{15}H_{24}N0_2$ 250.3616, found 250.1812. $^1$H NMR ($CDCl_3$, 400MHz): δ 7.32-7.23 (m, 5H), 3.90-3.68 (m, 5H), 2.80 (q, *J*= 4.2 Hz, 1H), 2.57 (dd, J=4.2, 14.7 Hz, 1H), 2.29 (dd, *J*= 8.4, 14.7 Hz, 1H), 1.29 (br s, 1H), 0.92 (s, 9H). $^{13}$C{$^1$H} NMR ($CDCl_3$, 100MHz): δ 26.7, 35.6, 37.0, 51.8, 54.3, 64.1, 127.0, 128.4, 141.2, 174.3.

## 3) General method for the synthesis of *N*-benzylated-β$^3$-amino acids

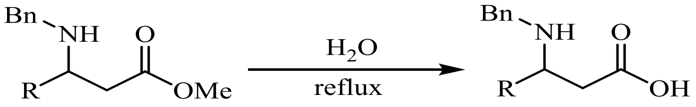

*rac*-**1** → *rac*-**2**

Representative example for the synthesis of *N*-benzylated-β$^{3}$-amino acids[5]: The appropriate *N*-benzylated-β$^{3}$-amino acid methyl ester is placed in a round flask equipped with a stir bar and 10 mL of water, 1 equivalent of NaOH is added when required (substrates **2c** to **2j**), the reaction was refluxed for 4 h. The solution was neutralized, the solvent was evaporated under vacuum and the product was washed with $CH_2Cl_2$, filtered and decanted with MeOH to obtain an off white solid.

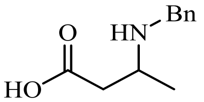

- *rac*-3-(*N*-Benzylamino)butanoic acid (*rac*-**2a**) [4], white off solid, 86% yield. mp 170-171 °C (170-171°C [4]). MS-TOF: calculated for $C_{11}H_{15}N0_2$ 194.1136, found 194.1175. $^1$H NMR ($D_2O$, 400MHz): δ 7.42 (s, 5H), 4.18 (q, *J*= 16.8 Hz, 2H), 3.52 (m, *J*= 6.4 Hz, 1H), 3.28 (br s, 1H), 2.59 (dd, *J*=4.8, 13 Hz, 1H), 2.54 (dd, *J*=4.5, 12.8 Hz, 1H), 1.32 (d, *J*= 6.4 Hz, 3H). $^{13}$C{$^1$H} NMR ($D_2O$, 400MHz): δ 15.9, 38.2, 48.1, 51.2, 129.3, 129.6, 130.9, 176.4.

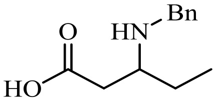

- *rac*-3-(*N*-Benzylamino)pentanoic acid (*rac*-**2b**) [14], 89% yield. white solid. mp 167-169°C (169-170°C [14]). MS-TOF: calculated for $C_{11}H_{15}N0_2$ 208.1293, found 208.1331. $^1$H NMR ($D_2O$, 400MHz): δ 7.40 (s, 5H), 4.17 (q, *J*= 9, 1 Hz, 2H), 3.31 (m, 1H), 2.56 (dd, *J*=3.6, 12.9 Hz, 1H), 2.40 (dd, *J*=5.4, 12.7 Hz, 1H), 1.78 (m, 1H), 1.59 (m, 1H),

0.88 (t, *J*= 7.6Hz, 3H). $^{13}C\{^1H\}$ NMR ($D_2O$, 400MHz): δ 8.8, 23.3, 35.3, 47.9, 56.7, 129.3, 129.5, 131.2, 178.2.

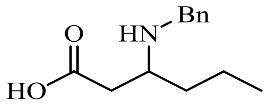

- *rac*-3-(*N*-Benzylamino)hexanoic acid (*rac*-**2c**) [14], 97% yield. white solid. mp 156-159°C (160-162°C [14]). MS-TOF: calculated for $C_{13}H_{20}N0_2$ 222.1449, found 222.1488. $^1H$ NMR ($D_2O$, 400MHz): δ 7.40 (s, 5H), 4.18 (q, *J*= 10.2 Hz, 2H), 3.55 (m, 1H), 2.56 (dd, *J*= 3.6, 12.6 Hz, 1H), 2.40 (dd, *J*= 5.7, 12.6 Hz, 1H), 1.70 (m, 1H), 1.55 (m, 1H), 1.30 (m, 2H), 0.83 (t, *J*= 5.7Hz, 3H). $^{13}C\{^1H\}$ NMR ($D_2O$, 400MHz): δ 12.9, 18.0, 32.3, 35.9, 47.8, 55.1, 129.3, 129.4, 129.5, 131.4, 178.3.

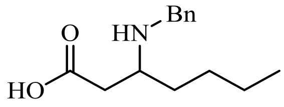

- *rac*-3-(*N*-Benzylamino)heptanoic acid (*rac*-**2d**) [15], 84% yield. white solid. mp 152-155°C. MS-TOF: calculated for $C_{14}H_{22}N0_2$ 236.1606, found 236.1644. $^1H$ NMR ($D_2O$, 400MHz): δ 7.26 (s, 5H), 4.0 (t, *J*= 10.2 Hz, 2H), 3.32 (m, 1H), 2.68 (dd, *J*= 4.8, 17.8 Hz, 1H), 2.28 (dd, *J*= 6.8, 18 Hz, 1H), 1.60 (m, 1H), 1.47 (m, 1H), 1.08 (m, 4H), 0.63 (t, *J*= 6.4 Hz, 3H). $^{13}C\{^1H\}$ NMR ($D_2O$, 400MHz): δ 12.9, 21.4, 26.4, 29.5, 34.0, 48.5, 54.1, 129.1, 129.5, 129.7, 130.3, 137.9.

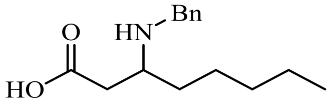

- *rac*-3-(*N*-Benzylamino)octanoic acid (*rac*-**2e**), 80% yield. white solid. mp 150-152°C. MS-TOF: calculated for $C_{15}H_{24}N0_2$ 250.1762, found 250.1803. $^1H$ NMR ($D_2O$, 400MHz): δ 7.25 (s, 5H), 4.03 (s, 2H), 3.32 (m, 1H), 2.67 (dd, *J*= 4.8, 18 Hz, 1H), 2.28 (dd, *J*= 6.8,

17.6 Hz, 1H), 1.58 (m, 1H), 1.46 (m, 1H), 1.03 (m, 6H), 0.60 (t, *J*= 6.4 Hz, 3H). $^{13}C\{^{1}H\}$ NMR ($D_2O$, 400MHz): δ 13.0, 21.4, 23.9, 29.7, 30.3, 34.0, 48.5, 54.1, 129.1, 129.5, 129.7, 130.2, 137.9.

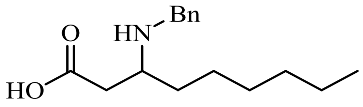


- *rac*-3-(*N*-Benzylamino)nonanoic acid (*rac*-**2f**), 83% yield. white solid. mp 151-153°C MS-TOF: calculated for $C_{16}H_{26}N0_2$ 264.1919, found 264.2005. $^{1}H$ NMR ($D_2O$, 400MHz): δ 7.36 (s, 5H), 4.18 (s, 2H), 3.41 (m, 1H), 2.76 (dd, *J*= 4.8, 17.8 Hz, 1H), 2.67 (dd, *J*= 6.8, 17.6 Hz, 1H), 1.69 (m, 1H), 1.56 (m, 1H), 1.16 (m, 8H), 0.72 (t, *J*= 6 Hz, 3H). $^{13}C\{^{1}H\}$ NMR ($D_2O$, 400MHz): δ 13.2, 21.7, 24.2, 27.8, 29.7, 30.6, 34.1, 48.6, 54.0, 129.2, 129.7, 129.8, 130.3, 174.0.

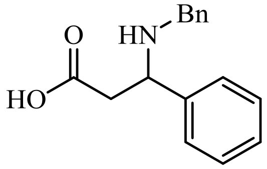


- *rac*-3-(*N*-Benzylamino)-3-phenylpropanoic acid (*rac*-**2g**) [4], 80% yield. white solid. mp 149-151°C (148-151°C [4]).$^{1}H$ NMR ($CD_3OD$, drops $NH_4OH$, 200MHz): δ 7.42-7.22 (m, 10H), 4.95 (dd, *J*= 4.4, 10.4, 1H), 3.33 (s, 2H), 2.63 (dd, *J*= 10.4, 16.8, 1H), 2.48 (dd, *J*= 4.4, 17.2, 1H), $^{13}C\{^{1}H\}$ NMR ($CD_3OD+NH_4OH$, 50MHz): δ 45.3, 50.9, 60.3, 126.6, 126.9, 127.3, 128.0, 128.1, 139.3, 142.6, 178.3.

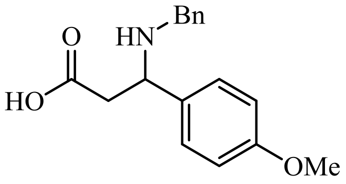


- *rac*-3-(*N*-Benzylamino)-3-(*p*-methoxy)phenylpropanoic acid (*rac*-**2h**), 54% yield. white solid. mp 172-174°C. MS-TOF: calculated for $C_{16}H_{25}N0_2$ 263.1885, found 263.2005. $^{1}H$ NMR ($CD_3OD$, 400MHz): δ 7.26 (d, *J*= 9.6 Hz, 7H), 7.04 (d, *J*= 8 Hz, 2H), 4.45 (dd, J=

3.2, 10.2 Hz 1H), 2.76 (dd, *J*= 4.8, 17.8 Hz, 1H), 4.00 (dd, *J*= 13.2, 25.2 Hz, 2H), 3.85 (s, 3H), 2.86 (dd, *J*= 10.8, 16.6 Hz, 1H), 2.65 (dd, *J*= 3.6, 16.8 Hz, 2H). $^{13}C\{^1H\}$ NMR ($CD_3OD$, 400MHz): δ 38.5, 48.1, 54.4, 59.3, 14.4, 126.4, 128.9, 128.9, 129.0, 129.1, 131.8, 160.7, 176.0.

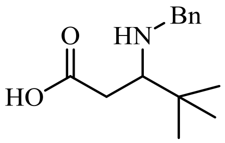


- *rac*-3-(*N*-Benzylamino)-4,4-dimethylpentanoic acid (*rac*-**2i**) [4], 88% yield. white solid. mp 152-153°C (150-153°C [4]). HRMS ($ES^+$): calculated for $C_{14}H_{22}N0_2$ 236.1651, found 236.1638. $^1H$ NMR ($D_2O$) δ 7.27 (s, 5H), 4.28 (m, 1H), 4.04 (m, 1H), 3.14 (m, 1H), 2.69 (m, 1H), 2.53 (m, 1H), 0.75 (m, 9H) $^{13}C\{^1H\}$ NMR ($CD_3OD$, 100MHz): δ 24.6, 32.0, 33.3, 50.6, 62.6, 129.1, 129.8, 130.2, 174.3.

## 4) General method for the enzymatic resolution using CALB.

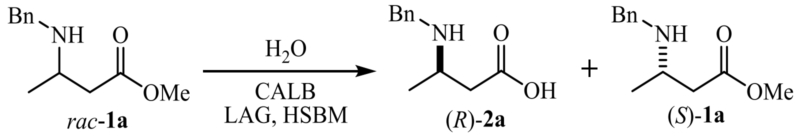


Representative example for the enzymatic resolution using CALB and *rac*-**1a**: 82 mg of substrate *rac*-**1a** (0.4 mmol), 3.6 μL of water (0.2 mmol) and 0.2 mL of 2M2B were placed in an Agate jar (12 mm of diameter) with an Agate ball (6 mm of diameter, 480 mg of weight), 40 mg of enzyme (CALB) were aggregated and the reactor was closed and placed in a Mixer Mill MM200 programed to carry out the reaction at 25 Hz during 30 min. Once the reaction is finished the content was extracted with methanol and transferred to a Falcon tube (50 mL), the solution was centrifuged at 3500 rpm for 5 min twice; the supernatant was collected and concentrated for his purification with silica gel, a mixture of hexanes and ethyl acetate was used as mobile phase for recovering the raw material and methanol and $CH_2Cl_2$ for the purification of the product. Methyl 3-(benzylamino)butanoic acid (*R*)-**2a** was obtain as a white solid (49% yield). In order to avoid contamination by the wear of the stainless-steel reactors we decided to use Agate reactors.

**Table S1: Search of the best parameters for the enzymatic hydrolysis resolution under HSBM.**

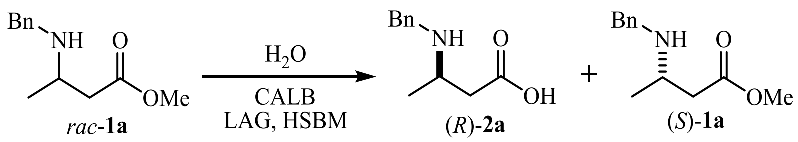


| entry[a] | LAG | frequency (Hz) | time (h) | (S)-**1a** recovered[b] (%) | (S)-**1a** *ee*[c] (%) | (S)-**1a** $[\alpha]_D^{25}$[d] | (R)-**2a** yield[b] (%) | (R)-**2a** *ee*[c] (%) | (R)-**2a** $[\alpha]_D^{25}$[e] | *c*[f] (%) | *E*[g] |
|---|---|---|---|---|---|---|---|---|---|---|---|
| 1 | 2M2B | 25 | 0.5 | 51 | 99 | 20.4 | 49 | 80 | -31.7 | 55 | 46 |
| 2 | 2M2B | 15 | 0.5 | 70 | 89 | 22.0 | 30 | 77 | -33.1 | 54 | 23 |
| **3** | **2M2B** | **15** | **1** | **51** | **99** | **22.5** | **49** | **95** | **-30.0** | **51** | **>200** |
| 4 | Hexane | 15 | 1 | 40 | 97 | 23.3 | 60 | 86 | -20.4 | 53 | 55 |
| 5 | AcOEt | 15 | 1 | 86 | 69 | 10.3 | 13 | 95 | -30.1 | 42 | 81 |
| 6 | DIPE | 15 | 1 | 76 | 92 | 17.3 | 39 | 88 | -25.6 | 51 | 51 |
| 7 | Toluene | 15 | 1 | 75 | 72 | 12.6 | 37 | 93 | -29.5 | 44 | 60 |
| 8 | Dioxane | 15 | 1 | 73 | 79 | 16.7 | 47 | 95 | -30.3 | 45 | 94 |
| 9 | IPA | 15 | 1 | 82 | 48 | 7.4 | 21 | 95 | -29.8 | 34 | 63 |
| 10 | $CH_3CN$ | 15 | 1 | 65 | 65 | 9.8 | 29 | 95 | -30.0 | 41 | 77 |
| 11 | - | 15 | 1 | 58 | 95 | 16.6 | 41 | 92 | -28.2 | 51 | 89 |
| 12 | - | 25 | 1 | 58 | 93 | 16.5 | 42 | 86 | -31.2 | 52 | 45 |
| 13[h] | - | 15 | 1 | 68 | 74 | 13.5 | 31 | 80 | -27.0 | 48 | 20 |
| 14[i] | 2M2B | 15 | 1 | - | - | - | 92 | *rac* | - | - | - |
| 15[j] | 2M2B | 15 | 1 | 89 | *rac* | - | - | - | - | - | - |

[a]Reactions were carried out using the general method. [b]Determinated after purification by flash chromatography. [c]Determined by HPLC with chiral stationary phase. [d]Using $CH_3Cl$ and c=0.33, [e]Using MeOH and c=0.33, [f]Calculated from $c = ee_s/(ee_s + ee_p)$. [g]$E = \ln[1-c(1+ee_p)]/\ln[1-c(1-ee_p)]$. [h]0.25 equivalents of water were used. [i]1 equivalent of water were used. [j] water-free conditions. [k]Enzyme with a pretreatment (Milling for 1h at 15 Hz). [l]Pretreatment was carried out using 0.2mL of LAG.

**Table S2: Substrate scope for the enzymatic resolution of N-benzylated-β$^3$-amino esters.**

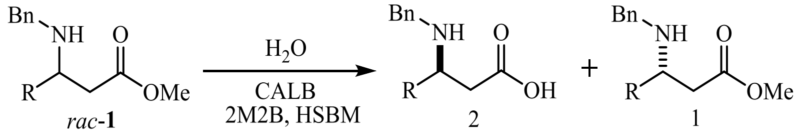


| entry[a] | *rac-1* | R | Substrate-1 recovered[b] (%) | Substrate-1 *ee*[c] (%) | Substrate-1 $[\alpha]_D^{25}$ | 2 yield[b] (%) | 2 *ee*[c] (%) | 2 $[\alpha]_D^{25}$ | *c*[d] (%) | *E*[e] | a.c.[f] |
|---|---|---|---|---|---|---|---|---|---|---|---|
| 1 | 1b | $CH_3$-($CH_2$)- | 51 | 91 | 4.5 | 49 | 97 | -36.5 | 48 | >200 | *R* |
| 2 | 1c | $CH_3$-($CH_2$)$_2$- | 53 | 84 | 2.1 | 43 | 98 | -45.2 | 46 | >200 | *R* |
| 3 | 1d | $CH_3$-($CH_2$)$_3$- | 68 | 23 | 2.0 | 29 | 94 | -35.3 | 20 | 40 | *R* |
| 4[g] | 1d | $CH_3$-($CH_2$)$_3$- | 66 | 57 | 0.2 | 25 | 94 | -33.3 | 38 | 58 | *R* |
| 5[h] | 1d | $CH_3$-($CH_2$)$_3$- | 51 | 30 | 2.7 | 41 | 85 | -40.5 | 26 | 17 | *R* |
| 6 | 1e | $CH_3$-($CH_2$)$_4$- | 74 | 16 | 1.8 | 24 | 94 | -40.0 | 15 | 38 | *R* |
| 7[g] | 1e | $CH_3$-($CH_2$)$_4$- | 76 | 27 | 0.8 | 22 | 82 | -30.2 | 25 | 13 | *R* |
| 8[h] | 1e | $CH_3$-($CH_2$)$_4$- | 82 | 27 | 2.7 | 10 | 76 | -13.0 | 26 | 10 | *R* |
| 9 | 1f | $CH_3$-($CH_2$)$_5$- | 79 | 13 | 0.8 | 18 | 91 | -39.7 | 13 | 24 | *R* |
| 10[g] | 1f | $CH_3$-($CH_2$)$_5$- | 85 | 21 | 1.1 | 11 | 82 | -28.5 | 20 | 12 | *R* |
| 11[h] | 1f | $CH_3$-($CH_2$)$_5$- | 84 | 18 | 0.7 | 8 | 83 | -28.9 | 18 | 13 | *R* |
| 12[i] | 1g | Ph | 92 | 18 | 3.4 | 10 | 83 | -35.0 | 18 | 13 | *S* |
| 13[i, h] | 1g | Ph | 86 | 18 | 3.0 | 15 | 68 | -44.0 | 21 | 6 | *S* |
| 14[j] | 1g | Ph | 90 | 16 | 3.2 | 9 | 37 | -18.1 | 30 | 3 | *S* |
| 15[i, k] | 1g | Ph | 80 | 5 | 2.5 | 20 | 48 | -21.0 | 9 | 3 | *S* |
| 16 | 1h | *p*-MeO-Ph | 89 | 1 | -0.5 | 10 | 80 | -31.7 | 1 | 9 | *S* |
| 17 | 1i | *t*-Bu | 89 | 4 | -0.6 | 4 | 94 | 12.8 | 4 | 34 | *S* |
| 18[h] | 1i | *t*-Bu | 87 | 5 | -0.7 | 6 | 89 | 16.6 | 5 | 18 | *S* |

[a]Reactions were carried out using the general method. [b]Determined after purification by flash chromatography. [c]Determined by HPLC with chiral stationary phase. [d]Calculated from $c = ee_s/(ee_s + ee_p)$. [e]$E = \ln[1-c(1+ee_p)]/\ln[1-c(1-ee_p)]$. **a.c**. Absolute Configuration of product **2.** [f] Assigned by chemical comparison and HPLC with chiral stationary phase. [g] Carried out with 15 Hz during 1 h. [h] Carried out with 25 Hz during 1 h. [i] 0.75 equiv. of water were used. [j] 1 equiv. of water were used. [k] 2 equiv. of enzyme were used.

**Table S3: Recycling capacity of immobilized CALB under HSBM conditions.**

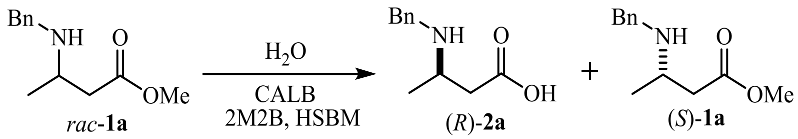


| entry[a] | rCALB (cycle) | (S)-1a recovered[b] (%) | (S)-1a *ee*[c] (%) | (S)-1a $[\alpha]_D^{25}$ | (R)-2a yield[b] (%) | (R)-2a *ee*[c] (%) | (R)-2a $[\alpha]_D^{25}$ | *c*[d] (%) | *E*[e] |
|---|---|---|---|---|---|---|---|---|---|
| 1 | - | 51 | >99 | 22.0 | 49 | 95 | -31.7 | 51 | >200 |
| 2 | 1 | 65 | 35 | 2.8 | 37 | 88 | -33.3 | 59 | 22 |
| 3 | 2 | 80 | 6 | 0.5 | 20 | 80 | -53.9 | 51 | 10 |
| 4 | 3 | 99 | 0 | 0.0 | 0 | 0 | - | - | - |
| 5 | 1 | 70 | 67 | 15.6 | 38 | 94 | -29.2 | 42 | 65 |
| 6 | 1 | 72 | 83 | 15.9 | 38 | 91 | -26.8 | 48 | 55 |

[a]Reactions were carried out using the general method. [b]Determined after purification by flash chromatography. [c]Determined by HPLC with chiral stationary phase. [d]Calculated from $c = ee_s/(ee_s + ee_p)$. [e]$E = \ln[1-c(1+ee_p)]/\ln[1-c(1-ee_p)]$.

**Table S4. Scaling-up for the enzymatic hydrolysis using the substrate *rac*-1a.**

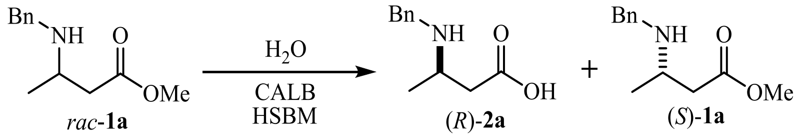

| entry[a] | substrate equivalents | (*S*)-1a | | | (*R*)-2a | | | $c^{d}$ (%) | $E^{e}$ |
|---|---|---|---|---|---|---|---|---|---|
| | | recovered[b] (%) | $ee^{c}$ (%) | $[\alpha]_D^{25}$ | yield[b] (%) | $ee^{c}$ (%) | $[\alpha]_D^{25}$ | | |
| 1 | 1 | 51 | >99 | 22.0 | 49 | 95 | -31.7 | 51 | >200 |
| 2 | 3 | 54 | 62 | 8.0 | 51 | 93 | -33.0 | 40 | 52 |
| 3 | 6 | 61 | 53 | 7.8 | 42 | 93 | -33.9 | 36 | 47 |
| 4 | 9 | 59 | 49 | 7.0 | 40 | 94 | -33.0 | 34 | 53 |

[a]Reactions were carried out using the general method but in absence of LAG. [b]Determined after purification by flash chromatography. [c]Determined by HPLC with chiral stationary phase. [d]Calculated from $c = ee_s/(ee_s + ee_p)$. [e]$E = \ln[1-c(1+ee_p)]/\ln[1-c(1-ee_p)]$.

5) $^{1}$H and $^{13}$C NMR spectra for TPA, M*t*E-b to M*t*E-d, *rac*-1a to *rac*-1j and products 2a to 2j.

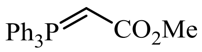


**TPA**

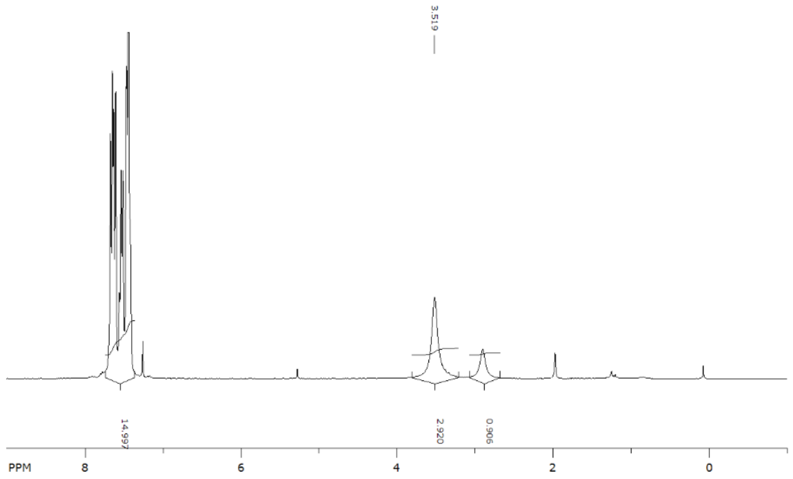


**Figure S1. $^{1}$H spectrum (300 MHz, $CDCl_3$) of**

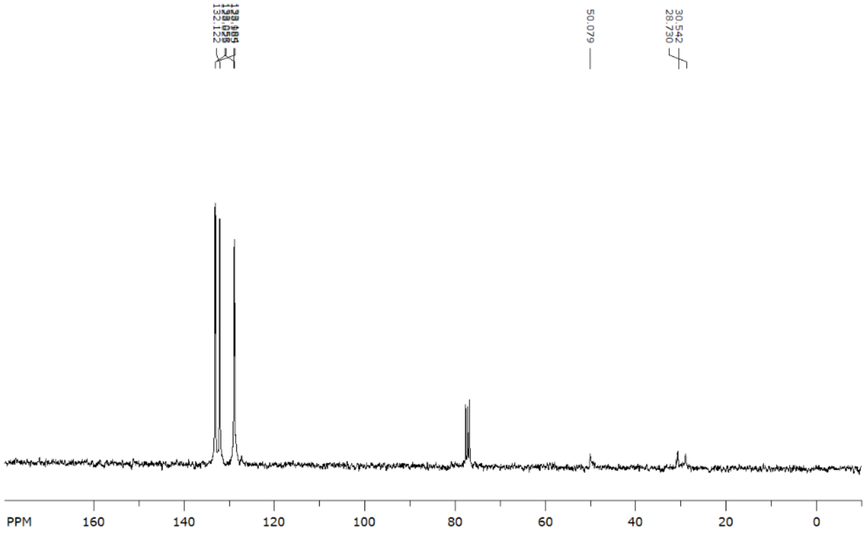


**TPAc**

**Figure S2. $^{13}$C spectrum (75 MHz, $CDCl_3$) of TPA**

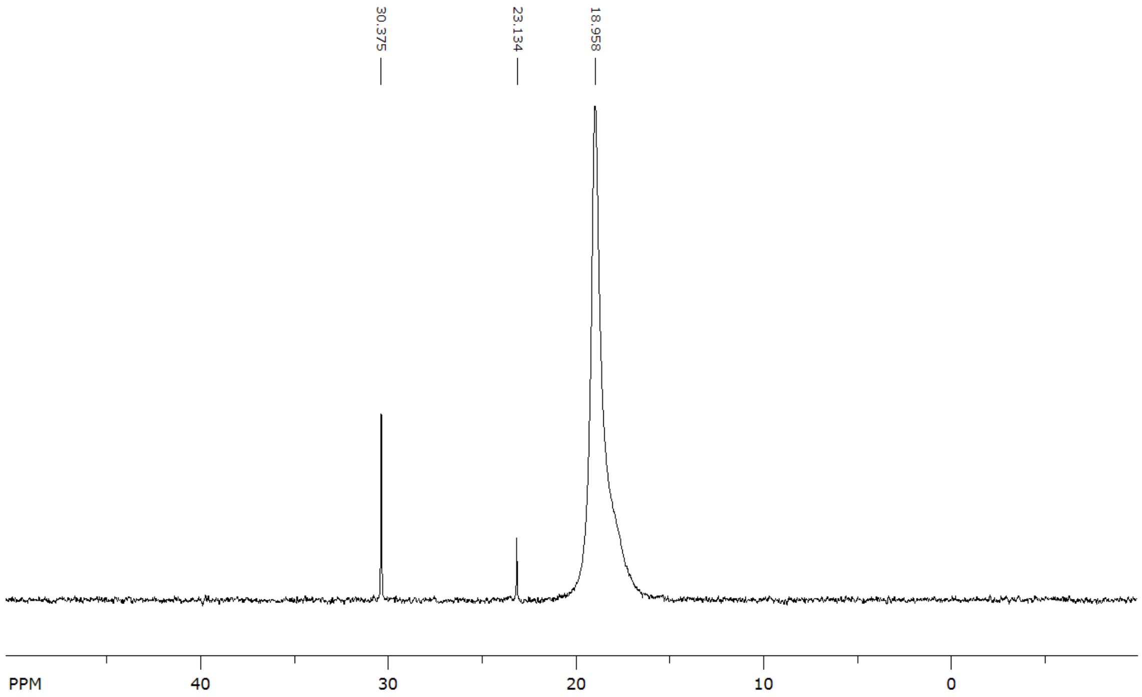


**Figure S3. $^{31}P$ spectrum (120 MHz, $CDCl_3$) of TPA**

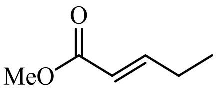

**M*t*E-b**

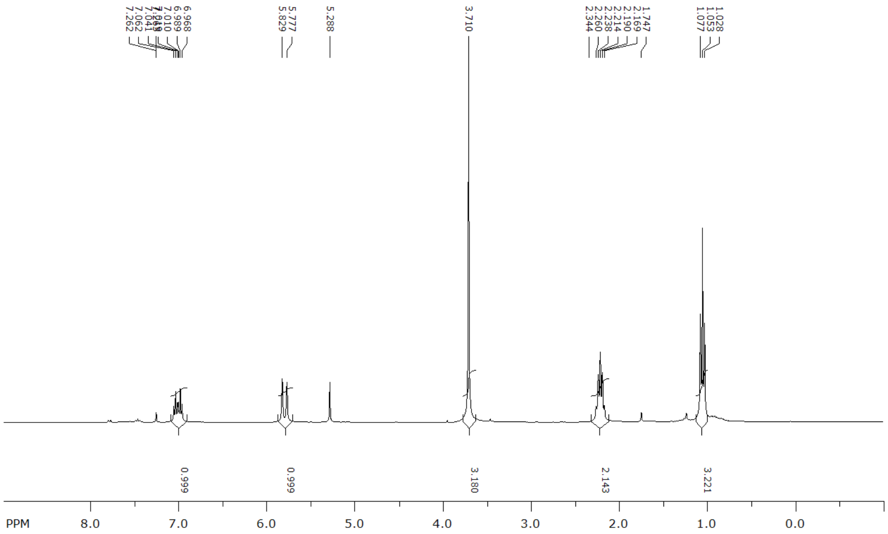


**Figure S4. $^{1}$H spectrum (300 MHz, $CDCl_3$) of M*t*E-b**

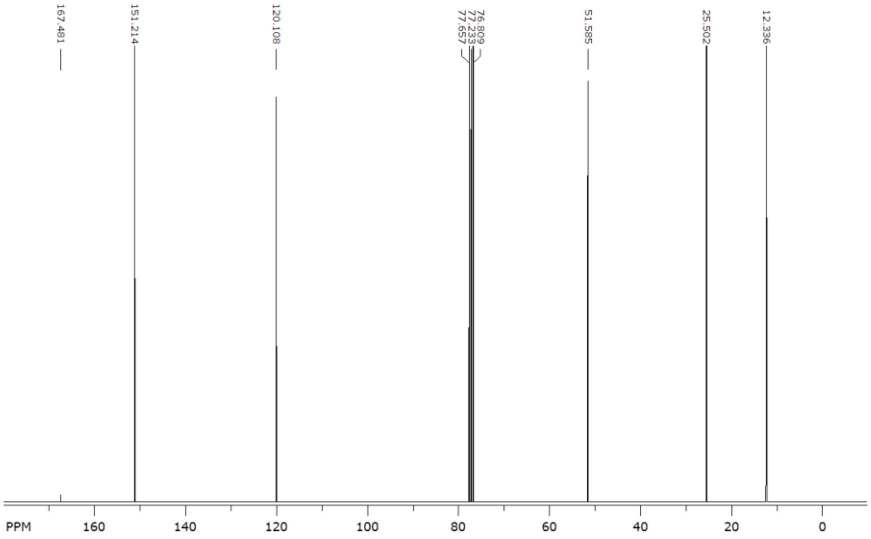


**Figure S5. $^{13}$C spectrum (75 MHz, $CDCl_3$) of M*t*E-b**

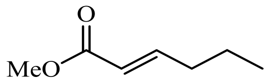

**M*t*E-c**

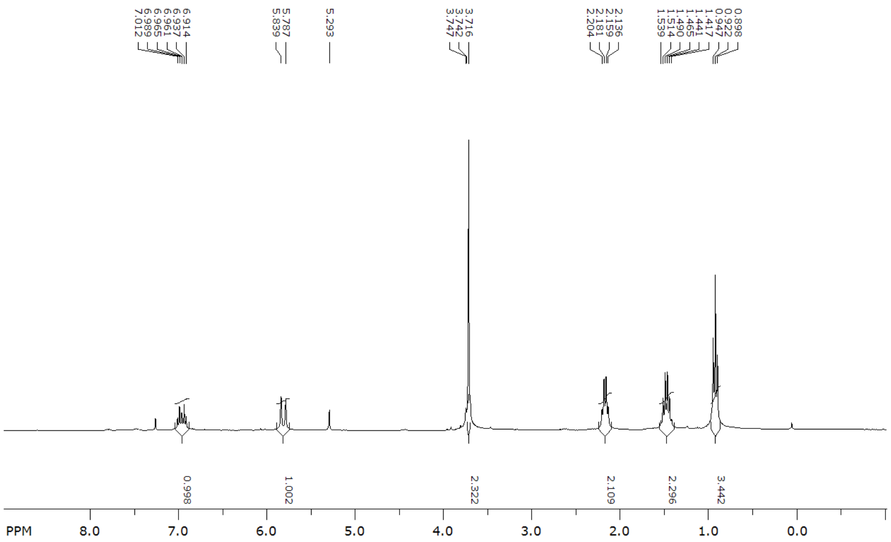


**Figure S6. $^1$H spectrum (300 MHz, $CDCl_3$) of M*t*E-c**

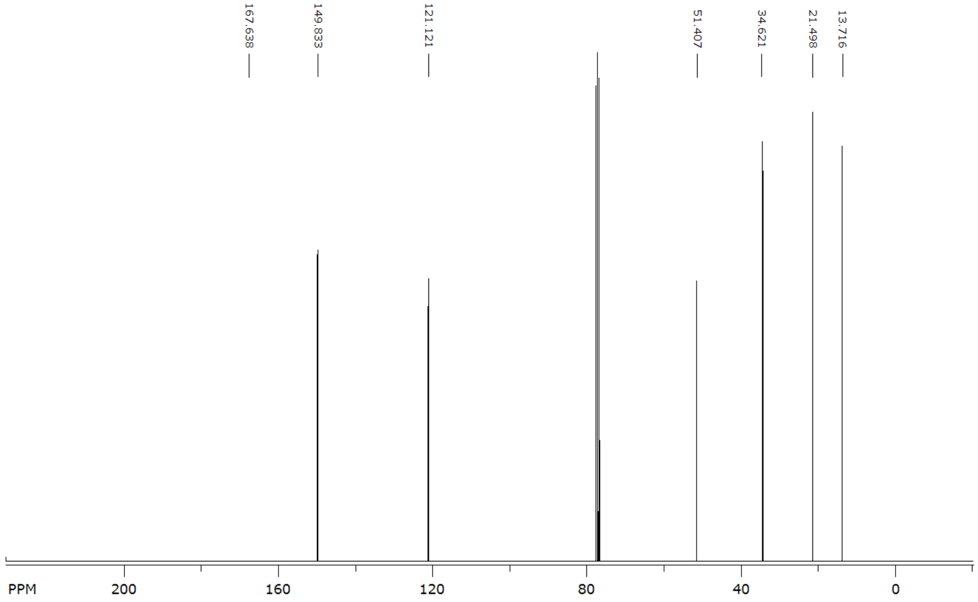


**Figure S7. $^{13}$C spectrum (75 MHz, $CDCl_3$) of M*t*E-c**

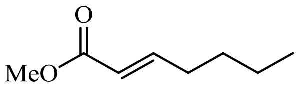


**M*t*E-c**

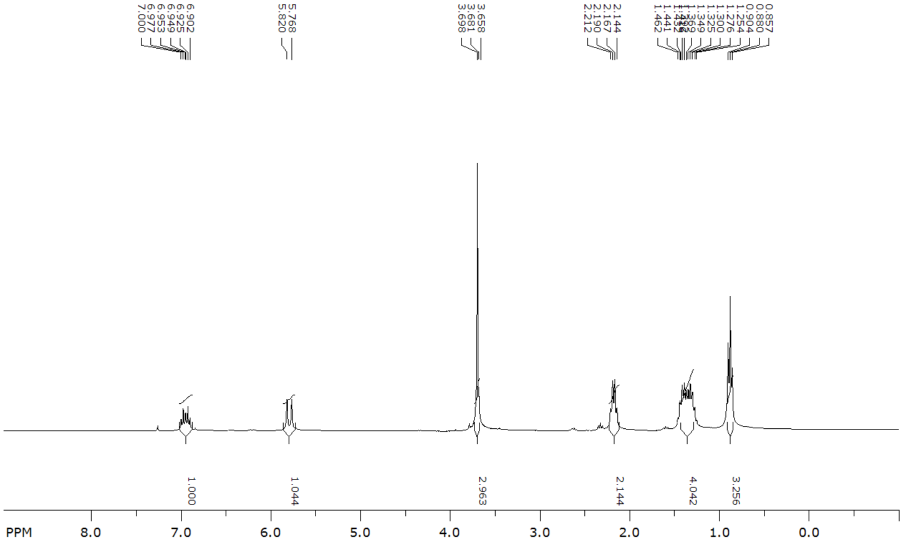


**Figure S8. $^{1}$H spectrum (300 MHz, $CDCl_3$) of M*t*E-d**

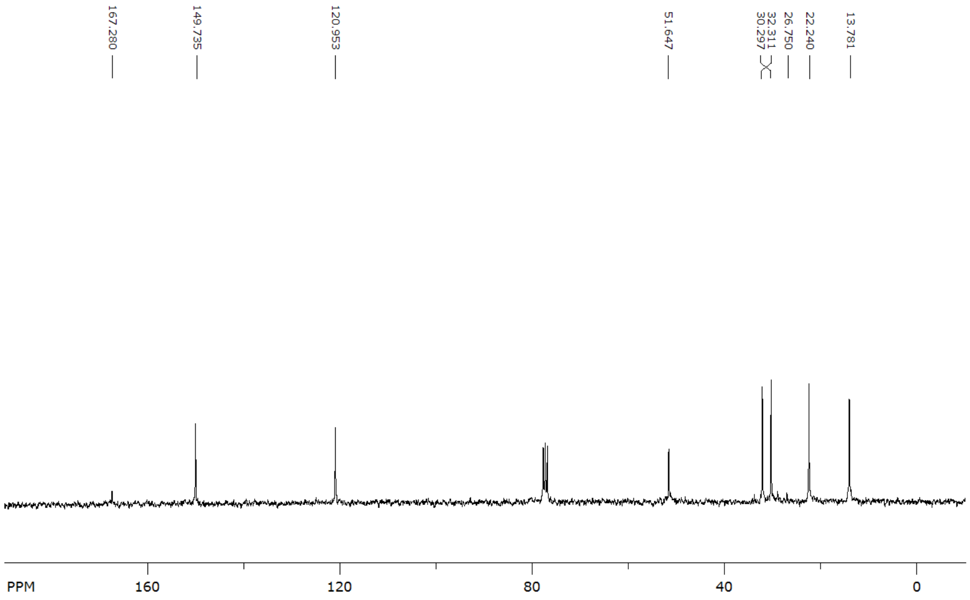


**Figure S9. $^{13}$C spectrum (75 MHz, $CDCl_3$) of M*t*E-d**

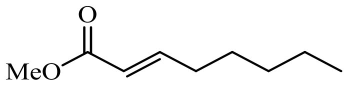


**M*t*E-d**

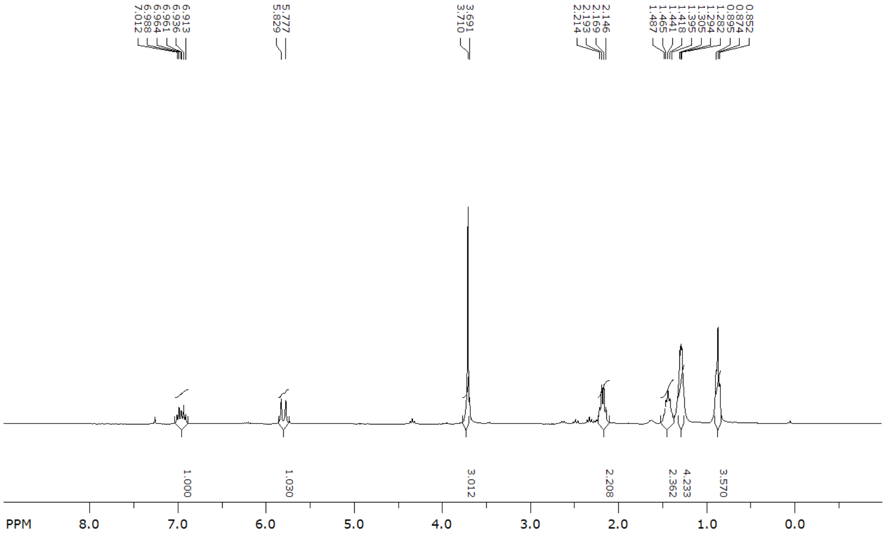


**Figure S10. $^{1}$H spectrum (300 MHz, $CDCl_3$) of M*t*E-e**

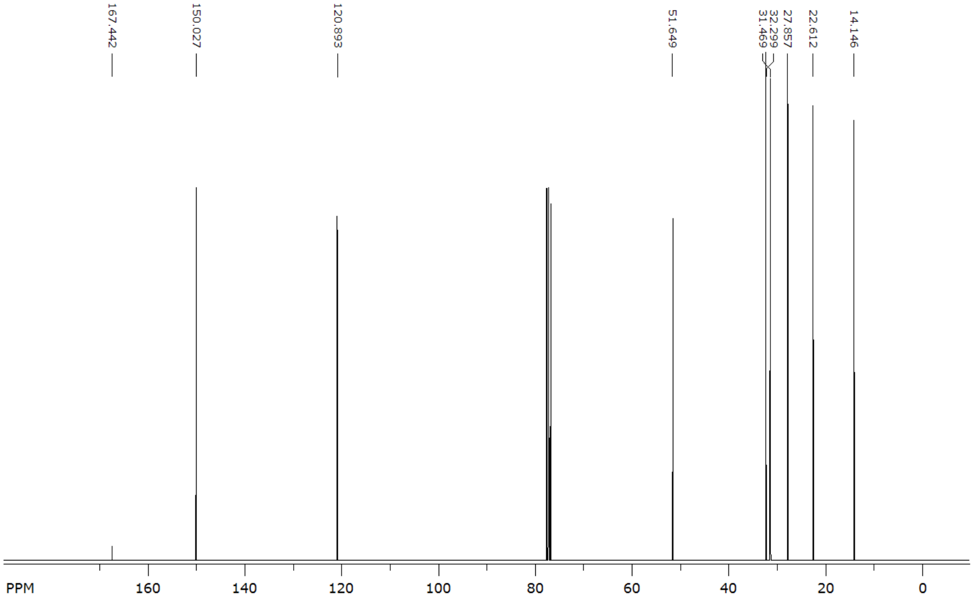


**Figure S11. $^{13}$C spectrum (75 MHz, $CDCl_3$) of M*t*E-e**

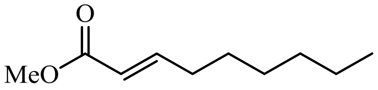


**M*t*E-e**

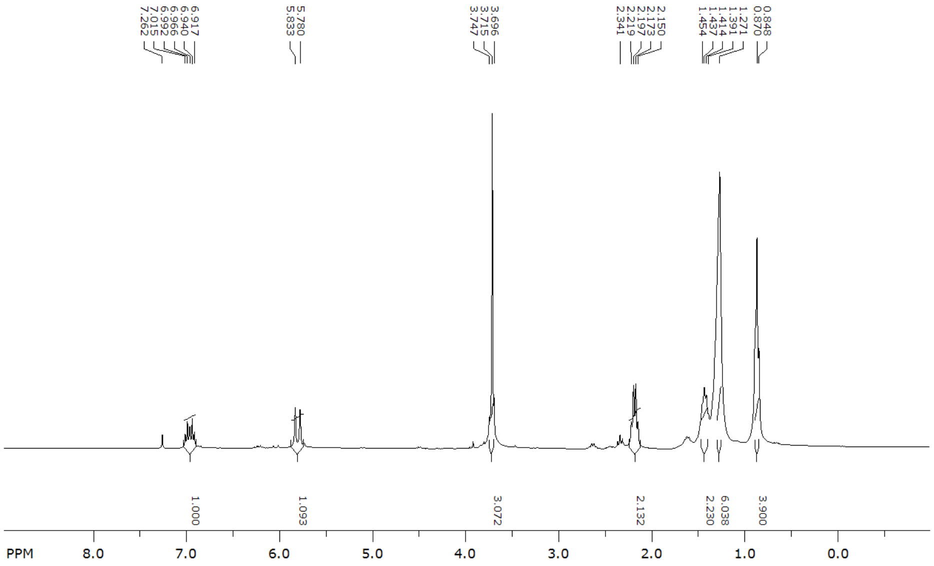


**Figure S12. $^{1}$H spectrum (300 MHz, $CDCl_3$) of M*t*E-f**

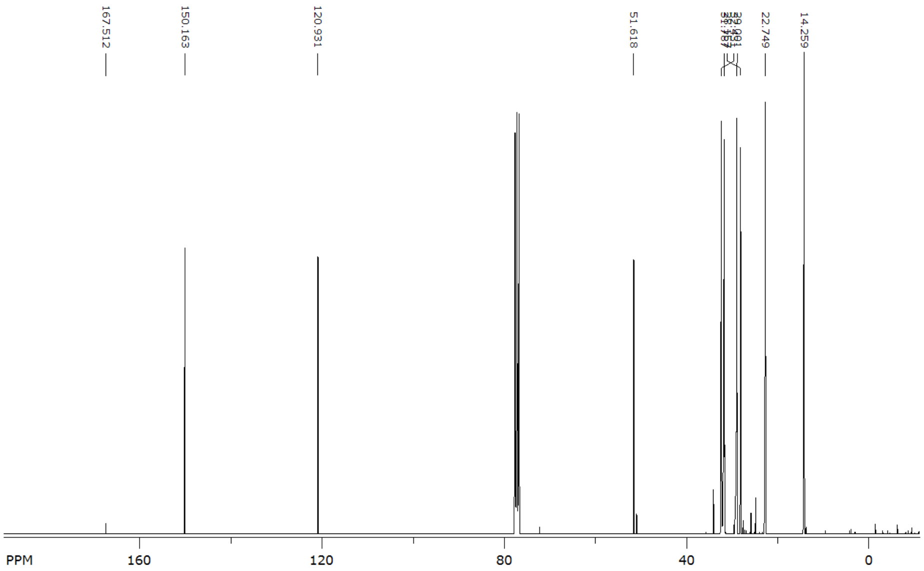


**Figure S13. $^{13}$C spectrum (75 MHz, $CDCl_3$) of M*t*E-f**

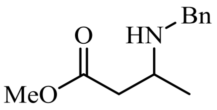


*rac*-**1a**

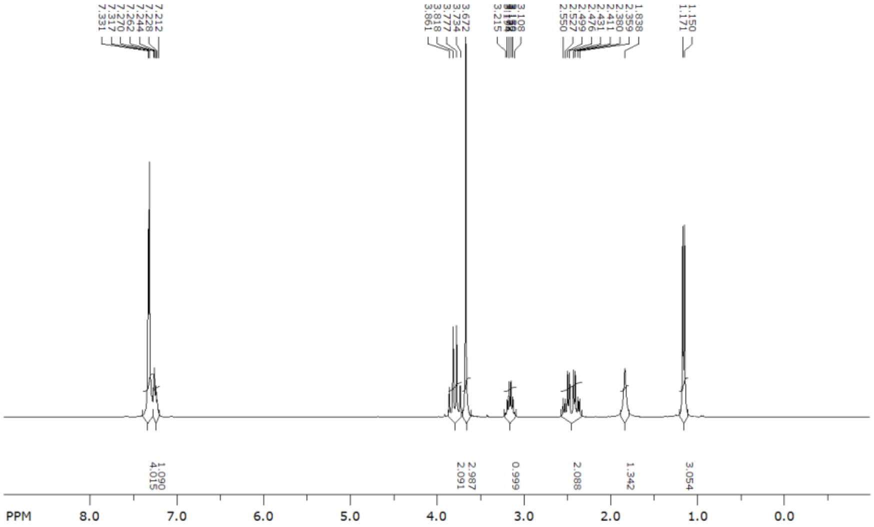


**Figure S14.** $^{1}$**H spectrum (300 MHz,** $CDCl_3$**) of *rac-1a***

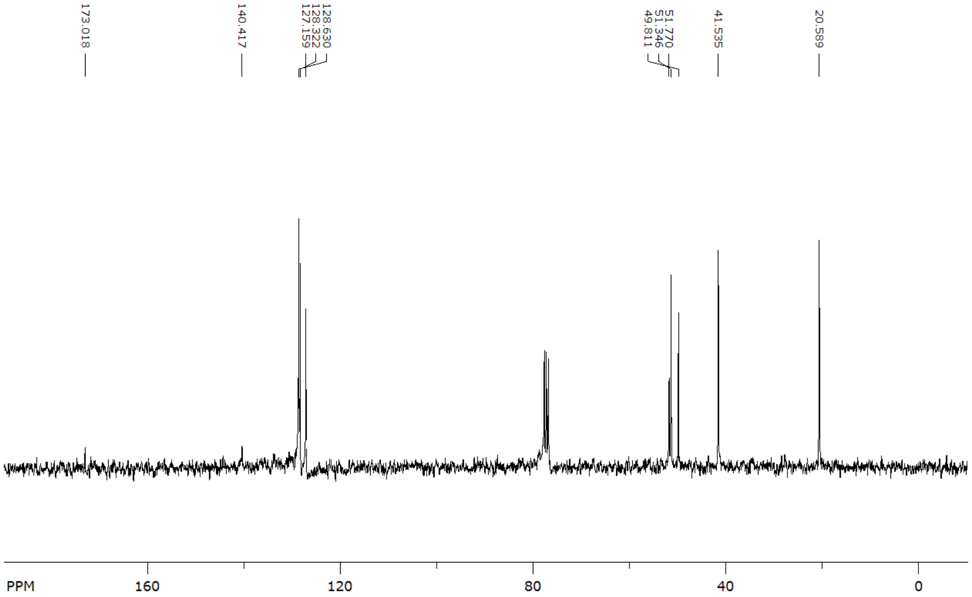


**Figure S15.** $^{13}$**C spectrum (75 MHz,** $CDCl_3$**) of *rac-1a***

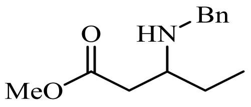


*rac*-**1b**

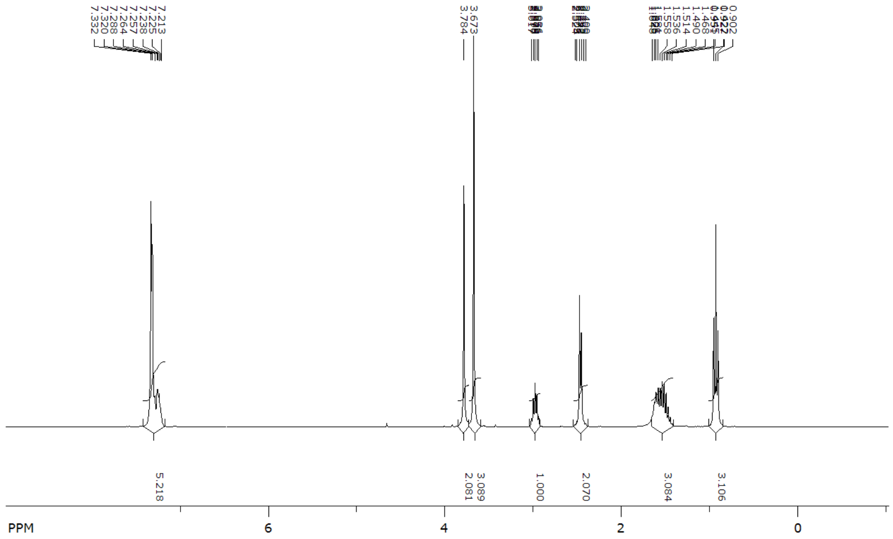

**Figure S16. $^{1}$H spectrum (300 MHz, $CDCl_3$) of *rac-1b***

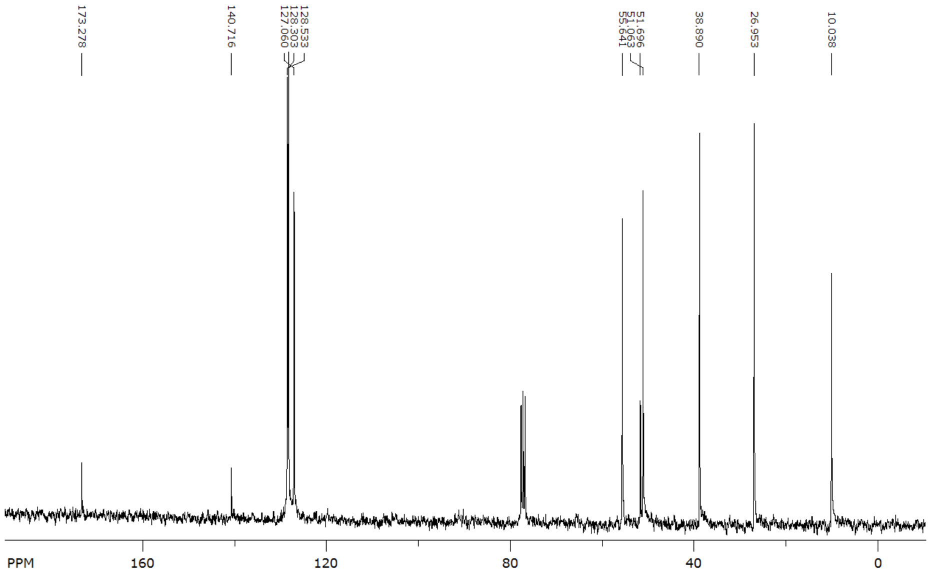

**Figure S17. $^{13}$C spectrum (75 MHz, $CDCl_3$) of *rac-1b***

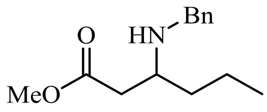

*rac*-**1c**

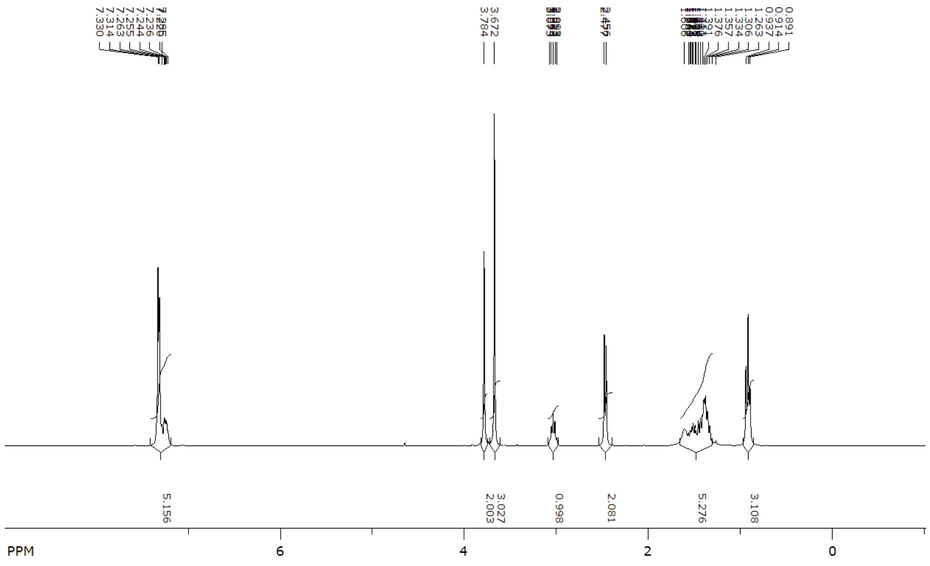

**Figure S18. $^{1}$H spectrum (300 MHz, $CDCl_3$) of *rac-1c***

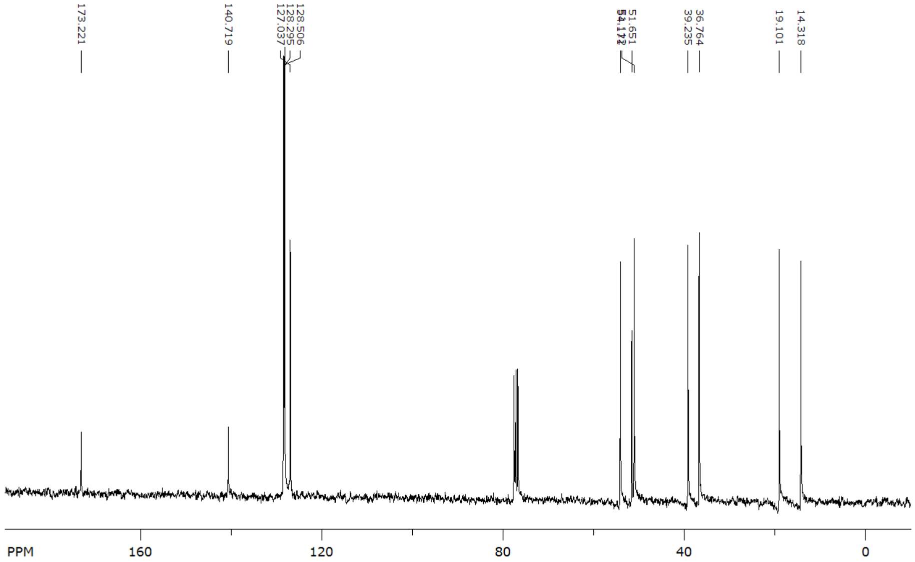

**Figure S19. $^{13}$C spectrum (75 MHz, $CDCl_3$) of *rac-1c***

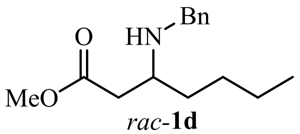


*rac*-**1d**

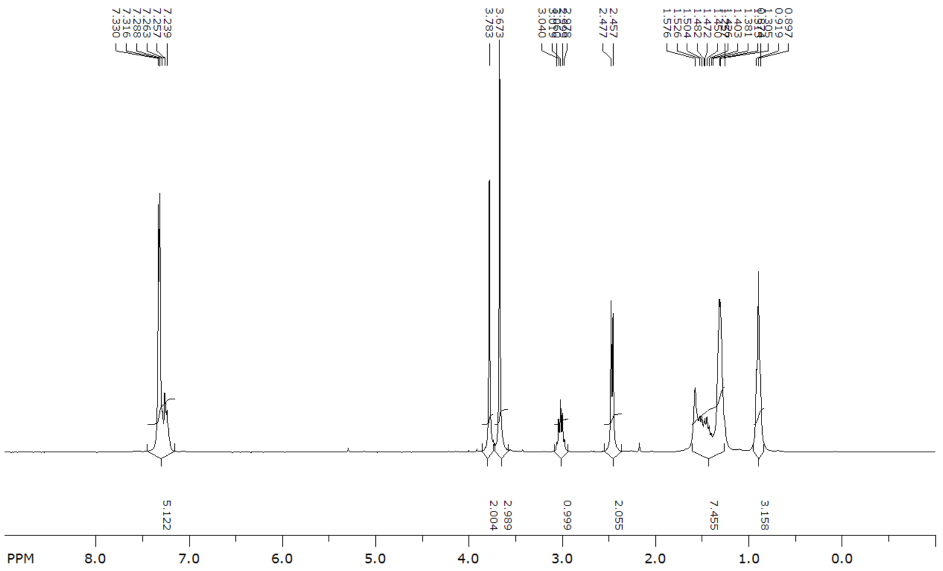


**Figure S20.** $^{1}$**H spectrum (300 MHz, $CDCl_3$) of *rac-1d***

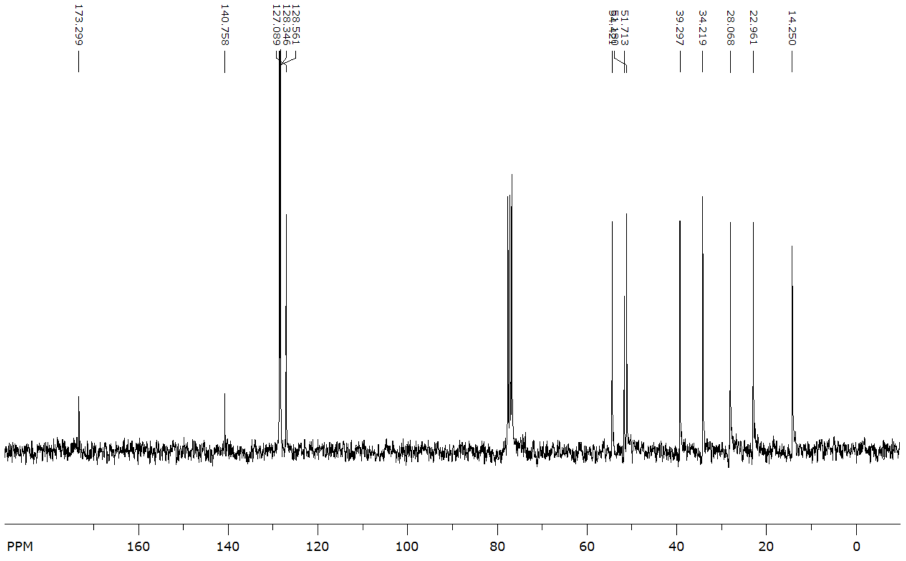


**Figure S21.** $^{13}$**C spectrum (75 MHz, $CDCl_3$) of *rac-1d***

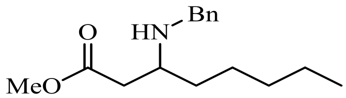


*rac*-**1e**

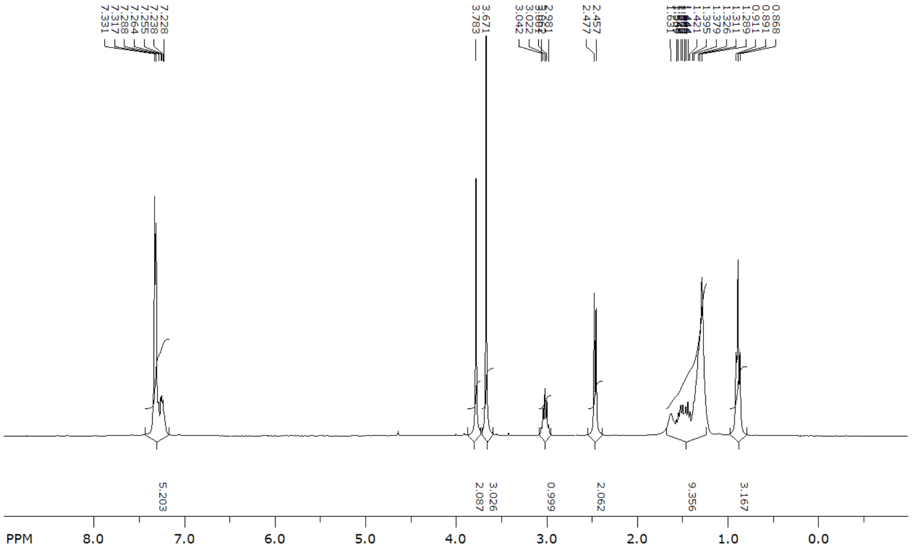

**Figure S22. $^{1}$H spectrum (300 MHz, $CDCl_3$) of *rac-1e***

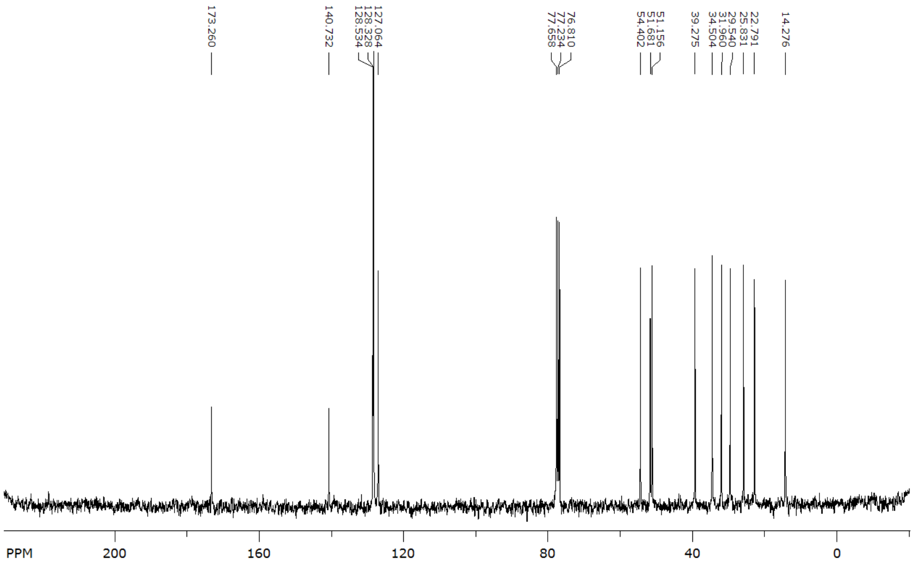

**Figure S23. $^{13}$C spectrum (75 MHz, $CDCl_3$) of *rac-1e***

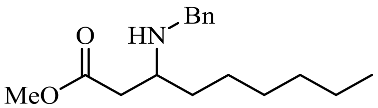

*rac*-**1f**

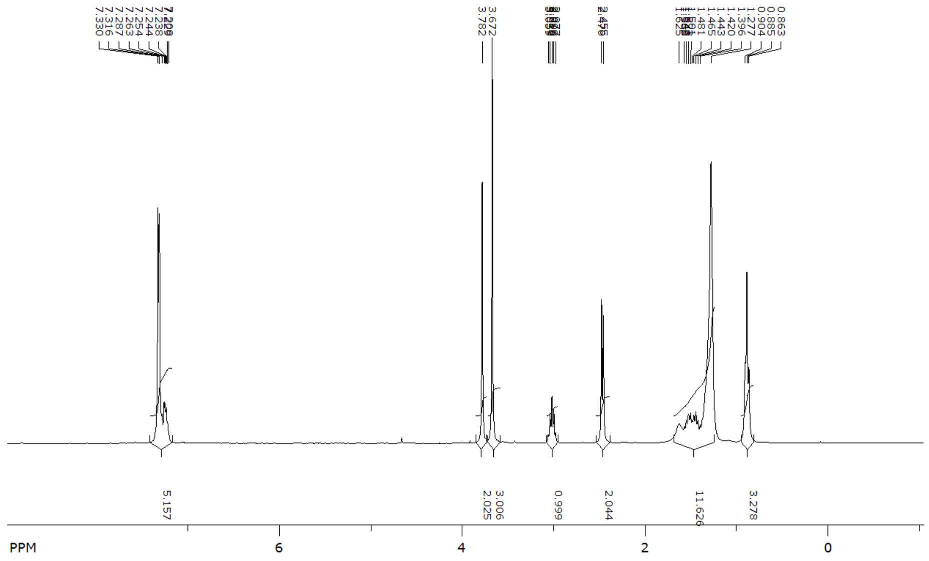

**Figure S24. $^{1}$H spectrum (300 MHz, $CDCl_3$) of *rac-1f***

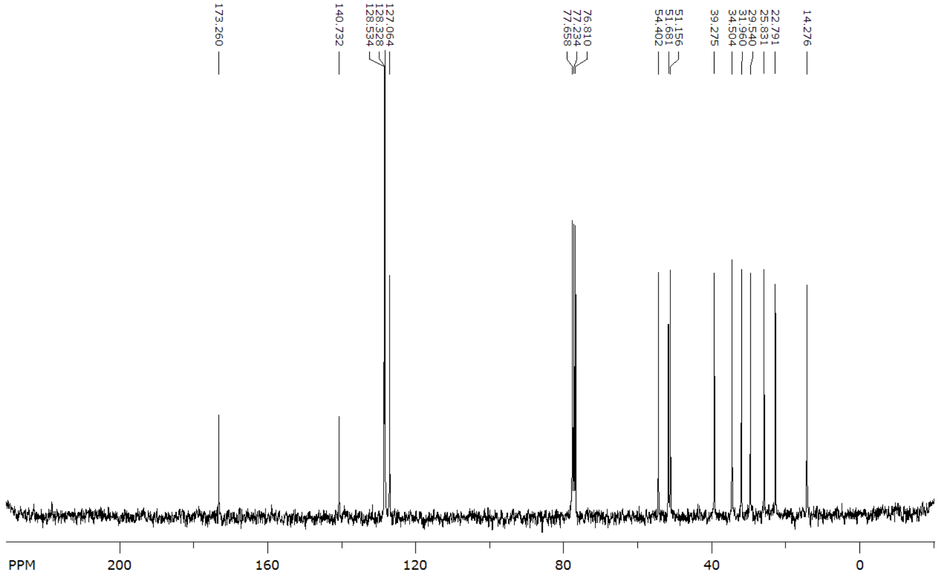

**Figure S25. $^{13}$C spectrum (75 MHz, $CDCl_3$) of *rac-1f***

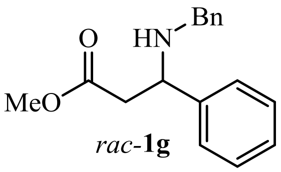


*rac*-**1g**

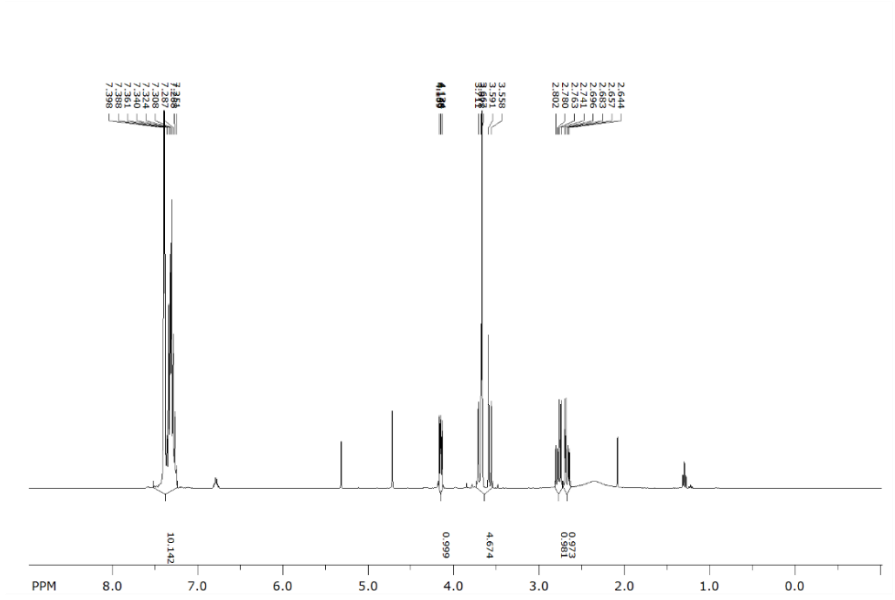


**Figure S26. $^{1}$H spectrum (300 MHz, $CDCl_3$) of *rac-1g.***

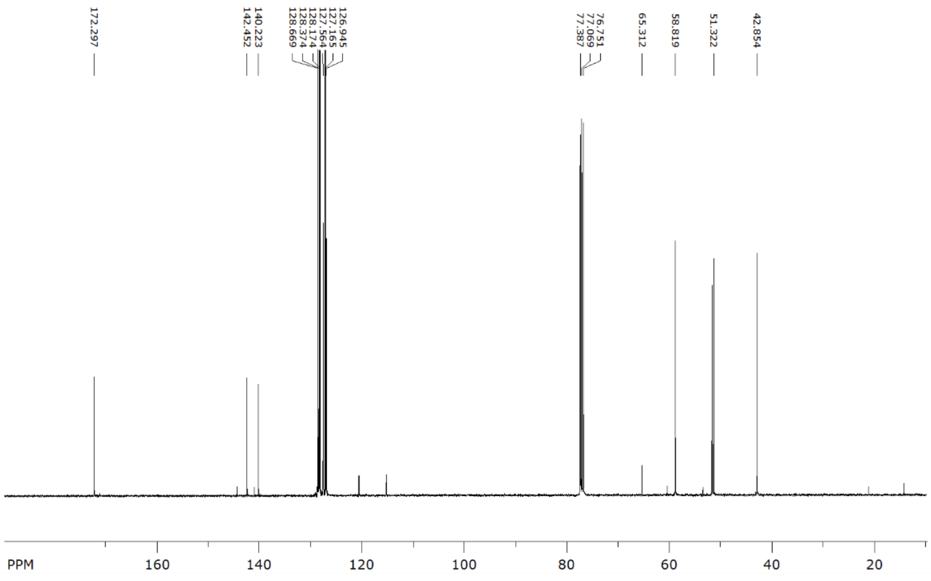


**Figure S27. $^{13}$C spectrum (75 MHz, $CDCl_3$) of *rac-1g.***

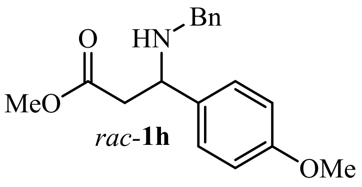


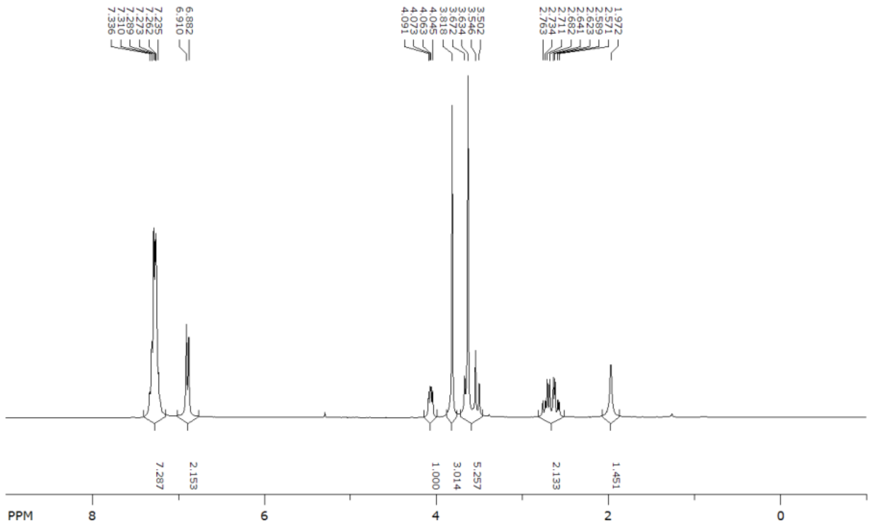


**Figure S28.** $^{1}$**H spectrum (300 MHz, CDCl$_3$) of *rac-1h.***

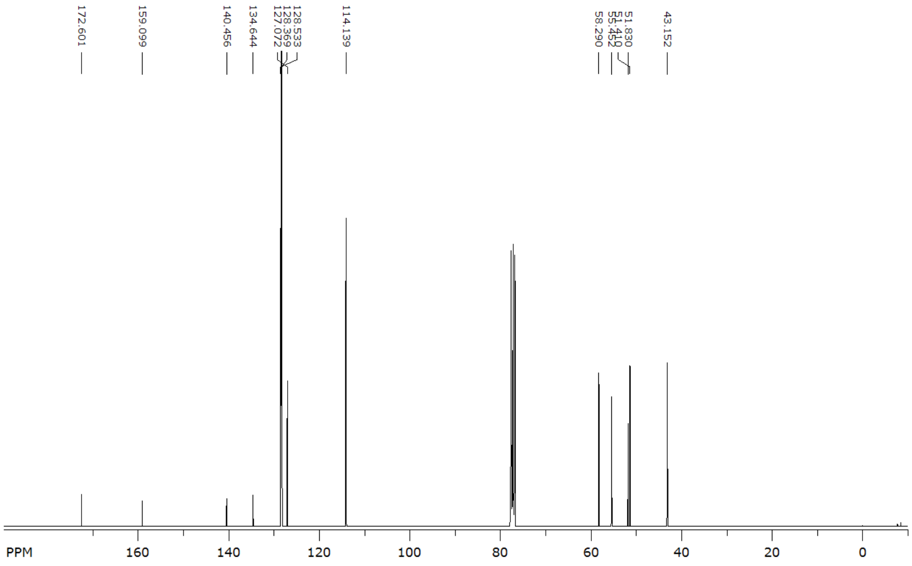


**Figure S29.** $^{13}$**C spectrum (75 MHz, CDCl$_3$) of *rac-1h.***

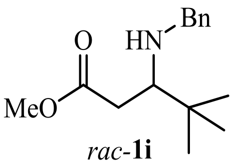


*rac*-**1i**

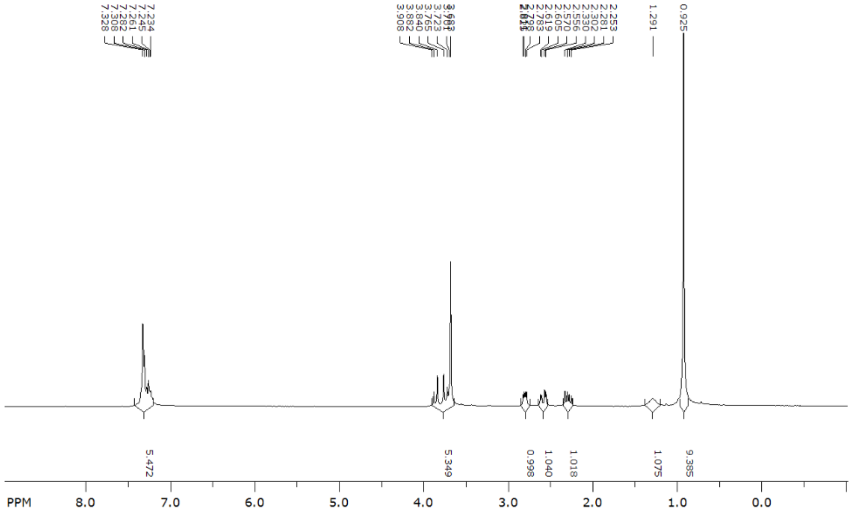


**Figure S30.** $^{1}$**H spectrum (300 MHz, CDCl$_3$) of *rac-1h.***

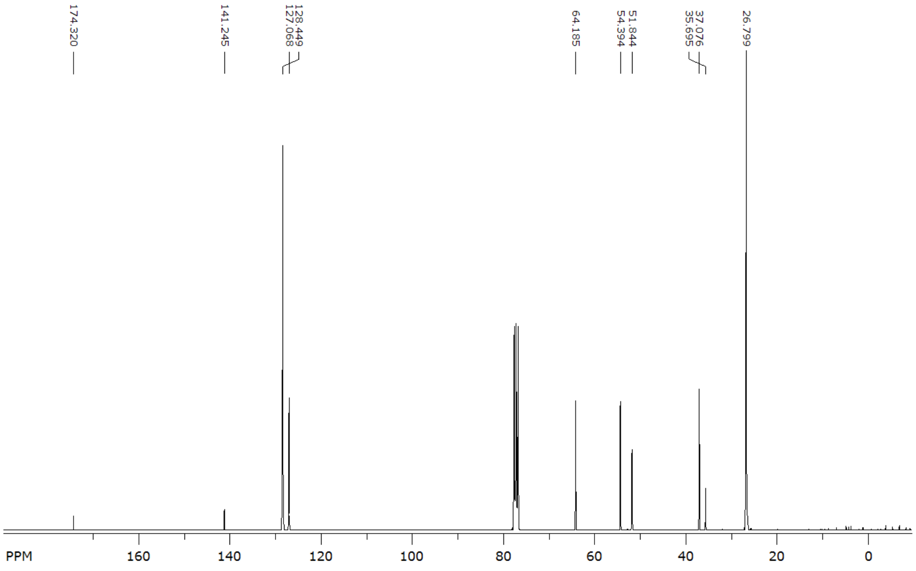


**Figure S31.** $^{13}$**C spectrum (75 MHz, CDCl$_3$) of *rac-1h.***

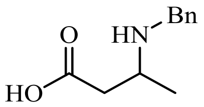

*rac*-**2a**

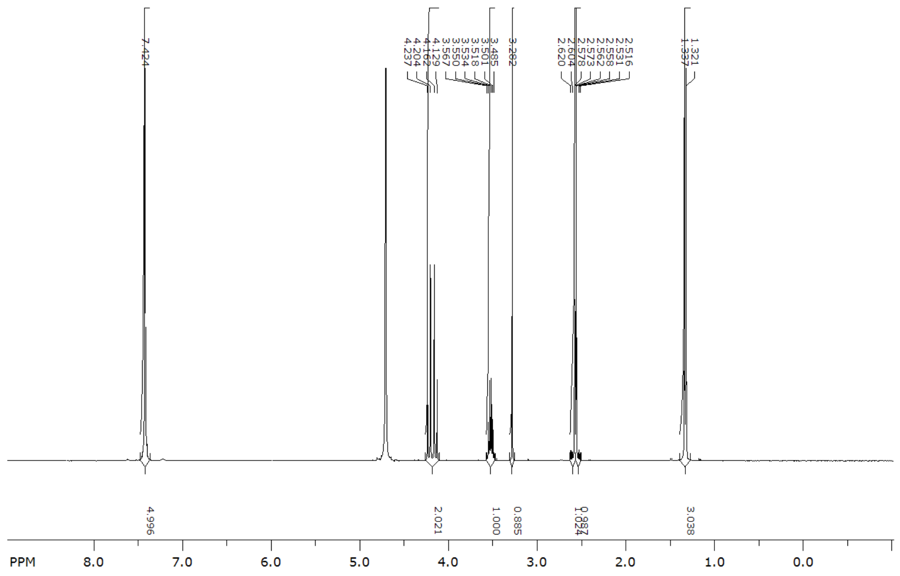

**Figure S32.** $^{1}H$ **spectrum (400 MHz,** $D_2O$**) of** ***2a***

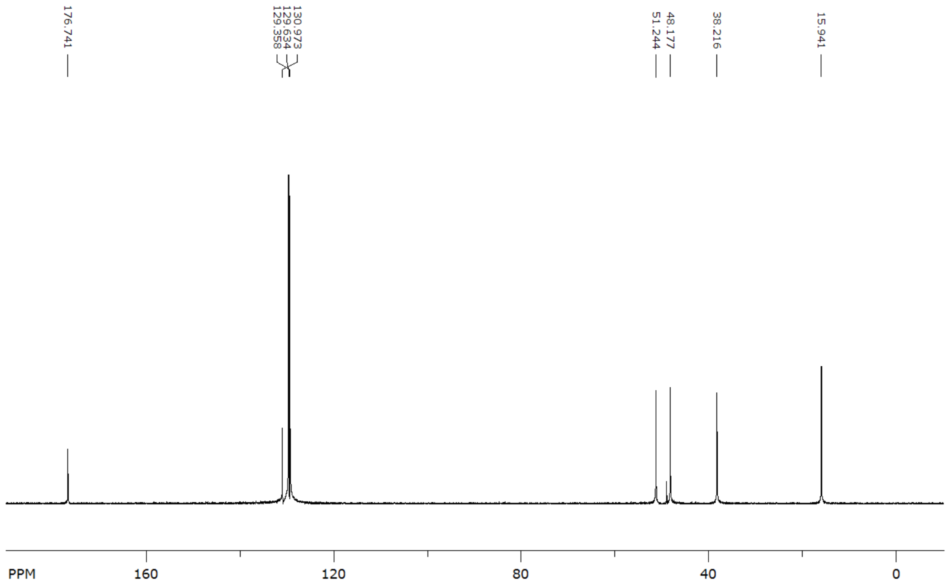

**Figure S33.** $^{13}C$ **spectrum (100 MHz,** $D_2O$**) of** ***2a***

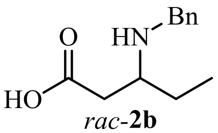


*rac*-**2b**

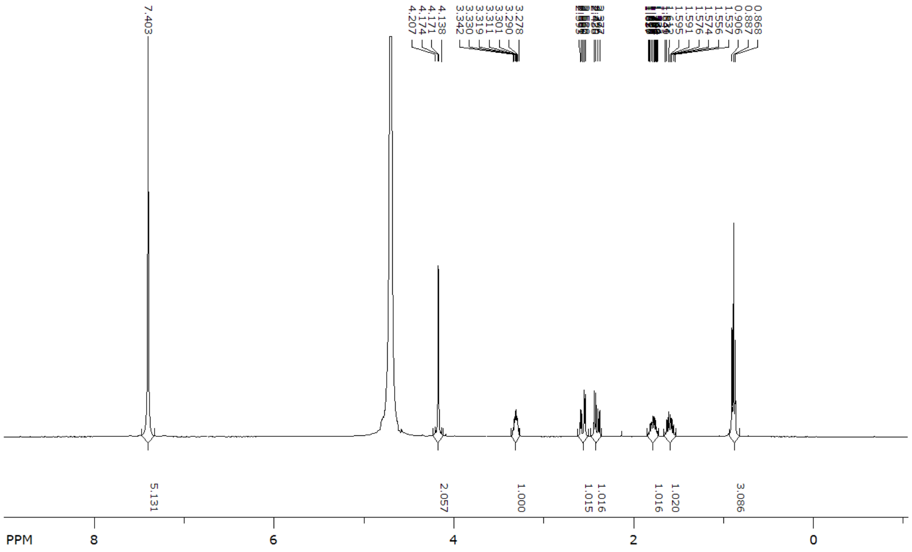


**Figure S34. $^{13}$C spectrum (400 MHz, $D_2O$) of *2b***

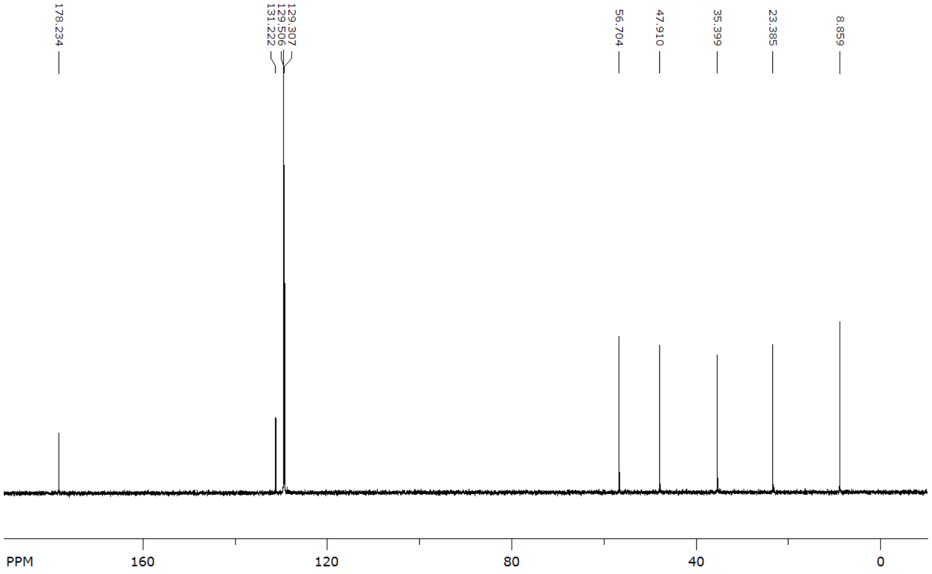


**Figure S35. $^{13}$C spectrum (100 MHz, $D_2O$) of *2b***

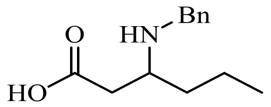


*rac*-**2c**

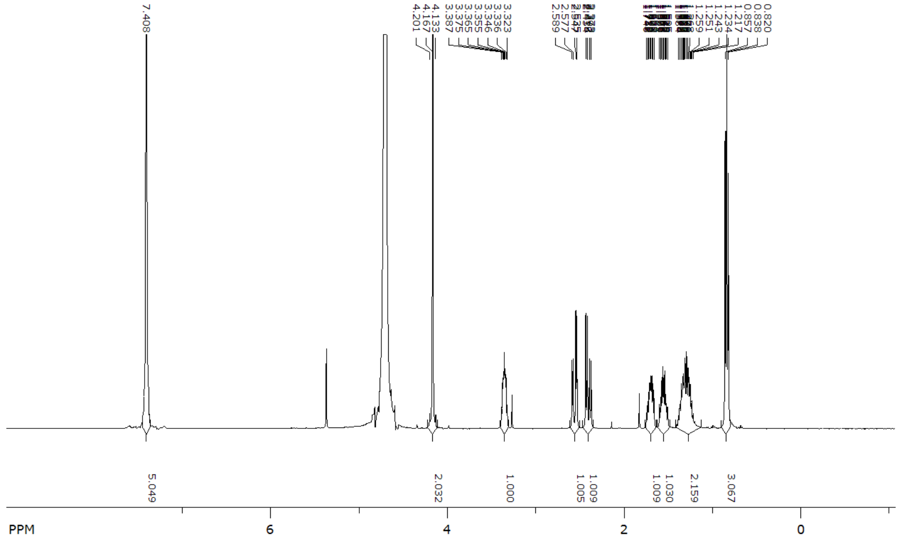


**Figure S36. $^{1}$H spectrum (400 MHz, $D_2O$) of *2c***

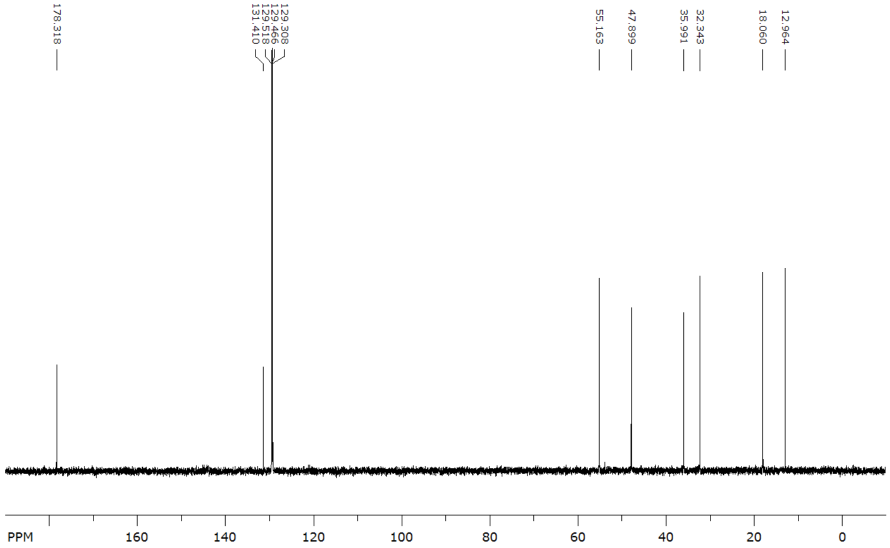


**Figure S37. $^{13}$C spectrum (100 MHz, $D_2O$) of *2c***

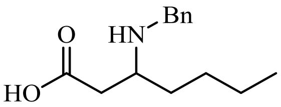


*rac*-**2d**

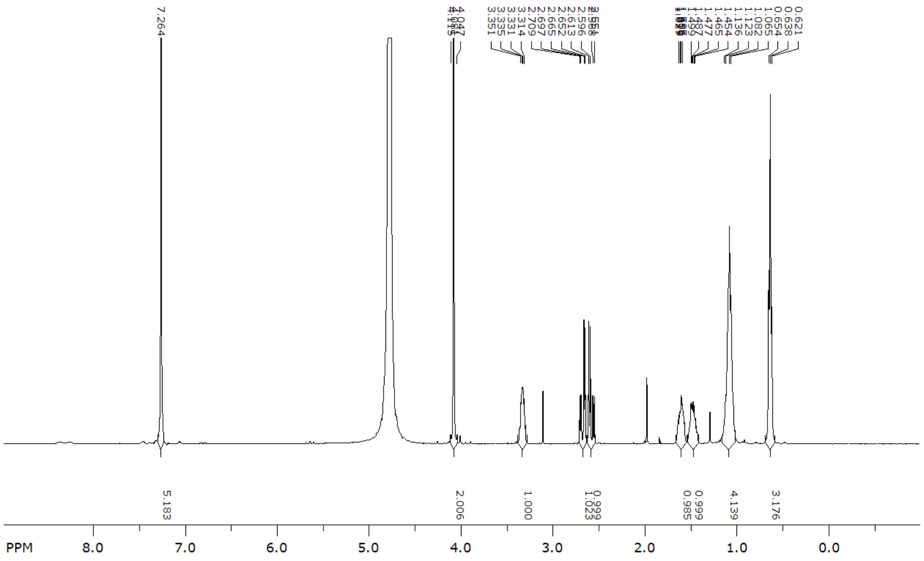


**Figure S38. $^1$H spectrum (400 MHz, $D_2O$) of *2d***

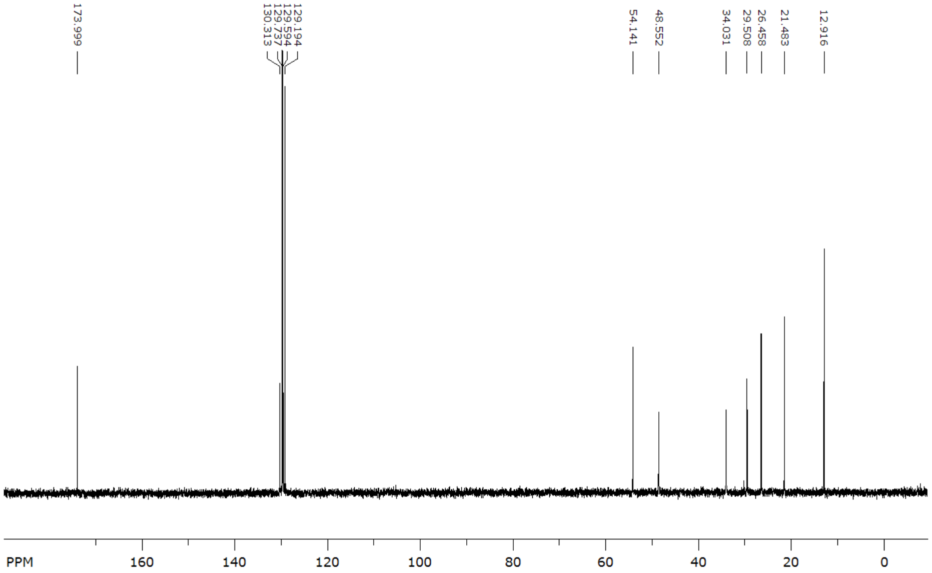


**Figure S39. $^{13}$C spectrum (100 MHz, $D_2O$) of *2d***

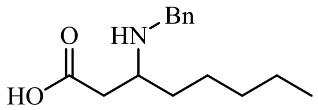


*rac*-**2e**

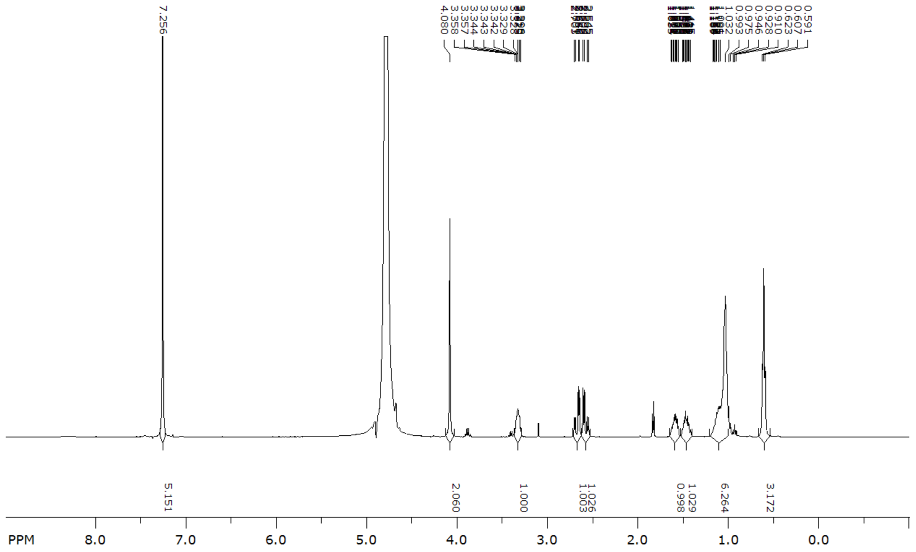


**Figure S40. $^{1}$H spectrum (400 MHz, $D_2O$) of *2e***

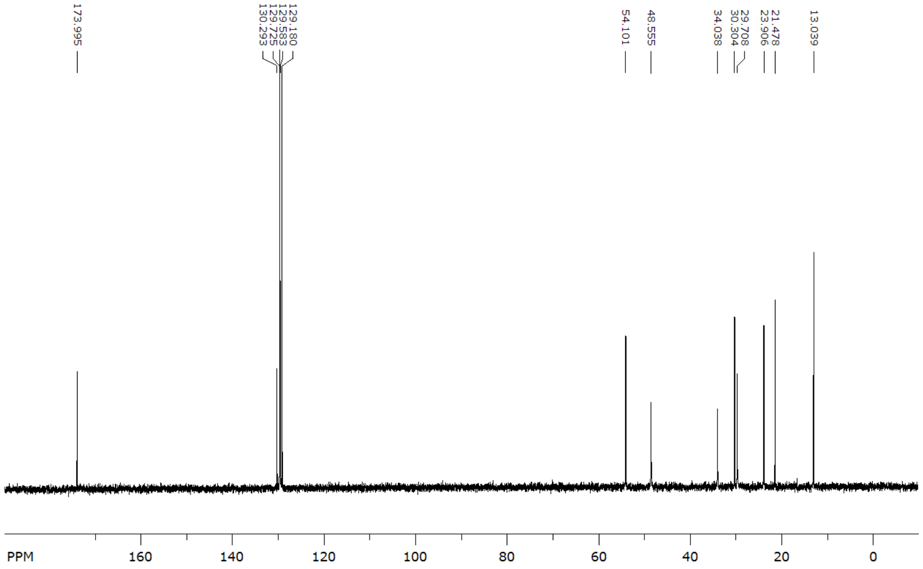


**Figure S41. $^{13}$C spectrum (100 MHz, $D_2O$) of *2e***

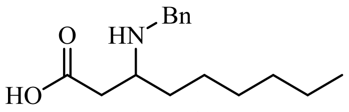

*rac*-**2f**

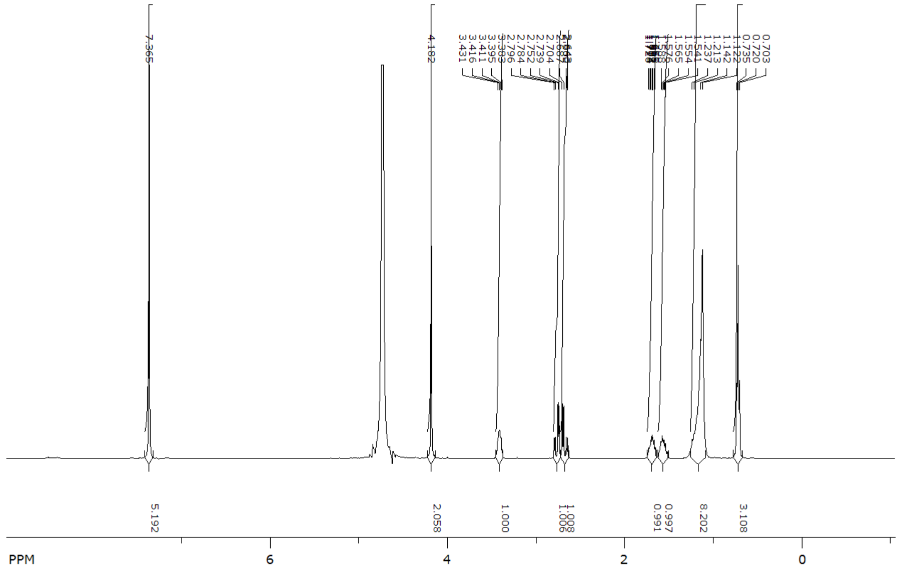

**Figure S42. $^{1}$H spectrum (400 MHz, $D_2O$) of *2f***

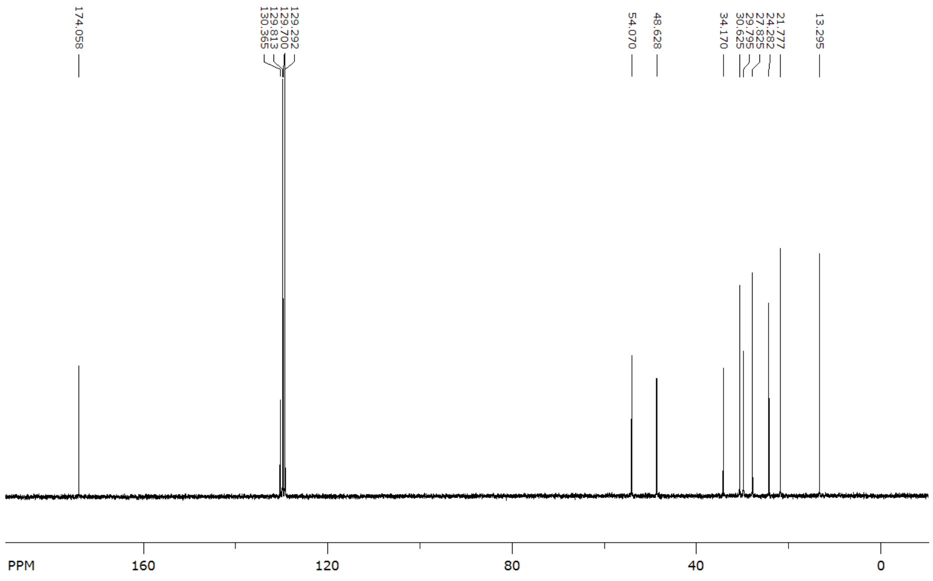

**Figure S43. $^{13}$C spectrum (100 MHz, $D_2O$) of *2f***

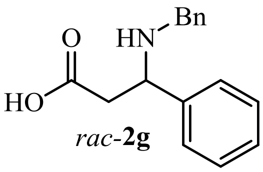


*rac*-**2g**

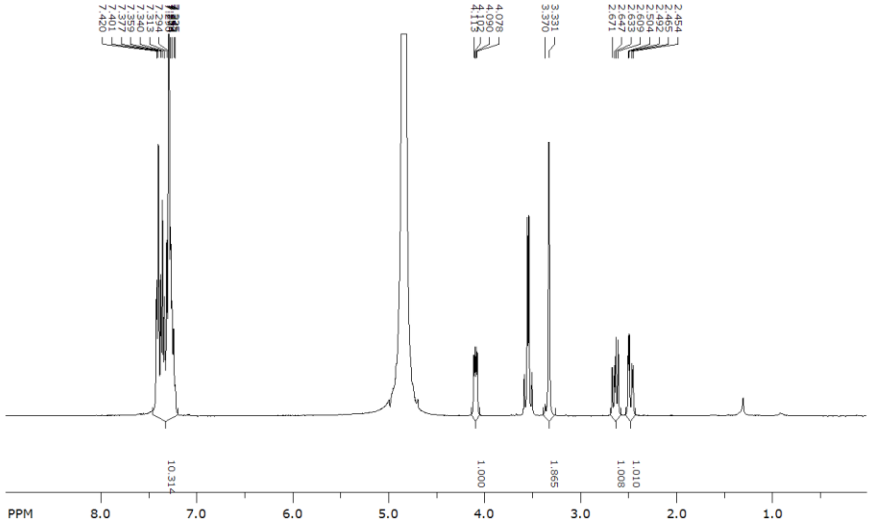

**Figure S44.** $^{1}$**H spectrum (400 MHz,** $CD_3OD$**) of** ***2g***

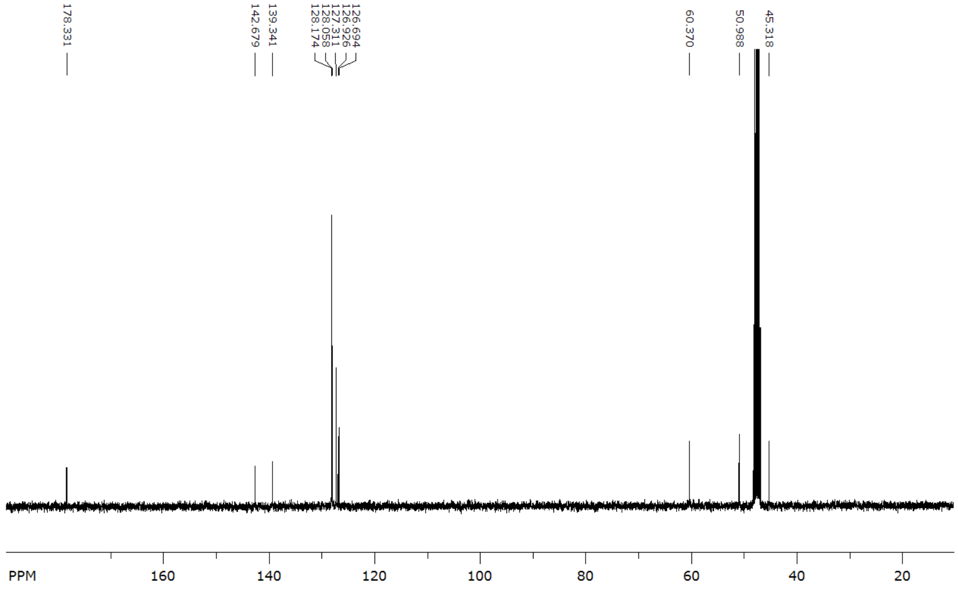

**Figure S45.** $^{13}$**C spectrum (100 MHz,** $CD_3OD$**) of** ***2g***

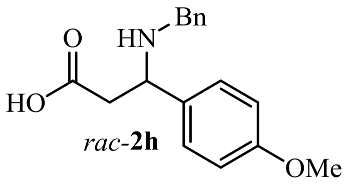


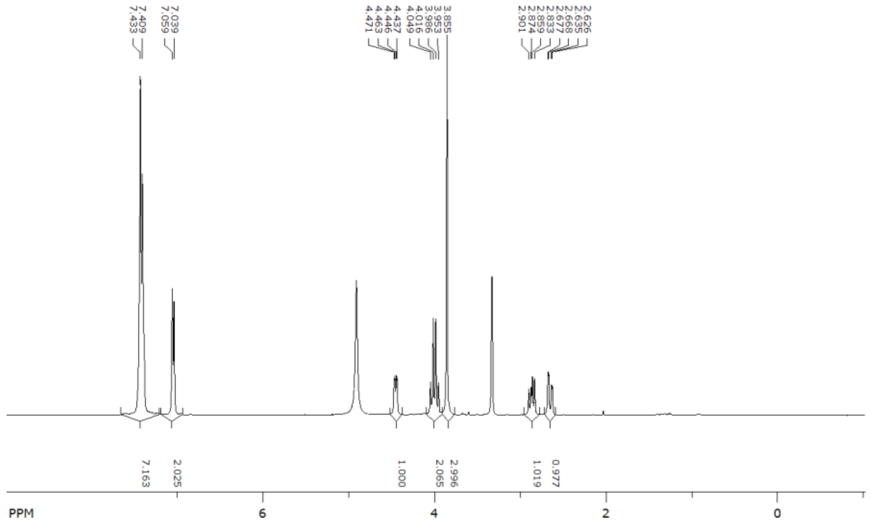


**Figure S46. $^{1}$H spectrum (400 MHz, $CD_3OD$) of *2h***

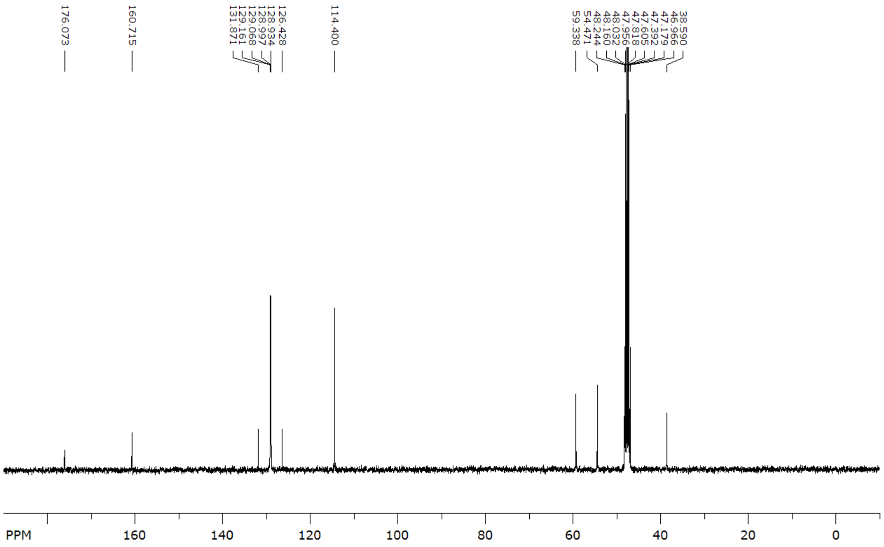


**Figure S47. $^{13}$C spectrum (100 MHz, $CD_3OD$) of *2h***

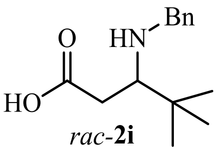


*rac*-**2i**

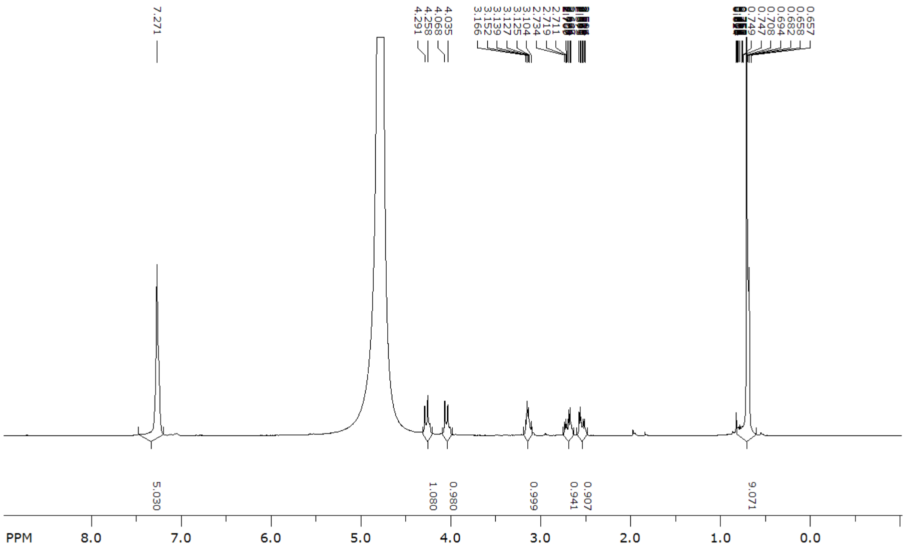


**Figure S48. $^{1}H$ spectrum (400 MHz, $D_2O$) of *2i***

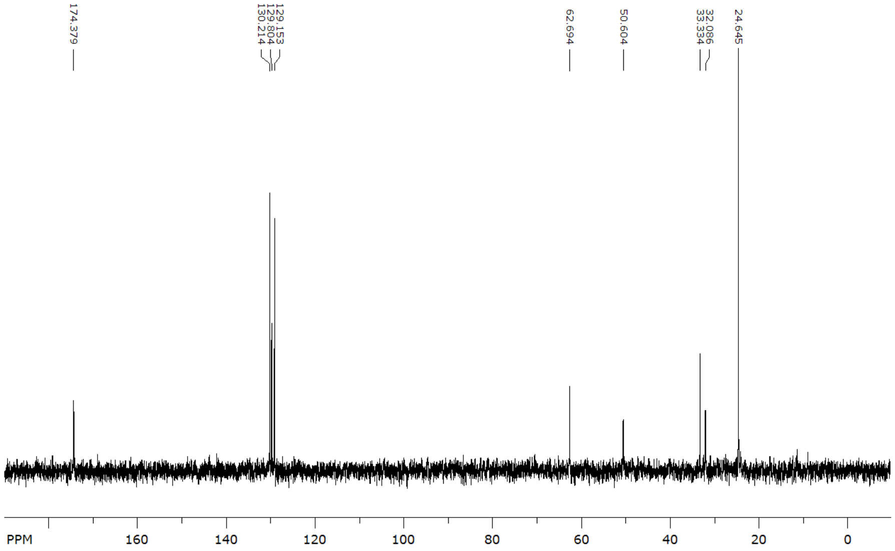


**Figure S49. $^{13}C$ spectrum (100 MHz, $D_2O$) of *2i***

## 6) Chromatograms for the determination of enantiomeric excess of raw material and hydrolysis products.

- **Chromatograms for the search of best conditions for the enzymatic hydrolysis resolution under HSBM (Table S1).**

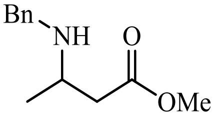


*rac*-**1a**

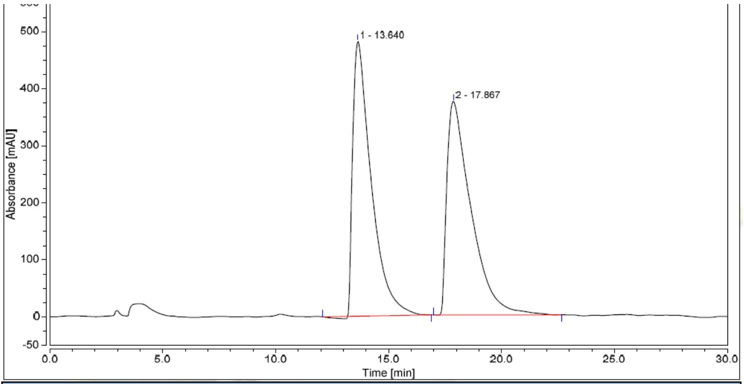


| Integration Results | | | | | | | |
|---|---|---|---|---|---|---|---|
| No. | Peak Name | Retention Time min | Area mAU*min | Height mAU | Relative Area % | Relative Height % | Amount n.a. |
| 1 | | 13.640 | 455.884 | 482.090 | 49.63 | 56.27 | n.a. |
| 2 | | 17.867 | 462.617 | 374.599 | 50.37 | 43.73 | n.a. |
| Total: | | | 918.501 | 856.689 | 100.00 | 100.00 | |

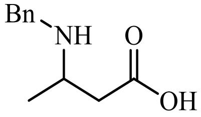


*rac*-**2a**

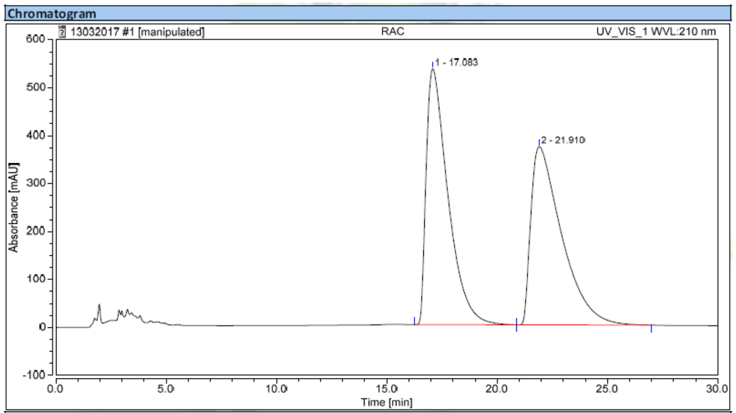


| Integration Results | | | | | | | |
|---|---|---|---|---|---|---|---|
| No. | Peak Name | Retention Time min | Area mAU*min | Height mAU | Relative Area % | Relative Height % | Amount n.a. |
| 1 | | 17.083 | 609.373 | 533.704 | 50.35 | 58.94 | n.a. |
| 2 | | 21.910 | 600.985 | 371.739 | 49.65 | 41.06 | n.a. |

**For entry 1.**

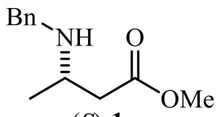

(*S*)-**1a**

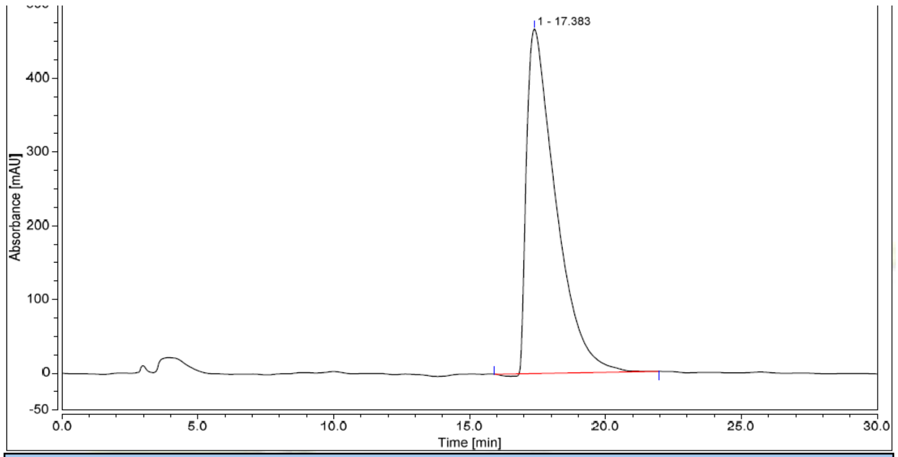


| Integration Results | | | | | | | |
|---|---|---|---|---|---|---|---|
| No. | Peak Name | Retention Time min | Area mAU*min | Height mAU | Relative Area % | Relative Height % | Amount n.a. |
| 1 | | 17.383 | 579.249 | 467.640 | 100.00 | 100.00 | n.a. |
| **Total:** | | | **579.249** | **467.640** | **100.00** | **100.00** | |

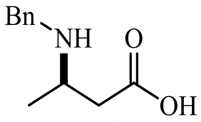

(*R*)-**2a**

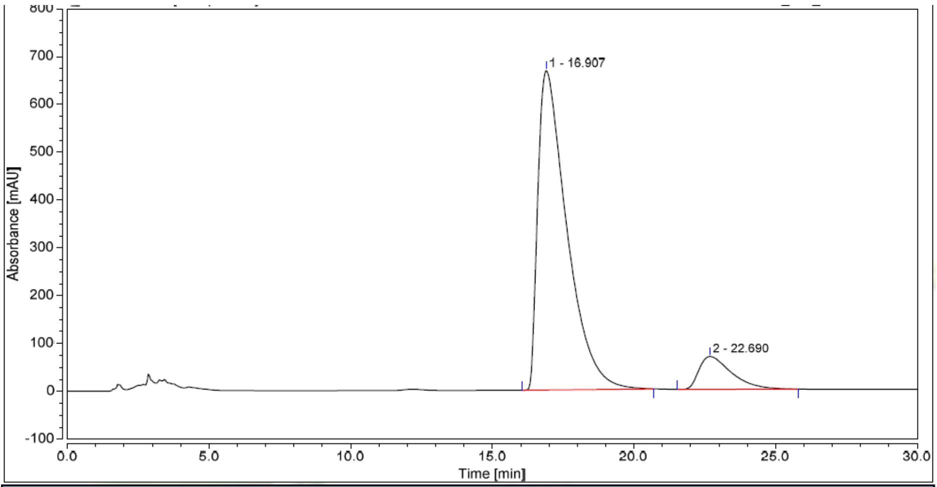


| Integration Results | | | | | | | |
|---|---|---|---|---|---|---|---|
| No. | Peak Name | Retention Time min | Area mAU*min | Height mAU | Relative Area % | Relative Height % | Amount n.a. |
| 1 | | 16.907 | 792.244 | 668.044 | 89.68 | 90.67 | n.a. |
| 2 | | 22.690 | 91.200 | 68.721 | 10.32 | 9.33 | n.a. |
| **Total:** | | | **883.444** | **736.764** | **100.00** | **100.00** | |

**For entry 2.**

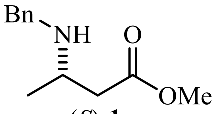


(*S*)-**1a**

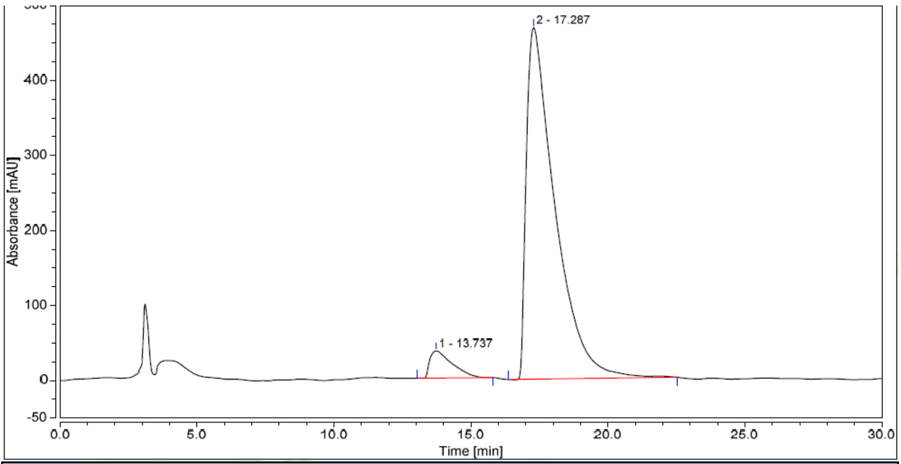


| Integration Results | | | | | | | |
|---|---|---|---|---|---|---|---|
| No. | Peak Name | Retention Time min | Area mAU*min | Height mAU | Relative Area % | Relative Height % | Amount n.a. |
| 1 | | 13.737 | 34.071 | 36.090 | 5.65 | 7.15 | n.a. |
| 2 | | 17.287 | 569.206 | 468.866 | 94.35 | 92.85 | n.a. |
| Total: | | | 603.276 | 504.957 | 100.00 | 100.00 | |

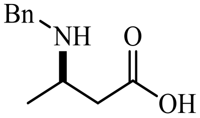


(*R*)-**2a**

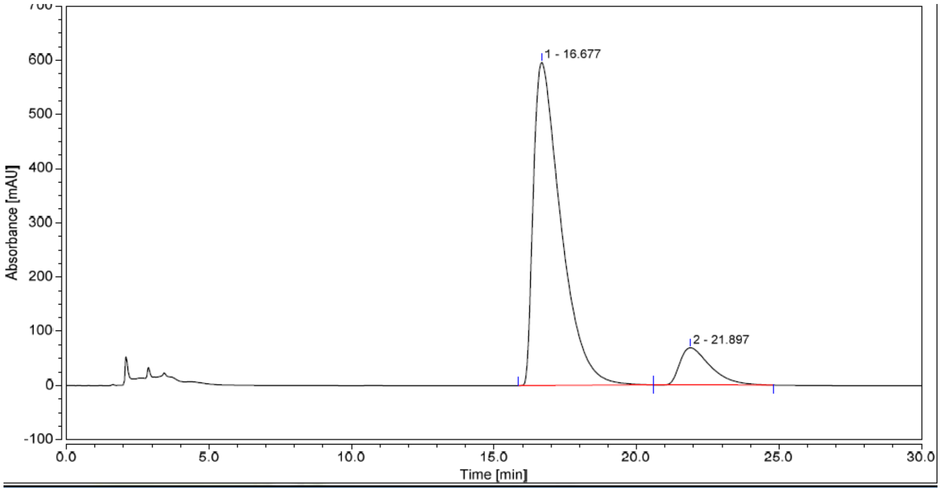


| Integration Results | | | | | | | |
|---|---|---|---|---|---|---|---|
| No. | Peak Name | Retention Time min | Area mAU*min | Height mAU | Relative Area % | Relative Height % | Amount n.a. |
| 1 | | 16.677 | 664.848 | 596.004 | 88.71 | 89.70 | n.a. |
| 2 | | 21.897 | 84.597 | 68.427 | 11.29 | 10.30 | n.a. |
| Total: | | | 749.446 | 664.430 | 100.00 | 100.00 | |

**For entry 3.**

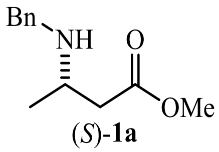


(*S*)-**1a**

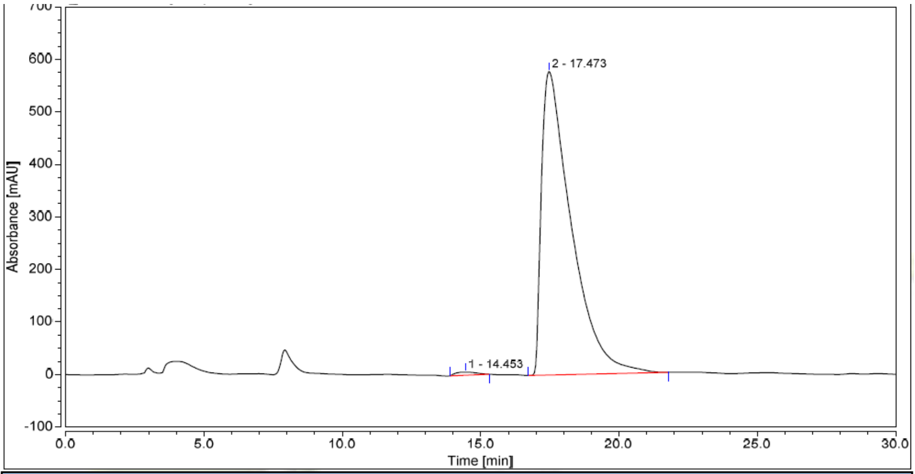


| Integration Results | | | | | | | |
|---|---|---|---|---|---|---|---|
| No. | Peak Name | Retention Time min | Area mAU*min | Height mAU | Relative Area % | Relative Height % | Amount n.a. |
| 1 | | 14.453 | 5.103 | 5.936 | 0.70 | 1.02 | n.a. |
| 2 | | 17.473 | 721.353 | 578.212 | 99.30 | 98.98 | n.a. |
| Total: | | | 726.456 | 584.148 | 100.00 | 100.00 | |

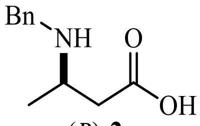


(*R*)-**2a**

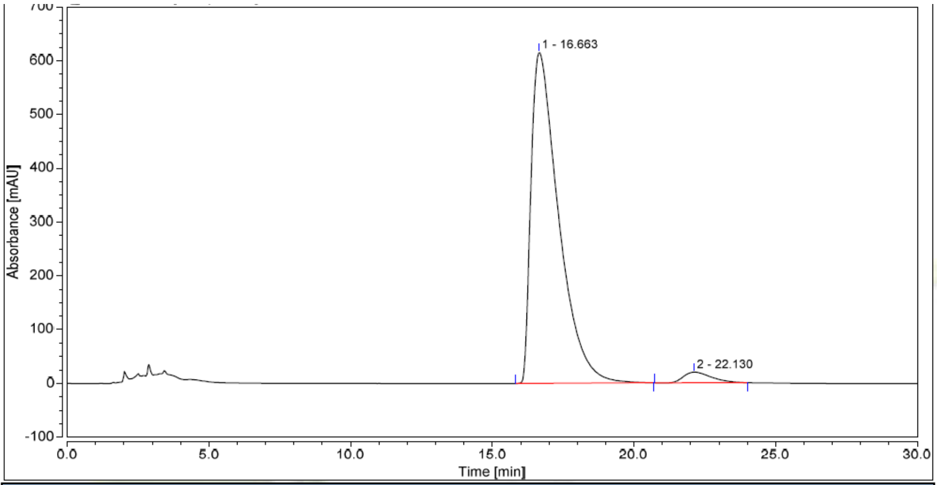


| Integration Results | | | | | | | |
|---|---|---|---|---|---|---|---|
| No. | Peak Name | Retention Time min | Area mAU*min | Height mAU | Relative Area % | Relative Height % | Amount n.a. |
| 1 | | 16.663 | 693.709 | 615.390 | 96.91 | 96.90 | n.a. |
| 2 | | 22.130 | 22.121 | 19.698 | 3.09 | 3.10 | n.a. |
| Total: | | | 715.830 | 635.088 | 100.00 | 100.00 | |

**For entry 4.**

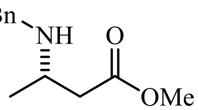

(*S*)-**1a**

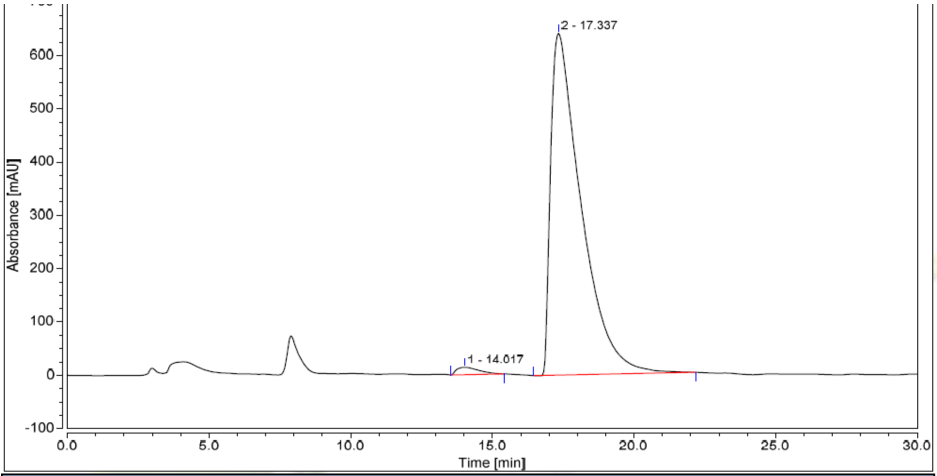


| Integration Results | | | | | | | |
|---|---|---|---|---|---|---|---|
| No. | Peak Name | Retention Time min | Area mAU*min | Height mAU | Relative Area % | Relative Height % | Amount n.a. |
| 1 | | 14.017 | 12.412 | 13.802 | 1.53 | 2.11 | n.a. |
| 2 | | 17.337 | 799.088 | 641.507 | 98.47 | 97.89 | n.a. |
| **Total:** | | | **811.499** | **655.309** | **100.00** | **100.00** | |

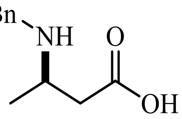

(*R*)-**2a**

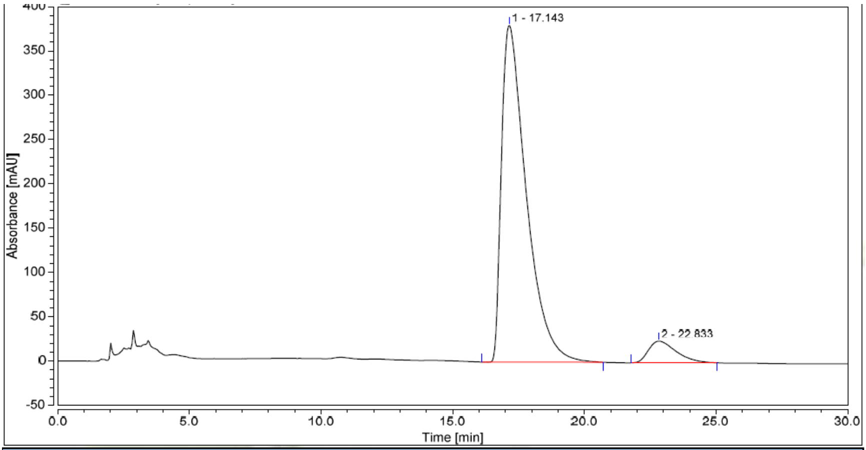


| Integration Results | | | | | | | |
|---|---|---|---|---|---|---|---|
| No. | Peak Name | Retention Time min | Area mAU*min | Height mAU | Relative Area % | Relative Height % | Amount n.a. |
| 1 | | 17.143 | 415.324 | 380.072 | 93.22 | 93.94 | n.a. |
| 2 | | 22.833 | 30.207 | 24.535 | 6.78 | 6.06 | n.a. |
| **Total:** | | | **445.531** | **404.607** | **100.00** | **100.00** | |

**For entry 5.**

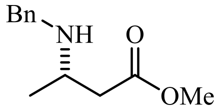


(*S*)-**1a**

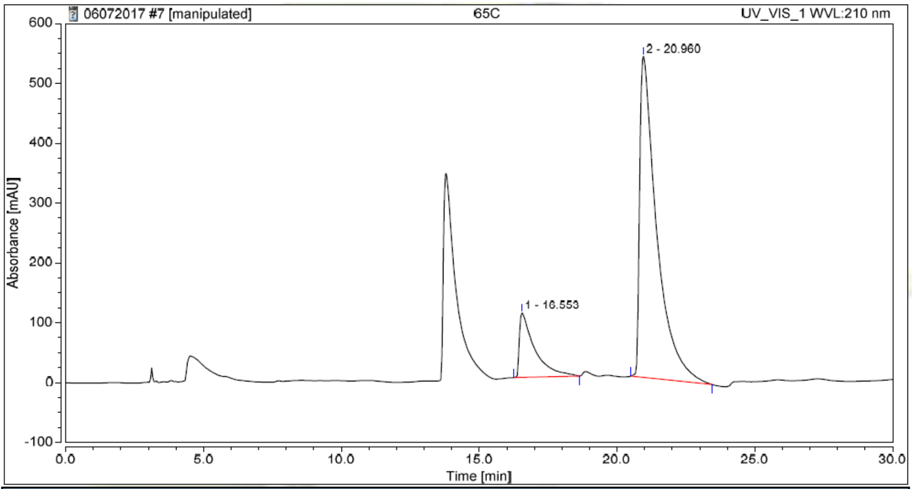


| Integration Results | | | | | | | |
|---|---|---|---|---|---|---|---|
| No. | Peak Name | Retention Time min | Area mAU*min | Height mAU | Relative Area % | Relative Height % | Amount n.a. |
| 1 | | 16.553 | 69.302 | 107.805 | 15.35 | 16.73 | n.a. |
| 2 | | 20.960 | 382.116 | 536.487 | 84.65 | 83.27 | n.a. |
| Total: | | | 451.419 | 644.292 | 100.00 | 100.00 | |

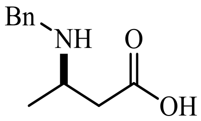


(*R*)-**2a**

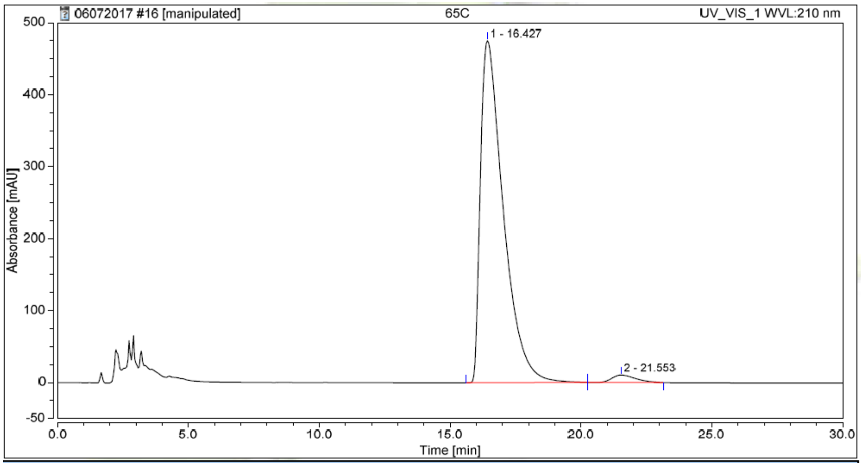


| Integration Results | | | | | | | |
|---|---|---|---|---|---|---|---|
| No. | Peak Name | Retention Time min | Area mAU*min | Height mAU | Relative Area % | Relative Height % | Amount n.a. |
| 1 | | 16.427 | 480.875 | 475.227 | 97.88 | 97.91 | n.a. |
| 2 | | 21.553 | 10.407 | 10.129 | 2.12 | 2.09 | n.a. |
| Total: | | | 491.282 | 485.356 | 100.00 | 100.00 | |

**For entry 6.**

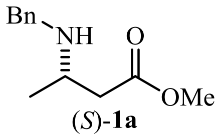


(*S*)-**1a**

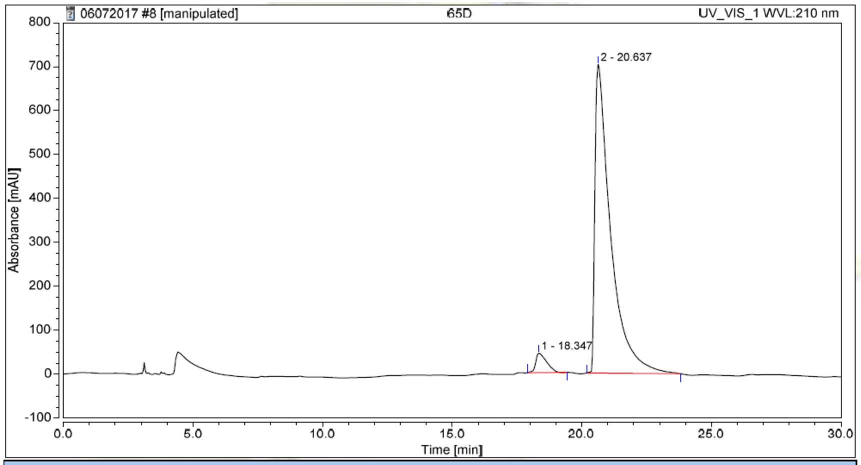


| Integration Results | | | | | | | |
|---|---|---|---|---|---|---|---|
| No. | Peak Name | Retention Time min | Area mAU*min | Height mAU | Relative Area % | Relative Height % | Amount n.a. |
| 1 | | 18.347 | 21.147 | 43.883 | 4.11 | 5.88 | n.a. |
| 2 | | 20.637 | 493.304 | 702.389 | 95.89 | 94.12 | n.a. |
| **Total:** | | | **514.451** | **746.271** | **100.00** | **100.00** | |

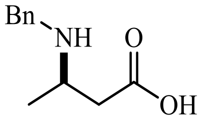


(*R*)-**2a**

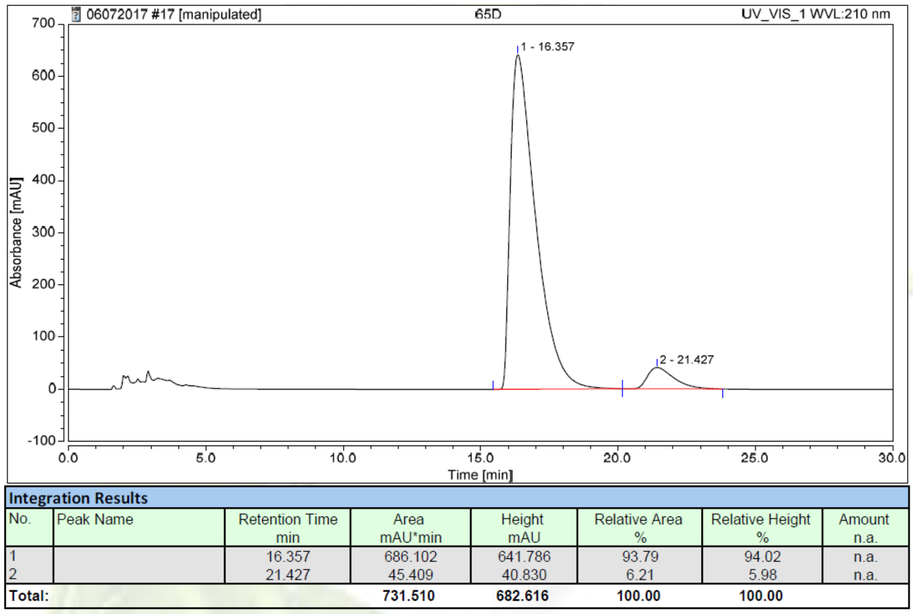


| Integration Results | | | | | | | |
|---|---|---|---|---|---|---|---|
| No. | Peak Name | Retention Time min | Area mAU*min | Height mAU | Relative Area % | Relative Height % | Amount n.a. |
| 1 | | 16.357 | 686.102 | 641.786 | 93.79 | 94.02 | n.a. |
| 2 | | 21.427 | 45.409 | 40.830 | 6.21 | 5.98 | n.a. |
| **Total:** | | | **731.510** | **682.616** | **100.00** | **100.00** | |

**For entry 7.**

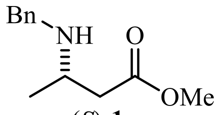

(*S*)-**1a**

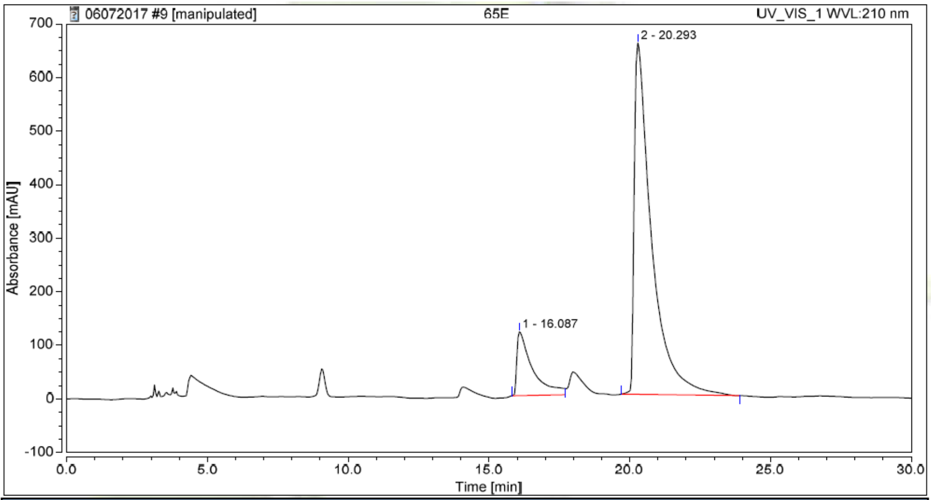


| Integration Results | | | | | | | |
|---|---|---|---|---|---|---|---|
| No. | Peak Name | Retention Time min | Area mAU*min | Height mAU | Relative Area % | Relative Height % | Amount n.a. |
| 1 | | 16.087 | 76.225 | 119.348 | 14.04 | 15.39 | n.a. |
| 2 | | 20.293 | 466.522 | 656.049 | 85.96 | 84.61 | n.a. |
| **Total:** | | | **542.746** | **775.398** | **100.00** | **100.00** | |

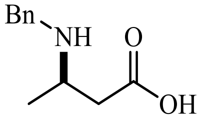

(*R*)-**2a**

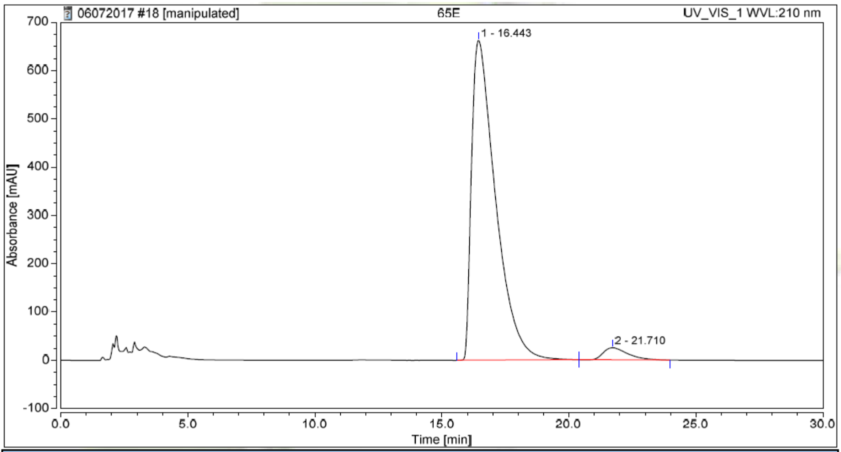


| Integration Results | | | | | | | |
|---|---|---|---|---|---|---|---|
| No. | Peak Name | Retention Time min | Area mAU*min | Height mAU | Relative Area % | Relative Height % | Amount n.a. |
| 1 | | 16.443 | 724.223 | 662.647 | 96.31 | 96.36 | n.a. |
| 2 | | 21.710 | 27.724 | 25.002 | 3.69 | 3.64 | n.a. |
| **Total:** | | | **751.947** | **687.648** | **100.00** | **100.00** | |

**For entry 8.**

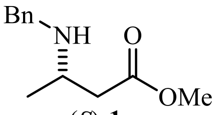

(*S*)-**1a**

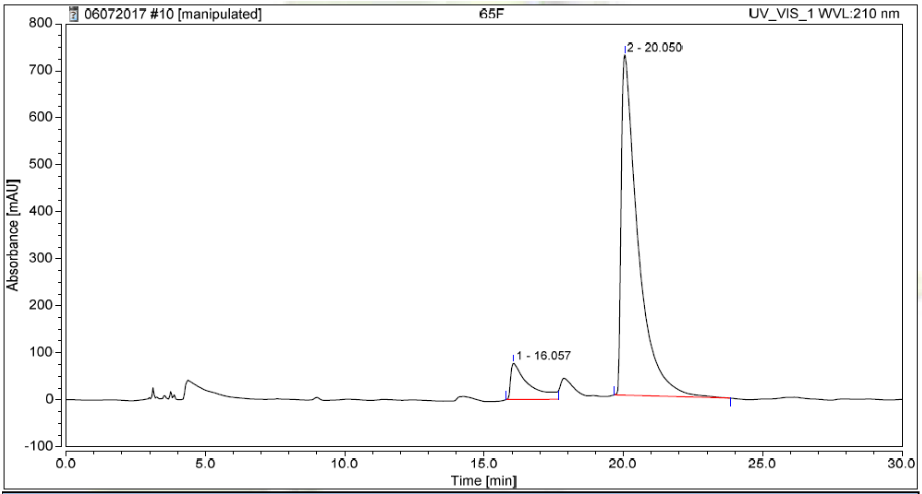


| Integration Results | | | | | | | |
|---|---|---|---|---|---|---|---|
| No. | Peak Name | Retention Time min | Area mAU*min | Height mAU | Relative Area % | Relative Height % | Amount n.a. |
| 1 | | 16.057 | 57.959 | 76.901 | 10.32 | 9.60 | n.a. |
| 2 | | 20.050 | 503.457 | 724.282 | 89.68 | 90.40 | n.a. |
| Total: | | | 561.415 | 801.182 | 100.00 | 100.00 | |

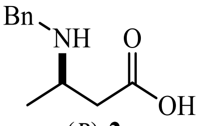

(*R*)-**2a**

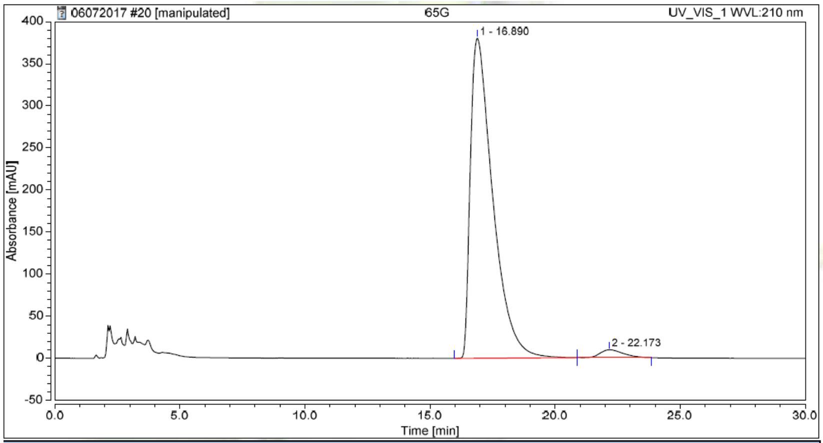


| Integration Results | | | | | | | |
|---|---|---|---|---|---|---|---|
| No. | Peak Name | Retention Time min | Area mAU*min | Height mAU | Relative Area % | Relative Height % | Amount n.a. |
| 1 | | 16.890 | 394.642 | 380.555 | 97.51 | 97.60 | n.a. |
| 2 | | 22.173 | 10.092 | 9.368 | 2.49 | 2.40 | n.a. |
| Total: | | | 404.734 | 389.923 | 100.00 | 100.00 | |

**For entry 9.**

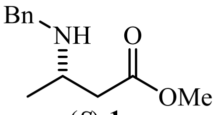

(*S*)-**1a**

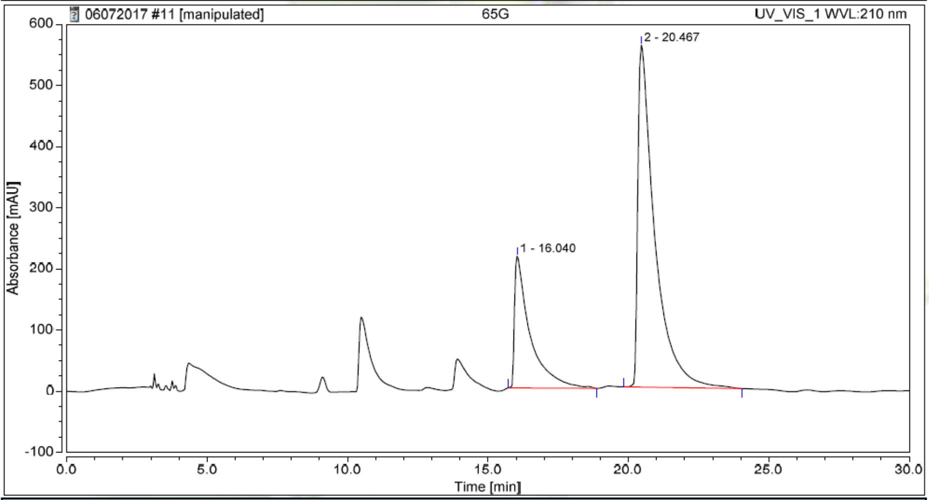


| Integration Results | | | | | | | |
|---|---|---|---|---|---|---|---|
| No. | Peak Name | Retention Time min | Area mAU*min | Height mAU | Relative Area % | Relative Height % | Amount n.a. |
| 1 | | 16.040 | 137.783 | 215.602 | 26.12 | 27.84 | n.a. |
| 2 | | 20.467 | 389.679 | 558.936 | 73.88 | 72.16 | n.a. |
| **Total:** | | | **527.463** | **774.538** | **100.00** | **100.00** | |

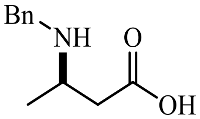

(*R*)-**2a**

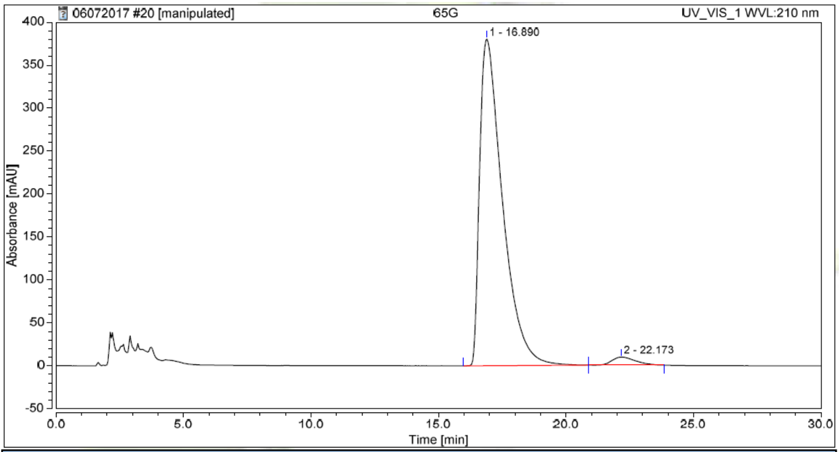


| Integration Results | | | | | | | |
|---|---|---|---|---|---|---|---|
| No. | Peak Name | Retention Time min | Area mAU*min | Height mAU | Relative Area % | Relative Height % | Amount n.a. |
| 1 | | 16.890 | 394.642 | 380.555 | 97.51 | 97.60 | n.a. |
| 2 | | 22.173 | 10.092 | 9.368 | 2.49 | 2.40 | n.a. |
| **Total:** | | | **404.734** | **389.923** | **100.00** | **100.00** | |

**For entry 10.**

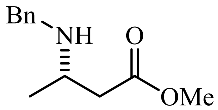

(*S*)-**1a**

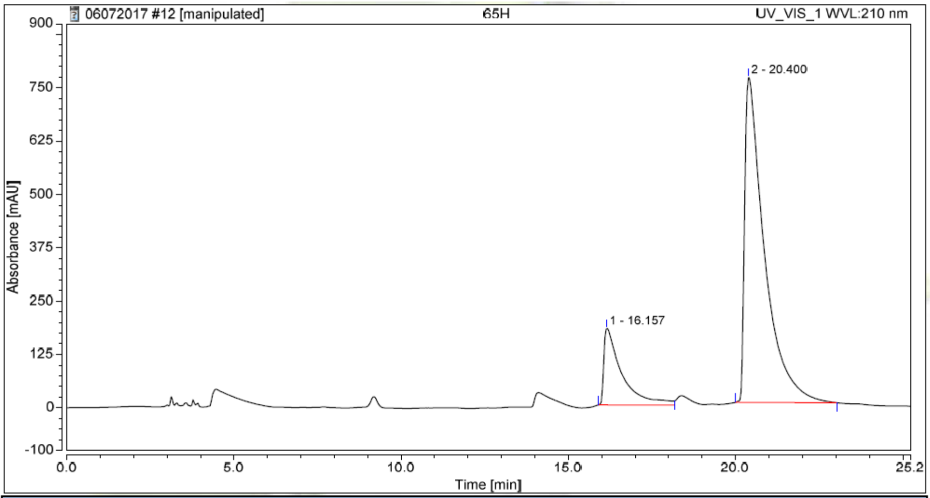


| Integration Results | | | | | | | |
|---|---|---|---|---|---|---|---|
| No. | Peak Name | Retention Time min | Area mAU*min | Height mAU | Relative Area % | Relative Height % | Amount n.a. |
| 1 | | 16.157 | 111.694 | 179.934 | 17.66 | 19.10 | n.a. |
| 2 | | 20.400 | 520.628 | 761.956 | 82.34 | 80.90 | n.a. |
| **Total:** | | | **632.323** | **941.890** | **100.00** | **100.00** | |

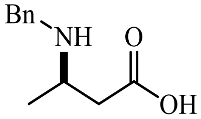

(*R*)-**2a**

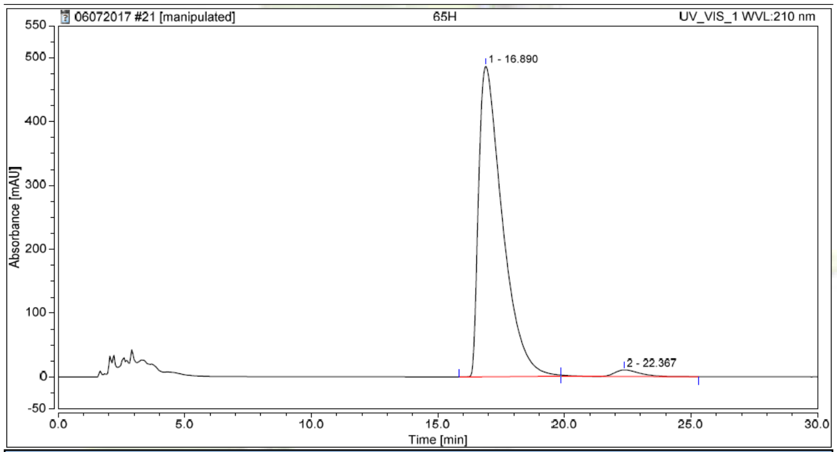


| Integration Results | | | | | | | |
|---|---|---|---|---|---|---|---|
| No. | Peak Name | Retention Time min | Area mAU*min | Height mAU | Relative Area % | Relative Height % | Amount n.a. |
| 1 | | 16.890 | 527.601 | 486.451 | 97.68 | 97.94 | n.a. |
| 2 | | 22.367 | 12.514 | 10.218 | 2.32 | 2.06 | n.a. |
| **Total:** | | | **540.114** | **496.669** | **100.00** | **100.00** | |

**For entry 11.**

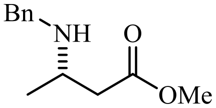
(*S*)-**1a**

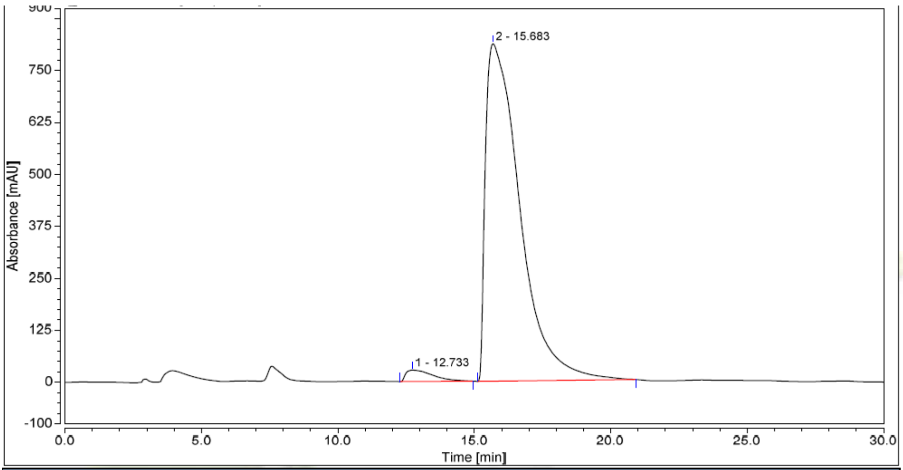


| Integration Results | | | | | | | |
|---|---|---|---|---|---|---|---|
| No. | Peak Name | Retention Time min | Area mAU*min | Height mAU | Relative Area % | Relative Height % | Amount n.a. |
| 1 | | 12.733 | 31.315 | 27.053 | 2.57 | 3.22 | n.a. |
| 2 | | 15.683 | 1185.288 | 811.859 | 97.43 | 96.78 | n.a. |
| Total: | | | 1216.603 | 838.912 | 100.00 | 100.00 | |

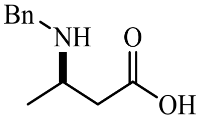
(*R*)-**2a**

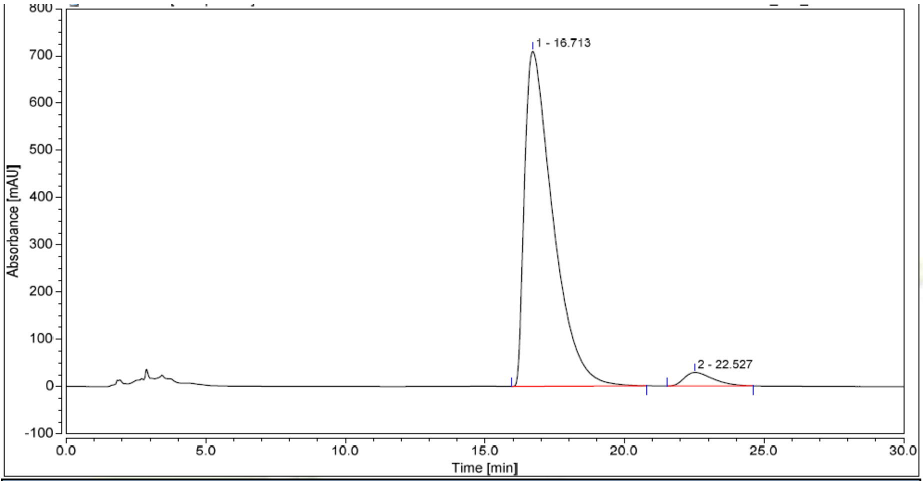


| Integration Results | | | | | | | |
|---|---|---|---|---|---|---|---|
| No. | Peak Name | Retention Time min | Area mAU*min | Height mAU | Relative Area % | Relative Height % | Amount n.a. |
| 1 | | 16.713 | 834.034 | 710.080 | 96.02 | 96.11 | n.a. |
| 2 | | 22.527 | 34.529 | 28.721 | 3.98 | 3.89 | n.a. |
| Total: | | | 868.563 | 738.800 | 100.00 | 100.00 | |

**For entry 12.**

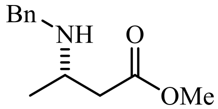

(*S*)-**1a**

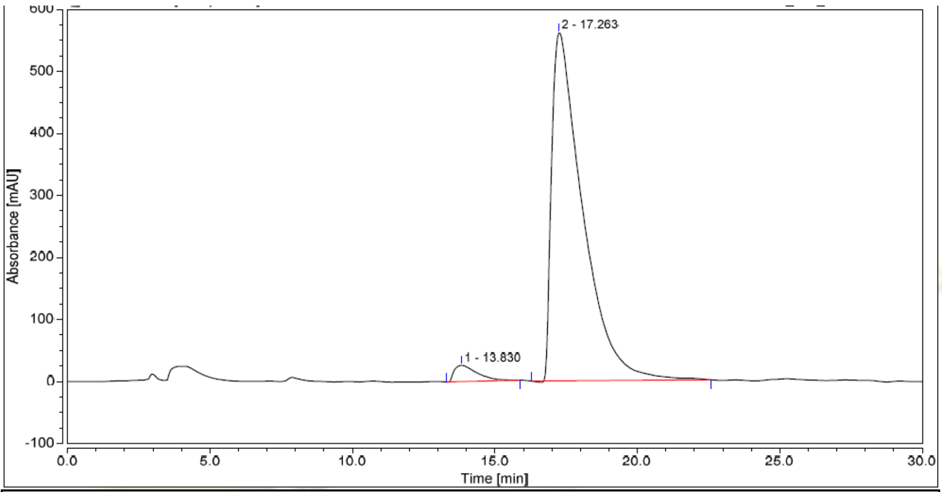


| Integration Results | | | | | | | |
|---|---|---|---|---|---|---|---|
| No. | Peak Name | Retention Time min | Area mAU*min | Height mAU | Relative Area % | Relative Height % | Amount n.a. |
| 1 | | 13.830 | 24.251 | 26.194 | 3.31 | 4.46 | n.a. |
| 2 | | 17.263 | 708.473 | 561.512 | 96.69 | 95.54 | n.a. |
| Total: | | | 732.724 | 587.706 | 100.00 | 100.00 | |

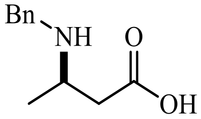

(*R*)-**2a**

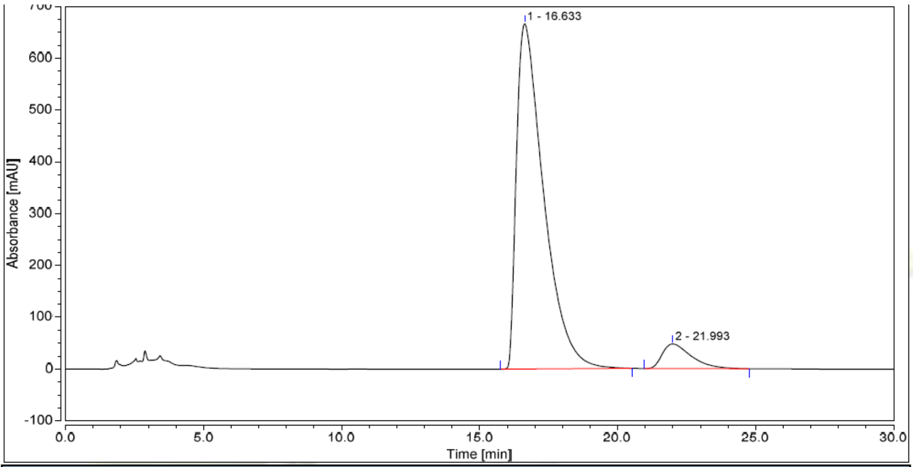


| Integration Results | | | | | | | |
|---|---|---|---|---|---|---|---|
| No. | Peak Name | Retention Time min | Area mAU*min | Height mAU | Relative Area % | Relative Height % | Amount n.a. |
| 1 | | 16.633 | 759.988 | 666.344 | 92.92 | 93.34 | n.a. |
| 2 | | 21.993 | 57.872 | 47.564 | 7.08 | 6.66 | n.a. |
| Total: | | | 817.860 | 713.909 | 100.00 | 100.00 | |

**For entry 13.**

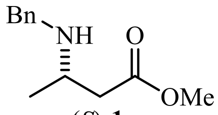


(*S*)-**1a**

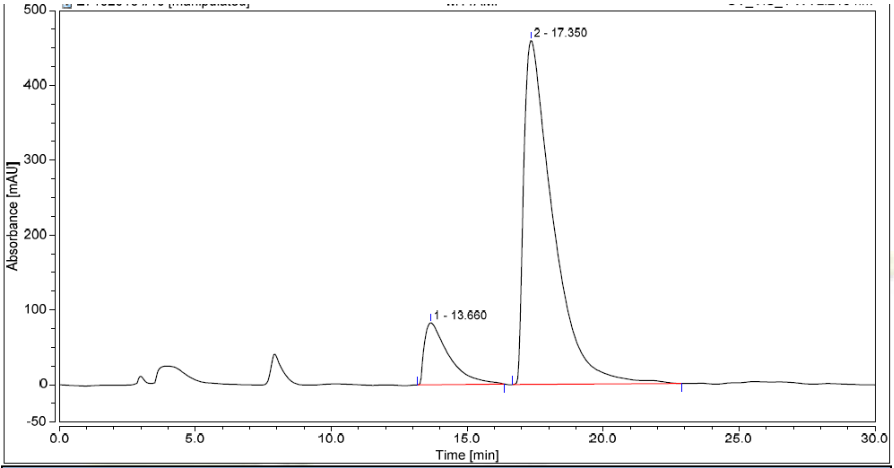


| Integration Results | | | | | | | |
|---|---|---|---|---|---|---|---|
| No. | Peak Name | Retention Time min | Area mAU*min | Height mAU | Relative Area % | Relative Height % | Amount n.a. |
| 1 | | 13.660 | 85.810 | 82.777 | 13.05 | 15.25 | n.a. |
| 2 | | 17.350 | 571.918 | 459.992 | 86.95 | 84.75 | n.a. |
| **Total:** | | | **657.728** | **542.768** | **100.00** | **100.00** | |

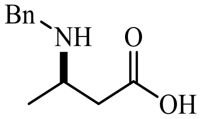


(*R*)-**2a**

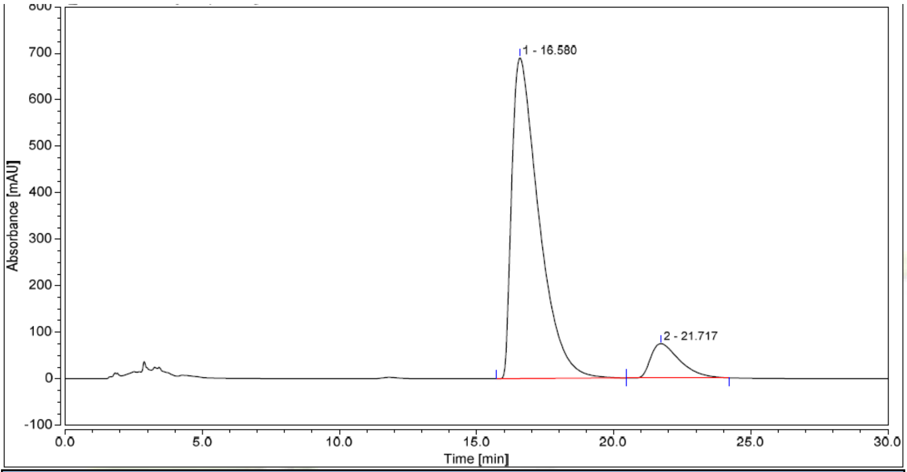


| Integration Results | | | | | | | |
|---|---|---|---|---|---|---|---|
| No. | Peak Name | Retention Time min | Area mAU*min | Height mAU | Relative Area % | Relative Height % | Amount n.a. |
| 1 | | 16.580 | 787.488 | 690.073 | 89.77 | 90.35 | n.a. |
| 2 | | 21.717 | 89.760 | 73.686 | 10.23 | 9.65 | n.a. |
| **Total:** | | | **877.247** | **763.759** | **100.00** | **100.00** | |

- **Chromatograms for substrate scope for the enzymatic resolution of N-benzylated-β$^3$-amino acid methyl esters (Table S2)**

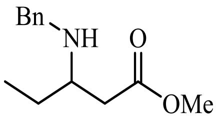


*rac*-**1b**

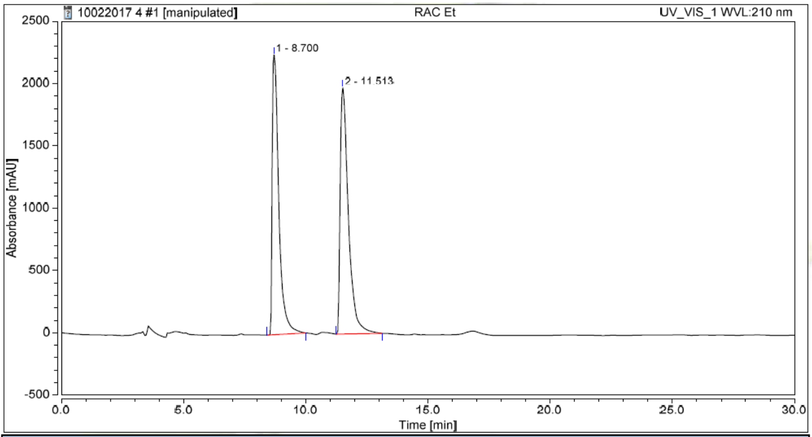


| Integration Results | | | | | | | |
|---|---|---|---|---|---|---|---|
| No. | Peak Name | Retention Time min | Area mAU*min | Height mAU | Relative Area % | Relative Height % | Amount n.a. |
| 1 | | 8.700 | 702.758 | 2245.976 | 47.86 | 53.19 | n.a. |
| 2 | | 11.513 | 765.462 | 1976.201 | 52.14 | 46.81 | n.a. |
| Total: | | | 1468.220 | 4222.176 | 100.00 | 100.00 | |

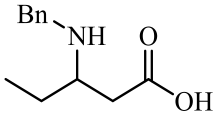


*rac*-**2b**

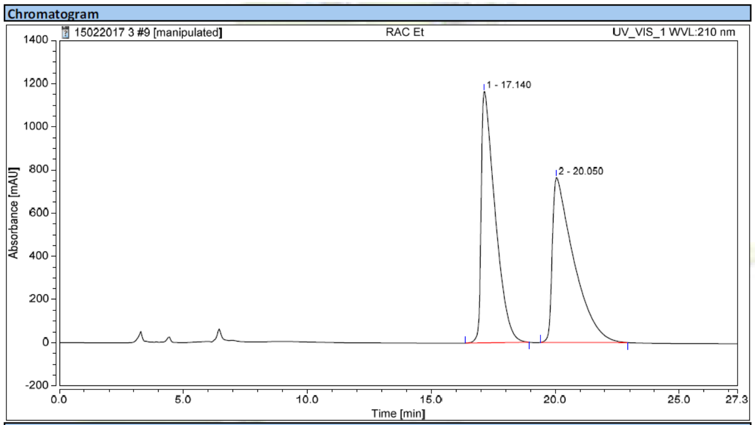


| Integration Results | | | | | | | |
|---|---|---|---|---|---|---|---|
| No. | Peak Name | Retention Time min | Area mAU*min | Height mAU | Relative Area % | Relative Height % | Amount n.a. |
| 1 | | 17.140 | 760.046 | 1165.262 | 49.83 | 60.36 | n.a. |
| 2 | | 20.050 | 765.325 | 765.205 | 50.17 | 39.64 | n.a. |
| Total: | | | 1525.371 | 1930.467 | 100.00 | 100.00 | |

**For entry 1.**

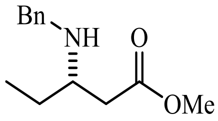


(*S*)-**1b**

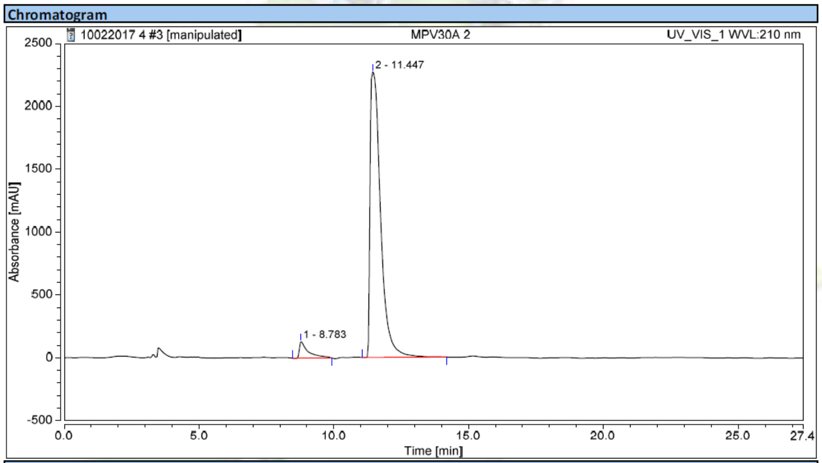


Integration Results

| No. | Peak Name | Retention Time min | Area mAU*min | Height mAU | Relative Area % | Relative Height % | Amount n.a. |
|---|---|---|---|---|---|---|---|
| 1 | | 8.783 | 49.842 | 133.253 | 4.63 | 5.54 | n.a. |
| 2 | | 11.447 | 1026.376 | 2273.509 | 95.37 | 94.46 | n.a. |
| Total: | | | 1076.218 | 2406.762 | 100.00 | 100.00 | |

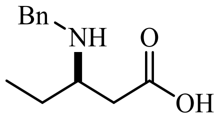


(*R*)-**2b**

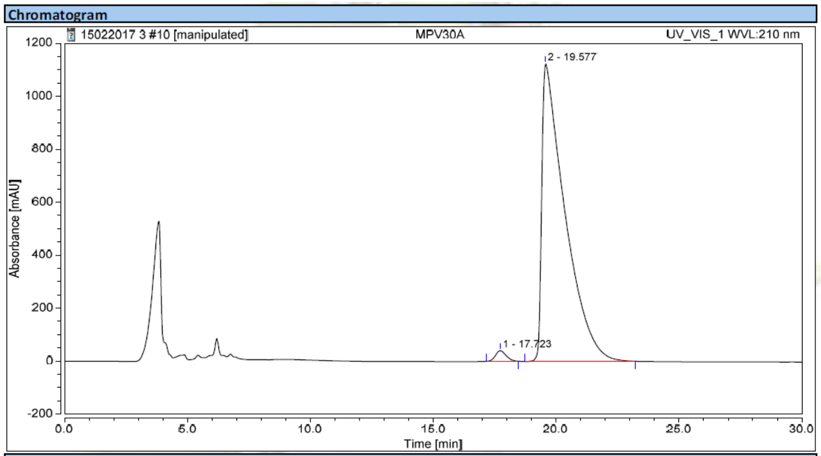


Integration Results

| No. | Peak Name | Retention Time min | Area mAU*min | Height mAU | Relative Area % | Relative Height % | Amount n.a. |
|---|---|---|---|---|---|---|---|
| 1 | | 17.723 | 20.051 | 40.113 | 1.61 | 3.45 | n.a. |
| 2 | | 19.577 | 1222.183 | 1124.148 | 98.39 | 96.55 | n.a. |
| Total: | | | 1242.234 | 1164.262 | 100.00 | 100.00 | |

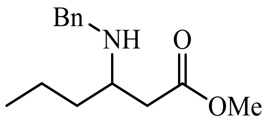

*rac*-**1c**

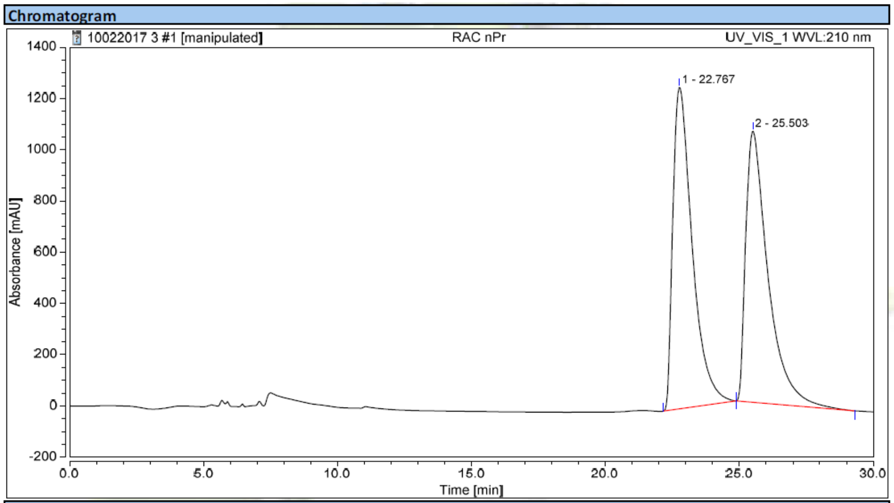


**Integration Results**

| No. | Peak Name | Retention Time min | Area mAU*min | Height mAU | Relative Area % | Relative Height % | Amount n.a. |
|---|---|---|---|---|---|---|---|
| 1 | | 22.767 | 1052.710 | 1257.157 | 50.81 | 54.25 | n.a. |
| 2 | | 25.503 | 1019.319 | 1060.358 | 49.19 | 45.75 | n.a. |
| Total: | | | **2072.028** | **2317.514** | **100.00** | **100.00** | |

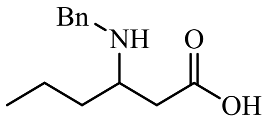

*rac*-**2c**

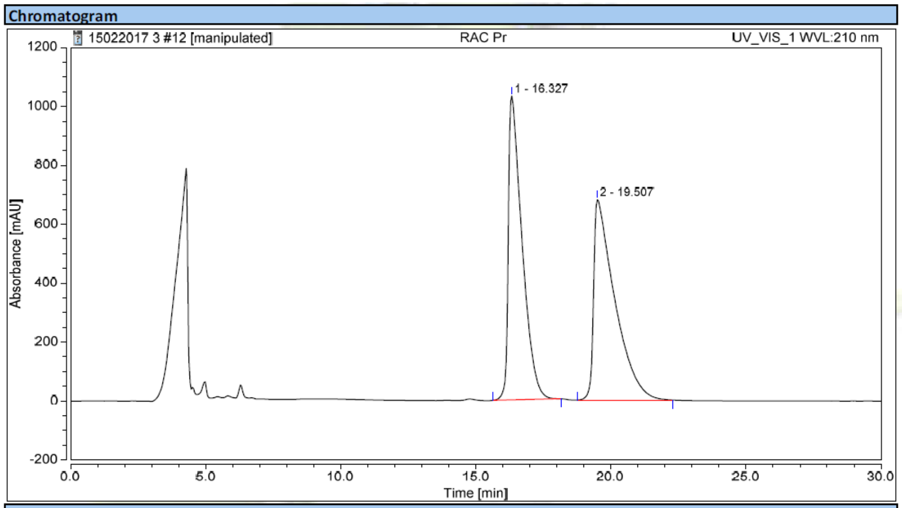


**Integration Results**

| No. | Peak Name | Retention Time min | Area mAU*min | Height mAU | Relative Area % | Relative Height % | Amount n.a. |
|---|---|---|---|---|---|---|---|
| 1 | | 16.327 | 608.778 | 1031.470 | 49.69 | 60.24 | n.a. |
| 2 | | 19.507 | 616.275 | 680.864 | 50.31 | 39.76 | n.a. |
| Total: | | | **1225.053** | **1712.334** | **100.00** | **100.00** | |

**For entry 2.**

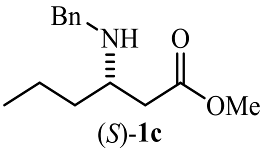


(*S*)-**1c**

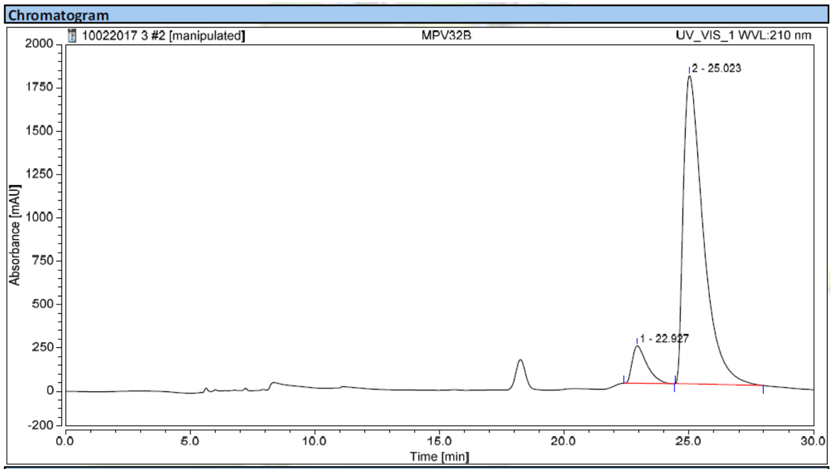


Integration Results

| No. | Peak Name | Retention Time min | Area mAU*min | Height mAU | Relative Area % | Relative Height % | Amount n.a. |
|---|---|---|---|---|---|---|---|
| 1 | | 22.927 | 147.188 | 217.331 | 8.17 | 10.88 | n.a. |
| 2 | | 25.023 | 1654.145 | 1780.392 | 91.83 | 89.12 | n.a. |
| **Total:** | | | **1801.333** | **1997.723** | **100.00** | **100.00** | |

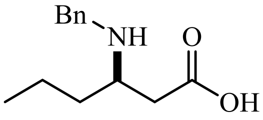


(*R*)-**2c**

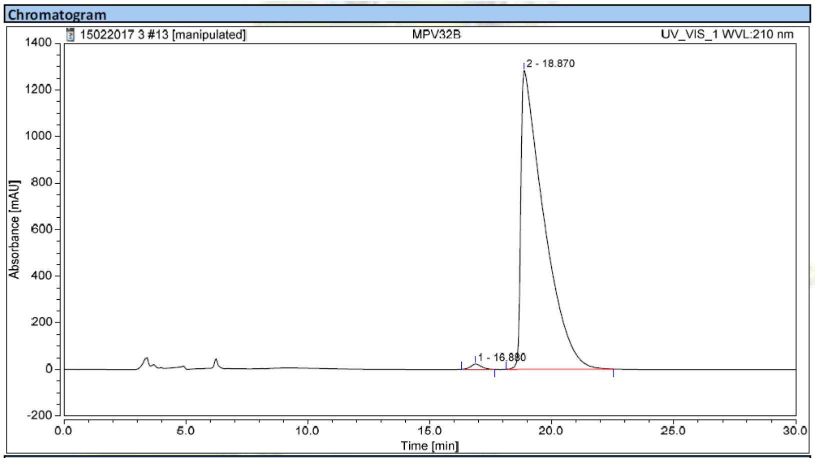


Integration Results

| No. | Peak Name | Retention Time min | Area mAU*min | Height mAU | Relative Area % | Relative Height % | Amount n.a. |
|---|---|---|---|---|---|---|---|
| 1 | | 16.880 | 10.995 | 21.961 | 0.78 | 1.68 | n.a. |
| 2 | | 18.870 | 1396.542 | 1282.962 | 99.22 | 98.32 | n.a. |
| **Total:** | | | **1407.537** | **1304.924** | **100.00** | **100.00** | |

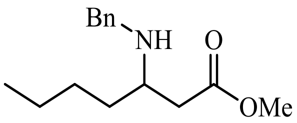


*rac*-**1d**

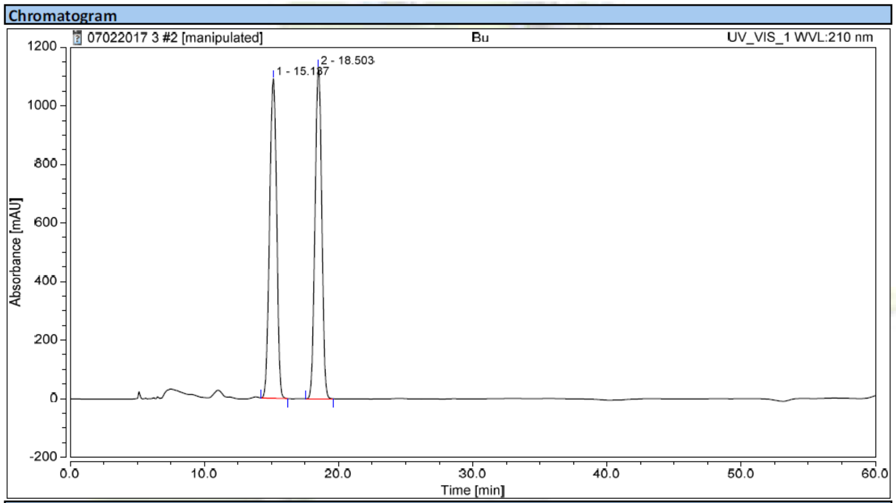


**Integration Results**

| No. | Peak Name | Retention Time min | Area mAU*min | Height mAU | Relative Area % | Relative Height % | Amount n.a. |
|---|---|---|---|---|---|---|---|
| 1 | | 15.137 | 662.031 | 1090.083 | 50.30 | 49.12 | n.a. |
| 2 | | 18.503 | 654.053 | 1128.935 | 49.70 | 50.88 | n.a. |
| **Total:** | | | **1316.084** | **2219.019** | **100.00** | **100.00** | |

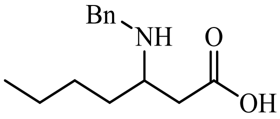


*rac*-**2d**

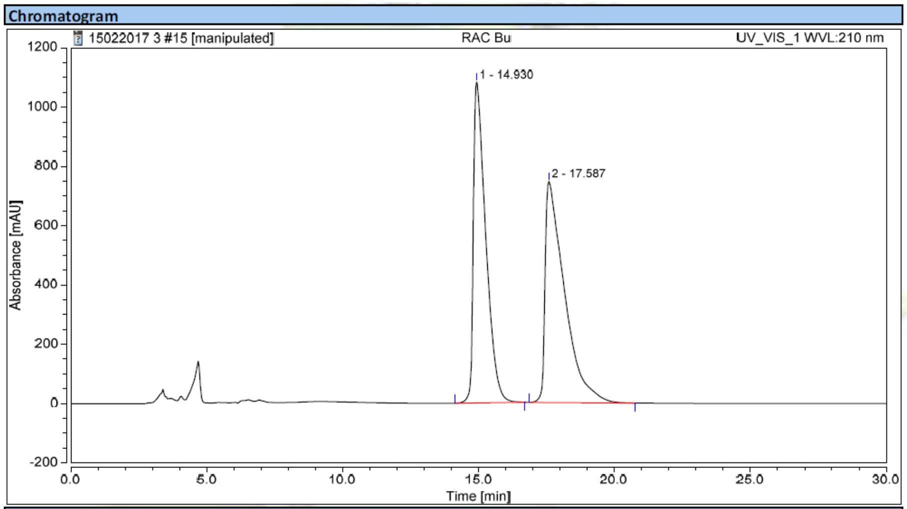


**Integration Results**

| No. | Peak Name | Retention Time min | Area mAU*min | Height mAU | Relative Area % | Relative Height % | Amount n.a. |
|---|---|---|---|---|---|---|---|
| 1 | | 14.930 | 571.538 | 1082.133 | 48.13 | 59.17 | n.a. |
| 2 | | 17.587 | 615.997 | 746.785 | 51.87 | 40.83 | n.a. |
| **Total:** | | | **1187.535** | **1828.918** | **100.00** | **100.00** | |

**For entry 3.**

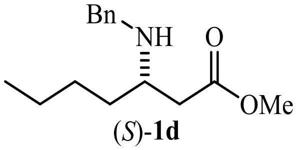


(*S*)-**1d**

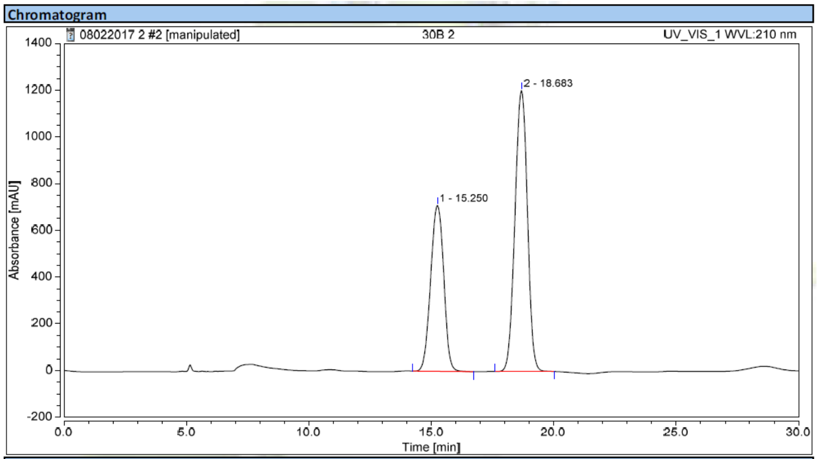


Integration Results

| No. | Peak Name | Retention Time min | Area mAU*min | Height mAU | Relative Area % | Relative Height % | Amount n.a. |
|---|---|---|---|---|---|---|---|
| 1 | | 15.250 | 440.385 | 711.040 | 38.30 | 37.13 | n.a. |
| 2 | | 18.683 | 709.586 | 1204.201 | 61.70 | 62.87 | n.a. |
| **Total:** | | | **1149.971** | **1915.241** | **100.00** | **100.00** | |

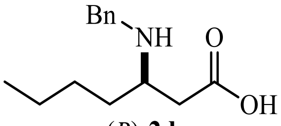


(*R*)-**2d**

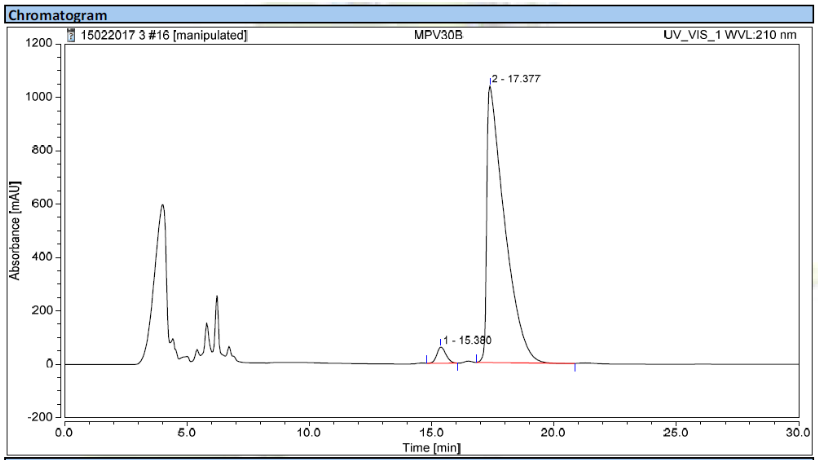


Integration Results

| No. | Peak Name | Retention Time min | Area mAU*min | Height mAU | Relative Area % | Relative Height % | Amount n.a. |
|---|---|---|---|---|---|---|---|
| 1 | | 15.380 | 26.131 | 60.623 | 2.92 | 5.53 | n.a. |
| 2 | | 17.377 | 868.954 | 1035.936 | 97.08 | 94.47 | n.a. |
| **Total:** | | | **895.085** | **1096.559** | **100.00** | **100.00** | |

**For entry 4.**

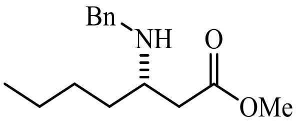


(*S*)-**1d**

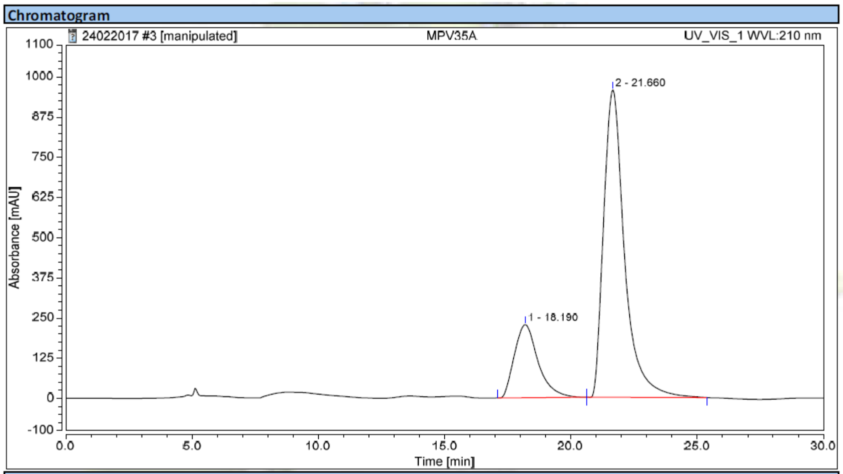


Integration Results

| No. | Peak Name | Retention Time min | Area mAU*min | Height mAU | Relative Area % | Relative Height % | Amount n.a. |
|---|---|---|---|---|---|---|---|
| 1 | | 18.190 | 248.053 | 227.708 | 21.28 | 19.22 | n.a. |
| 2 | | 21.660 | 917.817 | 956.889 | 78.72 | 80.78 | n.a. |
| **Total:** | | | **1165.870** | **1184.597** | **100.00** | **100.00** | |

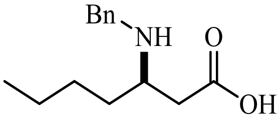


(*R*)-**2d**

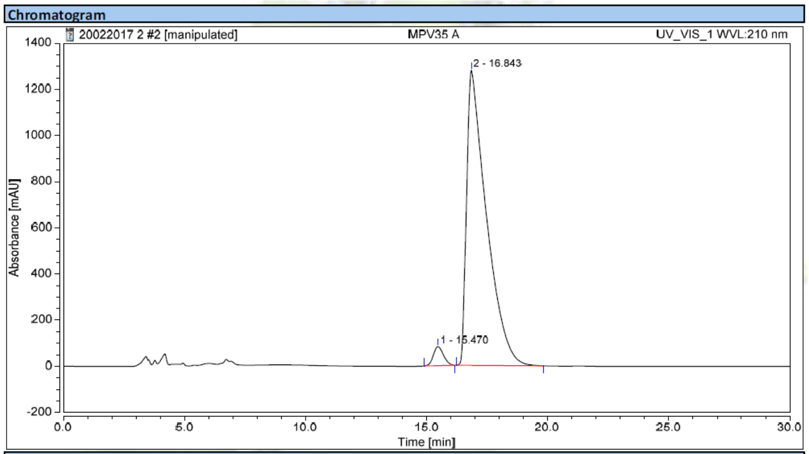


Integration Results

| No. | Peak Name | Retention Time min | Area mAU*min | Height mAU | Relative Area % | Relative Height % | Amount n.a. |
|---|---|---|---|---|---|---|---|
| 1 | | 15.470 | 39.917 | 82.793 | 3.19 | 6.08 | n.a. |
| 2 | | 16.843 | 1210.829 | 1279.227 | 96.81 | 93.92 | n.a. |
| **Total:** | | | **1250.746** | **1362.020** | **100.00** | **100.00** | |

**For entry 5.**

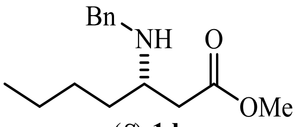


(*S*)-**1d**

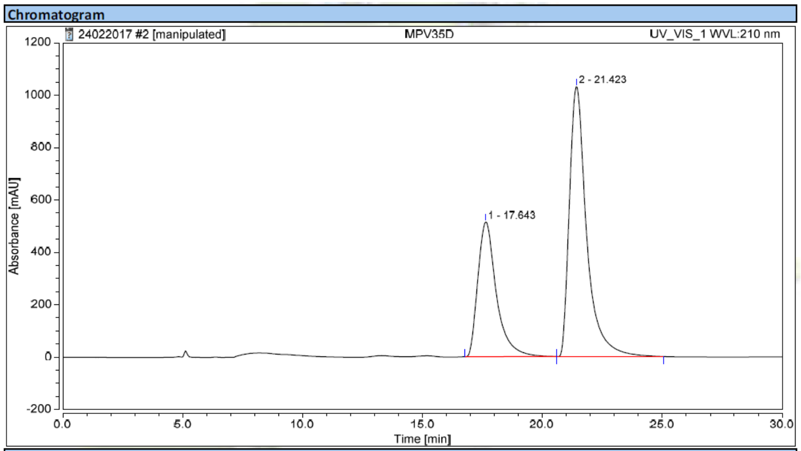


**Integration Results**

| No. | Peak Name | Retention Time min | Area mAU*min | Height mAU | Relative Area % | Relative Height % | Amount n.a. |
|---|---|---|---|---|---|---|---|
| 1 | | 17.643 | 460.277 | 514.746 | 34.93 | 33.28 | n.a. |
| 2 | | 21.423 | 857.341 | 1031.838 | 65.07 | 66.72 | n.a. |
| **Total:** | | | **1317.617** | **1546.583** | **100.00** | **100.00** | |

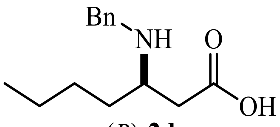


(*R*)-**2d**

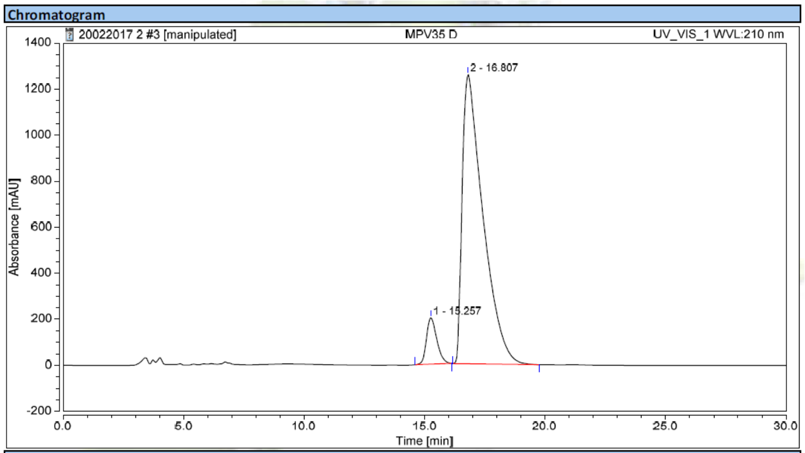


**Integration Results**

| No. | Peak Name | Retention Time min | Area mAU*min | Height mAU | Relative Area % | Relative Height % | Amount n.a. |
|---|---|---|---|---|---|---|---|
| 1 | | 15.257 | 100.306 | 201.792 | 7.42 | 13.83 | n.a. |
| 2 | | 16.807 | 1252.327 | 1257.107 | 92.58 | 86.17 | n.a. |
| **Total:** | | | **1352.633** | **1458.899** | **100.00** | **100.00** | |

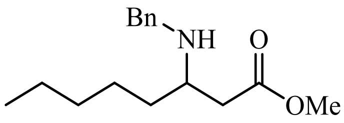

*rac*-**1e**

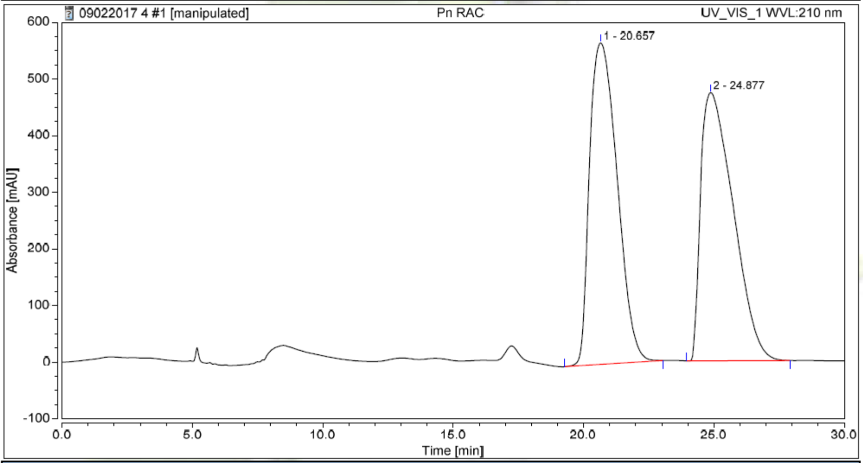


Integration Results

| No. | Peak Name | Retention Time min | Area mAU*min | Height mAU | Relative Area % | Relative Height % | Amount n.a. |
|---|---|---|---|---|---|---|---|
| 1 | | 20.657 | 695.448 | 567.757 | 50.37 | 54.52 | n.a. |
| 2 | | 24.877 | 685.210 | 473.576 | 49.63 | 45.48 | n.a. |
| Total: | | | 1380.657 | 1041.333 | 100.00 | 100.00 | |

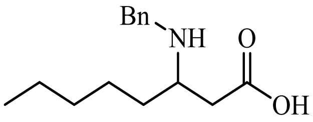

*rac*-**2e**

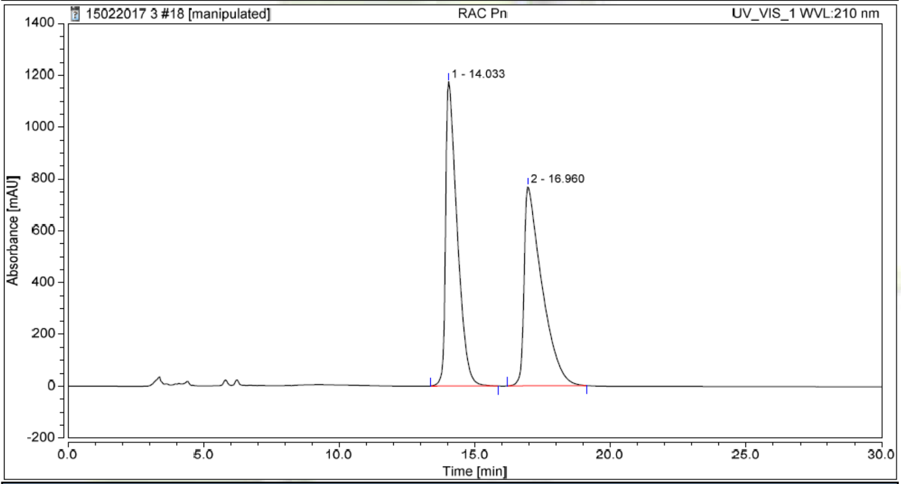


Integration Results

| No. | Peak Name | Retention Time min | Area mAU*min | Height mAU | Relative Area % | Relative Height % | Amount n.a. |
|---|---|---|---|---|---|---|---|
| 1 | | 14.033 | 579.628 | 1174.789 | 49.77 | 60.41 | n.a. |
| 2 | | 16.960 | 584.933 | 769.925 | 50.23 | 39.59 | n.a. |
| Total: | | | 1164.561 | 1944.715 | 100.00 | 100.00 | |

**For entry 6.**

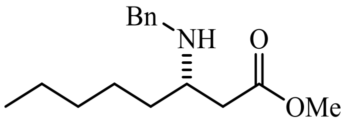

(*S*)-**1e**

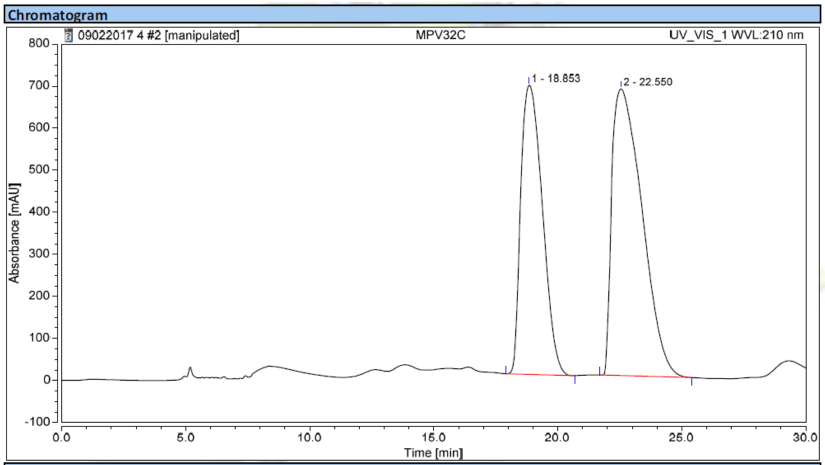


Integration Results

| No. | Peak Name | Retention Time min | Area mAU*min | Height mAU | Relative Area % | Relative Height % | Amount n.a. |
|---|---|---|---|---|---|---|---|
| 1 | | 18.853 | 695.731 | 688.045 | 41.96 | 50.22 | n.a. |
| 2 | | 22.550 | 962.181 | 681.963 | 58.04 | 49.78 | n.a. |
| Total: | | | 1657.912 | 1370.008 | 100.00 | 100.00 | |

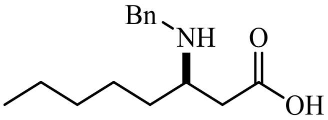

(*R*)-**2e**

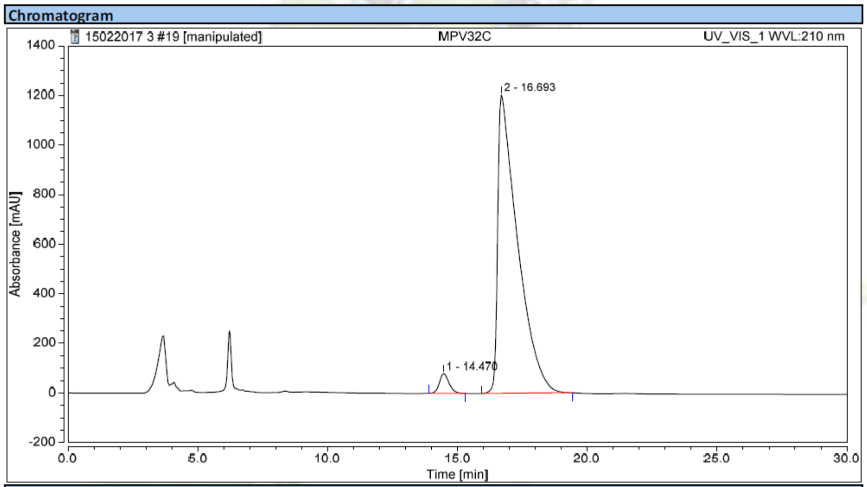


Integration Results

| No. | Peak Name | Retention Time min | Area mAU*min | Height mAU | Relative Area % | Relative Height % | Amount n.a. |
|---|---|---|---|---|---|---|---|
| 1 | | 14.470 | 34.387 | 79.297 | 3.13 | 6.18 | n.a. |
| 2 | | 16.693 | 1065.069 | 1204.825 | 96.87 | 93.82 | n.a. |
| Total: | | | 1099.456 | 1284.122 | 100.00 | 100.00 | |

**For entry 7.**

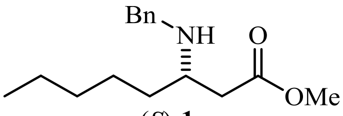


(*S*)-**1e**

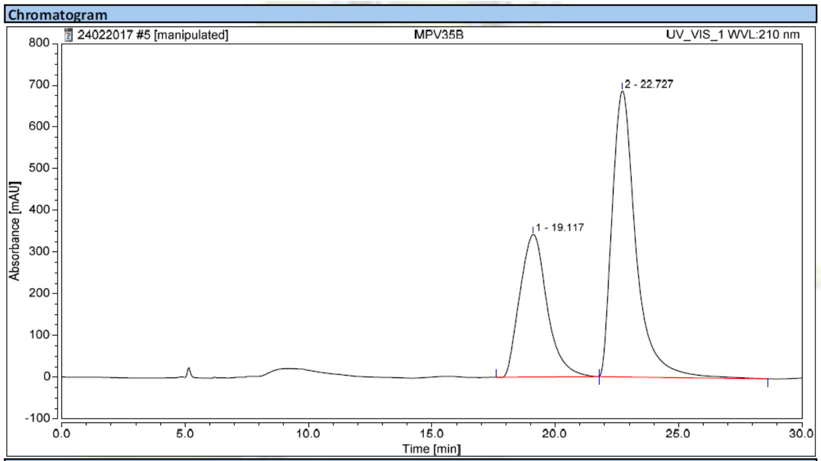


**Integration Results**

| No. | Peak Name | Retention Time min | Area mAU*min | Height mAU | Relative Area % | Relative Height % | Amount n.a. |
|---|---|---|---|---|---|---|---|
| 1 | | 19.117 | 437.471 | 342.127 | 36.34 | 33.26 | n.a. |
| 2 | | 22.727 | 766.410 | 686.432 | 63.66 | 66.74 | n.a. |
| **Total:** | | | **1203.881** | **1028.559** | **100.00** | **100.00** | |

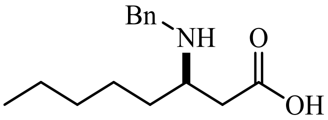


(*R*)-**2e**

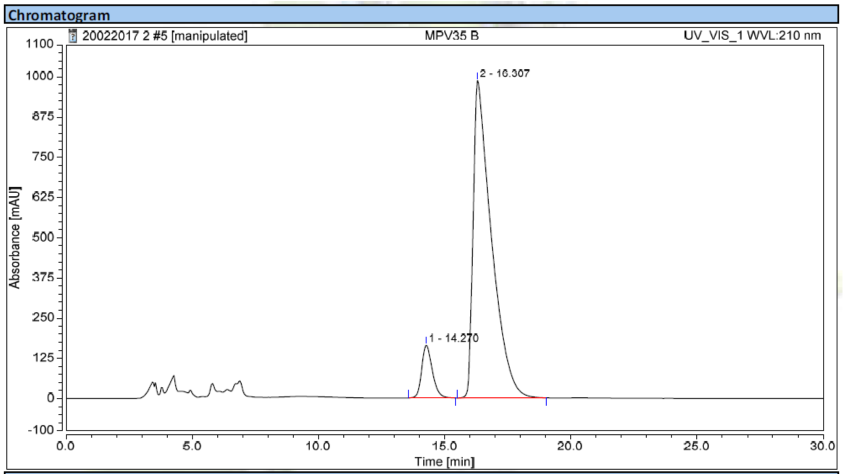


**Integration Results**

| No. | Peak Name | Retention Time min | Area mAU*min | Height mAU | Relative Area % | Relative Height % | Amount n.a. |
|---|---|---|---|---|---|---|---|
| 1 | | 14.270 | 80.210 | 164.459 | 8.84 | 14.29 | n.a. |
| 2 | | 16.307 | 827.187 | 986.707 | 91.16 | 85.71 | n.a. |
| **Total:** | | | **907.397** | **1151.166** | **100.00** | **100.00** | |

**For entry 8.**

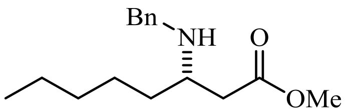


(*S*)-**1e**

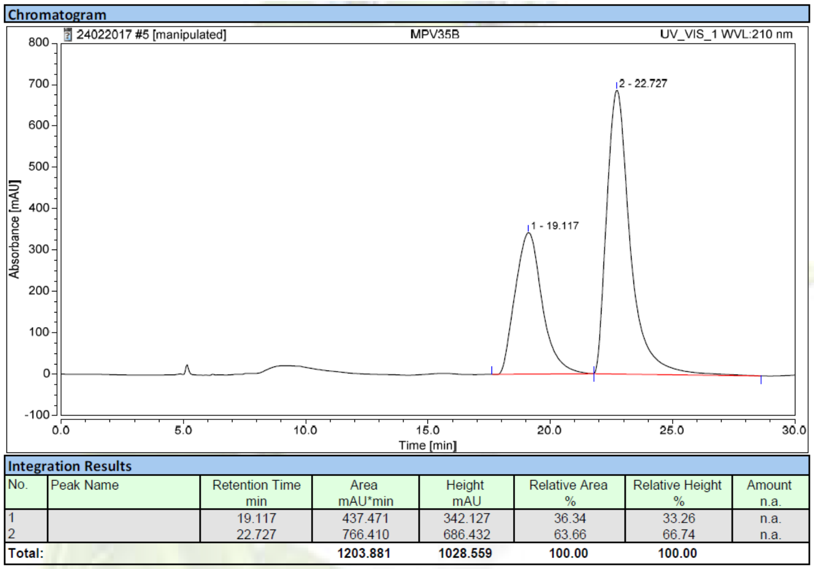


**Integration Results**

| No. | Peak Name | Retention Time<br>min | Area<br>mAU*min | Height<br>mAU | Relative Area<br>% | Relative Height<br>% | Amount<br>n.a. |
|---|---|---|---|---|---|---|---|
| 1 | | 19.117 | 437.471 | 342.127 | 36.34 | 33.26 | n.a. |
| 2 | | 22.727 | 766.410 | 686.432 | 63.66 | 66.74 | n.a. |
| **Total:** | | | **1203.881** | **1028.559** | **100.00** | **100.00** | |

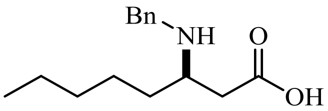


(*R*)-**2e**

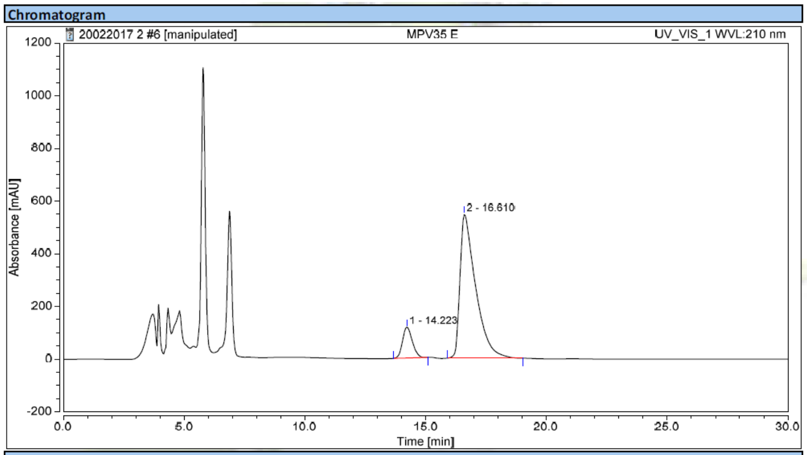


**Integration Results**

| No. | Peak Name | Retention Time<br>min | Area<br>mAU*min | Height<br>mAU | Relative Area<br>% | Relative Height<br>% | Amount<br>n.a. |
|---|---|---|---|---|---|---|---|
| 1 | | 14.223 | 54.586 | 117.205 | 11.79 | 17.68 | n.a. |
| 2 | | 16.610 | 408.540 | 545.777 | 88.21 | 82.32 | n.a. |
| **Total:** | | | **463.126** | **662.982** | **100.00** | **100.00** | |

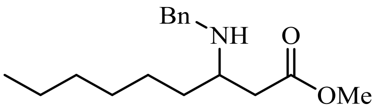


*rac*-**1f**

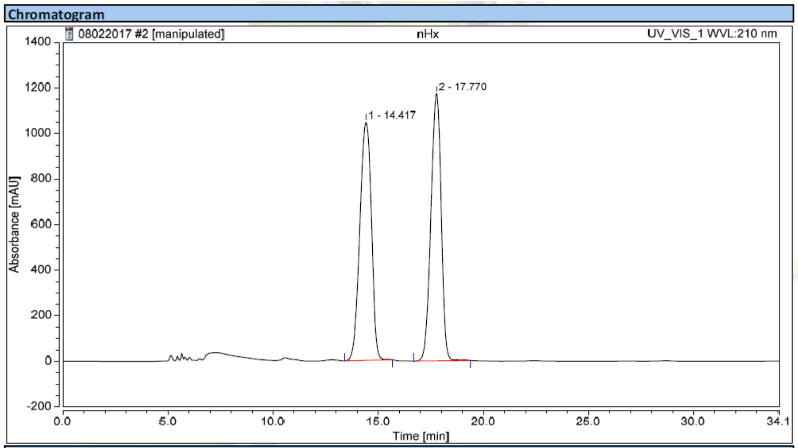


**Integration Results**

| No. | Peak Name | Retention Time min | Area mAU*min | Height mAU | Relative Area % | Relative Height % | Amount n.a. |
|---|---|---|---|---|---|---|---|
| 1 | | 14.417 | 669.334 | 1046.779 | 50.27 | 47.14 | n.a. |
| 2 | | 17.770 | 662.152 | 1173.847 | 49.73 | 52.86 | n.a. |
| Total: | | | 1331.486 | 2220.625 | 100.00 | 100.00 | |

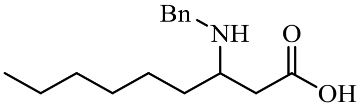


*rac*-**2f**

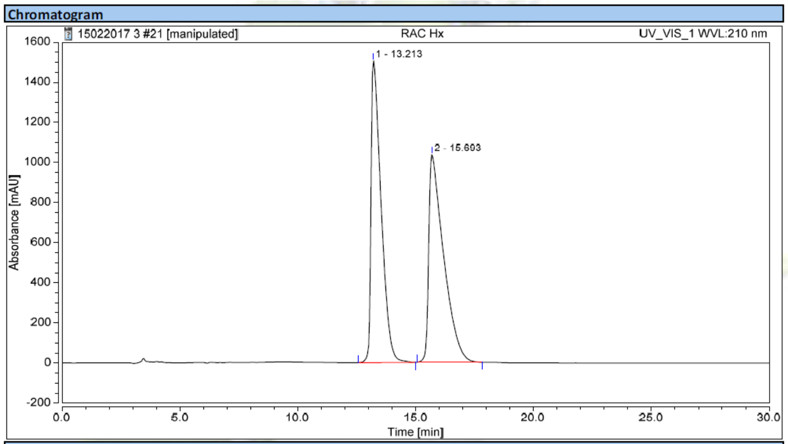


**Integration Results**

| No. | Peak Name | Retention Time min | Area mAU*min | Height mAU | Relative Area % | Relative Height % | Amount n.a. |
|---|---|---|---|---|---|---|---|
| 1 | | 13.213 | 744.585 | 1506.831 | 49.60 | 59.24 | n.a. |
| 2 | | 15.693 | 756.713 | 1036.692 | 50.40 | 40.76 | n.a. |
| Total: | | | 1501.298 | 2543.523 | 100.00 | 100.00 | |

**For entry 9.**

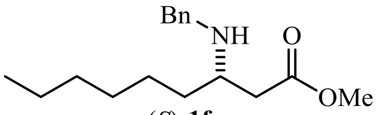


(*S*)-**1f**

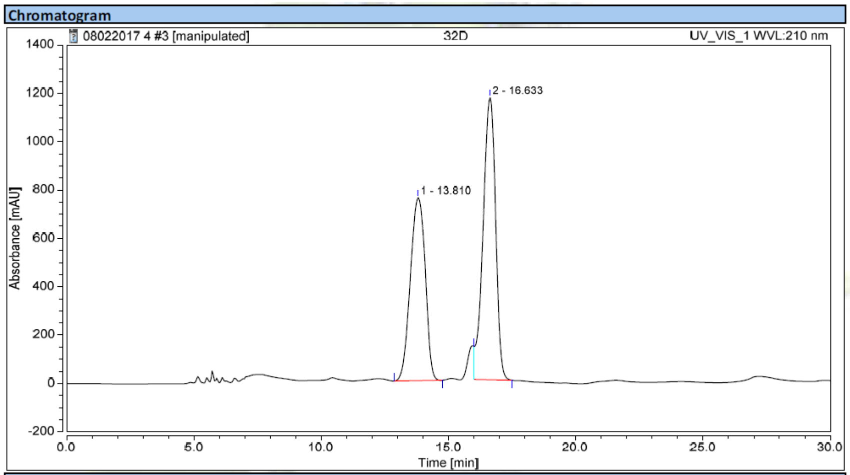


Integration Results

| No. | Peak Name | Retention Time min | Area mAU*min | Height mAU | Relative Area % | Relative Height % | Amount n.a. |
|---|---|---|---|---|---|---|---|
| 1 | | 13.810 | 512.845 | 757.376 | 43.45 | 39.33 | n.a. |
| 2 | | 16.633 | 667.419 | 1168.452 | 56.55 | 60.67 | n.a. |
| **Total:** | | | **1180.264** | **1925.828** | **100.00** | **100.00** | |

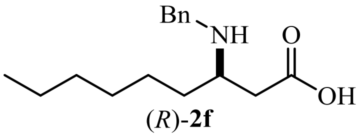


(*R*)-**2f**

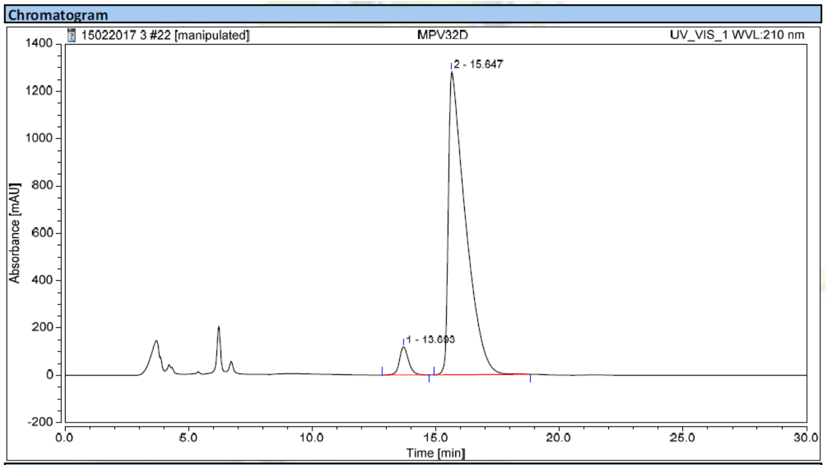


Integration Results

| No. | Peak Name | Retention Time min | Area mAU*min | Height mAU | Relative Area % | Relative Height % | Amount n.a. |
|---|---|---|---|---|---|---|---|
| 1 | | 13.693 | 50.504 | 119.174 | 4.68 | 8.50 | n.a. |
| 2 | | 15.647 | 1028.436 | 1282.751 | 95.32 | 91.50 | n.a. |
| **Total:** | | | **1078.940** | **1401.925** | **100.00** | **100.00** | |

**For entry 10.**

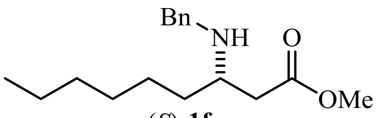


(*S*)-**1f**

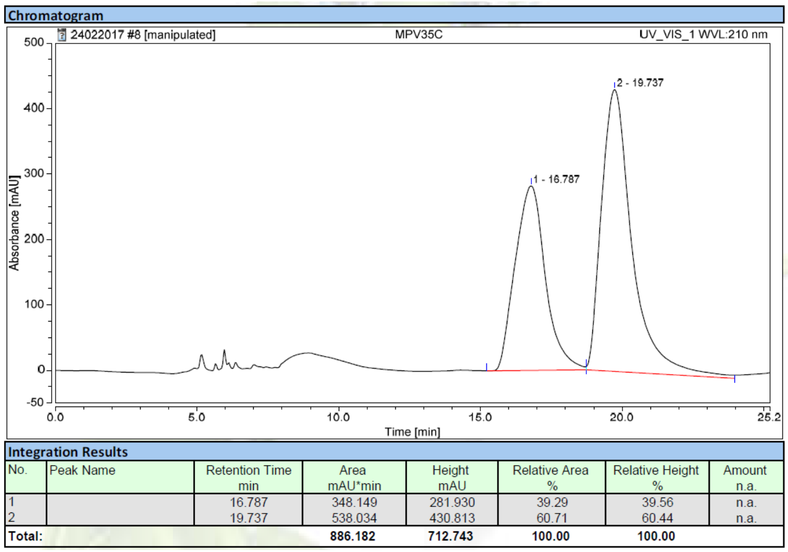


| Integration Results | | | | | | | |
|---|---|---|---|---|---|---|---|
| No. | Peak Name | Retention Time min | Area mAU*min | Height mAU | Relative Area % | Relative Height % | Amount n.a. |
| 1 | | 16.787 | 348.149 | 281.930 | 39.29 | 39.56 | n.a. |
| 2 | | 19.737 | 538.034 | 430.813 | 60.71 | 60.44 | n.a. |
| Total: | | | 886.182 | 712.743 | 100.00 | 100.00 | |

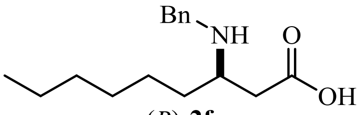


(*R*)-**2f**

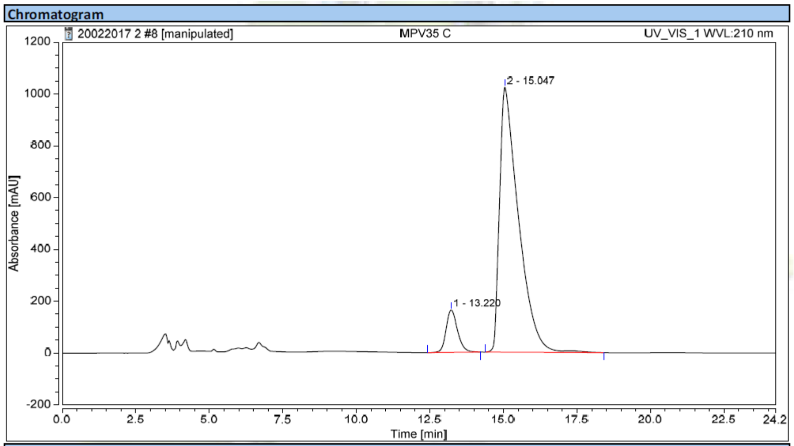


| Integration Results | | | | | | | |
|---|---|---|---|---|---|---|---|
| No. | Peak Name | Retention Time min | Area mAU*min | Height mAU | Relative Area % | Relative Height % | Amount n.a. |
| 1 | | 13.220 | 73.565 | 163.838 | 8.81 | 13.81 | n.a. |
| 2 | | 15.047 | 761.183 | 1022.806 | 91.19 | 86.19 | n.a. |
| Total: | | | 834.748 | 1186.645 | 100.00 | 100.00 | |

**For entry 11.**

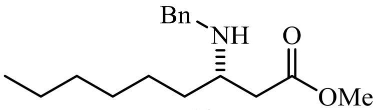


(*S*)-**1f**

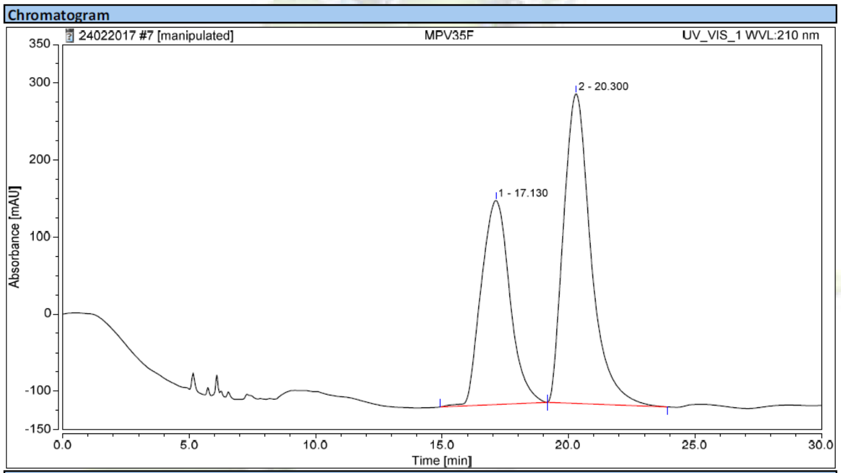


**Integration Results**

| No. | Peak Name | Retention Time min | Area mAU*min | Height mAU | Relative Area % | Relative Height % | Amount n.a. |
|---|---|---|---|---|---|---|---|
| 1 | | 17.130 | 347.593 | 264.963 | 40.81 | 39.71 | n.a. |
| 2 | | 20.300 | 504.212 | 402.237 | 59.19 | 60.29 | n.a. |
| **Total:** | | | **851.804** | **667.201** | **100.00** | **100.00** | |

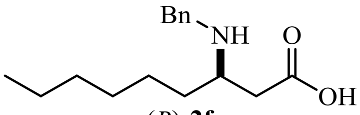


(*R*)-**2f**

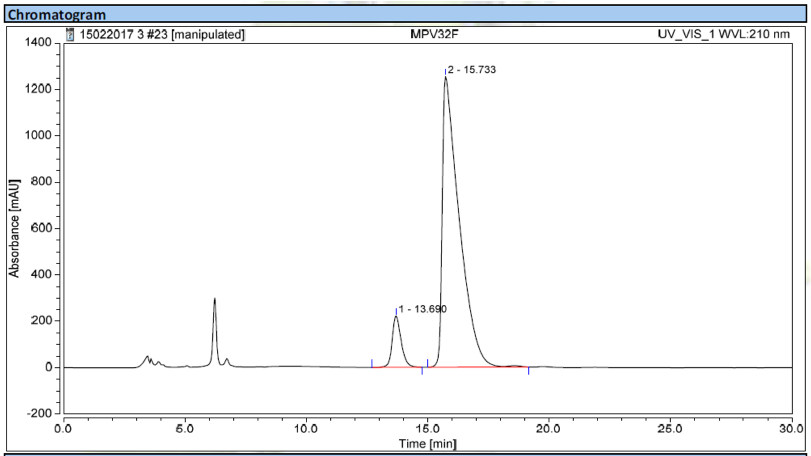


**Integration Results**

| No. | Peak Name | Retention Time min | Area mAU*min | Height mAU | Relative Area % | Relative Height % | Amount n.a. |
|---|---|---|---|---|---|---|---|
| 1 | | 13.690 | 94.699 | 221.585 | 8.55 | 15.01 | n.a. |
| 2 | | 15.733 | 1012.385 | 1254.546 | 91.45 | 84.99 | n.a. |
| **Total:** | | | **1107.084** | **1476.131** | **100.00** | **100.00** | |

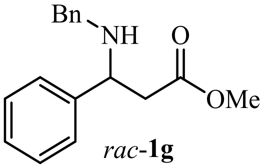

*rac*-**1g**

Chromatogram

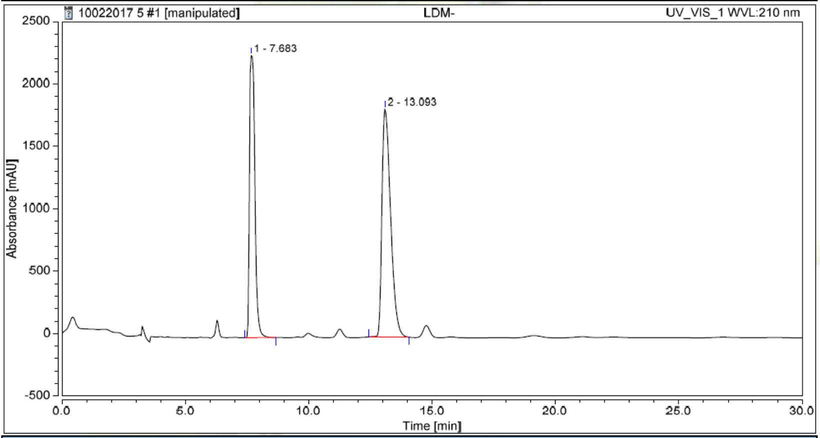


Integration Results

| No. | Peak Name | Retention Time min | Area mAU*min | Height mAU | Relative Area % | Relative Height % | Amount n.a. |
|---|---|---|---|---|---|---|---|
| 1 | | 7.683 | 575.296 | 2264.166 | 43.60 | 55.31 | n.a. |
| 2 | | 13.093 | 744.149 | 1829.154 | 56.40 | 44.69 | n.a. |
| **Total:** | | | **1319.445** | **4093.320** | **100.00** | **100.00** | |

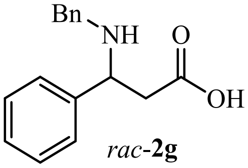

*rac*-**2g**

Chromatogram

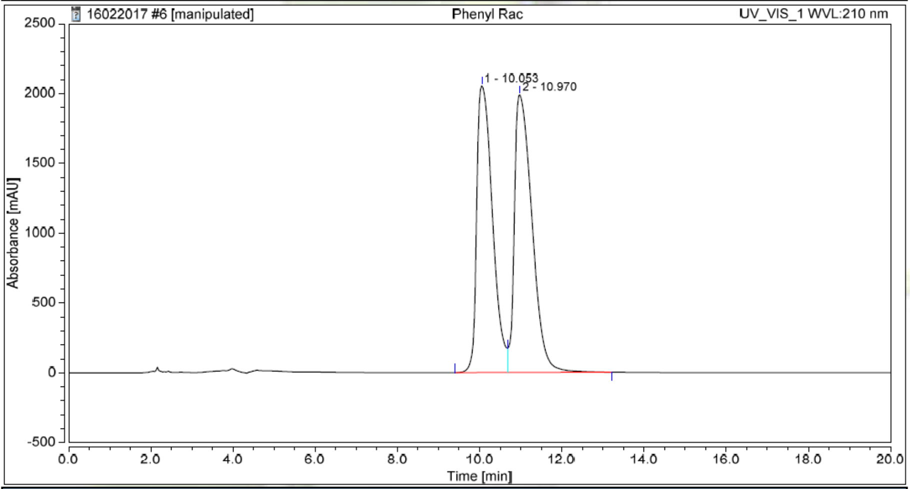


Integration Results

| No. | Peak Name | Retention Time min | Area mAU*min | Height mAU | Relative Area % | Relative Height % | Amount n.a. |
|---|---|---|---|---|---|---|---|
| 1 | | 10.053 | 911.637 | 2055.268 | 47.97 | 50.79 | n.a. |
| 2 | | 10.970 | 988.669 | 1991.275 | 52.03 | 49.21 | n.a. |
| **Total:** | | | **1900.306** | **4046.544** | **100.00** | **100.00** | |

**For entry 12.**

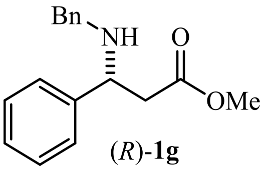

(*R*)-**1g**

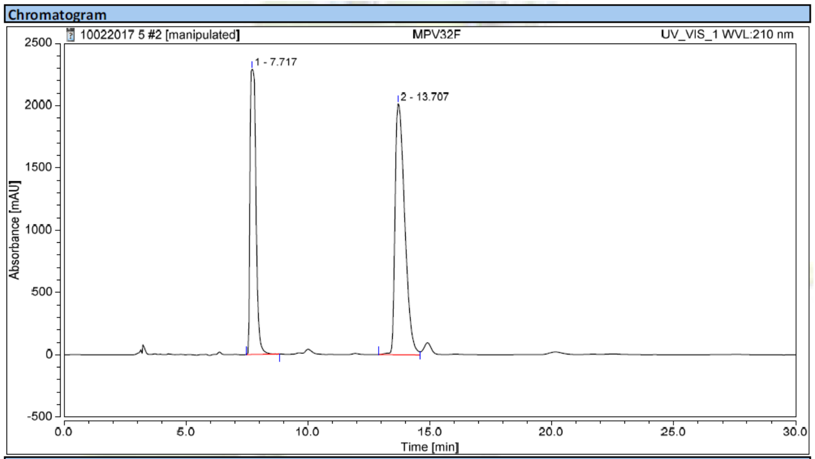


**Integration Results**

| No. | Peak Name | Retention Time min | Area mAU*min | Height mAU | Relative Area % | Relative Height % | Amount n.a. |
|---|---|---|---|---|---|---|---|
| 1 | | 7.717 | 648.985 | 2292.370 | 41.25 | 53.20 | n.a. |
| 2 | | 13.707 | 924.193 | 2016.550 | 58.75 | 46.80 | n.a. |
| **Total:** | | | **1573.178** | **4308.920** | **100.00** | **100.00** | |

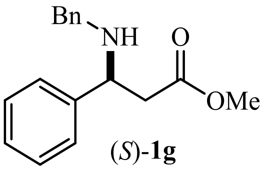

(*S*)-**1g**

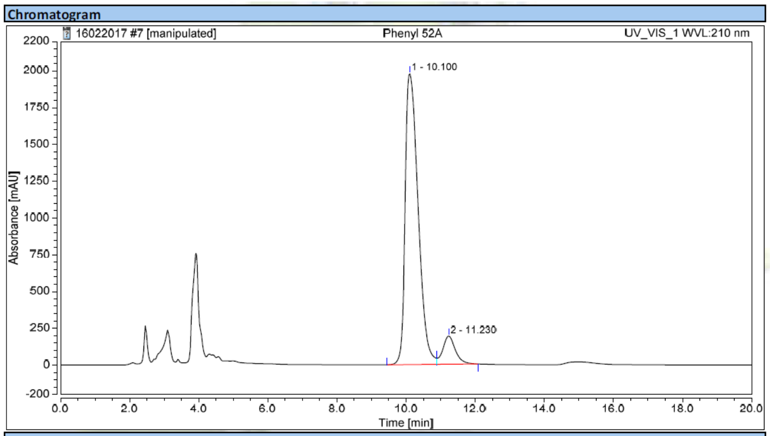


**Integration Results**

| No. | Peak Name | Retention Time min | Area mAU*min | Height mAU | Relative Area % | Relative Height % | Amount n.a. |
|---|---|---|---|---|---|---|---|
| 1 | | 10.100 | 855.620 | 1980.535 | 91.35 | 91.17 | n.a. |
| 2 | | 11.230 | 80.979 | 191.903 | 8.65 | 8.83 | n.a. |
| **Total:** | | | **936.599** | **2172.438** | **100.00** | **100.00** | |

**For entry 13.**

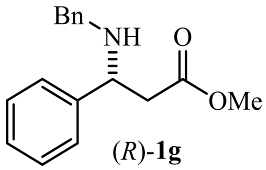

(*R*)-**1g**

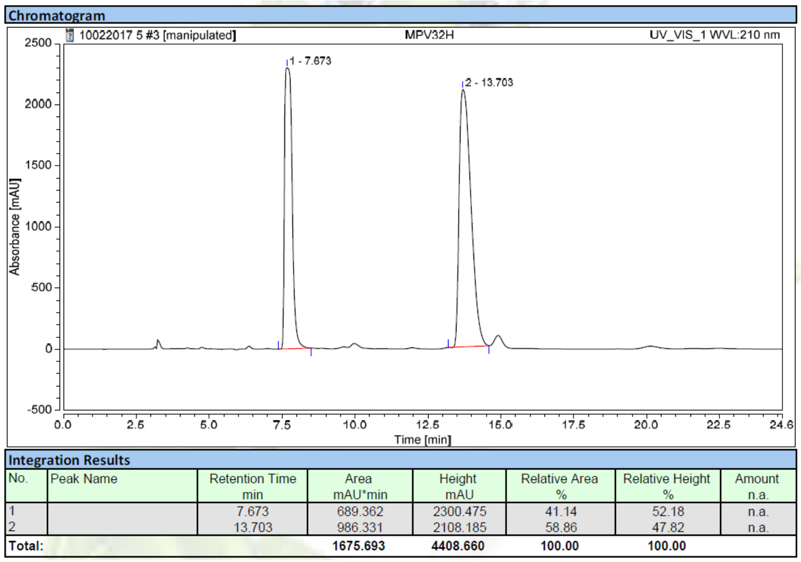

**Integration Results**

| No. | Peak Name | Retention Time min | Area mAU*min | Height mAU | Relative Area % | Relative Height % | Amount n.a. |
|---|---|---|---|---|---|---|---|
| 1 | | 7.673 | 689.362 | 2300.475 | 41.14 | 52.18 | n.a. |
| 2 | | 13.703 | 986.331 | 2108.185 | 58.86 | 47.82 | n.a. |
| Total: | | | 1675.693 | 4408.660 | 100.00 | 100.00 | |

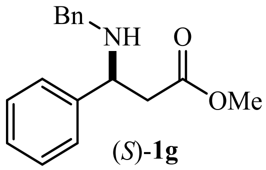

(*S*)-**1g**

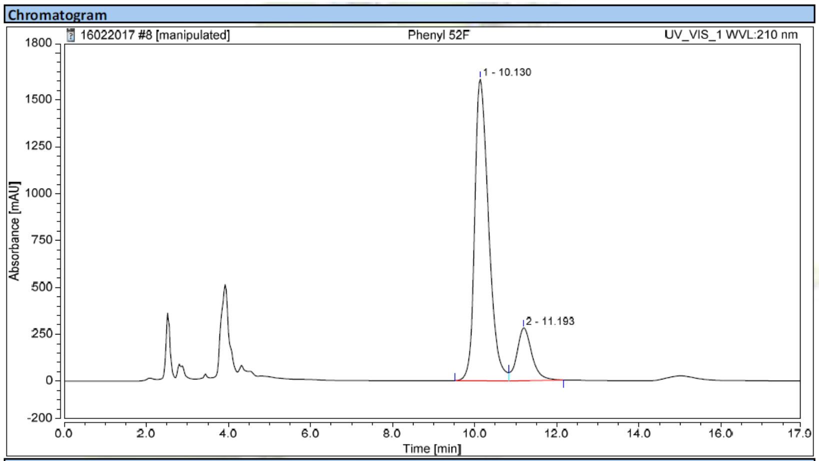

**Integration Results**

| No. | Peak Name | Retention Time min | Area mAU*min | Height mAU | Relative Area % | Relative Height % | Amount n.a. |
|---|---|---|---|---|---|---|---|
| 1 | | 10.130 | 629.952 | 1609.806 | 84.16 | 85.09 | n.a. |
| 2 | | 11.193 | 118.603 | 281.989 | 15.84 | 14.91 | n.a. |
| Total: | | | 748.555 | 1891.795 | 100.00 | 100.00 | |

**For entry 14.**

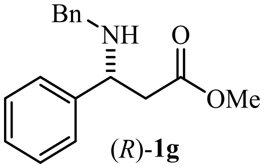


(*R*)-**1g**

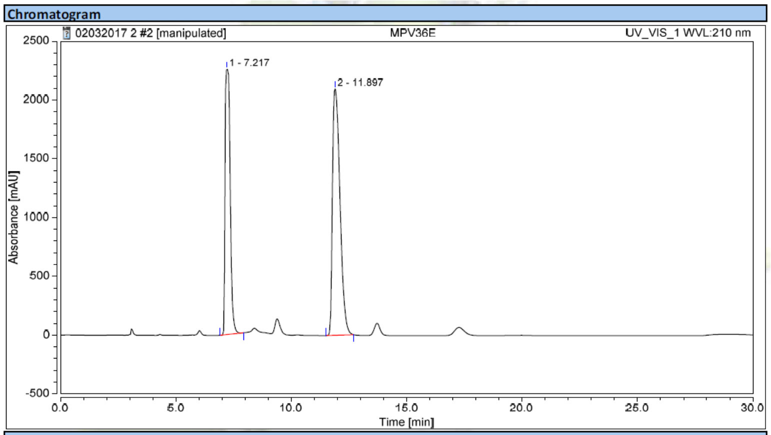


Integration Results

| No. | Peak Name | Retention Time min | Area mAU*min | Height mAU | Relative Area % | Relative Height % | Amount n.a. |
|---|---|---|---|---|---|---|---|
| 1 | | 7.217 | 575.806 | 2259.430 | 42.19 | 51.87 | n.a. |
| 2 | | 11.897 | 789.032 | 2096.621 | 57.81 | 48.13 | n.a. |
| **Total:** | | | **1364.837** | **4356.051** | **100.00** | **100.00** | |

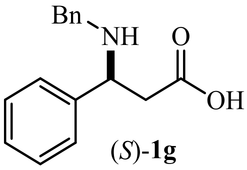


(*S*)-**1g**

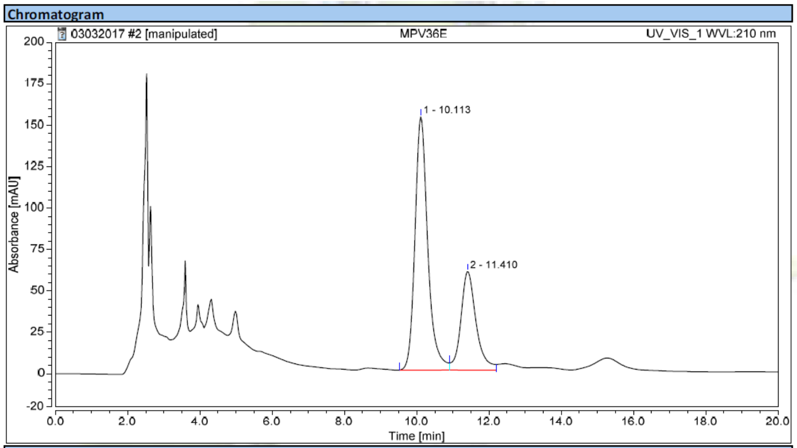


Integration Results

| No. | Peak Name | Retention Time min | Area mAU*min | Height mAU | Relative Area % | Relative Height % | Amount n.a. |
|---|---|---|---|---|---|---|---|
| 1 | | 10.113 | 62.639 | 152.985 | 68.40 | 71.97 | n.a. |
| 2 | | 11.410 | 28.937 | 59.581 | 31.60 | 28.03 | n.a. |
| **Total:** | | | **91.576** | **212.565** | **100.00** | **100.00** | |

**For entry 15.**

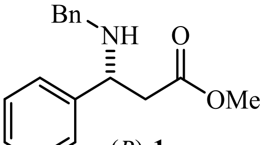


(*R*)-**1g**

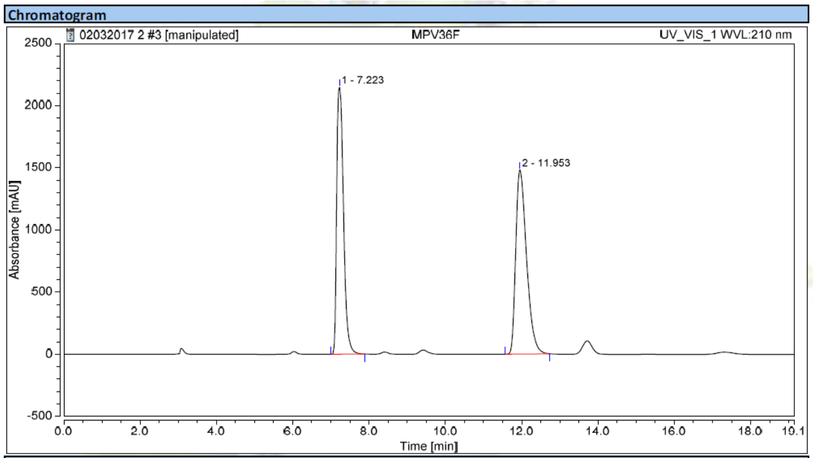


**Integration Results**

| No. | Peak Name | Retention Time min | Area mAU*min | Height mAU | Relative Area % | Relative Height % | Amount n.a. |
|---|---|---|---|---|---|---|---|
| 1 | | 7.223 | 442.323 | 2149.784 | 47.67 | 59.15 | n.a. |
| 2 | | 11.953 | 485.590 | 1484.526 | 52.33 | 40.85 | n.a. |
| **Total:** | | | **927.913** | **3634.310** | **100.00** | **100.00** | |

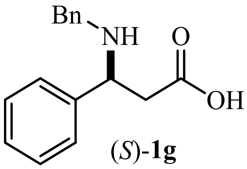


(*S*)-**1g**

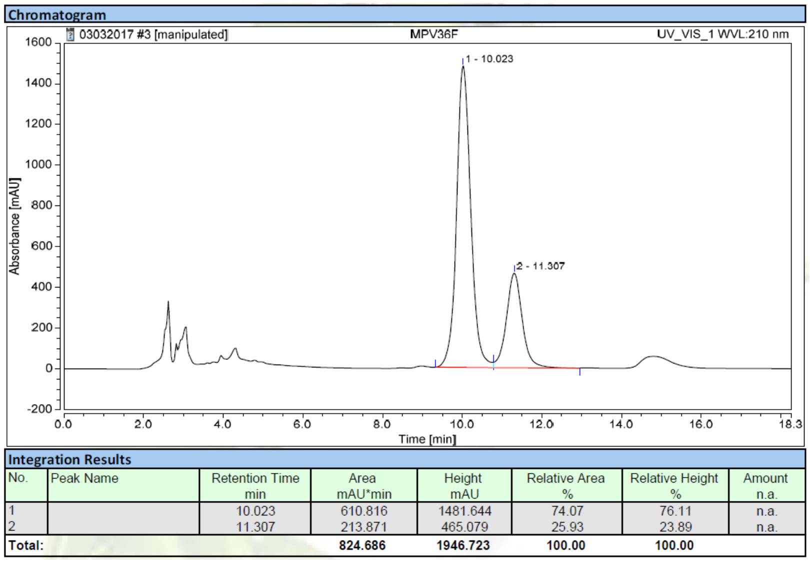


**Integration Results**

| No. | Peak Name | Retention Time min | Area mAU*min | Height mAU | Relative Area % | Relative Height % | Amount n.a. |
|---|---|---|---|---|---|---|---|
| 1 | | 10.023 | 610.816 | 1481.644 | 74.07 | 76.11 | n.a. |
| 2 | | 11.307 | 213.871 | 465.079 | 25.93 | 23.89 | n.a. |
| **Total:** | | | **824.686** | **1946.723** | **100.00** | **100.00** | |

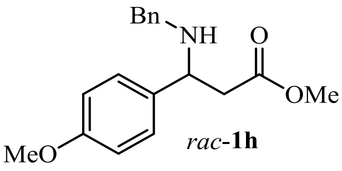


*rac*-**1h**

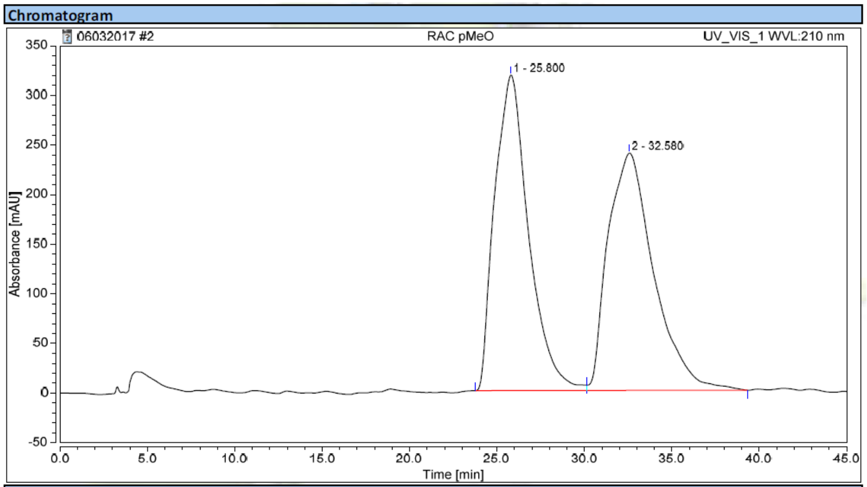


**Integration Results**

| No. | Peak Name | Retention Time min | Area mAU*min | Height mAU | Relative Area % | Relative Height % | Amount n.a. |
|---|---|---|---|---|---|---|---|
| 1 | | 25.800 | 714.671 | 318.077 | 49.67 | 57.07 | n.a. |
| 2 | | 32.580 | 724.084 | 239.269 | 50.33 | 42.93 | n.a. |
| Total: | | | 1438.754 | 557.347 | 100.00 | 100.00 | |

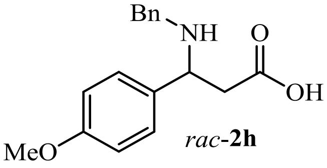


*rac*-**2h**

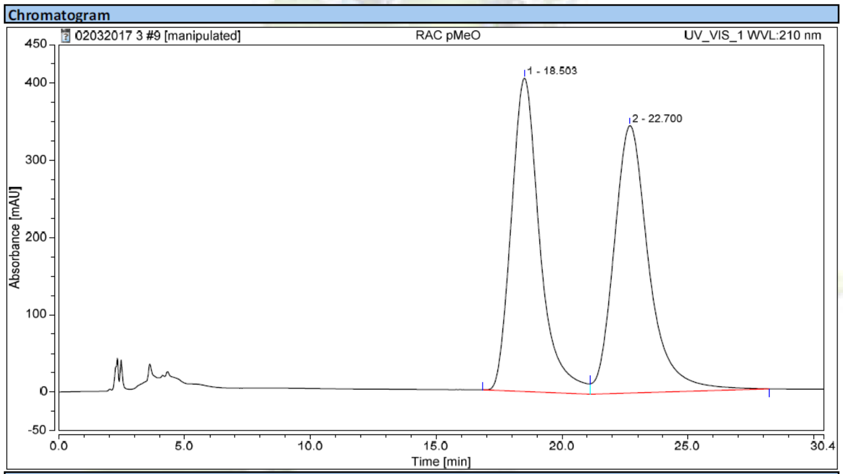


**Integration Results**

| No. | Peak Name | Retention Time min | Area mAU*min | Height mAU | Relative Area % | Relative Height % | Amount n.a. |
|---|---|---|---|---|---|---|---|
| 1 | | 18.503 | 534.089 | 406.076 | 48.90 | 53.96 | n.a. |
| 2 | | 22.700 | 558.163 | 346.477 | 51.10 | 46.04 | n.a. |
| Total: | | | 1092.252 | 752.553 | 100.00 | 100.00 | |

**For entry 16.**

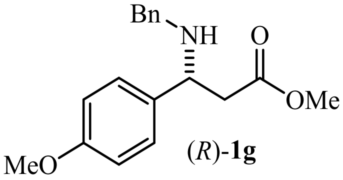


(*R*)-**1g**

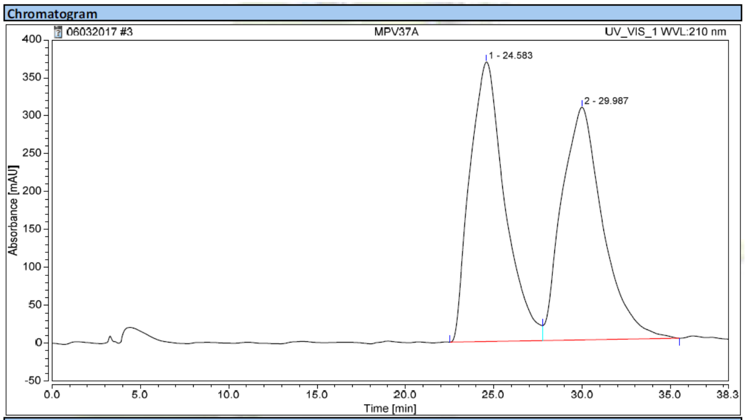


**Integration Results**

| No. | Peak Name | Retention Time min | Area mAU*min | Height mAU | Relative Area % | Relative Height % | Amount n.a. |
|---|---|---|---|---|---|---|---|
| 1 | | 24.583 | 842.045 | 368.822 | 50.44 | 54.57 | n.a. |
| 2 | | 29.987 | 827.432 | 307.072 | 49.56 | 45.43 | n.a. |
| Total: | | | 1669.477 | 675.895 | 100.00 | 100.00 | |

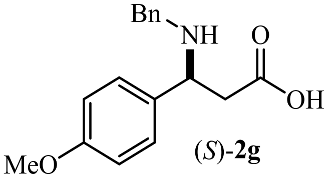


(*S*)-**2g**

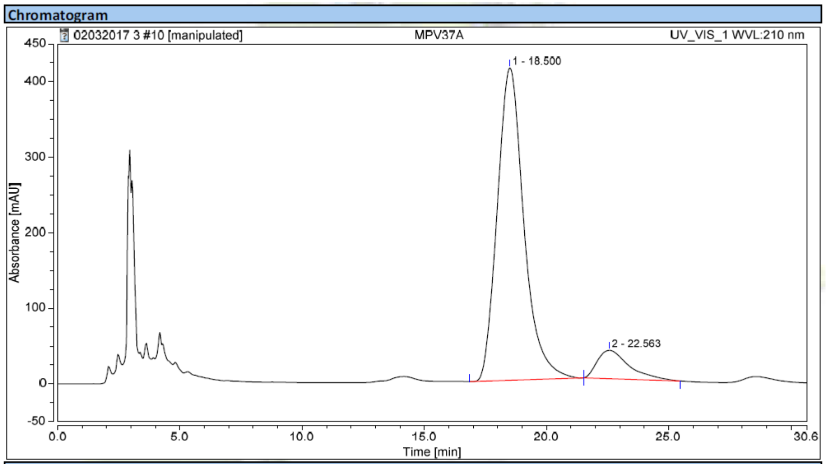


**Integration Results**

| No. | Peak Name | Retention Time min | Area mAU*min | Height mAU | Relative Area % | Relative Height % | Amount n.a. |
|---|---|---|---|---|---|---|---|
| 1 | | 18.500 | 521.347 | 413.777 | 90.19 | 91.69 | n.a. |
| 2 | | 22.563 | 56.687 | 37.507 | 9.81 | 8.31 | n.a. |
| Total: | | | 578.034 | 451.284 | 100.00 | 100.00 | |

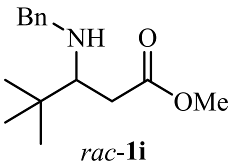


*rac*-**1i**

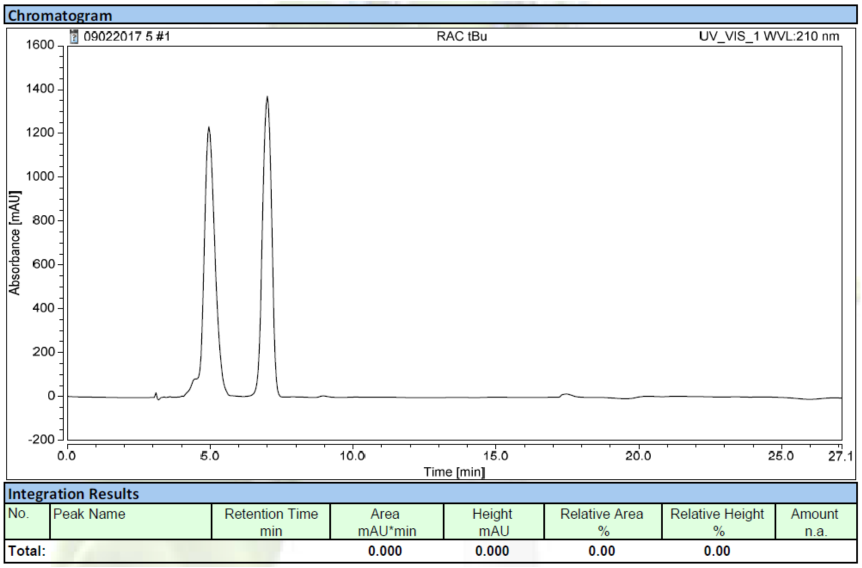


**Integration Results**

| No. | Peak Name | Retention Time min | Area mAU*min | Height mAU | Relative Area % | Relative Height % | Amount n.a. |
|---|---|---|---|---|---|---|---|
| **Total:** | | | **0.000** | **0.000** | **0.00** | **0.00** | |

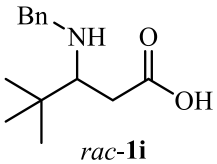


*rac*-**1i**

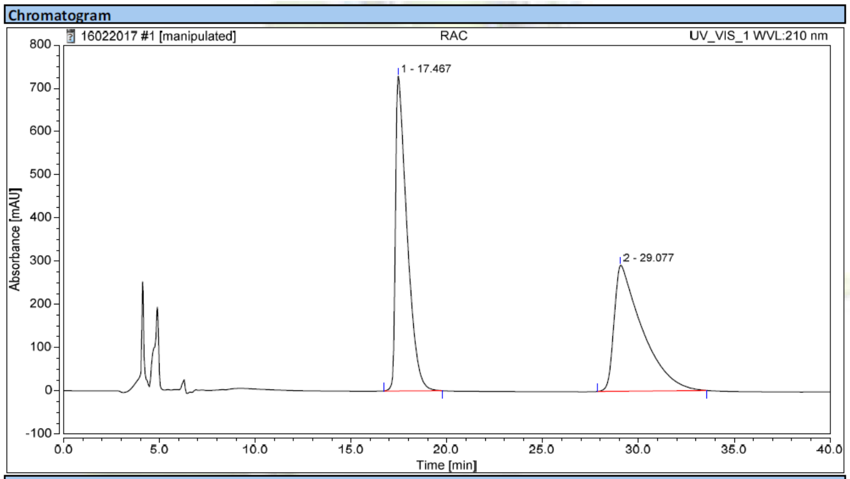


**Integration Results**

| No. | Peak Name | Retention Time min | Area mAU*min | Height mAU | Relative Area % | Relative Height % | Amount n.a. |
|---|---|---|---|---|---|---|---|
| 1 | | 17.467 | 504.303 | 728.813 | 50.45 | 71.38 | n.a. |
| 2 | | 29.077 | 495.300 | 292.180 | 49.55 | 28.62 | n.a. |
| **Total:** | | | **999.604** | **1020.993** | **100.00** | **100.00** | |

**For entry 17.**

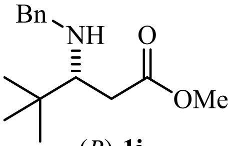


(*R*)-**1i**

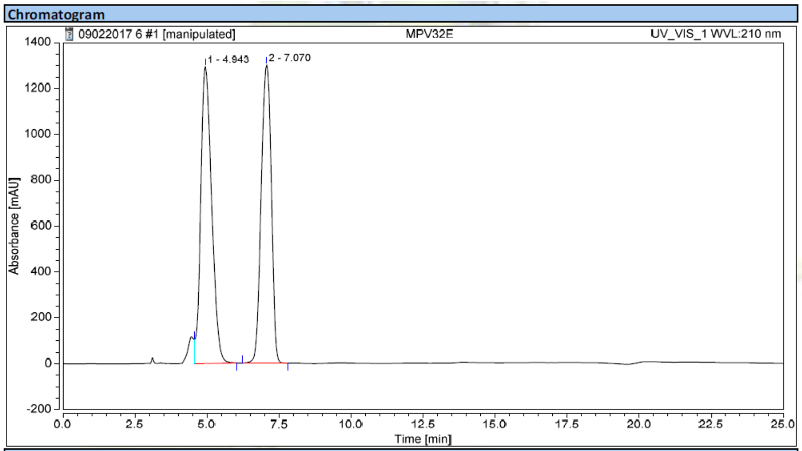


**Integration Results**

| No. | Peak Name | Retention Time min | Area mAU*min | Height mAU | Relative Area % | Relative Height % | Amount n.a. |
|---|---|---|---|---|---|---|---|
| 1 | | 4.943 | 569.716 | 1295.633 | 51.79 | 49.89 | n.a. |
| 2 | | 7.070 | 530.385 | 1301.572 | 48.21 | 50.11 | n.a. |
| **Total:** | | | **1100.101** | **2597.206** | **100.00** | **100.00** | |

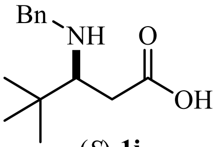


(*S*)-**1i**

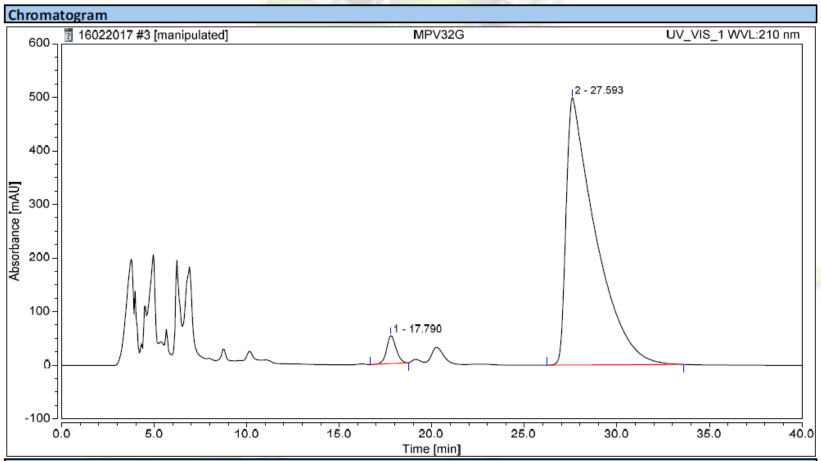


**Integration Results**

| No. | Peak Name | Retention Time min | Area mAU*min | Height mAU | Relative Area % | Relative Height % | Amount n.a. |
|---|---|---|---|---|---|---|---|
| 1 | | 17.790 | 30.531 | 51.968 | 3.23 | 9.42 | n.a. |
| 2 | | 27.593 | 916.024 | 499.535 | 96.77 | 90.58 | n.a. |
| **Total:** | | | **946.554** | **551.503** | **100.00** | **100.00** | |

**For entry 18.**

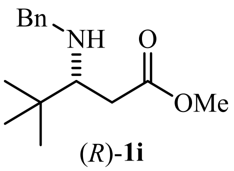


(*R*)-**1i**

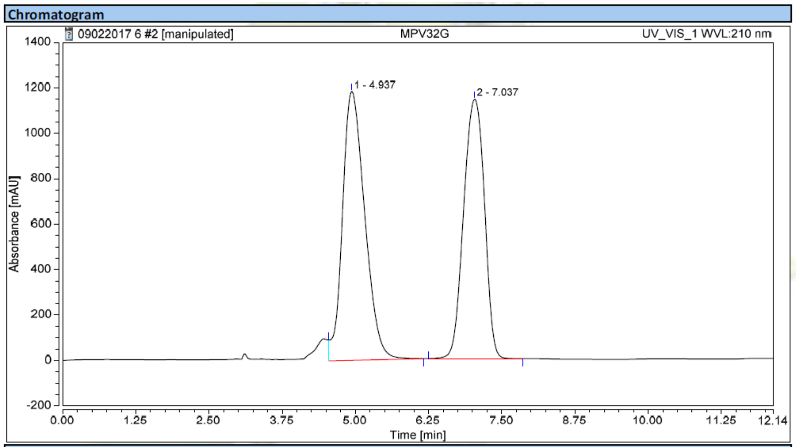


Integration Results

| No. | Peak Name | Retention Time min | Area mAU*min | Height mAU | Relative Area % | Relative Height % | Amount n.a. |
|---|---|---|---|---|---|---|---|
| 1 | | 4.937 | 522.647 | 1184.487 | 52.61 | 50.89 | n.a. |
| 2 | | 7.037 | 470.776 | 1143.065 | 47.39 | 49.11 | n.a. |
| **Total:** | | | **993.423** | **2327.552** | **100.00** | **100.00** | |

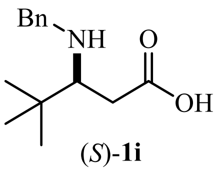


(*S*)-**1i**

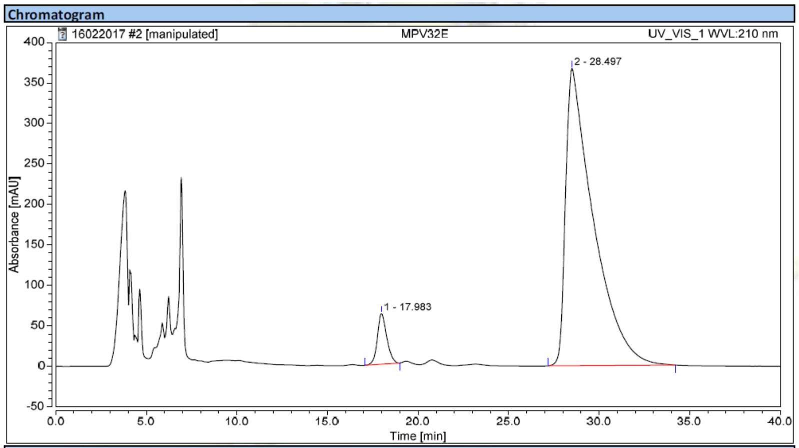


Integration Results

| No. | Peak Name | Retention Time min | Area mAU*min | Height mAU | Relative Area % | Relative Height % | Amount n.a. |
|---|---|---|---|---|---|---|---|
| 1 | | 17.983 | 37.731 | 62.562 | 5.47 | 14.55 | n.a. |
| 2 | | 28.497 | 651.750 | 367.461 | 94.53 | 85.45 | n.a. |
| **Total:** | | | **689.481** | **430.023** | **100.00** | **100.00** | |

- **Chromatograms for the evaluation of recycling capacity of immobilized CALB (Table S3).**

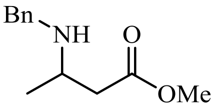


*rac*-**1a**

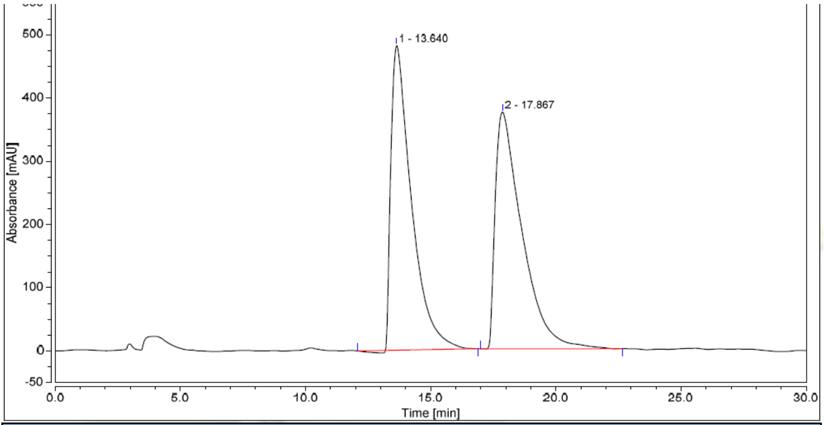


| Integration Results | | | | | | | |
|---|---|---|---|---|---|---|---|
| No. | Peak Name | Retention Time min | Area mAU*min | Height mAU | Relative Area % | Relative Height % | Amount n.a. |
| 1 | | 13.640 | 455.884 | 482.090 | 49.63 | 56.27 | n.a. |
| 2 | | 17.867 | 462.617 | 374.599 | 50.37 | 43.73 | n.a. |
| **Total:** | | | **918.501** | **856.689** | **100.00** | **100.00** | |

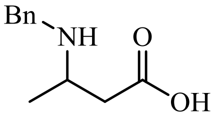


*rac*-**2a**

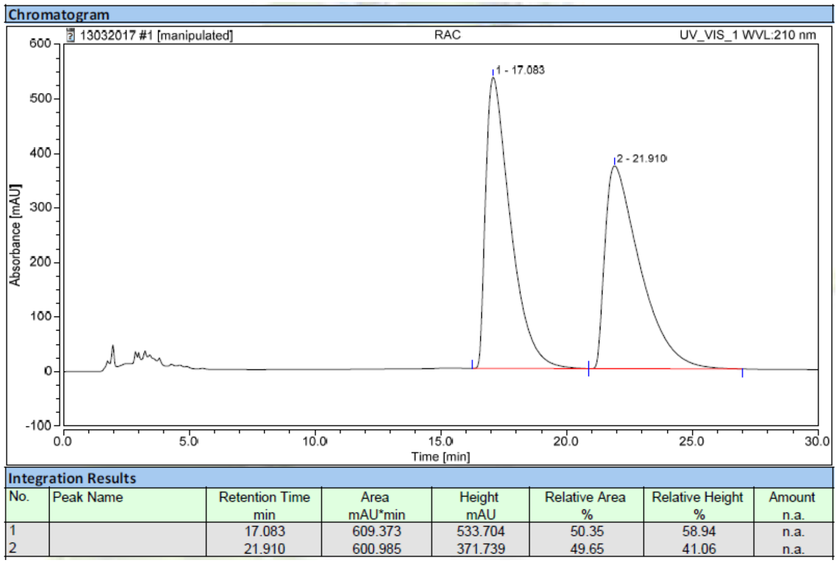


| Integration Results | | | | | | | |
|---|---|---|---|---|---|---|---|
| No. | Peak Name | Retention Time min | Area mAU*min | Height mAU | Relative Area % | Relative Height % | Amount n.a. |
| 1 | | 17.083 | 609.373 | 533.704 | 50.35 | 58.94 | n.a. |
| 2 | | 21.910 | 600.985 | 371.739 | 49.65 | 41.06 | n.a. |

**For entry 2.**

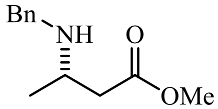


(*S*)-**1a**

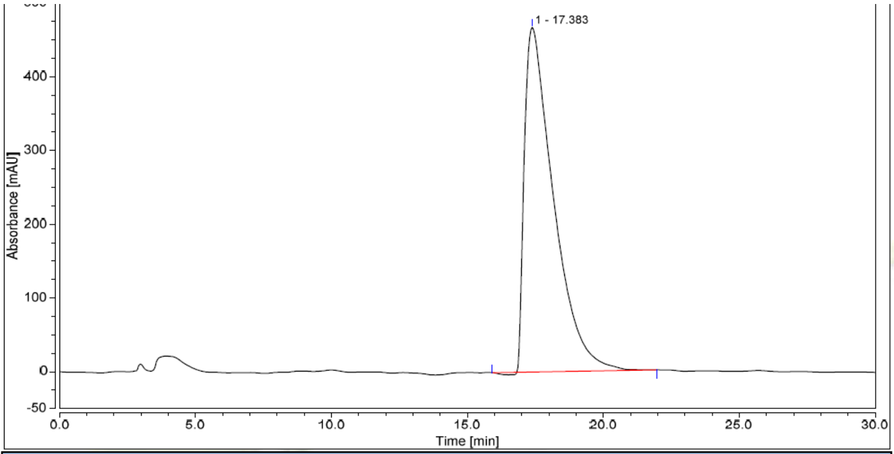


| Integration Results | | | | | | | |
|---|---|---|---|---|---|---|---|
| No. | Peak Name | Retention Time min | Area mAU*min | Height mAU | Relative Area % | Relative Height % | Amount n.a. |
| 1 | | 17.383 | 579.249 | 467.640 | 100.00 | 100.00 | n.a. |
| **Total:** | | | **579.249** | **467.640** | **100.00** | **100.00** | |

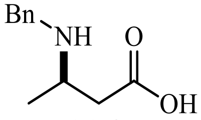


(*R*)-**2a**

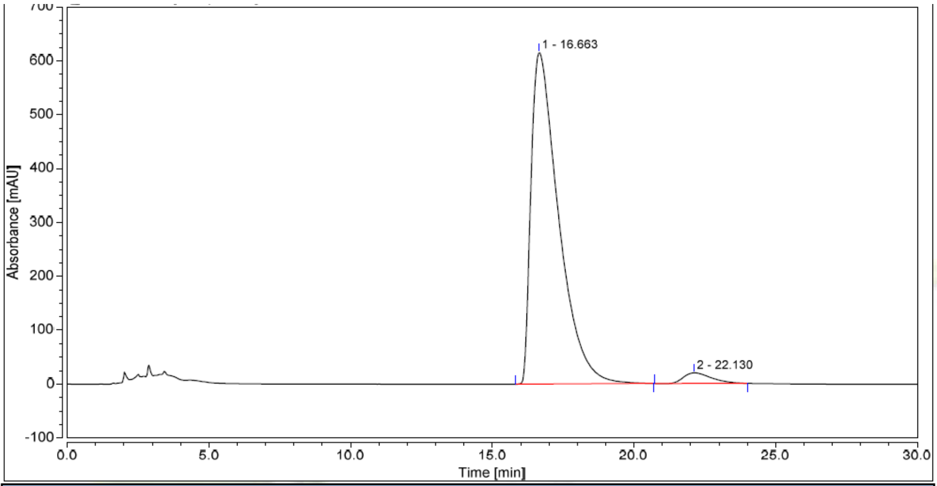


| Integration Results | | | | | | | |
|---|---|---|---|---|---|---|---|
| No. | Peak Name | Retention Time min | Area mAU*min | Height mAU | Relative Area % | Relative Height % | Amount n.a. |
| 1 | | 16.663 | 693.709 | 615.390 | 96.91 | 96.90 | n.a. |
| 2 | | 22.130 | 22.121 | 19.698 | 3.09 | 3.10 | n.a. |
| **Total:** | | | **715.830** | **635.088** | **100.00** | **100.00** | |

**For entry 3.**

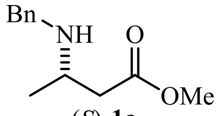


(*S*)-**1a**

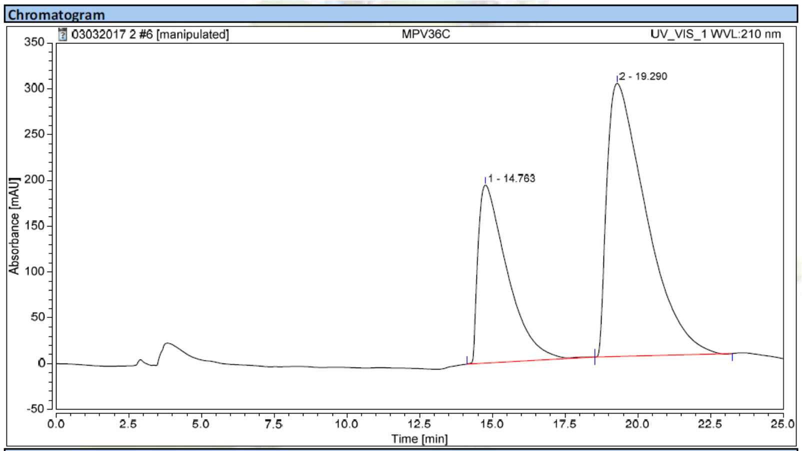


**Integration Results**

| No. | Peak Name | Retention Time min | Area mAU*min | Height mAU | Relative Area % | Relative Height % | Amount n.a. |
|---|---|---|---|---|---|---|---|
| 1 | | 14.763 | 226.069 | 194.317 | 32.61 | 39.46 | n.a. |
| 2 | | 19.290 | 467.236 | 298.136 | 67.39 | 60.54 | n.a. |
| **Total:** | | | **693.305** | **492.453** | **100.00** | **100.00** | |

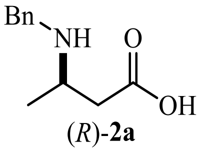


(*R*)-**2a**

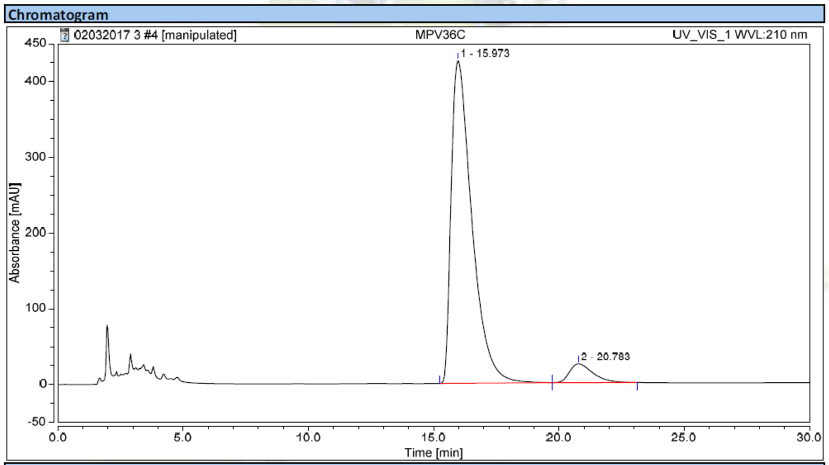


**Integration Results**

| No. | Peak Name | Retention Time min | Area mAU*min | Height mAU | Relative Area % | Relative Height % | Amount n.a. |
|---|---|---|---|---|---|---|---|
| 1 | | 15.973 | 407.857 | 426.512 | 93.83 | 94.50 | n.a. |
| 2 | | 20.783 | 26.832 | 24.844 | 6.17 | 5.50 | n.a. |
| **Total:** | | | **434.689** | **451.356** | **100.00** | **100.00** | |

**For entry 4.**

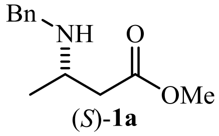

(*S*)-**1a**

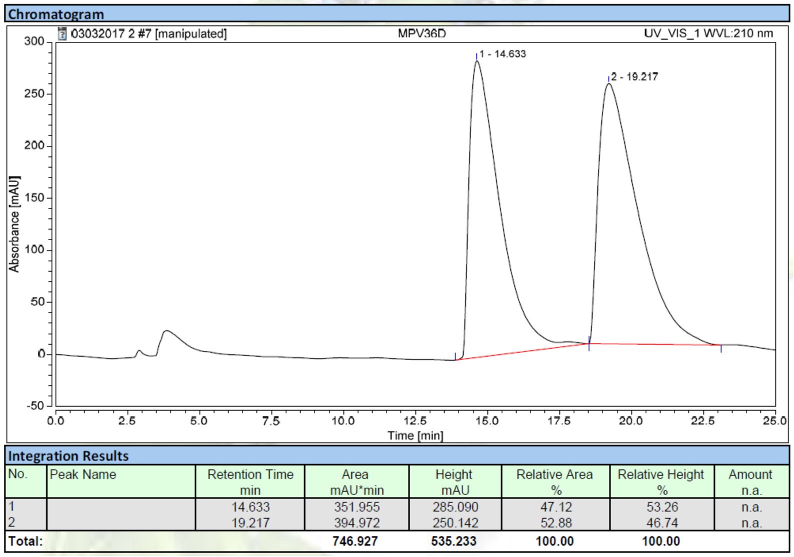


**Integration Results**

| No. | Peak Name | Retention Time min | Area mAU*min | Height mAU | Relative Area % | Relative Height % | Amount n.a. |
|---|---|---|---|---|---|---|---|
| 1 | | 14.633 | 351.955 | 285.090 | 47.12 | 53.26 | n.a. |
| 2 | | 19.217 | 394.972 | 250.142 | 52.88 | 46.74 | n.a. |
| **Total:** | | | **746.927** | **535.233** | **100.00** | **100.00** | |

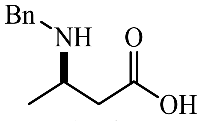

(*R*)-**2a**

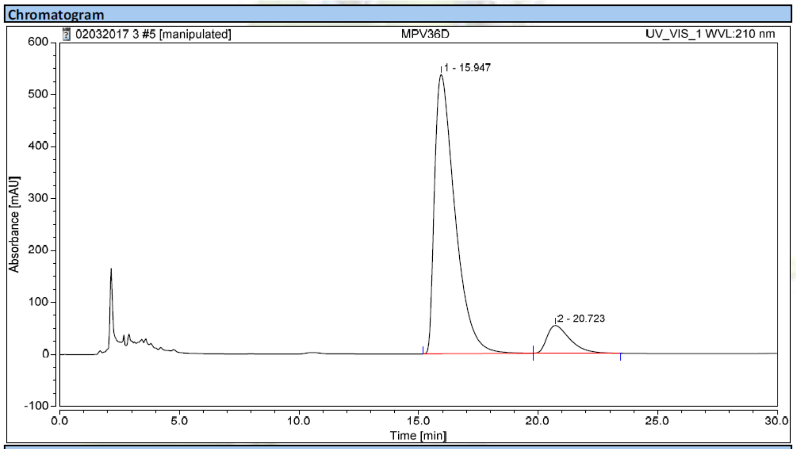


**Integration Results**

| No. | Peak Name | Retention Time min | Area mAU*min | Height mAU | Relative Area % | Relative Height % | Amount n.a. |
|---|---|---|---|---|---|---|---|
| 1 | | 15.947 | 532.103 | 537.873 | 89.94 | 90.97 | n.a. |
| 2 | | 20.723 | 59.531 | 53.416 | 10.06 | 9.03 | n.a. |
| **Total:** | | | **591.634** | **591.289** | **100.00** | **100.00** | |

**For entry 5.**

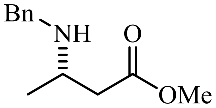


(*S*)-**1a**

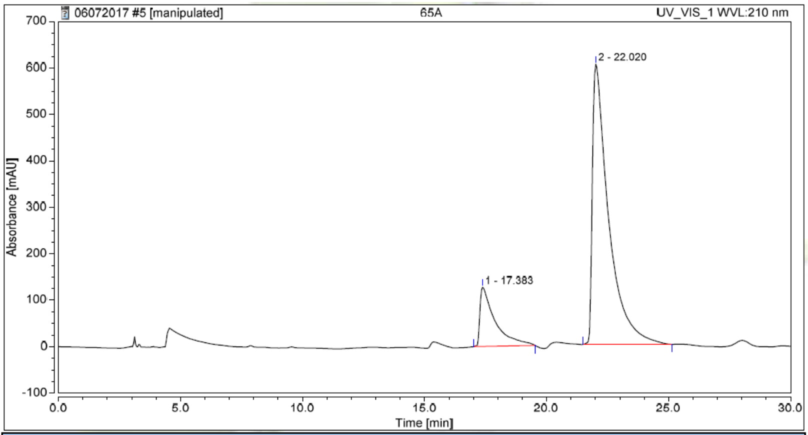


| Integration Results | | | | | | | |
|---|---|---|---|---|---|---|---|
| No. | Peak Name | Retention Time min | Area mAU*min | Height mAU | Relative Area % | Relative Height % | Amount n.a. |
| 1 | | 17.383 | 91.498 | 127.524 | 16.49 | 17.44 | n.a. |
| 2 | | 22.020 | 463.448 | 603.731 | 83.51 | 82.56 | n.a. |
| **Total:** | | | **554.946** | **731.255** | **100.00** | **100.00** | |

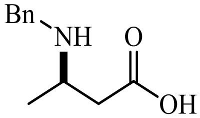


(*R*)-**2a**

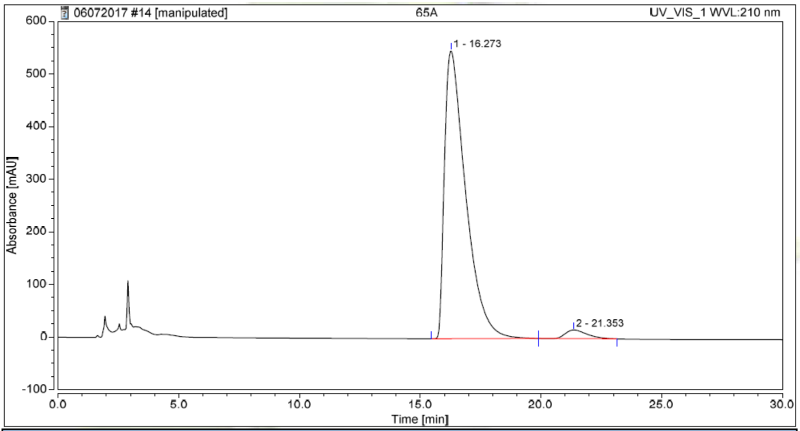


| Integration Results | | | | | | | |
|---|---|---|---|---|---|---|---|
| No. | Peak Name | Retention Time min | Area mAU*min | Height mAU | Relative Area % | Relative Height % | Amount n.a. |
| 1 | | 16.273 | 556.137 | 547.420 | 97.05 | 97.09 | n.a. |
| 2 | | 21.353 | 16.933 | 16.431 | 2.95 | 2.91 | n.a. |
| **Total:** | | | **573.070** | **563.851** | **100.00** | **100.00** | |

**For entry 6.**

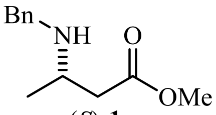

(*S*)-**1a**

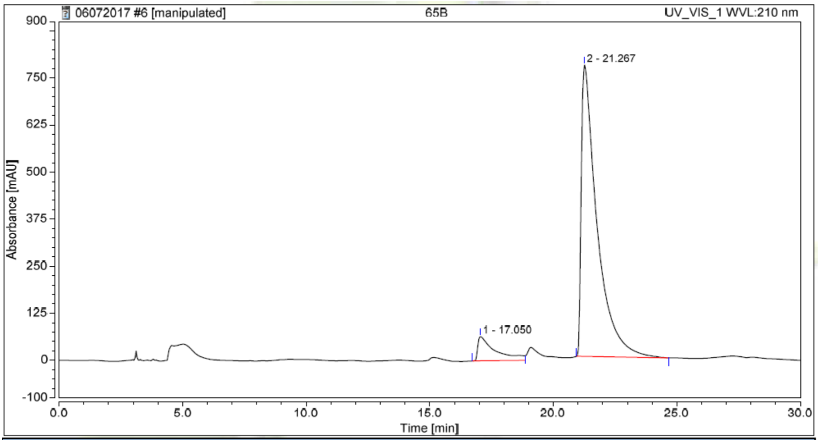


| Integration Results | | | | | | | |
|---|---|---|---|---|---|---|---|
| No. | Peak Name | Retention Time min | Area mAU*min | Height mAU | Relative Area % | Relative Height % | Amount n.a. |
| 1 | | 17.050 | 53.349 | 64.519 | 8.54 | 7.69 | n.a. |
| 2 | | 21.267 | 571.031 | 774.103 | 91.46 | 92.31 | n.a. |
| Total: | | | 624.381 | 838.622 | 100.00 | 100.00 | |

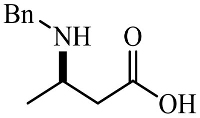

(*R*)-**2a**

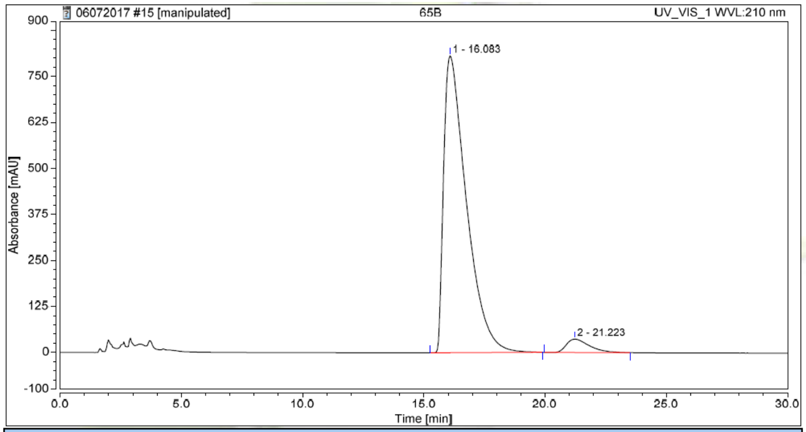


| Integration Results | | | | | | | |
|---|---|---|---|---|---|---|---|
| No. | Peak Name | Retention Time min | Area mAU*min | Height mAU | Relative Area % | Relative Height % | Amount n.a. |
| 1 | | 16.083 | 876.598 | 807.399 | 95.70 | 95.70 | n.a. |
| 2 | | 21.223 | 39.370 | 36.295 | 4.30 | 4.30 | n.a. |
| Total: | | | 915.968 | 843.694 | 100.00 | 100.00 | |

- **Chromatograms for scaling-up for the enzymatic hydrolysis using the substrate 1a (Table S4).**

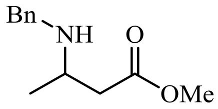


*rac*-**1a**

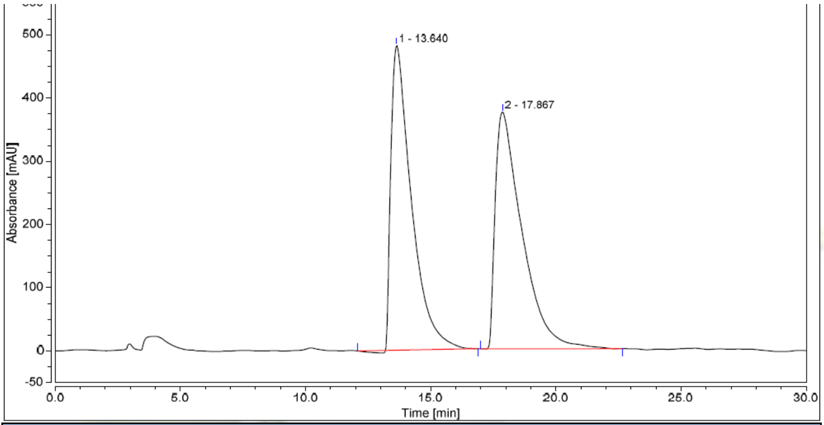


| Integration Results | | | | | | | |
|---|---|---|---|---|---|---|---|
| No. | Peak Name | Retention Time min | Area mAU*min | Height mAU | Relative Area % | Relative Height % | Amount n.a. |
| 1 | | 13.640 | 455.884 | 482.090 | 49.63 | 56.27 | n.a. |
| 2 | | 17.867 | 462.617 | 374.599 | 50.37 | 43.73 | n.a. |
| Total: | | | 918.501 | 856.689 | 100.00 | 100.00 | |

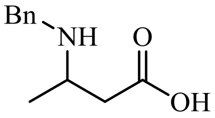


*rac*-**2a**

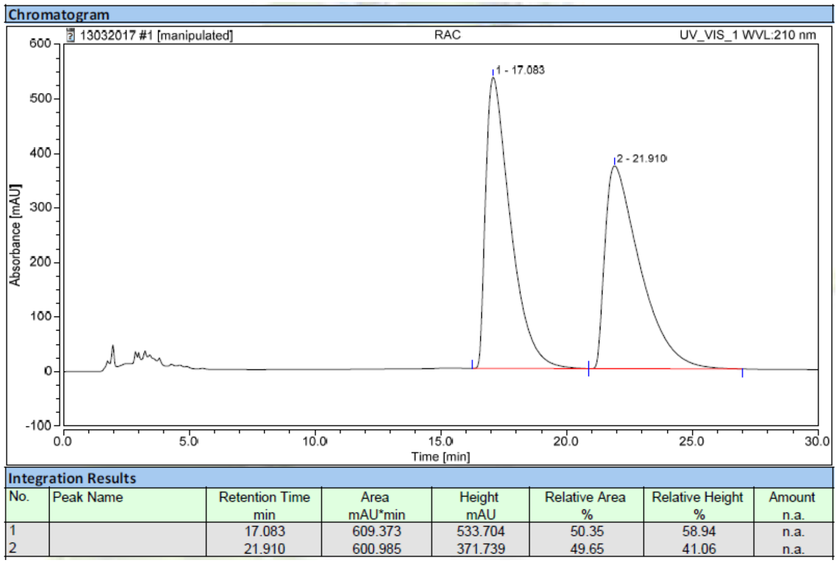


| Integration Results | | | | | | | |
|---|---|---|---|---|---|---|---|
| No. | Peak Name | Retention Time min | Area mAU*min | Height mAU | Relative Area % | Relative Height % | Amount n.a. |
| 1 | | 17.083 | 609.373 | 533.704 | 50.35 | 58.94 | n.a. |
| 2 | | 21.910 | 600.985 | 371.739 | 49.65 | 41.06 | n.a. |

**For entry 1.**

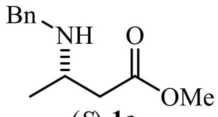


(*S*)-**1a**

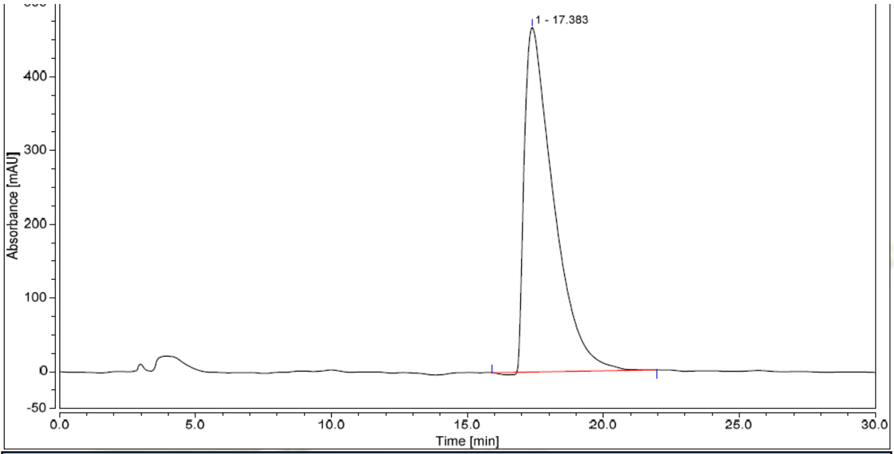


| Integration Results | | | | | | | |
|---|---|---|---|---|---|---|---|
| No. | Peak Name | Retention Time min | Area mAU*min | Height mAU | Relative Area % | Relative Height % | Amount n.a. |
| 1 | | 17.383 | 579.249 | 467.640 | 100.00 | 100.00 | n.a. |
| **Total:** | | | **579.249** | **467.640** | **100.00** | **100.00** | |

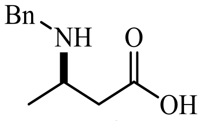


(*R*)-**2a**

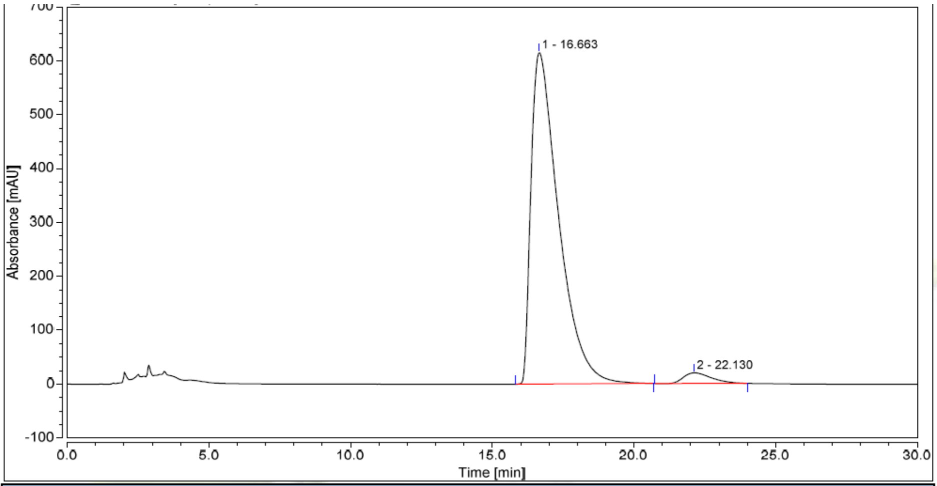


| Integration Results | | | | | | | |
|---|---|---|---|---|---|---|---|
| No. | Peak Name | Retention Time min | Area mAU*min | Height mAU | Relative Area % | Relative Height % | Amount n.a. |
| 1 | | 16.663 | 693.709 | 615.390 | 96.91 | 96.90 | n.a. |
| 2 | | 22.130 | 22.121 | 19.698 | 3.09 | 3.10 | n.a. |
| **Total:** | | | **715.830** | **635.088** | **100.00** | **100.00** | |

**For entry 2.**

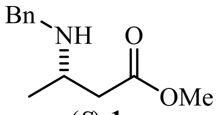


(*S*)-**1a**

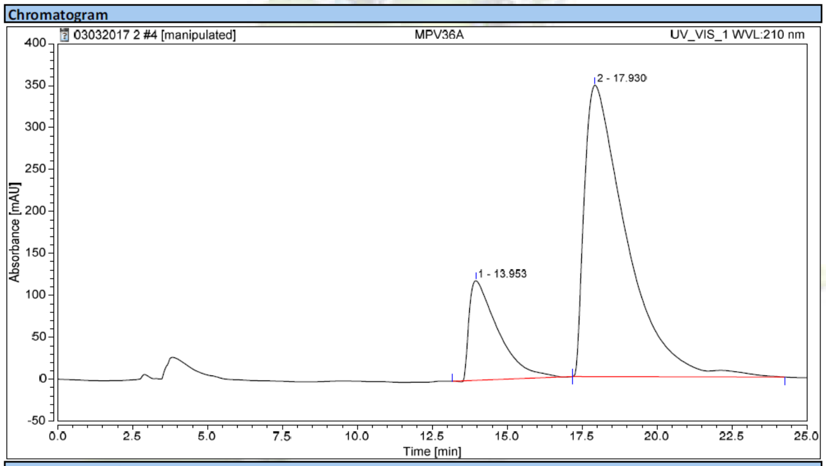


**Integration Results**

| No. | Peak Name | Retention Time<br>min | Area<br>mAU*min | Height<br>mAU | Relative Area<br>% | Relative Height<br>% | Amount<br>n.a. |
|---|---|---|---|---|---|---|---|
| 1 | | 13.953 | 132.279 | 118.717 | 18.94 | 25.46 | n.a. |
| 2 | | 17.930 | 565.995 | 347.548 | 81.06 | 74.54 | n.a. |
| **Total:** | | | **698.273** | **466.264** | **100.00** | **100.00** | |

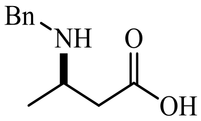


(*R*)-**2a**

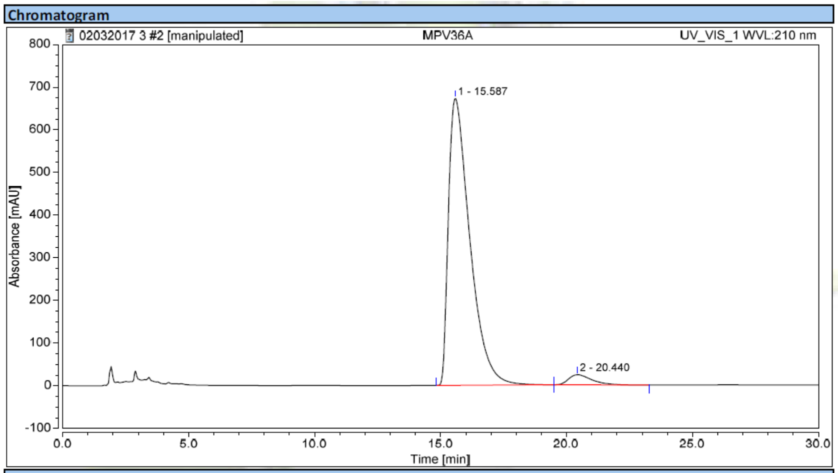


**Integration Results**

| No. | Peak Name | Retention Time<br>min | Area<br>mAU*min | Height<br>mAU | Relative Area<br>% | Relative Height<br>% | Amount<br>n.a. |
|---|---|---|---|---|---|---|---|
| 1 | | 15.587 | 661.362 | 673.225 | 96.35 | 96.62 | n.a. |
| 2 | | 20.440 | 25.060 | 23.541 | 3.65 | 3.38 | n.a. |
| **Total:** | | | **686.423** | **696.767** | **100.00** | **100.00** | |

**For entry 3.**

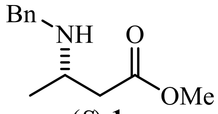


(*S*)-**1a**

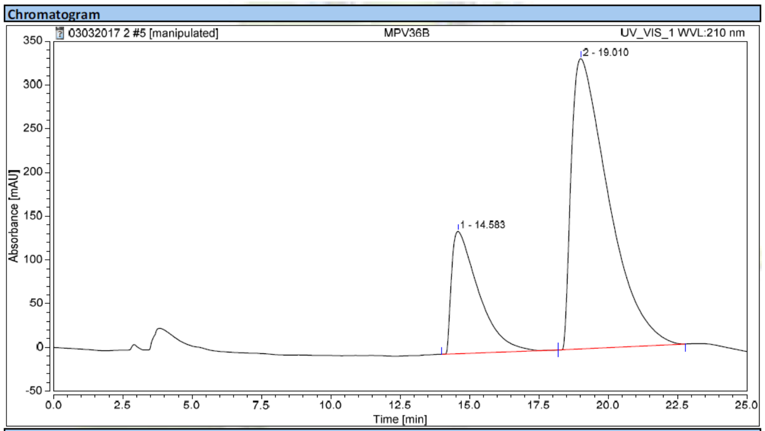


Integration Results

| No. | Peak Name | Retention Time min | Area mAU*min | Height mAU | Relative Area % | Relative Height % | Amount n.a. |
|---|---|---|---|---|---|---|---|
| 1 | | 14.583 | 158.096 | 139.861 | 23.40 | 29.65 | n.a. |
| 2 | | 19.010 | 517.470 | 331.834 | 76.60 | 70.35 | n.a. |
| Total: | | | 675.566 | 471.695 | 100.00 | 100.00 | |

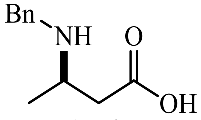


(*R*)-**2a**

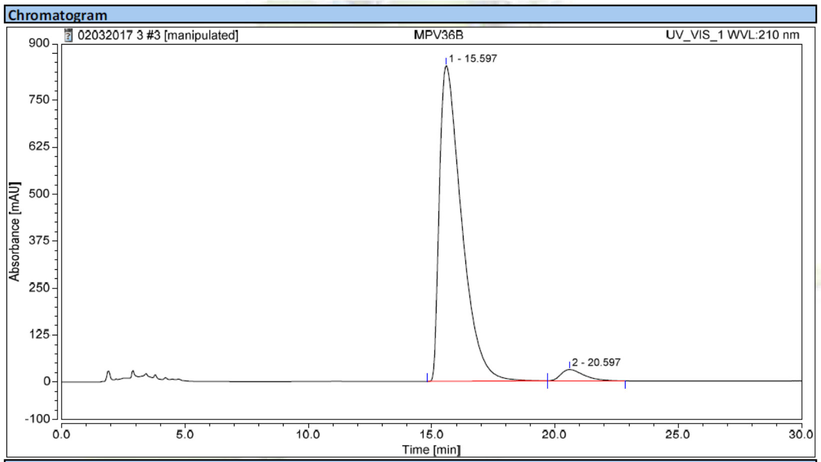


Integration Results

| No. | Peak Name | Retention Time min | Area mAU*min | Height mAU | Relative Area % | Relative Height % | Amount n.a. |
|---|---|---|---|---|---|---|---|
| 1 | | 15.597 | 873.351 | 840.736 | 96.46 | 96.55 | n.a. |
| 2 | | 20.597 | 32.079 | 30.054 | 3.54 | 3.45 | n.a. |
| Total: | | | 905.430 | 870.790 | 100.00 | 100.00 | |

**For entry 4.**

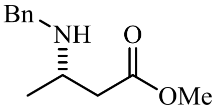


($S$)-**1a**

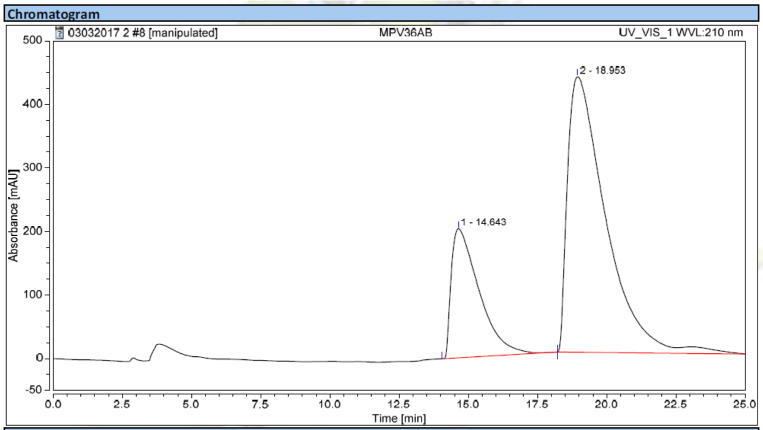


Integration Results

| No. | Peak Name | Retention Time min | Area mAU*min | Height mAU | Relative Area % | Relative Height % | Amount n.a. |
|---|---|---|---|---|---|---|---|
| 1 | | 14.643 | 236.489 | 203.292 | 25.35 | 31.91 | n.a. |
| 2 | | 18.953 | 696.572 | 433.696 | 74.65 | 68.09 | n.a. |
| **Total:** | | | **933.061** | **636.988** | **100.00** | **100.00** | |

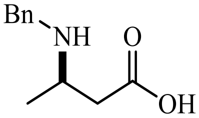


($R$)-**2a**

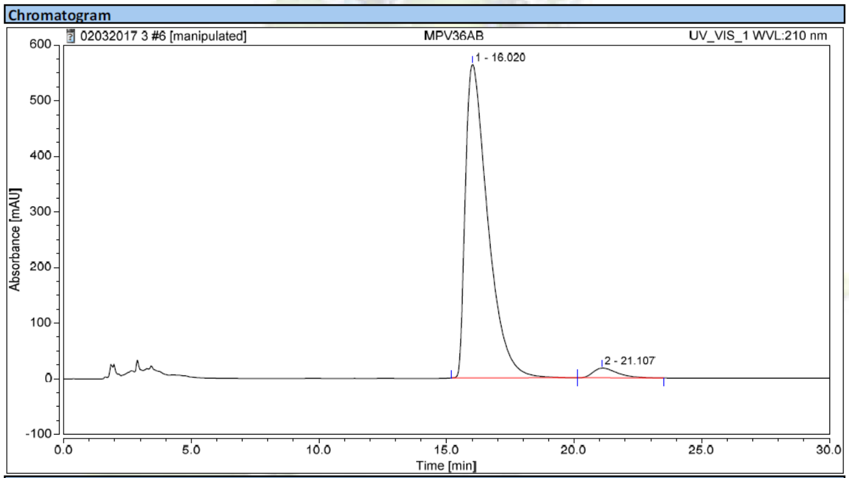


Integration Results

| No. | Peak Name | Retention Time min | Area mAU*min | Height mAU | Relative Area % | Relative Height % | Amount n.a. |
|---|---|---|---|---|---|---|---|
| 1 | | 16.020 | 572.809 | 563.838 | 96.73 | 96.97 | n.a. |
| 2 | | 21.107 | 19.386 | 17.592 | 3.27 | 3.03 | n.a. |
| **Total:** | | | **592.195** | **581.431** | **100.00** | **100.00** | |

## 7) HPLC conditions and retention times for the substrates *rac*-1a to *rac*-1j and hydrolysis products 2a to 2j.

| | Substrates<br>Conditions<br>Column, Eluent, Flow(mL/min), Time(min) | | Products<br>Conditions<br>Column, Eluent, Flow(mL/min), Time(min) |
|---|---|---|---|
| 1a | Chiralpack **OD**, Hex/IPA (99:1), 1, 30 | 2a | Chirobiotic **TAG**, MeOH, 1, 30 |
| 1b | Chiralpack **OD-H**, Hex/IPA (98:2), 1, 30 | 2b | Chirobiotic **T**, EtOH/$H_2O$ (70:30), 0.5, 30 |
| 1c | Chiralpack **OD-H**, Hex/IPA (99:1), 0.6, 30 | 2c | Chirobiotic **T**, EtOH/$H_2O$ (70:30), 0.5, 30 |
| 1d | Chiralpack **AD-H**, Hex/IPA (99:1), 0.6, 30 | 2d | Chirobiotic **T**, EtOH/$H_2O$ (70:30), 0.5, 30 |
| 1e | Chiralpack **AD-H**, Hex/IPA (99:1), 0.6, 30 | 2e | Chirobiotic **T**, EtOH/$H_2O$ (70:30), 0.5, 30 |
| 1f | Chiralpack **AD-H**, Hex/IPA (99:1), 0.6, 30 | 2f | Chirobiotic **T**, EtOH/$H_2O$ (70:30), 0.5, 30 |
| 1g | Chiralpack **OD-H**, Hex/IPA (95:5), 1, 30 | 2g | Chirobiotic **T**, MeOH/$H_2O$ (95:5), 0.8, 20 |
| 1h | Chiralpack **OD**, Hex/IPA (99:1), 0.9, 45 | 2h | Chirobiotic **TAG**, MeOH, 0.8, 30 |
| 1i | Chiralpack **AD-H**, Hex/IPA (99:1), 1, 15 | 2i | Chirobiotic **T**, EtOH/$H_2O$ (70:30), 0.5, 40 |

| Substrates | | | Products | | |
|---|---|---|---|---|---|
| Retention time (min) | | | Retention time (min) | | |
| | $t_R$ | $t_S$ | | $t_R$ | $t_S$ |
| 1a | 13.64 | 17.86 | 2a | 17.08 | 21.91 |
| 1b | 8.70 | 11.51 | 2b | 17.14 | 20.50 |
| 1c | 22.76 | 25.50 | 2c | 16.31 | 19.50 |
| 1d | 15.13 | 18.50 | 2d | 14.93 | 17.58 |
| 1e | 20.65 | 24.87 | 2e | 14.03 | 16.96 |
| 1f | 14.41 | 17.70 | 2f | 13.21 | 15.69 |
| 1g | 13.09 | 7.68 | 2g | 10.97 | 10.05 |
| 1h | 24.58 | 32.58 | 2h | 7.96 | 7.13 |
| 1i | 4.93 | 7.03 | 2i | 17.46 | 29.07 |

## 8) Determinations of the absolute configuration for the products 2a to 2j.

For the determination of the absolute configuration of all products we used different methodologies. A crystallographic report for product (*R*)-**2a** was obtained and the ORTEP diagram are show below, as well, a CIF report is attached to this archive, the absolute configuration for this product was determined as *R,* furthermore, the specific optical rotation was compared with the reported by Escalante [4] (Table S2, entry 1).

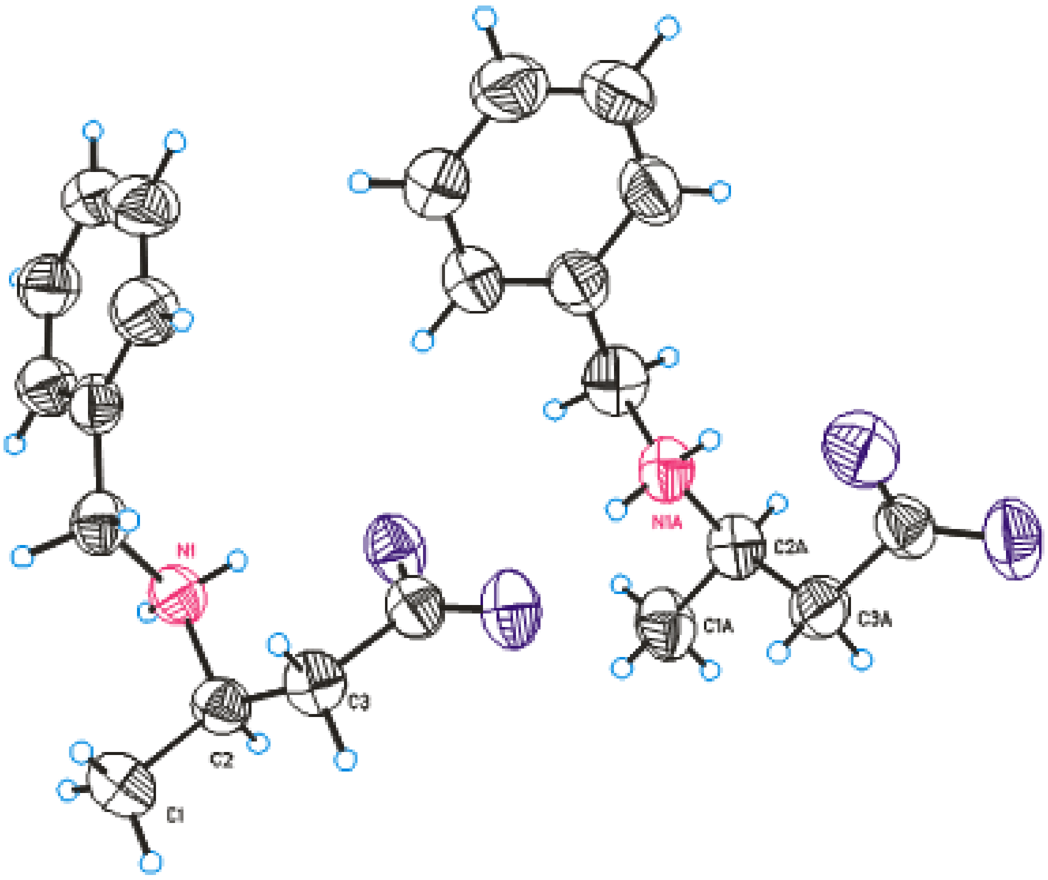


**Figure S50. ORTEP diagram for product (*R*)-2a.**

The absolute configuration of product **2b** was assigned by chemical comparison with the reported data [17], **reference** (*S*)-**2b** $[\alpha]_D^{25}$ = +32.4 and ee > 99%**, product** (*R*)-**2b** $[\alpha]_D^{25}$ = -36.5 and ee = 97%**,** obtaining an *R* configuration. The products **2c** to **2f** were assigned followed an elution sequence general rule already describe [18] using the same column that we used (Chirobiotic T). With the certainty that product **2b** has a well know absolute configuration and that the elution sequence for the majority product is the same for product **2b** to **2f** we assigned the same configuration for all them (*R*).

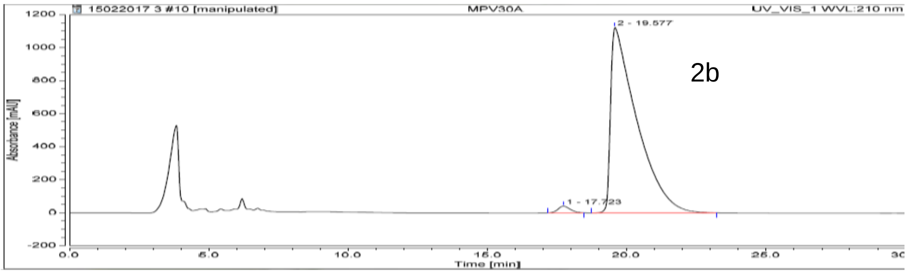


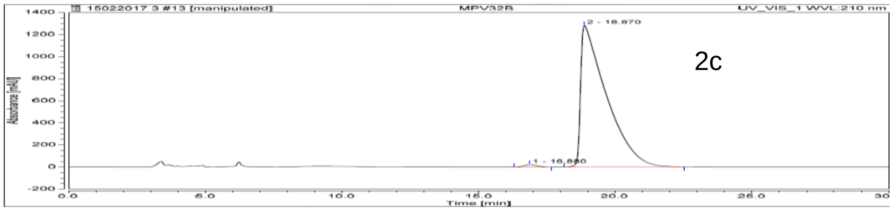


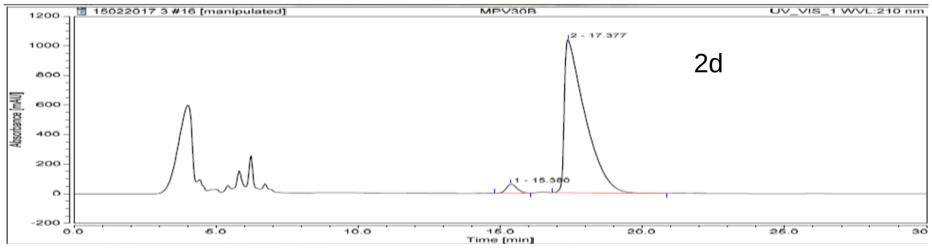


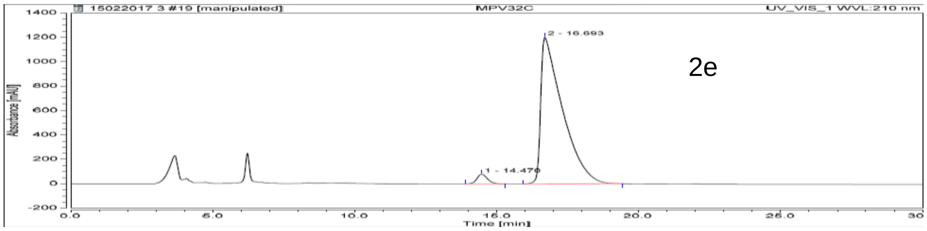

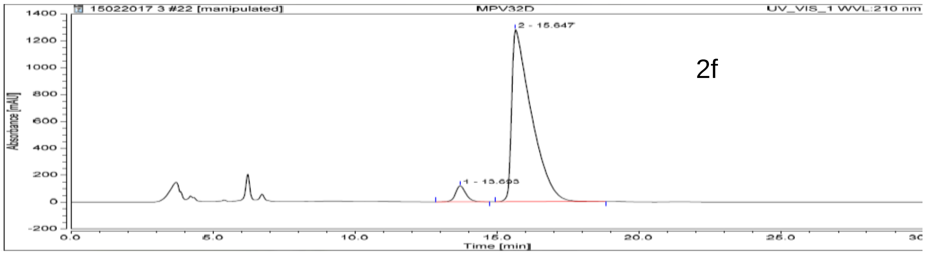


The absolute configuration for products **2g** and **2i** was assigned by chemical comparison [4], **reference** (*S*)-**2g**[4] $[\alpha]_D^{20}$ = -51.9 and ee > 99%**, product** (*S*)-**2g** $[\alpha]_D^{25}$ = -35.0 and ee = 83%**, reference** (*S*)-**2i**[4] $[\alpha]_D^{20}$ = +26 and ee > 99%**, product** (*S*)-**2i** $[\alpha]_D^{25}$ = +12.8 and ee = 94%**,** and for product **2h** an elution sequence general rule was used, compare product **2a** and **2h**, obtaining the *S* configuration for product **2h**.

## References

[1] Andrews, I. P.; Know, O. *Organic Synthesis*. **2012**, *12*, 586.

[2] Shearouse, W. C.; Korte, C. M.; Mack, J. *Green Chem*. **2011**, *13*, 598.

[3] Börner, C.; Dennis, M. R.; Sinn, E.; Woodward, S. *Eur. J. Org. Chem*. **2001**, 2435.

[4] Rangel, H.; Morales, M. C.; Galindo, J. M.; Castillo, E.; Zúñiga, A. O.; Juaristi, E.; Escalante, J. *Tetrahedron: Asymmetry*. **2015**, *26*, 325.

[5] Kandula, S. R.; Kumar, P. *Tetrahedron Asymmetry*, **2005**, *16*, 3268-3274.

[6] Alcock, S. G.; Baldwin, J. E.; Bohlmann, R.; Harwood, L. M.; Seeman, J. I. *J. Org. Chem*., **1985**, *50*, 3526-3535.

[7] O´Brien, C. J.; Tellez, J. L.; Nixon, Z. S.; Kang, L. J.; Carter, A. L.; Kunkel, S. R.; Przeworski, K. C.; Chass, G. A. *Angew. Chem. Int. Ed*., **2009**, *48*, 6836-6839.

[8] Malla, R. K.; Ridenour, J. N.; Spilling, C. D. *Beilstein J. Org. Chem*., **2014**, *10*, 1933-1941.

[9] Choudary, B. M.; Mahendar, K.; Kantam, M. L.; Ranganath, K. V. S.; Athar, T. *Adv. Synth. Catal*., **2006**, *348*, 1977-1985.

[10] Asao, N.; Uyehara, T.; Tamamoto, Y. *Tetrahedron,* **1988**, *44*, 4173-4180.

[11] Guizzetti, S.; Benaglia, M.; Bonsignore, M.; Raimondi, L. *Org. Biomol. Chem*., **2011**, *9*, 739-743.

[12] Wang, W.-B.; Roskamp, E. J. *J. Am. Chem. Soc*., **1993**, *115*, 9417, 9420.

[13] Bonsignore, M.; Benaglia, M.; Annunziata, R.; Celentano, G. *SYNLETT*, **2011**, *8*, 1085-1088.

[14] Gedey, S.; Liljeblad, A.; Lázár, L.; Fülöp, F.; Kanerva, L. T. *Tetrahedron: Asymmetry*, **2001**, *12*, 105-110.

[15] Bach, K. K.; El-Seedi, H. R.; Jensen, H. M.; Nielsen, H. B.; Thomsen, I.; Torssell, K. B. G. *Tetrahedron*, **1994**, *50*, 7543-7556.

[16] Blicke, F. F.; Gould, W. A. *J. Org. Chem*. **1985**, *23*, 1102.

[17] Ma, D-Y.; Wang, D.-X.; Pan, J.; Huang, Z-T.; Wang, M.-X. *J. Org. Chem*. **2008**, *73*, 4087.

[18] Péter, A.; Lázár, L.; Fülöp, F.; Armstrong, D.W. *J. Chromatogr. A*. **2001**, *926*, 229.
