# Supplementary material for: Correction: Mechanochemical enzymatic resolution of N-benzylated-β3-amino esters
Source: Beilstein J Org Chem. 2017 Oct 12;13:2128–30. doi: 10.3762/bjoc.13.210 (PMC5647731; doi:10.3762/bjoc.13.210)
Supplement: File 1 — Experimental section, NMR spectra, chromatograms and X-ray diffraction data. [file Beilstein_J_Org_Chem-13-2128-s001.pdf]

Supporting Information

for

Correction: Mechanochemical enzymatic resolution of *N*-benzylated-β^3-amino esters

Mario Pérez-Venegas[1], Gloria Reyes-Rangel[1], Adrián Neri[2], Jaime Escalante*[,2] and Eusebio Juaristi.*[,1,3]

Address: [1]Departamento de Química, Centro de Investigación y de Estudios Avanzados, Avenida I.P.N. 2508, Ciudad de México, 07360, Mexico, [2]Centro de Investigaciones Químicas, Universidad Autónoma del Estado de Morelos, Av. Universidad 1001, Cuernavaca, Morelos, 62210, Mexico and [3]El Colegio Nacional, Luis Gonzáles Obregón 23, Centro Histórico, Ciudad de México, 06020, Mexico

Email: Jaime Escalante* - jaime@uaem.mx; Eusebio Juaristi* - juaristi@relaq.mx

*Corresponding author

Experimental section, NMR spectra, chromatograms and X-ray diffraction data

Table of Contents

1) **General methods** **S2**

2) **General method for the synthesis of *N*-benzylated-β^3-amino esters** **S2**

3) **General method for the synthesis of *N*-benzylated-β^3-amino acid** **S9**

4) **General method for the enzymatic resolution using CALB** **S13**

5) **^{1}H and ^{13}C spectra for M*t*E-b to M*t*E-d, *rac*-1a to *rac*-1j and products 2a to 2j** **S18**

6) **Chromatograms for the determination of enantiomeric excess of raw material and hydrolysis products** **S43**

7) **HPLC conditions and retention times for the substrates *rac*-1a to *rac*-1j and hydrolysis products *rac*-2a to *rac*-2j** **S94**

8) **Determinations of the absolute configuration for the products 2a to 2j.** **S95**

9) **References** **S98**

1) General methods

NMR spectra were recorded on a BRUKER DP300 (300 MHz) and a Jeol Eclipse (400 MHz). High resolution mass spectra were recorded on a HPLC 1100 coupled to a MSD-TOF Agilent series HR-MSTOF model 1969 A. Chromatograms were acquired in a Dionex HPLC Ultimate 3000 with a UV/Visible detector, with diode array, at 210 and 254 nm. HSBM reactions were carry out in a Retsch, Mixer Mill (MM200). *N*-benzylated-β^3-amino esters were synthetized according to the literature. All reagents for the synthesis were purchased from Sigma-Aldrich. Immobilized CALB was purchased from Novozymes, Novozym 453 (Immobilized on acrylic resine, U/g >100000)

2) General method for the synthesis of *N*-benzylated-β^3-amino esters.

The substrates *rac*-**1a** to *rac*-**1f** (Table 2 in article) for the enzymatic resolution were synthetized according to previously reported methodologies [1-3], the synthesis for *rac*-**1a** starts from Methyl crotonate. For substrates *rac*-**1g to** *rac*-**1i** we used the methodology describe by Escalante [4].

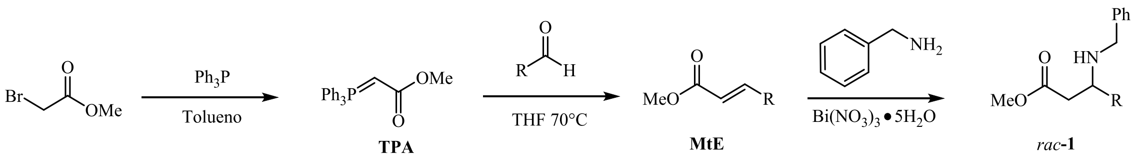

Scheme 1. Route for the synthesis of substrates rac-1.

Synthesis of Methyl (triphenylphosphoranyldiene) acetate (**TPA**): Triphenylphosphine (7.86 g, 0.03 mmol) was placed in a round flask equipped with a magnetic bar and was dissolved in Toluene (100 mL), methyl bromoacetate (5.01 g, 0.03 mmol) was added and the reaction was stirred overnight. The reaction product was filtered and the resulting solid was dissolved in water (200 mL), basified till pH 9 using KOH (2M) and phenolphthalein as indicator and was extracted with CH_2Cl_2 (150 mL x 2), the organic fractions were collect, dried with anhydride

sodium sulfate and concentrate to obtain 9.3 g (93% yield) of a white solid (**TPA**). MS-TOF: calculated for $C_{21}H_{20}0_2P$ 335.1156, found 335.1197. ^{1}H NMR ($CDCl_3$, 300MHz): δ= 7.53 (m, 15H), 3.51 (s, 3H). ^{13}C{^{1}H} NMR ($CDCl_3$, 300MHz): δ 28.7, 30.5, 50.0, 128.8, 128.9, 132.1, 133.0, 133.1. ^{31}P{^{1}H} NMR ($CDCl_3$, 300MHz): δ 18.9, 23.1, 30.3.

Representative example for the synthesis of Methyl *trans*-2 pentenoate (**M*t*E-b**): In a round flask equipped with a magnetic bar **TPA** (10.02 g 0.03 mmol) was dissolved in anhydride THF (150 mL), propionaldehyde (1.32 g, 0.03 mmol) was added to the solution and the reaction mixture was refluxed for 4 hrs. The reaction was filtered and evaporated for his purification. The product was purified by flash chromatography on silica gel (Haxanes/Ethyl acetate) to give 5.2 g of a colorless liquid (**M(*E*)E-b**) (86% yield).

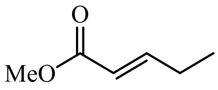

- Methyl *trans*-2 pentenoate (**M*t*E-b**) [5], from propionaldehyde, colorless liquid, 86% yield. ^{1}H NMR ($CDCl_3$, 300MHz): δ 7.01 (dt, J=6.3 Hz, J_{trans}=15.6 Hz, 1H), 5.80 (d, J=15.6 Hz, 1H), 3.71 (s, 3H), 2.21 (quint, J=6.7 Hz, 2H), 1.05 (t, J=7.2 Hz, 3H), 5.28 (CH_2Cl_2). ^{13}C{^{1}H} NMR ($CDCl_3$, 300MHz): δ 12.3, 25.5, 51.5, 120.1, 151.2, 167.4.

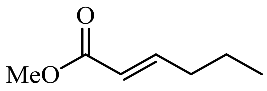

- Methyl *trans*-2 hexenoate (**M*t*E-c**) [6], from butyraldehyde, colorless liquid, 70% yield. ^{1}H NMR ($CDCl_3$, 300MHz): δ 6.96 (dt, J=6.9 Hz, J_{trans}=15.6 Hz, 1H), 5.81 (d, J=15.6 Hz, 1H), 3.71 (s, 3H), 2.21 (q, J=6.8 Hz, 2H), 1.47 (m, J=7.5 Hz, 2H), 0.92 (t, J=7.3 Hz, 3H), 5.29 (CH_2Cl_2). ^{13}C{^{1}H} NMR ($CDCl_3$, 300MHz): δ 13.7, 21.4, 34.6, 51.4, 121.1, 149.8, 167.6.

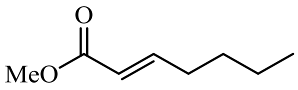

- Methyl *trans*-2 heptenoate (**M*t*E-d**) [7], from valeraldehyde, colorless liquid, 83% yield. ^{1}H NMR ($CDCl_3$, 300MHz): δ 6.95 (dt, *J*=6.9 Hz, J_{trans}=15.6 Hz, 1H), 5.79 (d, *J*=15.6 Hz, 1H), 3.69 (s, 3H), 2.17 (q, *J*=6.9 Hz, 2H), 1.35 (m, 2H), 0.88 (t, *J*=7.2 Hz, 3H). ^{13}C{^{1}H} NMR ($CDCl_3$, 300MHz): δ 13.7, 22.4, 30.2, 32.3, 51.6, 120.9, 149.7, 167.2.

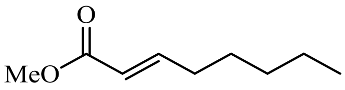

- Methyl *trans*-2 octenoate (**M*t*E-e**) [8], from hexanal, colorless liquid, 80% yield. ^{1}H NMR ($CDCl_3$, 300MHz): δ 6.82 (dt, *J*=7.2 Hz, J_{trans}=15.6 Hz, 1H), 5.80 (d, *J*=15.6 Hz, 1H), 3.71 (s, 3H), 2.18 (q, *J*=7.2 Hz, 2H), 1.44 (quint, *J*=16.5 Hz, 2H), 1.29 (m, 4H), 0.87 (t, *J*=6.3 Hz, 3H). ^{13}C{^{1}H} NMR ($CDCl_3$, 300MHz): δ 41.1, 22.6, 27.8, 31.4, 32.2, 120.8, 150.0, 167.4.

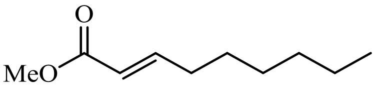

- Methyl *trans*-2 nonenoate (**M*t*E-f**) [9], from heptanal, colorless liquid, 75% yield. ^{1}H NMR ($CDCl_3$, 300MHz): δ 6.96 (dt, *J*=6.9 Hz, J_{trans}=15.6 Hz, 1H), 5.85 (d, *J*=15.9 Hz, 1H), 3.71 (s, 3H), 2.18 (q, *J*=7.2 Hz, 2H), 1.43 (quint, *J*=5.1 Hz, 2H), 1.27 (m, 4H), 0.87 (t, *J*=6.6 Hz, 3H). ^{13}C{^{1}H} NMR ($CDCl_3$, 300MHz): δ 14.2, 22.7, 28.1, 29.0, 31.7, 32.4, 51.6, 120.9, 150.1, 167.5.

Representative example for the synthesis of Methyl 3-(benzylamino) butanoate (*rac*-**1a**): $Bi(NO_3)_3 \cdot 5H_2O$ (0.8 g) was added to a round flask containing Methyl crotonate (3.18 mL, 0.03 mmol) equipped with a stir bar, the reaction was stirred by 5 min at 0 °C, after that benzylamine (3.27 mL, 0.03 mmol) was added. The reaction was stirred overnight, filtered, extracted with CH_2Cl_2, dried with anhydride sodium sulfate and concentrated for his purification, obtaining 4 g (63% yield) of an amber liquid, *rac*-**1a**. Compounds *rac*-**1g** to *rac*-**1i** were synthetized as describe in literature [4].

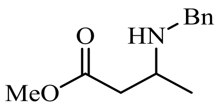

- *rac*-Methyl 3-(benzylamino) butanoate (*rac*-**1a**) [10], from methyl crotonate, amber liquid, 63% yield. MS-TOF: calculated for $C_{12}H_{18}N0_2$ 208.1293, found 208.1331. ^1H NMR ($CDCl_3$, 300MHz): δ 7.32 (d, *J*=4.2 Hz, 4H), 7.26 (m, 1H), 3.79 (q, *J*=12.3 Hz, 2H), 3.67 (s, 3H), 3.16 (m, *J*=6.3 Hz, 1H), 2.51 (dd, *J*= 6.9, 15.3, 1H), 2.39 (dd, *J*= 6, 15, 1H), 1.83 (br s, 1H), 1.43 (d, *J*=6.3 Hz, 3H). $^{13}C\{^1H\}$ NMR ($CDCl_3$, 300MHz): δ 20.58, 41.5, 49.8, 51.3, 51.7, 127.1, 128.3, 128.6, 140.4, 173.0.

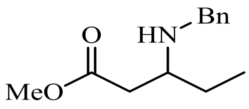

- *rac*-Methyl 3-(benzylamino) pentanoate (*rac*-**1b**) [11], from **M*t*E-b**, amber liquid, 67% yield. MS-TOF: calculated for $C_{13}H_{20}N0_2$ 222.1449, found 222.1489. ^1H NMR ($CDCl_3$, 300MHz): δ 7.32 (m, 5H), 3.78 (s, 2H), 3.67 (s, 3H), 2.97 (quint, J= 6 Hz, 1H), 2.46 (m, 2H), 1.53 (m, 2H) + (br, 1H), 0.92 (t, *J*= 7.2 Hz, 3H). $^{13}C\{^1H\}$ NMR ($CDCl_3$, 300MHz): δ 10.0, 26.9, 38.8, 51.0, 51.6, 55.6, 127.0, 128.3, 128.5, 140.7, 173.2.

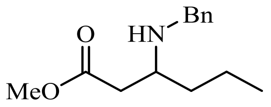

- *rac*-Methyl 3-(benzylamino) hexanoate (*rac*-**1c**) [12], from **M*t*E-c**, amber liquid, 57% yield. MS-TOF: calculated for $C_{14}H_{22}N0_2$ 236.1606, found 236.1647. ^{1}H NMR ($CDCl_3$, 300MHz): δ 7.27 (m, 5H), 3.78 (s, 2H), 3.67 (s, 3H), 3.03 (quint, *J*= 6.3 Hz, 1H), 2.46 (d, *J*= 6.3 Hz, 2H), 1.43 (m, 4H) + (br, 1H), 0.91 (t, *J*= 6.9 Hz, 3H). $^{13}C\{^1H\}$ NMR ($CDCl_3$, 300MHz): δ 14.3, 19.1, 36.7, 39.2, 51.1, 51.6, 54.1, 127.0, 128.2, 128.5, 140.7, 173.2.

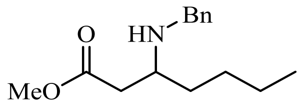

- *rac*-Methyl 3-(benzylamino) heptanoate (*rac*-**1d**), from **M*t*E-d**, amber liquid, 79% yield. MS-TOF: calculated for $C_{15}H_{24}N0_2$ 250.1762, found 250.1803. ^{1}H NMR ($CDCl_3$, 300MHz): δ 7.28 (m, 5H), 3.78 (s, 2H), 3.67 (s, 3H), 3.01 (quint, *J*= 6.3 Hz, 1H), 2.46 (d, *J*= 6.0 Hz, 2H), 1.42 (m, 6H) + (br, 1H), 0.89 (t, *J*= 6.6 Hz, 3H). $^{13}C\{^1H\}$ NMR ($CDCl_3$, 300MHz): δ 14.2, 22.9, 28.0, 34.2, 39.2, 51.1, 51.7, 54.4, 127.0, 128.3, 128.5, 140.7, 173.2.

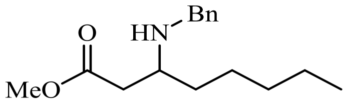

- *rac*-Methyl 3-(benzylamino) octanoate (*rac*-**1e**), from **M*t*E-e**, amber liquid, 60% yield. MS-TOF: calculated for $C_{16}H_{24}N0_2$ 264.1919, found 264.1956. ^{1}H NMR ($CDCl_3$, 300MHz): δ 7.27 (m, 5H), 3.78 (s, 2H), 3.67 (s, 3H), 3.02 (quint, *J*= 6 Hz, 1H), 2.46 (d, *J*= 6 Hz, 2H), 1.46 (m, 8H) + (br, 1H), 0.89 (t, *J*= 6 Hz, 3H). $^{13}C\{^1H\}$ NMR ($CDCl_3$, 300MHz): δ 14.1, 22.7, 25.5, 32.0, 34.4, 39.2, 51.1, 51.6, 54.3, 127.0, 128.2, 128.5, 140.7, 173.2.

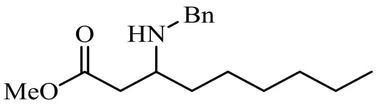

- *rac*-Methyl 3-(benzylamino) nonanoate (*rac*-**1f**), from **M*t*E-f**, amber liquid, 50% yield. MS-TOF: calculated for $C_{17}H_{28}N0_2$ 278.2075, found 278.1156. ^{1}H NMR ($CDCl_3$, 300MHz): δ 7.26 (m, 5H), 3.78 (s, 2H), 3.67 (s, 3H), 3.01 (quint, *J*= 6 Hz, 1H), 2.46 (d, *J*= 6.3 Hz, 2H), 1.46 (m, 10H) + (br, 1H), 0.88 (t, *J*= 5.7 Hz, 3H). ^{13}C{^{1}H} NMR ($CDCl_3$, 300MHz): δ 14.2, 22.7, 25.8, 29.5, 31.9, 34.5, 39.2, 51.1, 51.6, 54.4, 127.0, 128.3, 128.5, 140.7, 173.2.

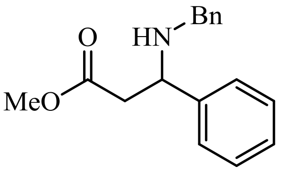

- *rac*-Methyl 3-(benzylamino)-3-phenylpropanoate (*rac*-**1g**) [4], amber liquid. MS-TOF: calculated for $C_{17}H_{20}N0_2$ 270.1494, found 270.1501. 79% yield. ^{1}H NMR ($CDCl_3$, 400MHz): δ 7.39-7.25 (m, 10H), 4.15 (dd, *J*= 5.2, 8.6 Hz, 1H), 3.71-3.55 (m, 5H), 2.77 (dd, *J*= 8.8, 15.6 Hz, 1H), 2.55 (dd, *J*= 5.2, 15.6 Hz, 1H). ^{13}C{^{1}H} NMR ($CDCl_3$, 400MHz): δ 42.8, 51.3, 58.8, 65.3, 126.9, 127.1, 127.5, 128.1, 128.3, 128.6, 140.2, 142.4, 172.2.

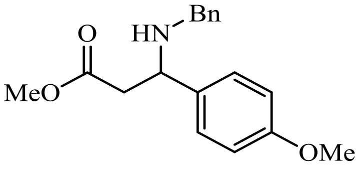

- *rac*-Methyl 3-(benzylamino)-3-(*p*-metoxy)phenylpropanoate (*rac*-**1h**) [13], amber liquid, 50% yield (0.03 mmol). ^{1}H NMR ($CDCl_3$, 300MHz): δ 7.33-7.23 (m, 7H), 6.85 (d, *J*=9 Hz, 2H), 4.06 (q, *J*= 5.4 Hz, 1H), 3.81 (s, 3H), 3.67-3.50 (m, 5H), 2.76-2.57 (m, 2H), 1.97 (br s, 1H). ^{13}C{^{1}H} NMR ($CDCl_3$, 300MHz): δ 43.1, 51.4, 51.8, 55.4, 58.2, 114.1, 127.0, 128.3, 128.5, 134.6, 140.4, 159.0, 172.6.

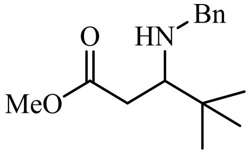

- *rac*-Methyl 3-(benzylamino)-4,4-dimethylpentanoate (*rac*-**1i**) [4], amber liquid. HRMS(ES$^+$): calculated for $C_{15}H_{24}N0_2$ 250.3616, found 250.1812. ^{1}H NMR ($CDCl_3$, 400MHz): δ 7.32-7.23 (m, 5H), 3.90-3.68 (m, 5H), 2.80 (q, *J*= 4.2 Hz, 1H), 2.57 (dd, J=4.2, 14.7 Hz, 1H), 2.29 (dd, *J*= 8.4, 14.7 Hz, 1H), 1.29 (br s, 1H), 0.92 (s, 9H). ^{13}C{^{1}H} NMR ($CDCl_3$, 100MHz): δ 26.7, 35.6, 37.0, 51.8, 54.3, 64.1, 127.0, 128.4, 141.2, 174.3.

3) General method for the synthesis of *N*-benzylated-β^3-amino acids

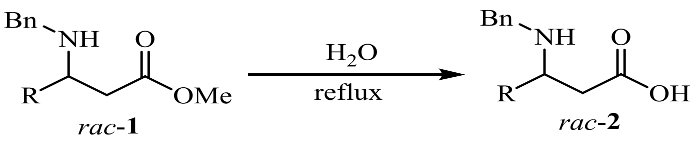

Representative example for the synthesis of *N-benzylated*-β^3-amino acids[5]: The appropriate *N*-benzylated-β^3-amino acid methyl ester is placed in a round flask equipped with a stir bar and 10 mL of water, 1 equivalent of NaOH is added when required (substrates **2c** to **2j**), the reaction was refluxed for 4 hrs. The solution was neutralized, the solvent was evaporated under vacuum and the product was washed with CH_2Cl_2, filtered and decanted with MeOH to obtain a withe off solid.

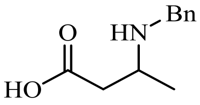

- *rac*-3-(N-benzylamino) butanoic acid (*rac*-**2a**) [4], white off solid, 86% yield. mp 170-171 °C (170-171°C [4]). MS-TOF: calculated for $C_{11}H_{15}N0_2$ 194.1136, found 194.1175. ^{1}H NMR (D_2O, 400MHz): δ 7.42 (s, 5H), 4.18 (q, *J*= 16.8 Hz, 2H), 3.52 (m, *J*= 6.4 Hz, 1H), 3.28 (br s, 1H), 2.59 (dd, *J*=4.8, 13 Hz, 1H), 2.54 (dd, *J*=4.5, 12.8 Hz, 1H), 1.32 (d, *J*= 6.4 Hz, 3H). ^{13}C{^{1}H} NMR (D_2O, 400MHz): δ 15.9, 38.2, 48.1, 51.2, 129.3, 129.6, 130.9, 176.4.

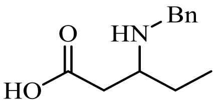

- *rac*-3-(N-benzylamino) pentanoic acid (*rac*-**2b**) [14], 89% yield. white solid. mp 167-169°C (169-170°C [14]). MS-TOF: calculated for $C_{11}H_{15}N0_2$ 208.1293, found 208.1331. ^{1}H NMR (D_2O, 400MHz): δ 7.40 (s, 5H), 4.17 (q, *J*= 9, 1 Hz, 2H), 3.31 (m, 1H), 2.56 (dd, *J*=3.6, 12.9 Hz, 1H), 2.40 (dd, *J*=5.4, 12.7 Hz, 1H), 1.78 (m, 1H), 1.59 (m, 1H),

0.88 (t, *J*= 7.6Hz, 3H). $^{13}C\{^1H\}$ NMR (D_2O, 400MHz): δ 8.8, 23.3, 35.3, 47.9, 56.7, 129.3, 129.5, 131.2, 178.2.

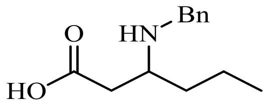

- *rac*-3-(N-benzylamino) hexanoic acid (*rac*-**2c**) [14], 97% yield. white solid. mp 156-159°C (160-162°C [14]). MS-TOF: calculated for $C_{13}H_{20}N0_2$ 222.1449, found 222.1488. ^1H NMR (D_2O, 400MHz): δ 7.40 (s, 5H), 4.18 (q, *J*= 10.2 Hz, 2H), 3.55 (m, 1H), 2.56 (dd, *J*= 3.6, 12.6 Hz, 1H), 2.40 (dd, *J*= 5.7, 12.6 Hz, 1H), 1.70 (m, 1H), 1.55 (m, 1H), 1.30 (m, 2H), 0.83 (t, *J*= 5.7Hz, 3H). $^{13}C\{^1H\}$ NMR (D_2O, 400MHz): δ 12.9, 18.0, 32.3, 35.9, 47.8, 55.1, 129.3, 129.4, 129.5, 131.4, 178.3.

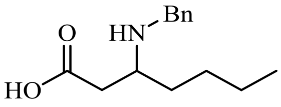

- *rac*-3-(N-benzylamino) heptanoic acid (*rac*-**2d**) [15], 84% yield. white solid. mp 152-155°C. MS-TOF: calculated for $C_{14}H_{22}N0_2$ 236.1606, found 236.1644. ^1H NMR (D_2O, 400MHz): δ 7.26 (s, 5H), 4.0 (t, *J*= 10.2 Hz, 2H), 3.32 (m, 1H), 2.68 (dd, *J*= 4.8, 17.8 Hz, 1H), 2.28 (dd, *J*= 6.8, 18 Hz, 1H), 1.60 (m, 1H), 1.47 (m, 1H), 1.08 (m, 4H), 0.63 (t, *J*= 6.4 Hz, 3H). $^{13}C\{^1H\}$ NMR (D_2O, 400MHz): δ 12.9, 21.4, 26.4, 29.5, 34.0, 48.5, 54.1, 129.1, 129.5, 129.7, 130.3, 137.9.

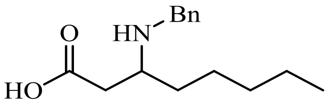

- *rac*-3-(N-benzylamino) octanoic acid (*rac*-**2e**), 80% yield. white solid. mp 150-152°C. MS-TOF: calculated for $C_{15}H_{24}N0_2$ 250.1762, found 250.1803. ^1H NMR (D_2O, 400MHz): δ 7.25 (s, 5H), 4.03 (s, 2H), 3.32 (m, 1H), 2.67 (dd, *J*= 4.8, 18 Hz, 1H), 2.28 (dd, *J*= 6.8,

17.6 Hz, 1H), 1.58 (m, 1H), 1.46 (m, 1H), 1.03 (m, 6H), 0.60 (t, *J*= 6.4 Hz, 3H). $^{13}C\{^{1}H\}$ NMR (D_2O, 400MHz): δ 13.0, 21.4, 23.9, 29.7, 30.3, 34.0, 48.5, 54.1, 129.1, 129.5, 129.7, 130.2, 137.9.

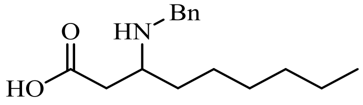

- *rac*-3-(N-benzylamino) nonanoic acid (*rac*-**2f**), 83% yield. white solid. mp 151-153°C MS-TOF: calculated for $C_{16}H_{26}N0_2$ 264.1919, found 264.2005. ^{1}H NMR (D_2O, 400MHz): δ 7.36 (s, 5H), 4.18 (s, 2H), 3.41 (m, 1H), 2.76 (dd, *J*= 4.8, 17.8 Hz, 1H), 2.67 (dd, *J*= 6.8, 17.6 Hz, 1H), 1.69 (m, 1H), 1.56 (m, 1H), 1.16 (m, 8H), 0.72 (t, *J*= 6 Hz, 3H). $^{13}C\{^{1}H\}$ NMR (D_2O, 400MHz): δ 13.2, 21.7, 24.2, 27.8, 29.7, 30.6, 34.1, 48.6, 54.0, 129.2, 129.7, 129.8, 130.3, 174.0.

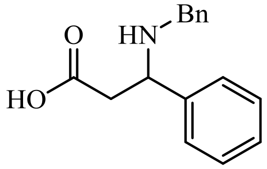

- *rac*-3-(N-benzylamino)-3-phenylpropanoic acid (*rac*-**2g**) [4], 80% yield. white solid. mp 149-151°C (148-151°C [4]).^{1}H NMR (CD_3OD, drops NH_4OH, 200MHz): δ 7.42-7.22 (m, 10H), 4.95 (dd, *J*= 4.4, 10.4, 1H), 3.33 (s, 2H), 2.63 (dd, *J*= 10.4, 16.8, 1H), 2.48 (dd, *J*= 4.4, 17.2, 1H), $^{13}C\{^{1}H\}$ NMR (CD_3OD+NH_4OH, 50MHz): δ 45.3, 50.9, 60.3, 126.6, 126.9, 127.3, 128.0, 128.1, 139.3, 142.6, 178.3.

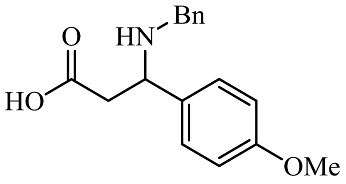

- *rac*-3-(N-benzylamino)-3-(*p*-methoxy)phenylpropanoic acid (*rac*-**2h**), 54% yield. white solid. mp 172-174°C. MS-TOF: calculated for $C_{16}H_{25}N0_2$ 263.1885, found 263.2005. ^{1}H NMR (CD_3OD, 400MHz): δ 7.26 (d, *J*= 9.6 Hz, 7H), 7.04 (d, *J*= 8 Hz, 2H), 4.45 (dd, J=

3.2, 10.2 Hz 1H), 2.76 (dd, *J*= 4.8, 17.8 Hz, 1H), 4.00 (dd, *J*= 13.2, 25.2 Hz, 2H), 3.85 (s, 3H), 2.86 (dd, *J*= 10.8, 16.6 Hz, 1H), 2.65 (dd, *J*= 3.6, 16.8 Hz, 2H). $^{13}C\{^1H\}$ NMR (CD_3OD, 400MHz): δ 38.5, 48.1, 54.4, 59.3, 14.4, 126.4, 128.9, 128.9, 129.0, 129.1, 131.8, 160.7, 176.0.

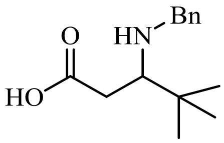

- *rac*-3-(N-benzylamino)-4,4-dimethylpentanoic acid (*rac*-**2i**) [4], 88% yield. white solid. mp 152-153°C (150-153°C [4]). HRMS (ES^+): calculated for $C_{14}H_{22}N0_2$ 236.1651, found 236.1638. ^1H NMR (D_2O) δ 7.27 (s, 5H), 4.28 (m, 1H), 4.04 (m, 1H), 3.14 (m, 1H), 2.69 (m, 1H), 2.53 (m, 1H), 0.75 (m, 9H) $^{13}C\{^1H\}$ NMR (CD_3OD, 100MHz): δ 24.6, 32.0, 33.3, 50.6, 62.6, 129.1, 129.8, 130.2, 174.3.

4) General method for the enzymatic resolution using CALB.

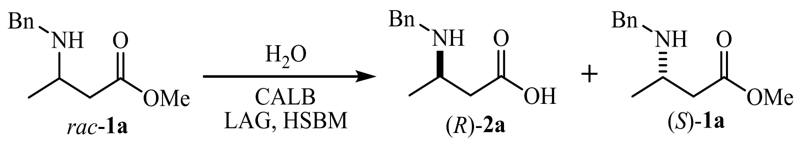

Representative example for the enzymatic resolution using CALB and *rac*-**1a**: 82 mg of substrate *rac*-**1a** (0.4 mmol), 3.6 μL of water (0.2 mmol) and 0.2 mL of 2M2B were placed in an Agate jar (12 mm of Diameter) with an Agate ball (6 mm of Diameter, 480mg of weight), 40 mg of enzyme (CALB) were aggregated and the reactor was closed and placed in a Mixer Mill MM200 programed to carry out the reaction at 25 Hz during 30 min. Once the reaction is finished the content was extracted with methanol and transferred to a Falcon tube (50 mL), the solution was centrifuged at 3500 rpm for 5 min twice; the supernatant was collected and concentrated for his purification with silica gel, a mixture of Hexanes and ethyl acetate was used as mobile phase for recovering the raw material and Methanol and CH_2Cl_2 for the purification of the product. Methyl 3-(benzylamino) butanoic acid (*R*)-**2a** was obtain as a white solid (49% yield). In order to avoid contamination by the wear of the stainless-steel reactors we decided to use Agate reactors.

Table S1. Search of the best parameters for the enzymatic hydrolysis resolution under HSBM.

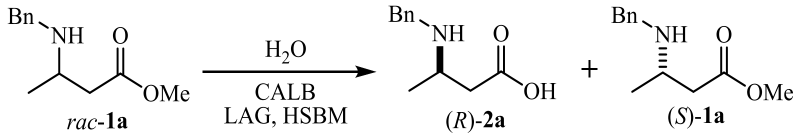

entry[a]	LAG	frequency (Hz)	time (h)	(*S*)-**1a**			(*R*)-**2a**			c^f (%)	E^g
				recovered[b] (%)	*ee*[c] (%)	$[\alpha]_D^{25}$[d]	yield[b] (%)	*ee*[c] (%)	$[\alpha]_D^{25}$[e]		
1	2M2B	25	0.5	51	99	20.4	49	80	-31.7	55	46
2	2M2B	15	0.5	70	89	22.0	30	77	-33.1	54	23
**3**	**2M2B**	**15**	**1**	**51**	**99**	**22.5**	**49**	**95**	**-30.0**	**51**	**>200**
4	Hexane	15	1	40	97	23.3	60	86	-20.4	53	55
5	AcOEt	15	1	86	69	10.3	13	95	-30.1	42	81
6	DIPE	15	1	76	92	17.3	19	88	-25.6	51	51
7	Toluene	15	1	75	72	12.6	25	93	-29.5	44	60
8	Dioxane	15	1	53	79	16.7	47	95	-30.3	45	94
9	IPA	15	1	80	48	7.4	20	95	-29.8	34	63
10	CH_3CN	15	1	65	65	9.8	29	95	-30.0	41	77
11	-	15	1	58	95	16.6	41	92	-28.2	51	89
12	-	25	1	58	93	16.5	42	86	-31.2	52	45
13[h]	-	15	1	68	74	13.5	31	80	-27.0	48	20
14[i]	2M2B	15	1	-	-	-	92	*rac*	-	-	-
15[j]	2M2B	15	1	89	*rac*	-	-	-	-	-	-

[a]Reactions were carried out using the general method. [b]Determinated after purification by flash chromatography. [c]Determined by HPLC with chiral stationary phase. [d]Using CH_3Cl and c=0.33, [e]Using MeOH and c=0.33, [f]Calculated from $c = ee_s/(ee_s + ee_p)$. [g]$E = \ln[1-c(1+ee_p)]/\ln[1-c(1-ee_p)]$. [h]0.25 equivalents of water were used. [i]1 equivalent of water were used. [j] water-free conditions.

Table S2. Substrate scope for the enzymatic resolution of N-benzylated-β[3]-amino esters.

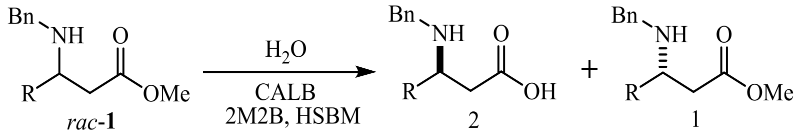

entry[a]	*rac-1*	R	Substrate-1 recovered[b] (%)	Substrate-1 *ee*[c] (%)	Substrate-1 $[\alpha]_D^{25}$	2 yield[b] (%)	2 *ee*[c] (%)	2 $[\alpha]_D^{25}$	*c*[d] (%)	*E*[e]	a.c.[f]
1	1b	CH_3-(CH_2)-	51	91	4.5	49	97	-36.5	48	>200	*R*
2	1c	CH_3-(CH_2)$_2$-	53	84	2.1	43	98	-45.2	46	>200	*R*
3	1d	CH_3-(CH_2)$_3$-	68	23	2.0	29	94	-35.3	20	40	*R*
4[g]	1d	CH_3-(CH_2)$_3$-	66	57	0.2	25	94	-33.3	38	58	*R*
5[h]	1d	CH_3-(CH_2)$_3$-	51	30	2.7	41	85	-40.5	26	17	*R*
6	1e	CH_3-(CH_2)$_4$-	74	16	1.8	24	94	-40.0	15	38	*R*
7[g]	1e	CH_3-(CH_2)$_4$-	76	27	0.8	22	82	-30.2	25	13	*R*
8[h]	1e	CH_3-(CH_2)$_4$-	82	27	2.7	10	76	-13.0	26	10	*R*
9	1f	CH_3-(CH_2)$_5$-	79	13	0.8	18	91	-39.7	13	24	*R*
10[g]	1f	CH_3-(CH_2)$_5$-	85	21	1.1	11	82	-28.5	20	12	*R*
11[h]	1f	CH_3-(CH_2)$_5$-	84	18	0.7	8	83	-28.9	18	13	*R*
12[i]	1g	Ph	90	18	3.4	10	83	-35.0	18	13	*S*
13[i, h]	1g	Ph	85	18	3.0	15	68	-44.0	21	6	*S*
14[j]	1g	Ph	90	16	3.2	9	37	-18.1	30	3	*S*
15[i, k]	1g	Ph	80	5	2.5	20	48	-21.0	9	3	*S*
16	1h	*p*-MeO-Ph	89	1	-0.5	10	80	-31.7	1	9	*S*
17	1i	*t*-Bu	89	4	-0.6	4	94	12.8	4	34	*S*
18[h]	1i	*t*-Bu	87	5	-0.7	6	89	16.6	5	18	*S*

[a]Reactions were carried out using the general method. [b]Determined after purification by flash chromatography. [c]Determined by HPLC with chiral stationary phase. [d]Calculated from $c = ee_s/(ee_s + ee_p)$. [e]$E = \ln[1-c(1+ee_p)]/\ln[1-c(1-ee_p)]$. **a.c**. Absolute Configuration of product **2.** [f] Assigned by chemical comparison and HPLC with chiral stationary phase. [g] Carried out with 15 Hz during 1 h. [h] Carried out with 25 Hz during 1 h. [i] 0.75 equiv. of water were used. [j] 1 equiv. of water were used. [k] 2 equiv. of enzyme were used.

Table S3. Recycling capacity of immobilized CALB under HSBM conditions.

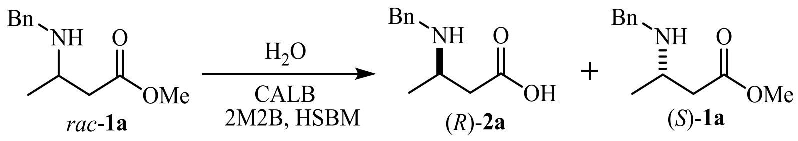

entry[a]	rCALB (cycle)	(S)-1a recovered[b] (%)	(S)-1a *ee*[c] (%)	(S)-1a $[\alpha]_D^{25}$	(R)-2a yield[b] (%)	(R)-2a *ee*[c] (%)	(R)-2a $[\alpha]_D^{25}$	*c*[d] (%)	*E*[e]
1	-	51	>99	22.0	49	95	-31.7	51	>200
2	1	65	35	2.8	35	88	-33.3	28	22
3	2	80	6	0.5	20	80	-53.9	7	10
4	3	100	0	0.0	0	0	-	-	-
5[f]	1	60	67	15.6	38	94	-29.2	42	65
6[g]	1	62	83	15.9	38	91	-26.8	48	55

[a]Reactions were carried out using the general method. [b]Determined after purification by flash chromatography. [c]Determined by HPLC with chiral stationary phase. [d]Calculated from $c = ee_s/(ee_s + ee_p)$. [e]$E = \ln[1-c(1+ee_p)]/\ln[1-c(1-ee_p)]$. [f]Enzyme with a pretreatment (Milling for 1h at 15 Hz). [g]Pretreatment was carried out using 0.2mL of LAG.

Table S4. Scaling-up for the enzymatic hydrolysis using the substrate *rac*-1a.

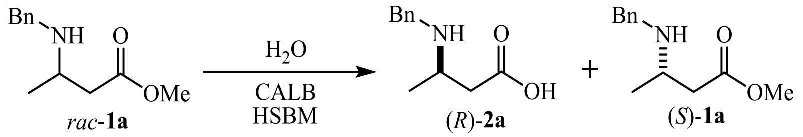

entry[a]	substrate equivalents	(S)-1a recovered[b] (%)	(S)-1a ee^c (%)	(S)-1a $[\alpha]_D^{25}$	(R)-2a yield[b] (%)	(R)-2a ee^c (%)	(R)-2a $[\alpha]_D^{25}$	c^d (%)	E^e
1	1	51	>99	22.0	49	95	-31.7	51	>200
2	3	52	62	8.0	48	93	-33.0	40	52
3	6	61	53	7.8	38	93	-33.9	36	47
4	9	59	49	7.0	40	94	-33.0	34	53

[a]Reactions were carried out using the general method but in absence of LAG. [b]Determined after purification by flash chromatography. [c]Determined by HPLC with chiral stationary phase. [d]Calculated from $c = ee_s/(ee_s + ee_p)$. [e]$E = \ln[1-c(1+ee_p)]/\ln[1-c(1-ee_p)]$.

5) ^{1}H and ^{13}C NMR spectra for TPA, M*t*E-b to M*t*E-d, *rac*-1a to *rac*-1j and products 2a to 2j.

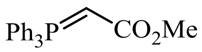

TPA

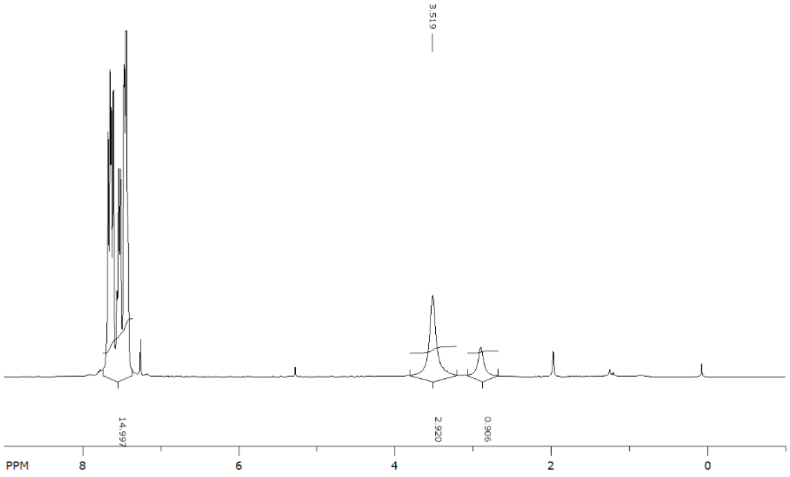

Figure S1. ^{1}H spectrum (300 MHz, $CDCl_3$) of TPAc

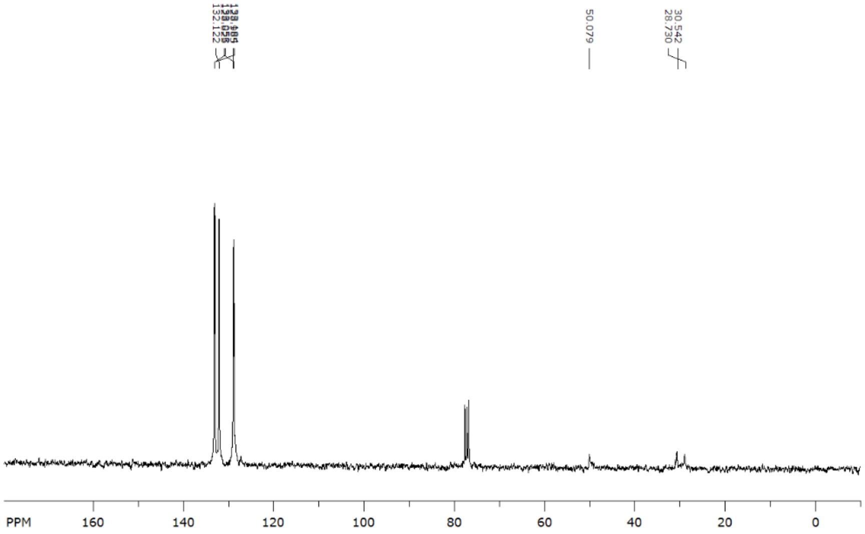

Figure S2. ^{13}C spectrum (75 MHz, $CDCl_3$) of TPA

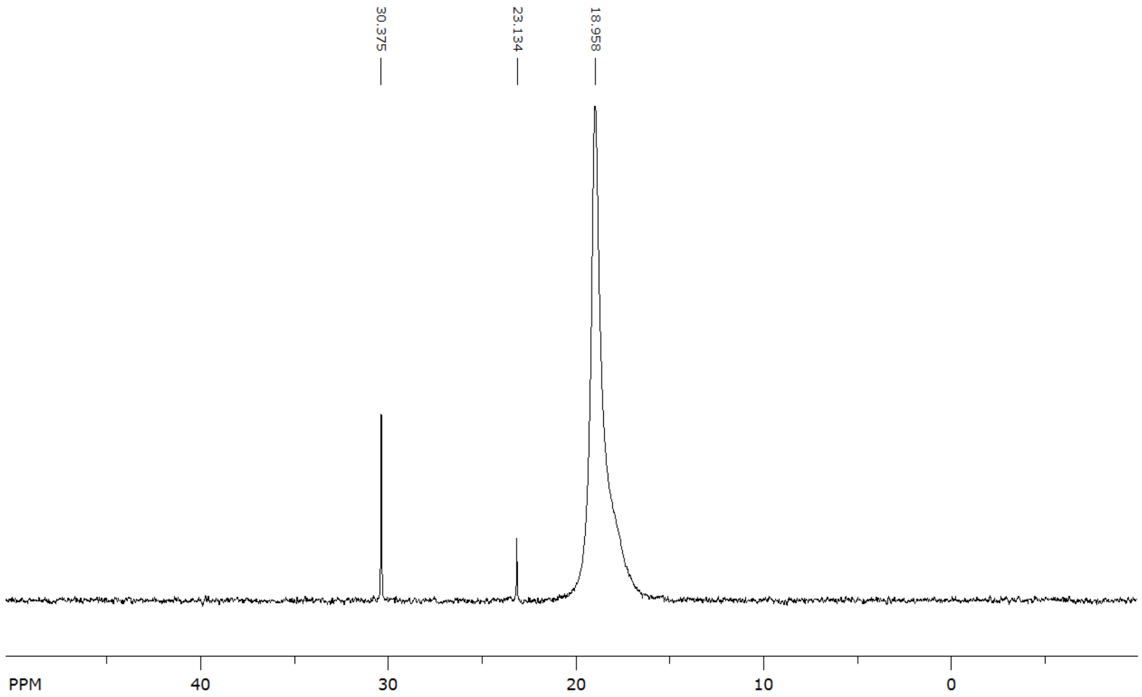

Figure S3. ^{31}P **spectrum (120 MHz,** $CDCl_3$**) of TPA**

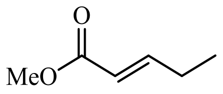

M*t*E-b

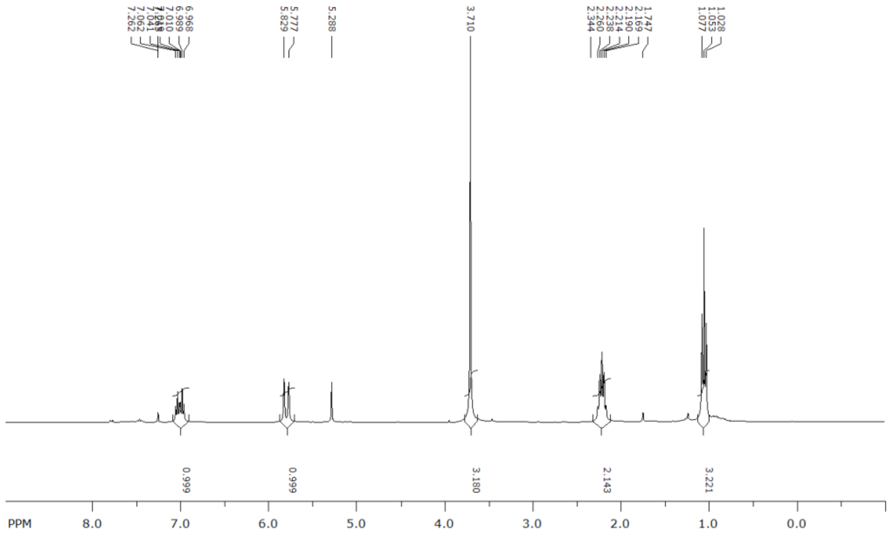

Figure S4. ^{1}H spectrum (300 MHz, $CDCl_3$) of M*t*E-b

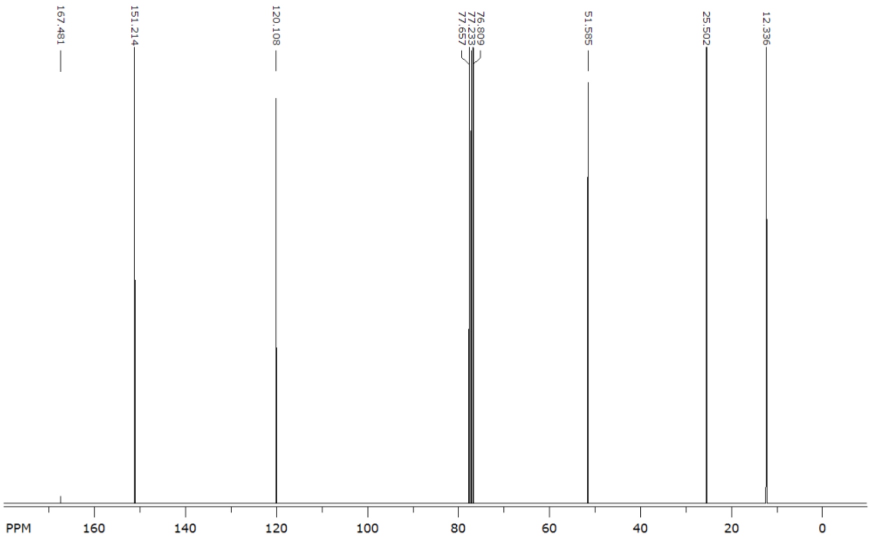

Figure S5. ^{13}C spectrum (75 MHz, $CDCl_3$) of M*t*E-b

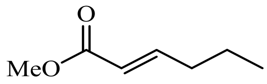

M*t*E-c

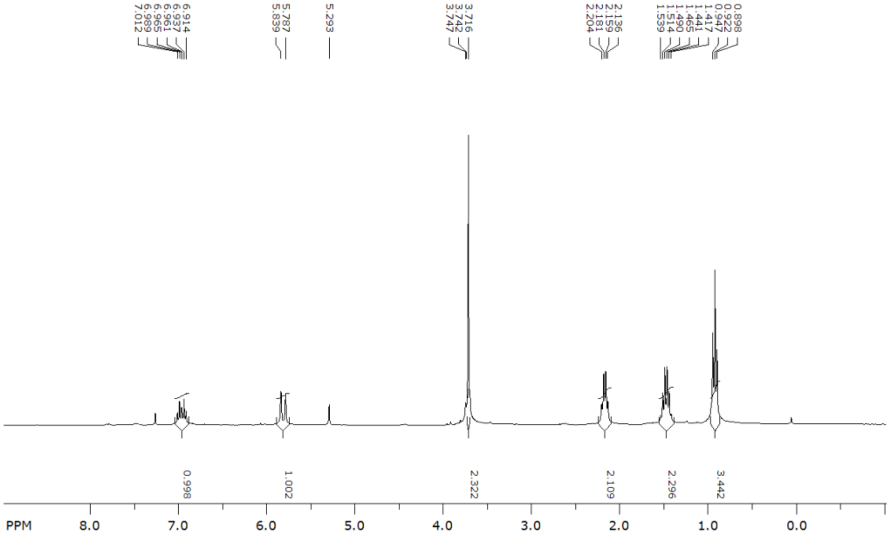

Figure S6. ^{1}H spectrum (300 MHz, $CDCl_3$) of M*t*E-c

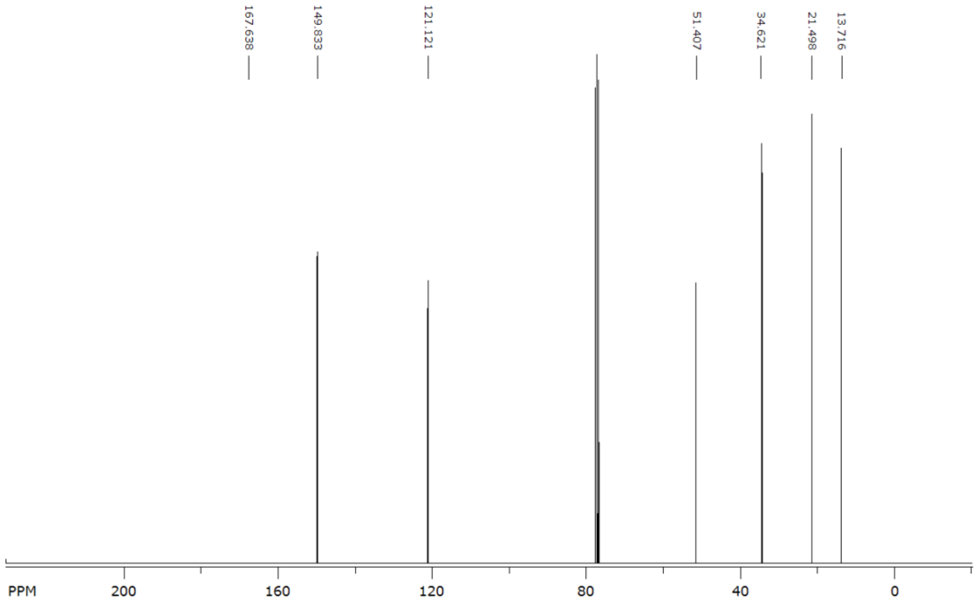

Figure S7. ^{13}C spectrum (75 MHz, $CDCl_3$) of M*t*E-c

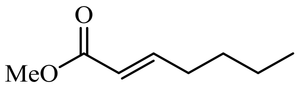

M*t*E-c

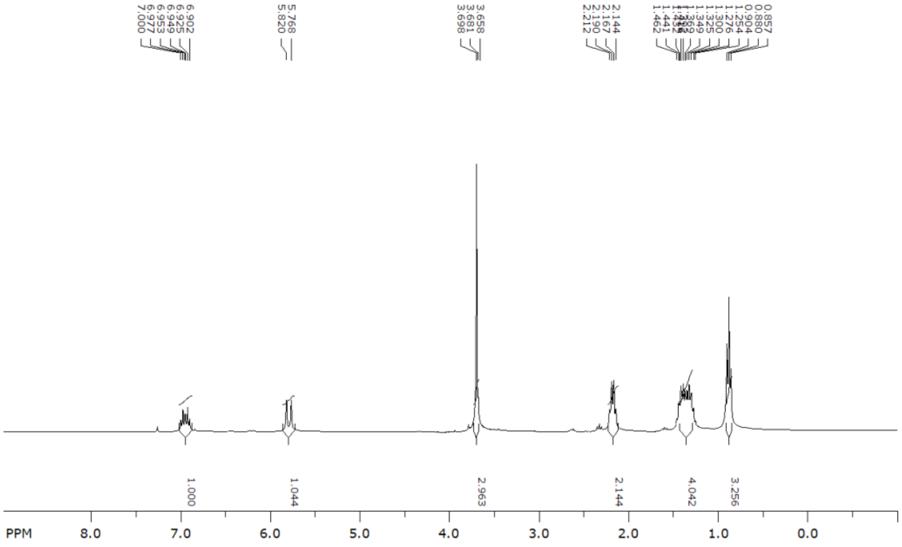

Figure S8. ^{1}H spectrum (300 MHz, $CDCl_3$) of M*t*E-d

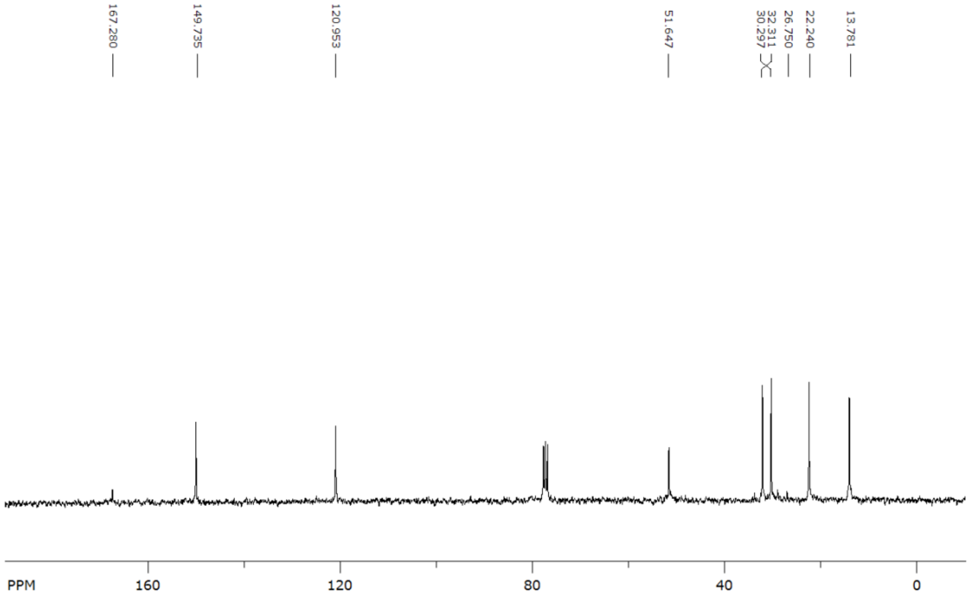

Figure S9. ^{13}C spectrum (75 MHz, $CDCl_3$) of M*t*E-d

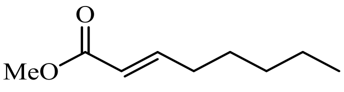

M*t*E-d

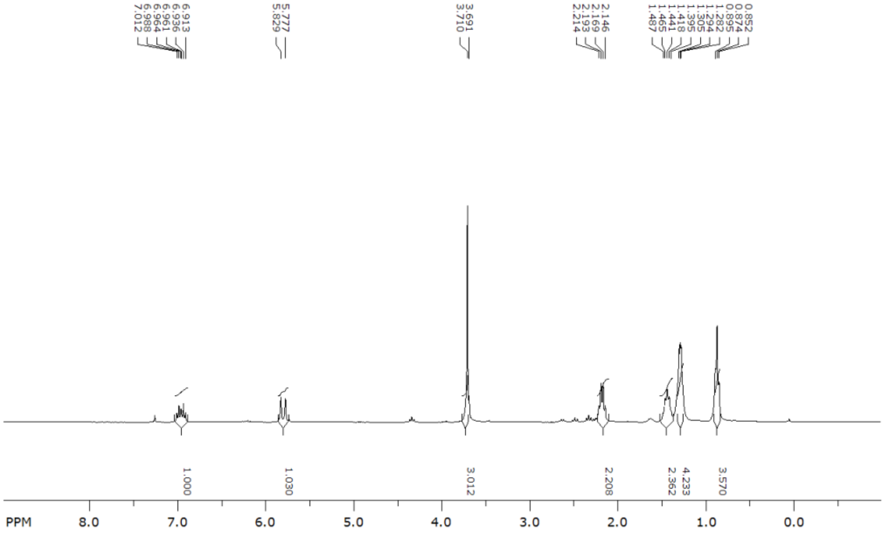

Figure S10. ^{1}H spectrum (300 MHz, $CDCl_3$) of M*t*E-e

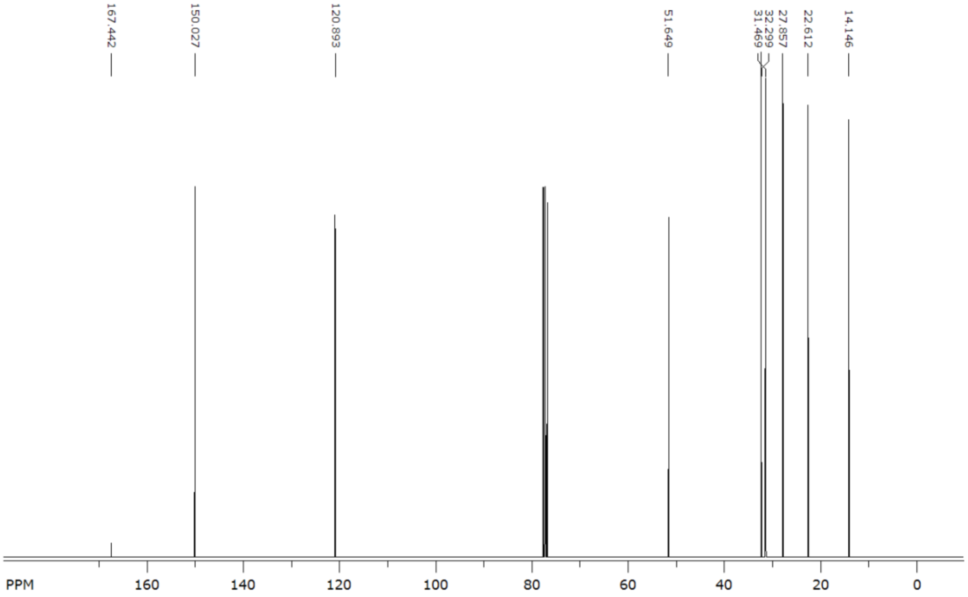

Figure S11. ^{13}C spectrum (75 MHz, $CDCl_3$) of M*t*E-e

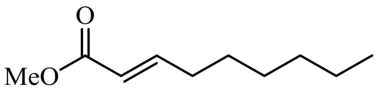

M*t*E-e

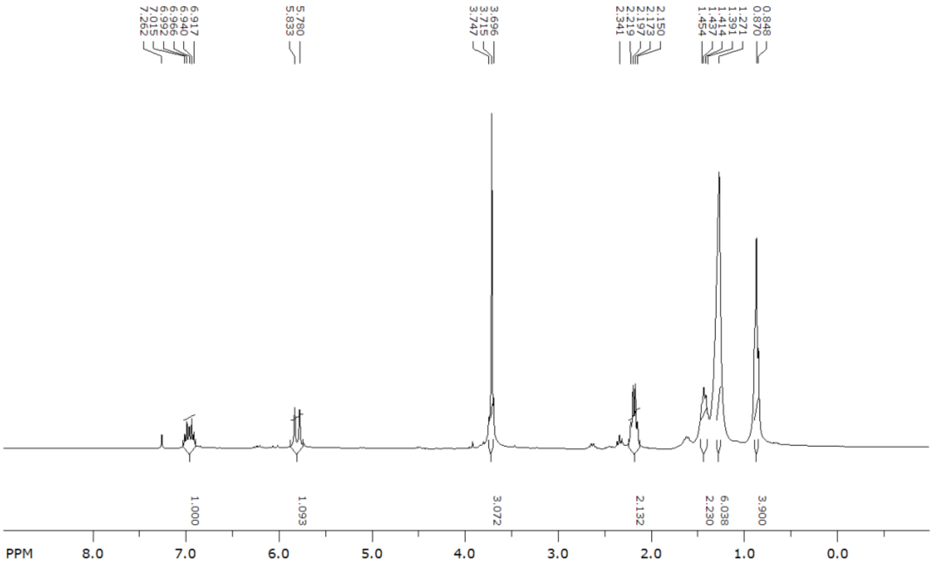

Figure S12. ^{1}H spectrum (300 MHz, $CDCl_3$) of M*t*E-f

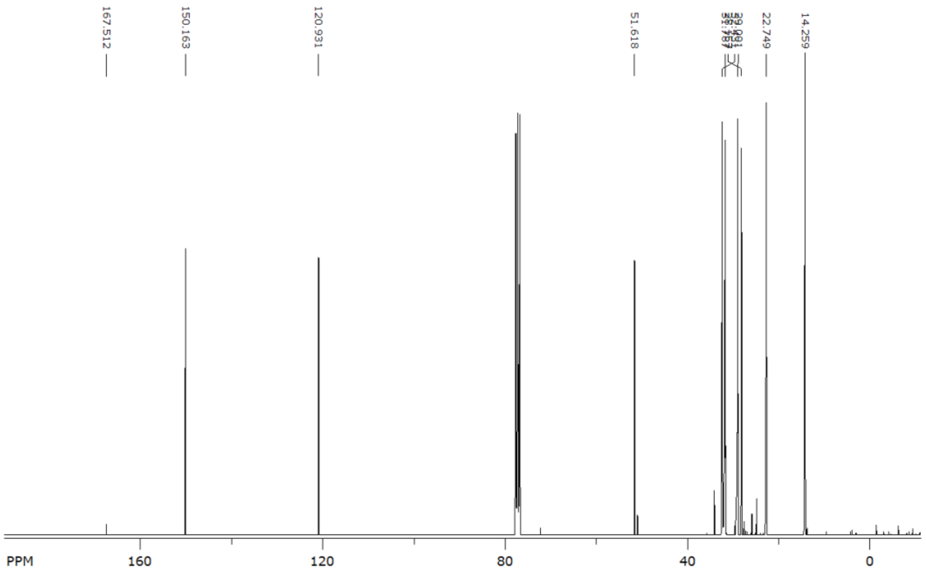

Figure S13. ^{13}C spectrum (75 MHz, $CDCl_3$) of M*t*E-f

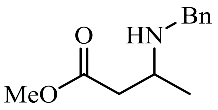

rac-**1a**

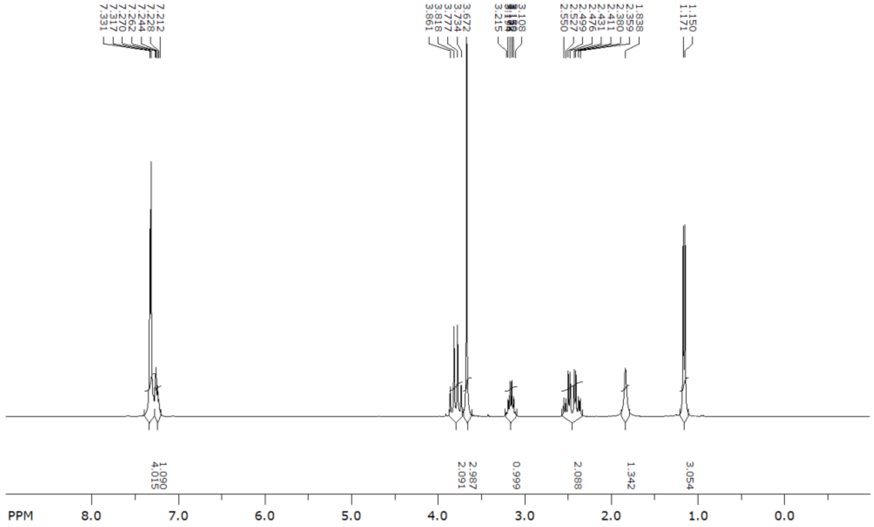

Figure S14. ^{1}**H spectrum (300 MHz,** $CDCl_3$**) of** ***rac-1a***

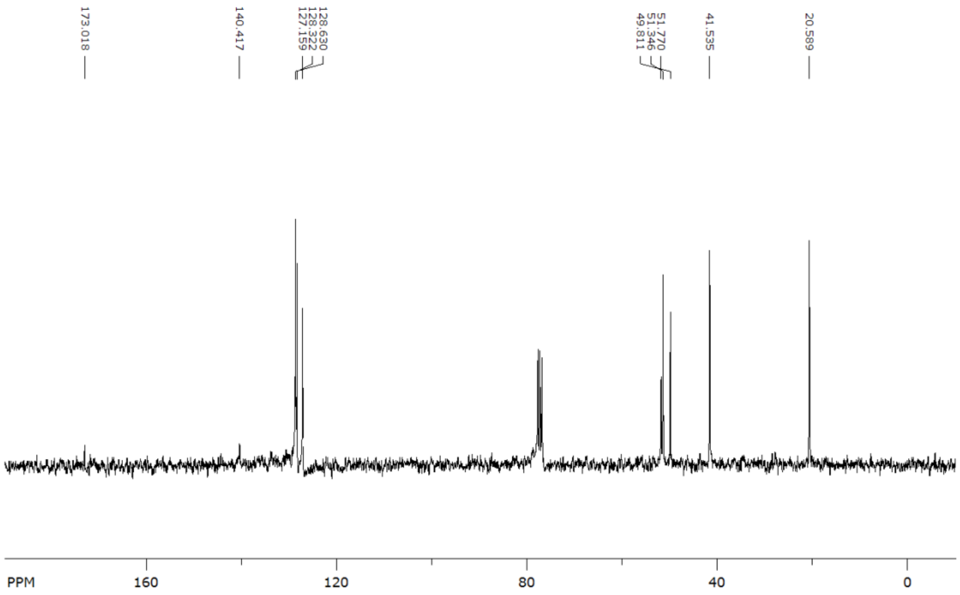

Figure S15. ^{13}**C spectrum (75 MHz,** $CDCl_3$**) of** ***rac-1a***

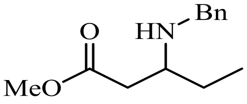

rac-**1b**

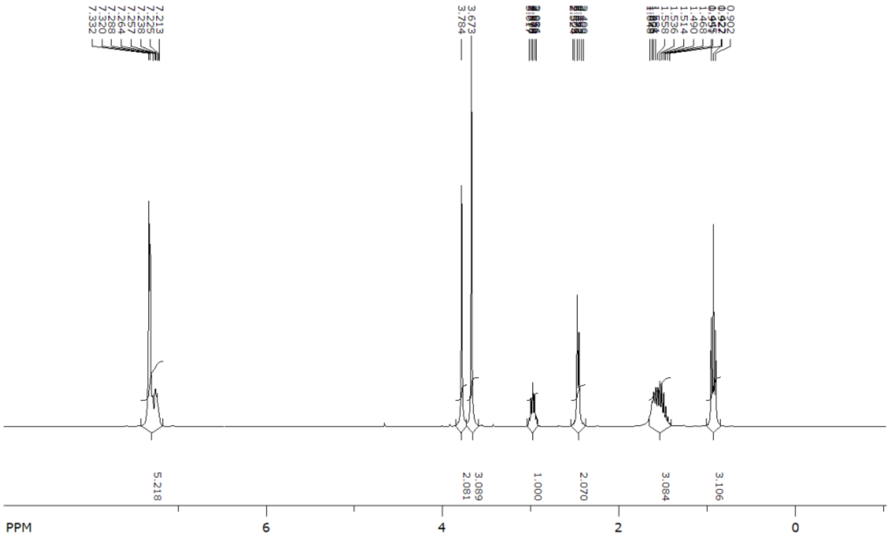

Figure S16. ^{1}H spectrum (300 MHz, $CDCl_3$) of *rac-1b*

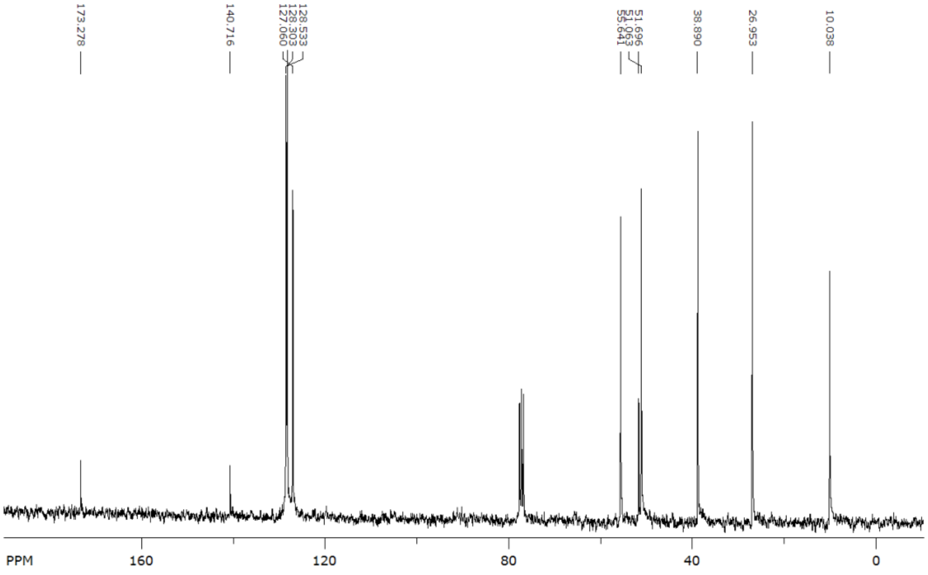

Figure S17. ^{13}C spectrum (75 MHz, $CDCl_3$) of *rac-1b*

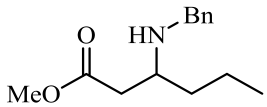

rac-**1c**

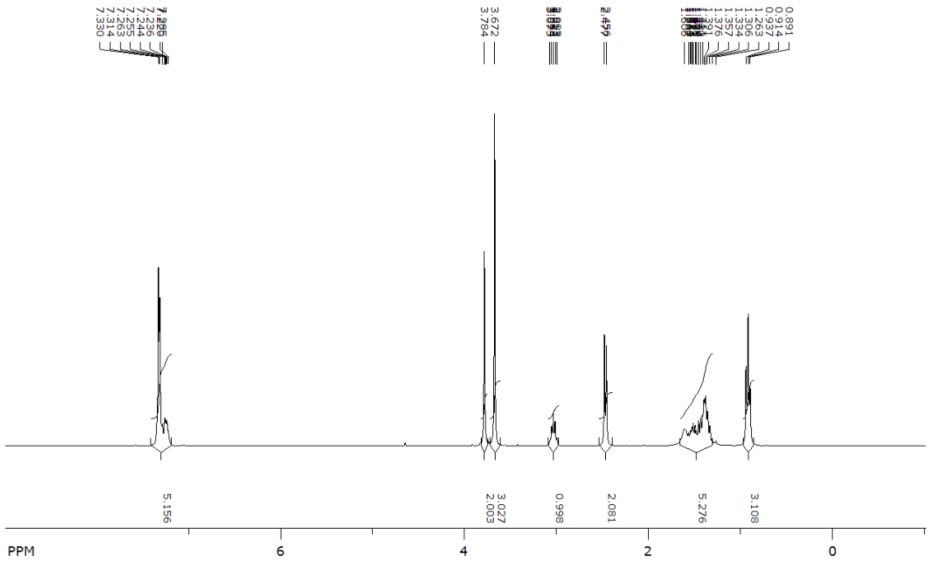

Figure S18. ^{1}H spectrum (300 MHz, $CDCl_3$) of *rac-1c*

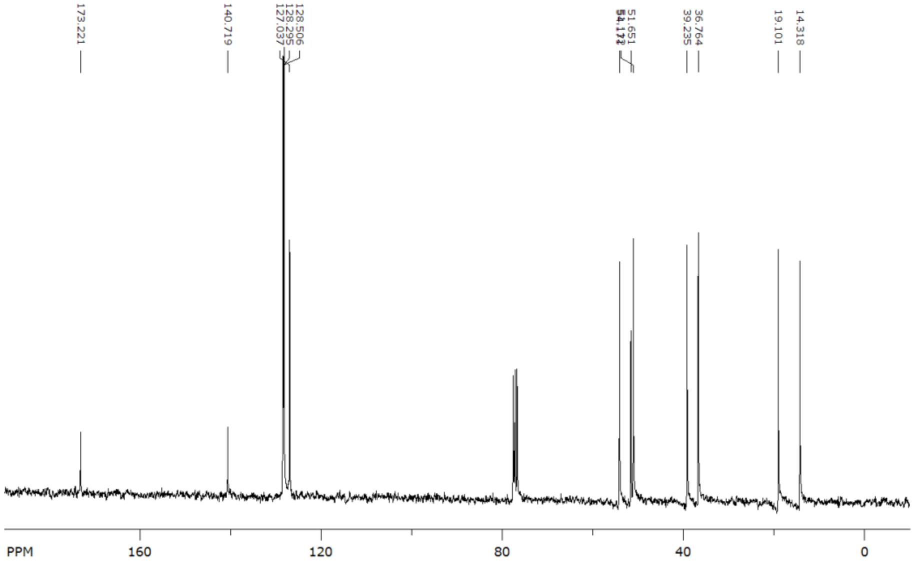

Figure S19. ^{13}C spectrum (75 MHz, $CDCl_3$) of *rac-1c*

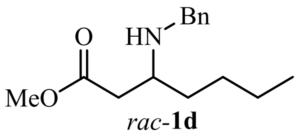

rac-**1d**

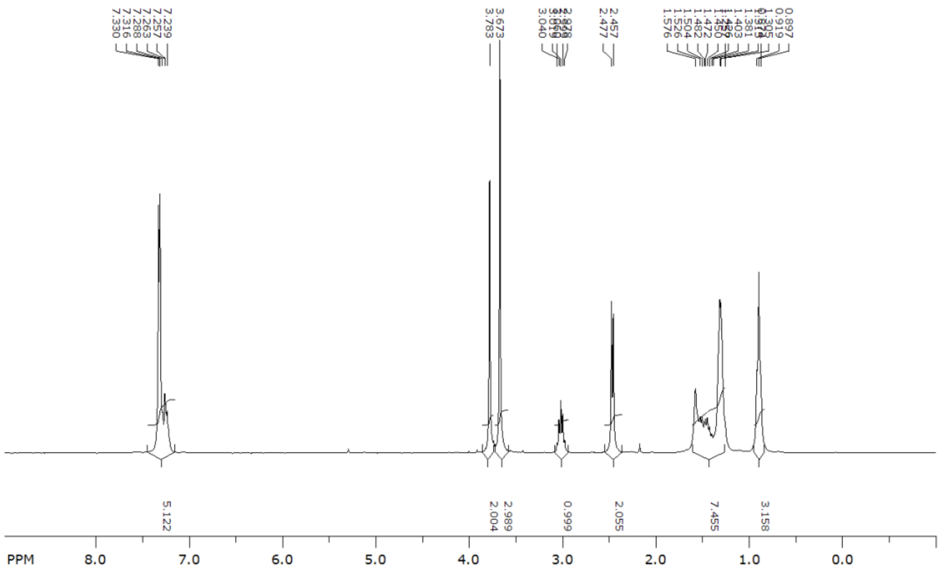

Figure S20. ^{1}H spectrum (300 MHz, $CDCl_3$) of *rac-1d*

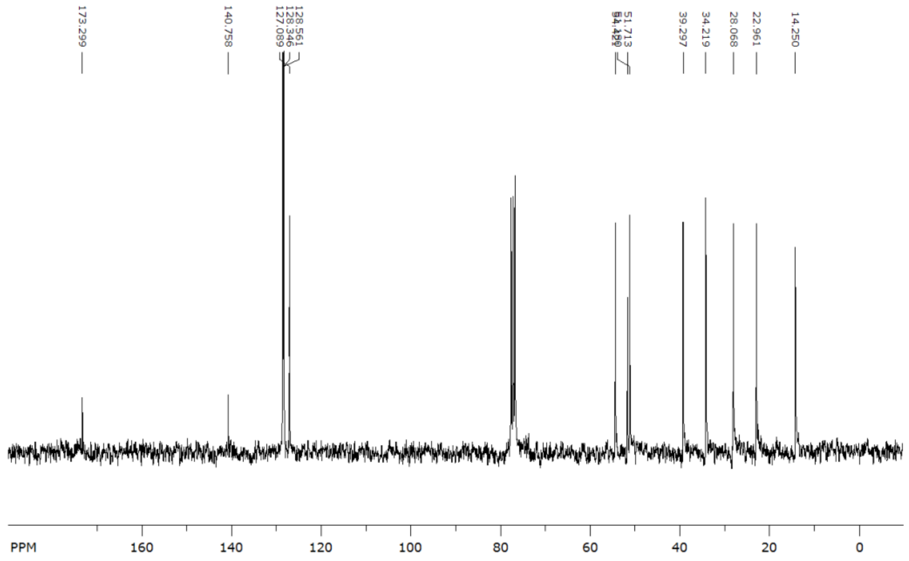

Figure S21. ^{13}C spectrum (75 MHz, $CDCl_3$) of *rac-1d*

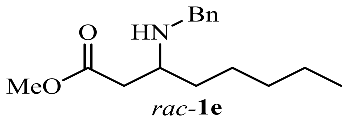

rac-**1e**

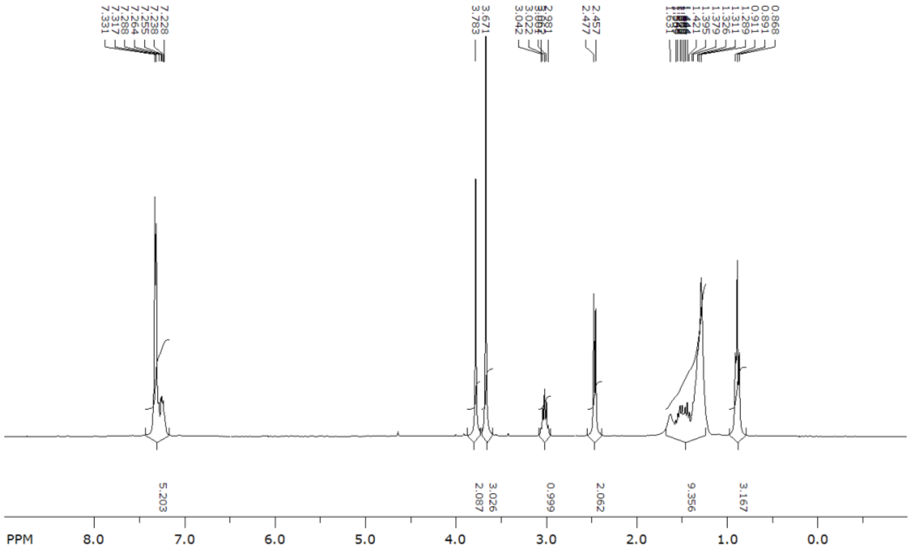

Figure S22. ^{1}H spectrum (300 MHz, $CDCl_3$) of *rac-1e*

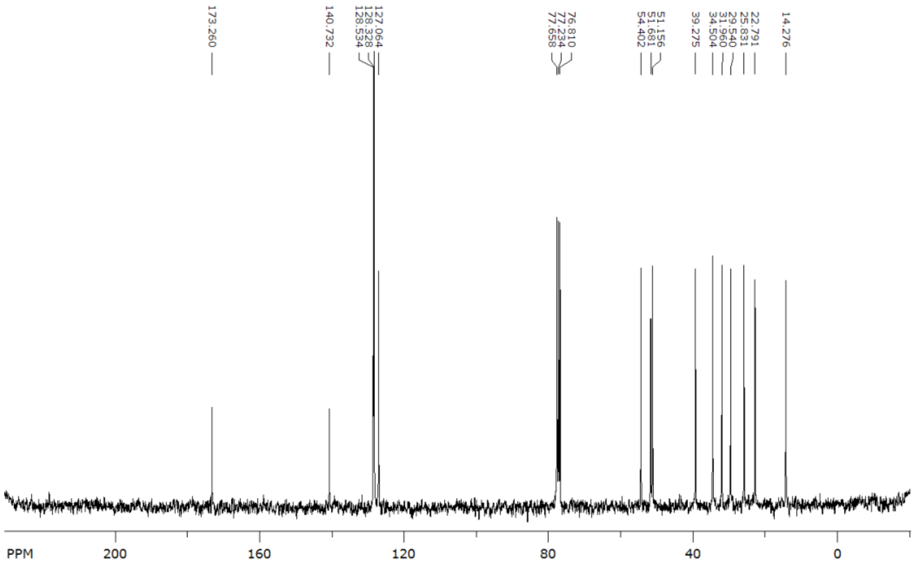

Figure S23. ^{13}C spectrum (75 MHz, $CDCl_3$) of *rac-1e*

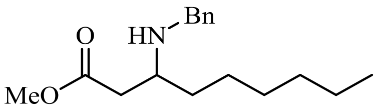

rac-**1f**

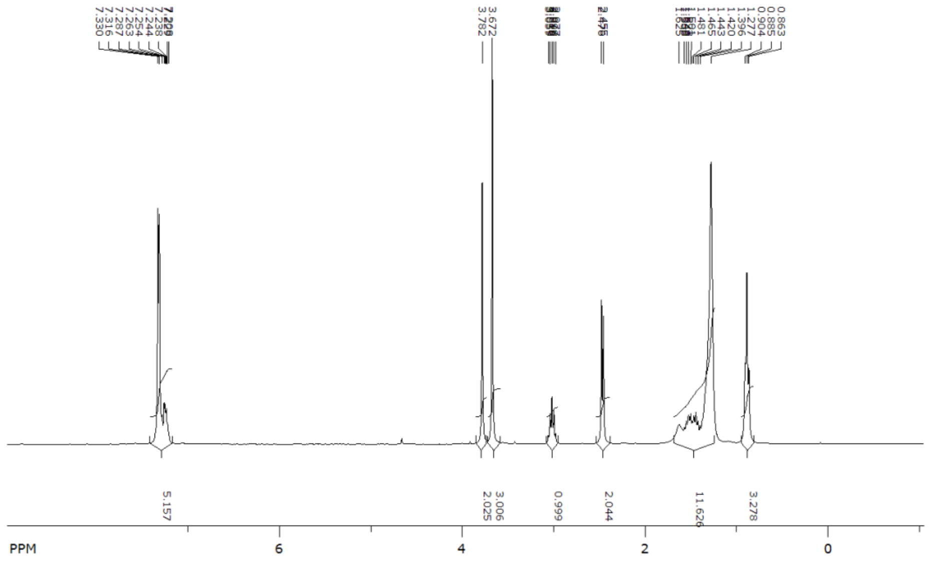

Figure S24. ^{1}H spectrum (300 MHz, $CDCl_3$) of *rac-1f*

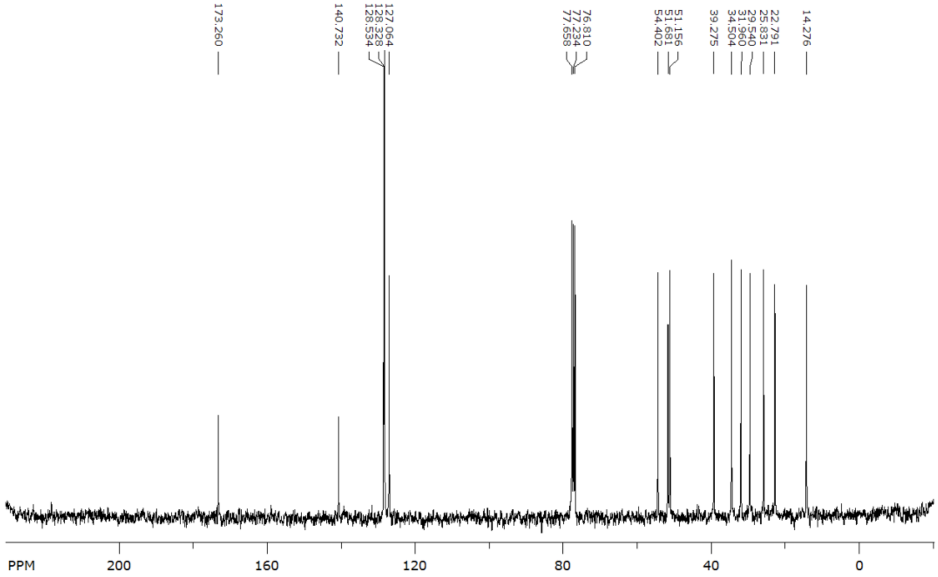

Figure S25. ^{13}C spectrum (75 MHz, $CDCl_3$) of *rac-1f*

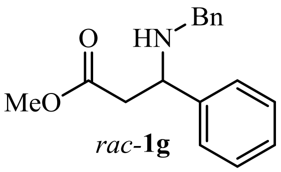

rac-**1g**

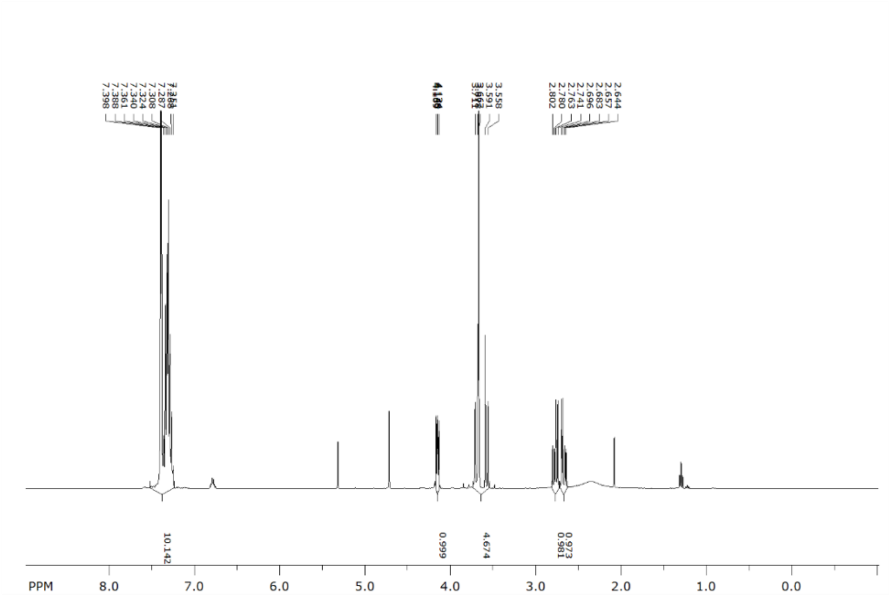

Figure S26. ^{1}**H spectrum (300 MHz,** $CDCl_3$**) of *rac-1g.***

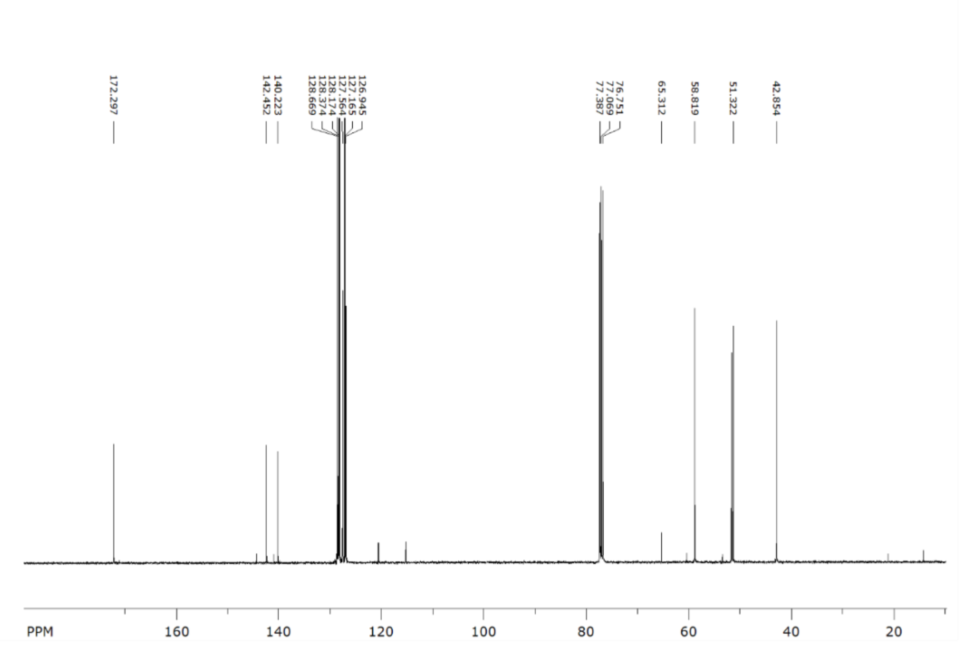

Figure S27. ^{13}**C spectrum (75 MHz,** $CDCl_3$**) of *rac-1g.***

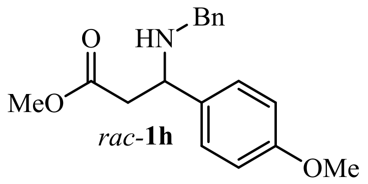

rac-**1h**

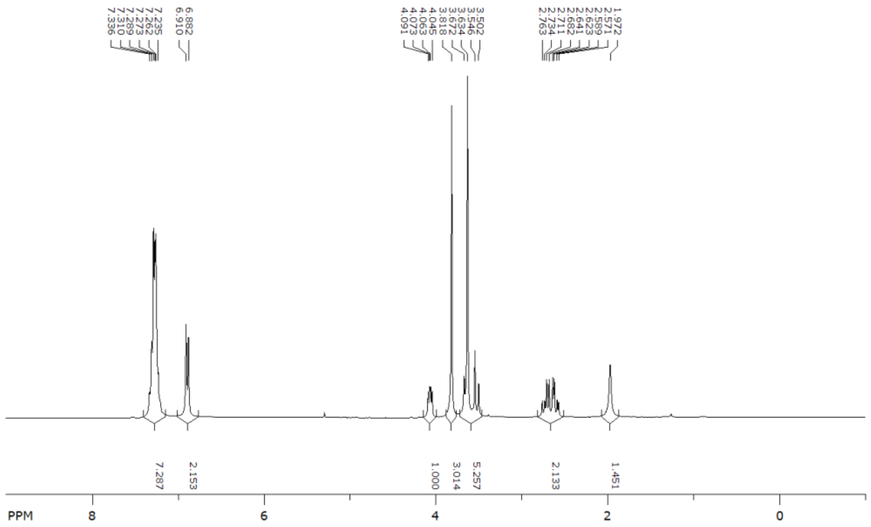

Figure S28. ^{1}H spectrum (300 MHz, $CDCl_3$) of *rac-1h.*

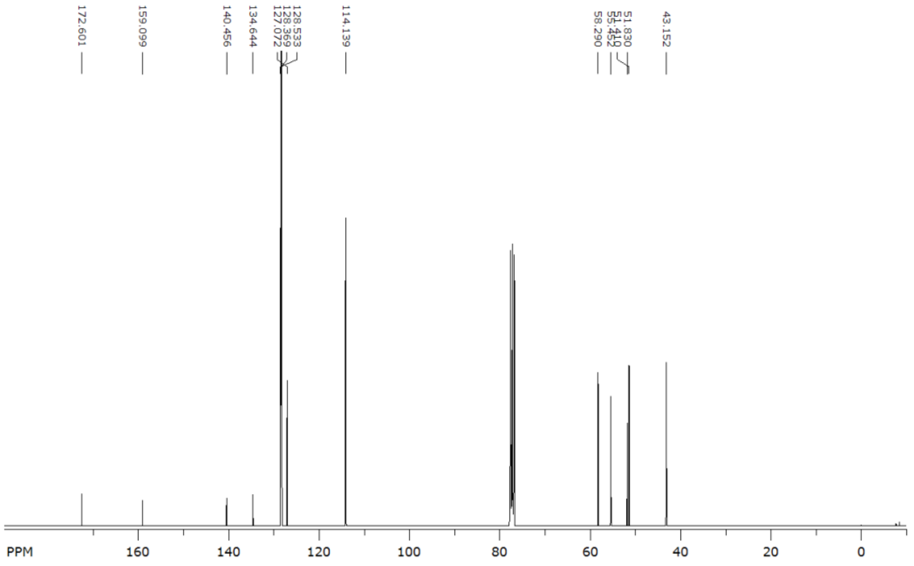

Figure S29. ^{13}C spectrum (75 MHz, $CDCl_3$) of *rac-1h.*

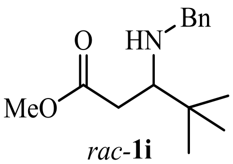

rac-**1i**

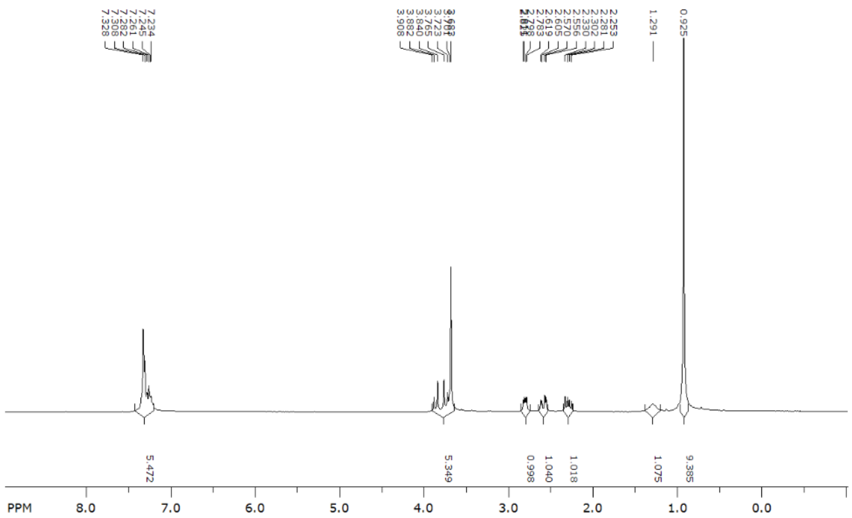

Figure S30. ^{1}H spectrum (300 MHz, $CDCl_3$) of *rac-1h.*

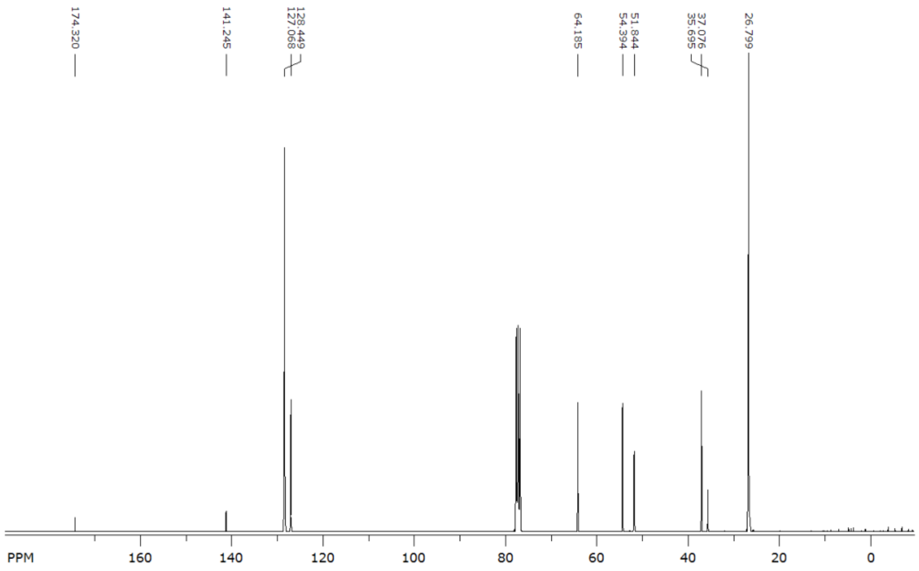

Figure S31. ^{13}C spectrum (75 MHz, $CDCl_3$) of *rac-1h.*

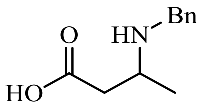

rac-**2a**

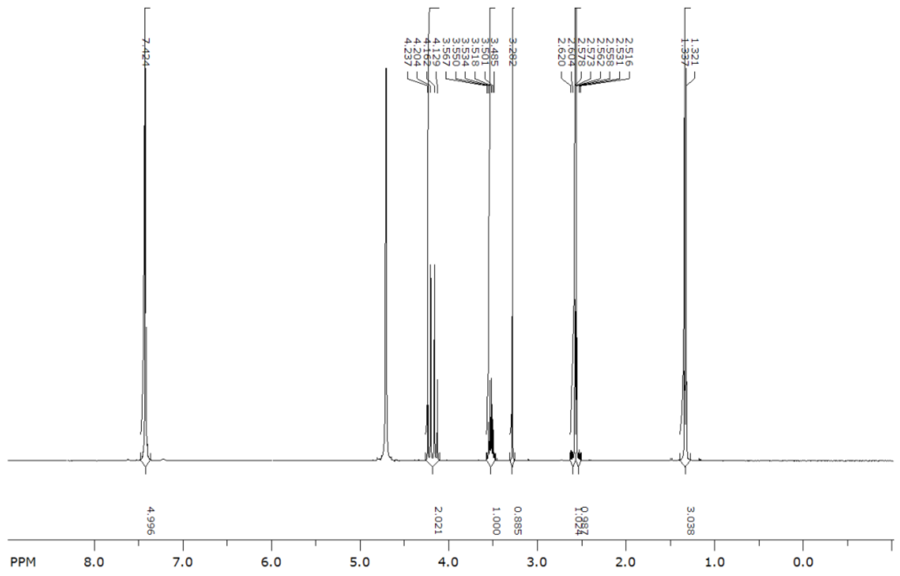

Figure S32. **^{1}H spectrum (400 MHz, D_2O) of *2a***

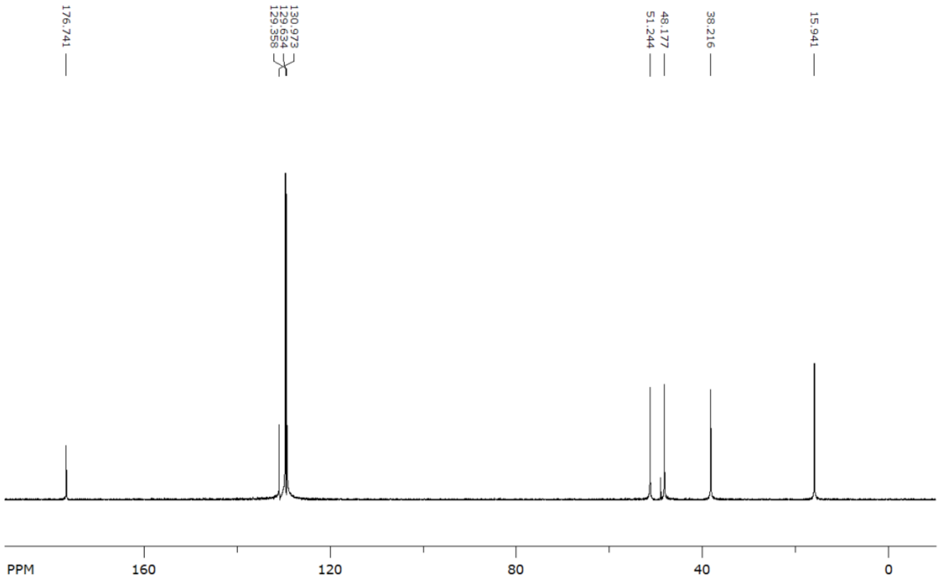

Figure S33. **^{13}C spectrum (100 MHz, D_2O) of *2a***

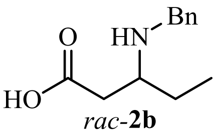

rac-**2b**

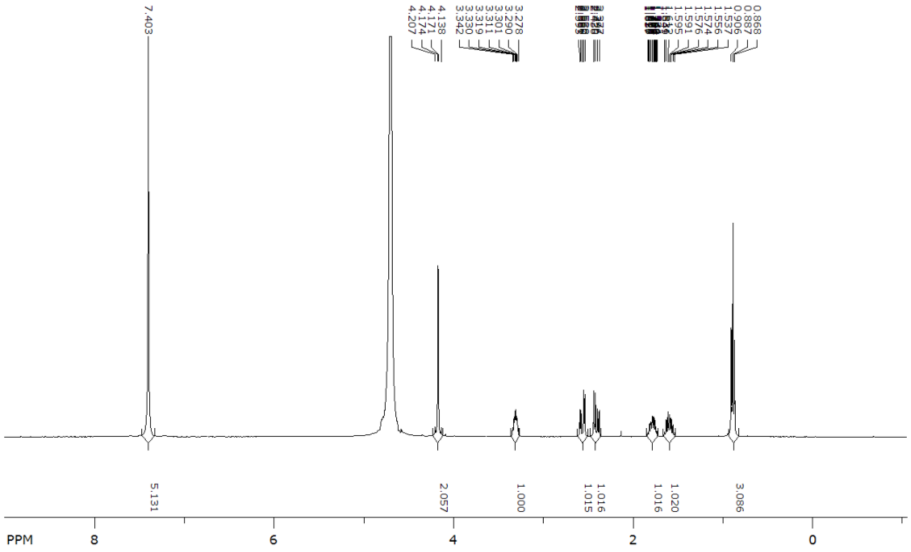

Figure S34. ^{13}C spectrum (400 MHz, D_2O) of *2b*

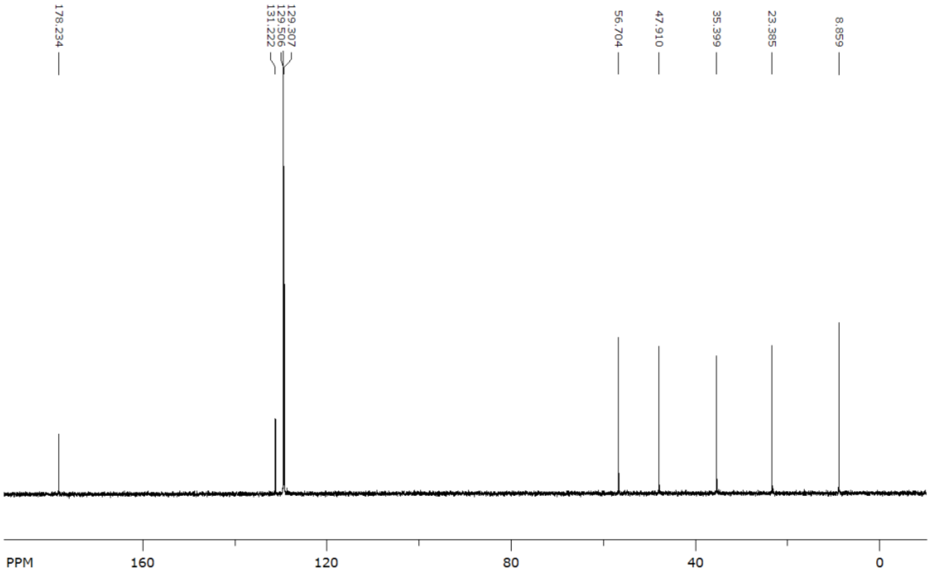

Figure S35. ^{13}C spectrum (100 MHz, D_2O) of *2b*

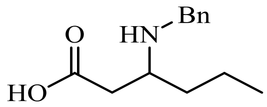

rac-**2c**

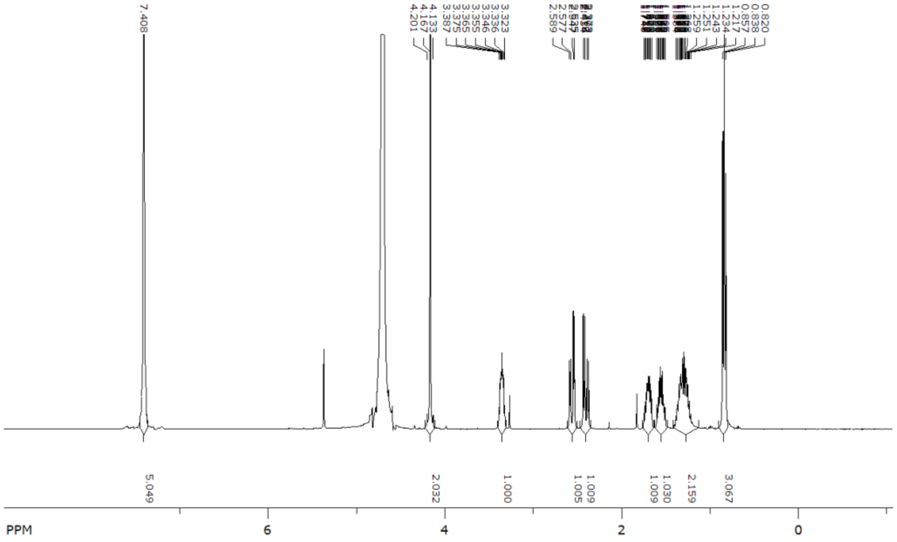

Figure S36. ^{1}H spectrum (400 MHz, D_2O) of *2c*

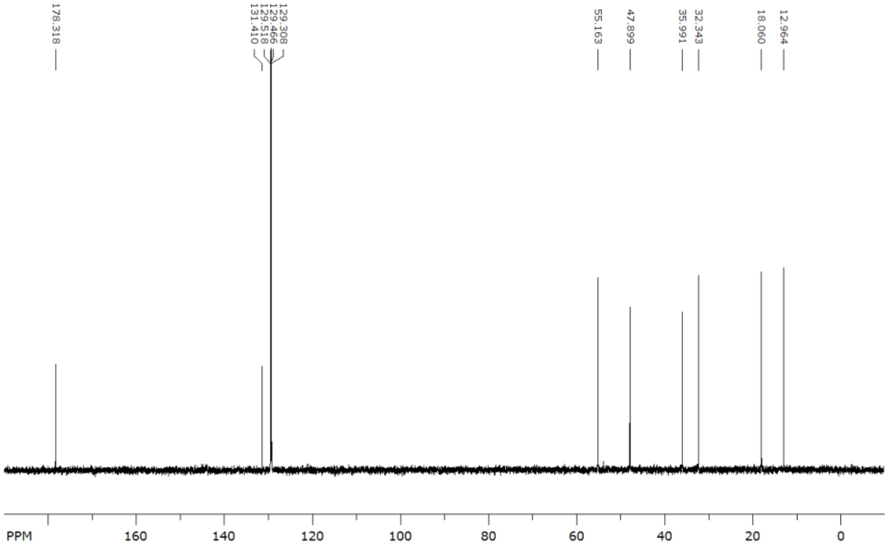

Figure S37. ^{13}C spectrum (100 MHz, D_2O) of *2c*

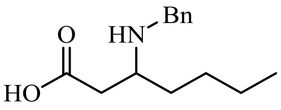

rac-**2d**

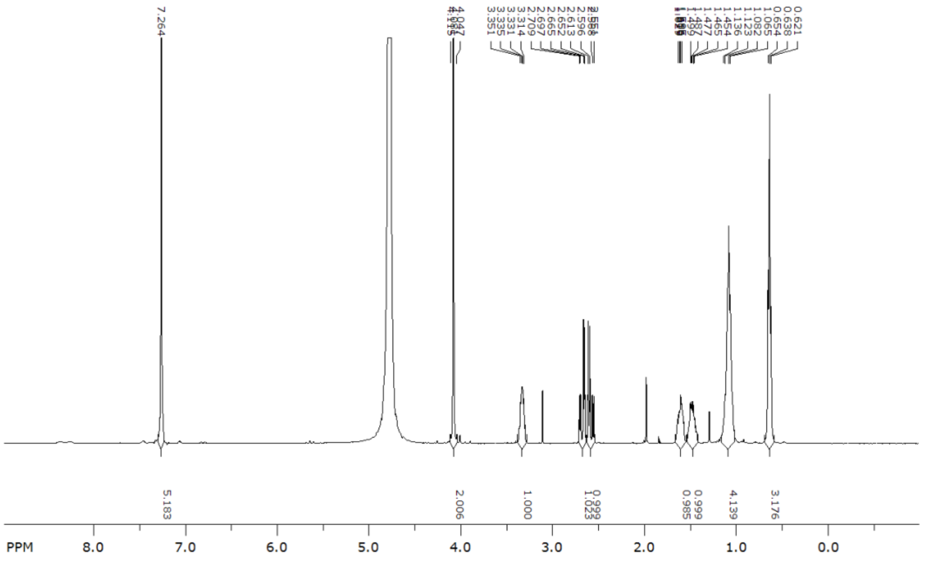

Figure S38. ^{1}H spectrum (400 MHz, D_2O) of *2d*

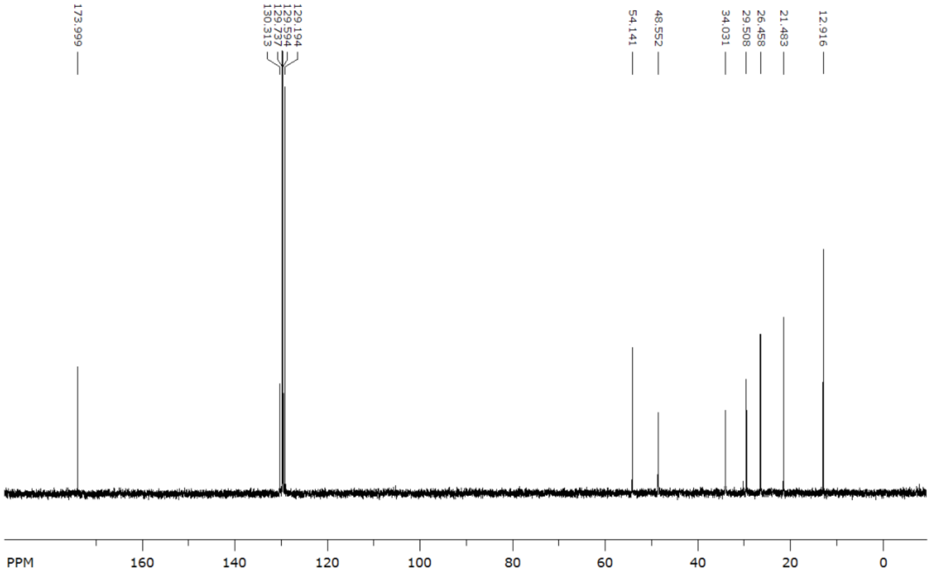

Figure S39. ^{13}C spectrum (100 MHz, D_2O) of *2d*

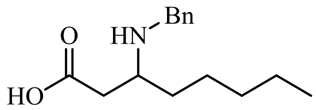

rac-**2e**

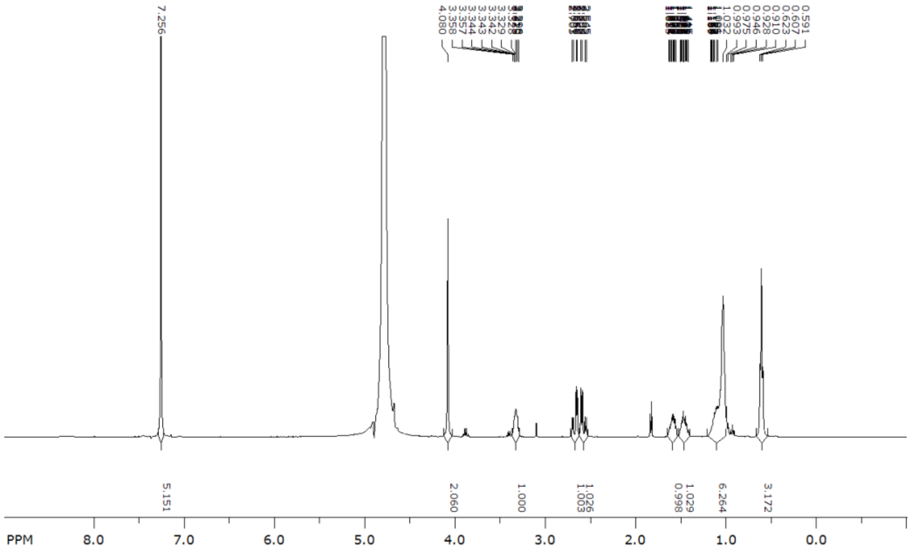

Figure S40. ^{1}H spectrum (400 MHz, D_2O) of *2e*

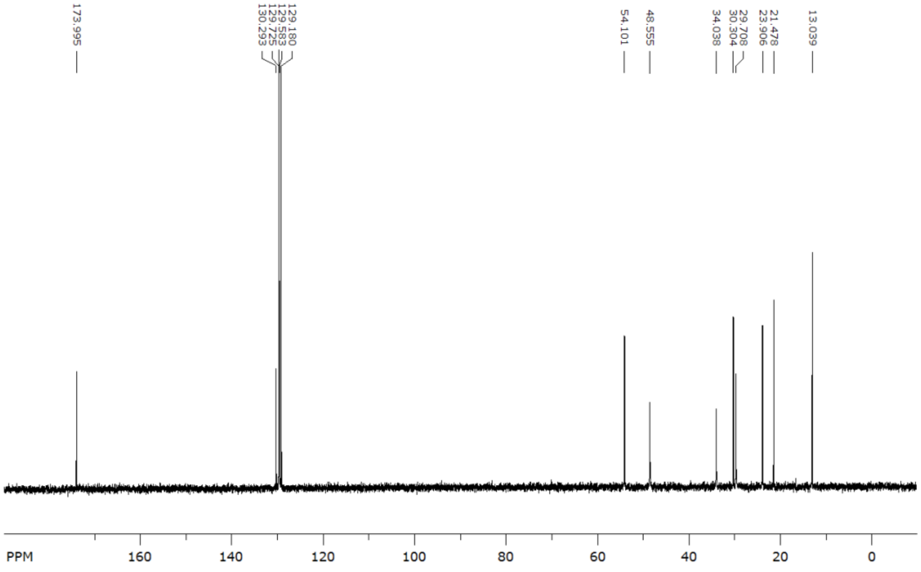

Figure S41. ^{13}C spectrum (100 MHz, D_2O) of *2e*

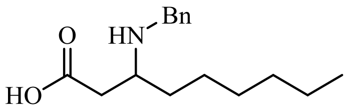

rac-**2f**

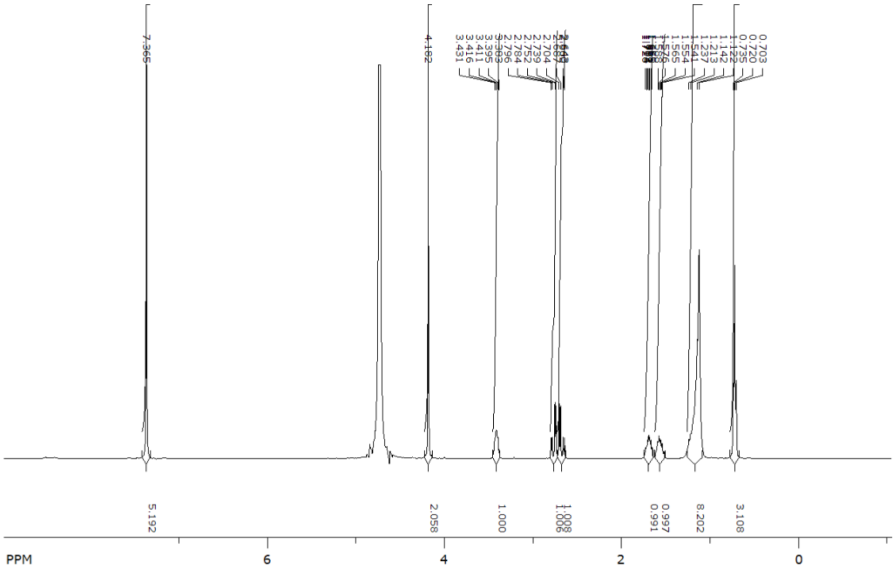

Figure S42. ^{1}H spectrum (400 MHz, D_2O) of *2f*

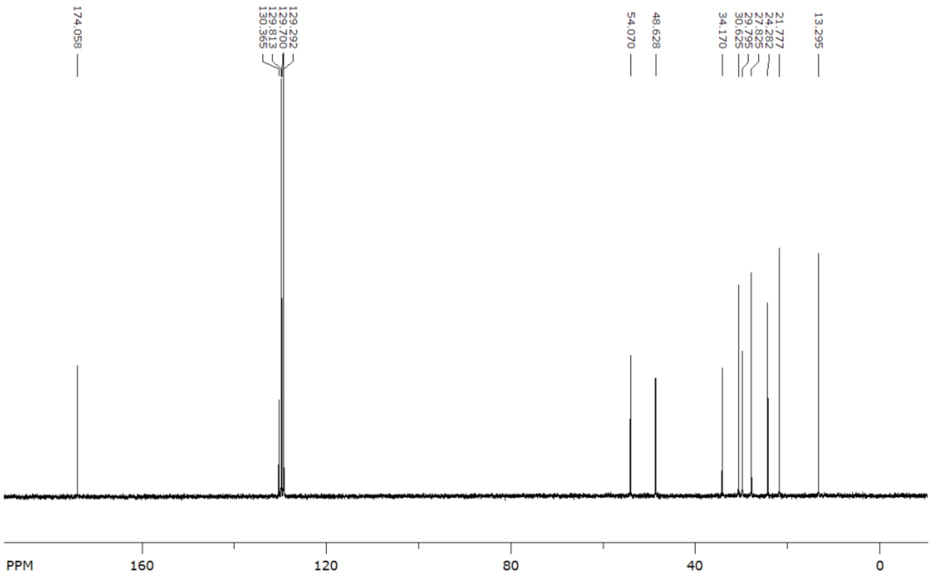

Figure S43. ^{13}C spectrum (100 MHz, D_2O) of *2f*

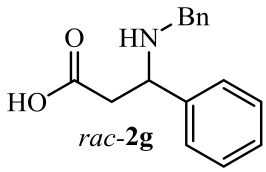

rac-**2g**

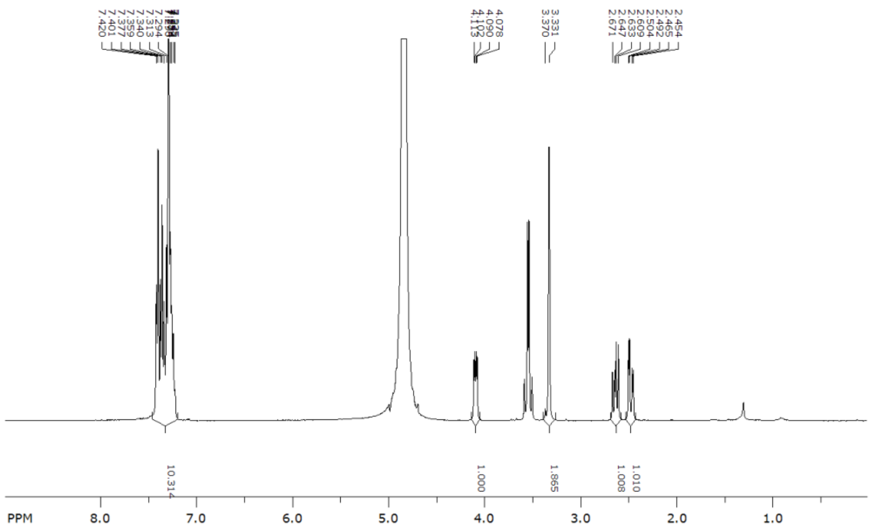

Figure S44. ^{1}H spectrum (400 MHz, CD_3OD) of *2g*

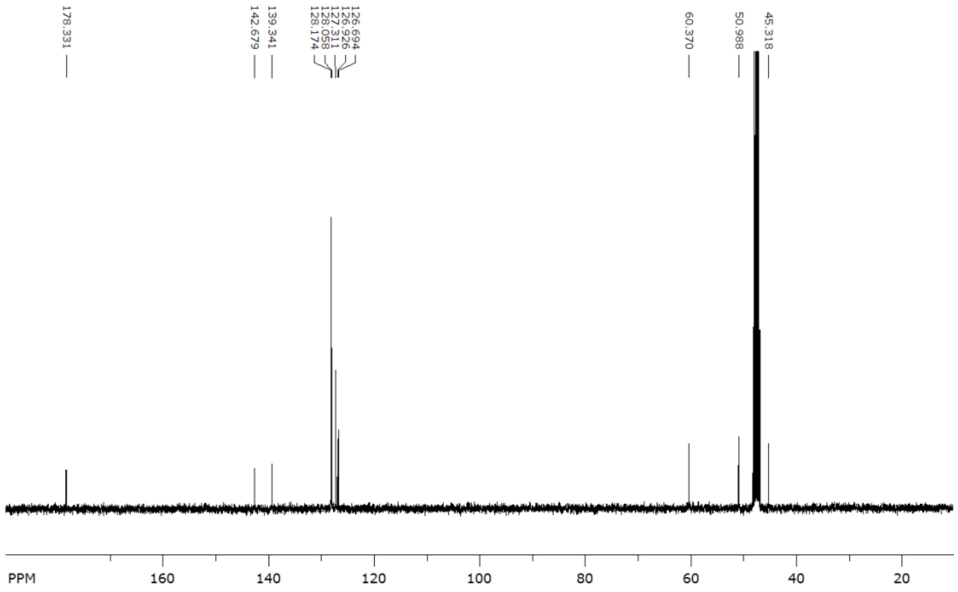

Figure S45. ^{13}C spectrum (100 MHz, CD_3OD) of *2g*

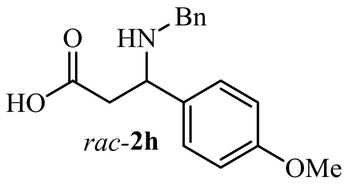

rac-**2h**

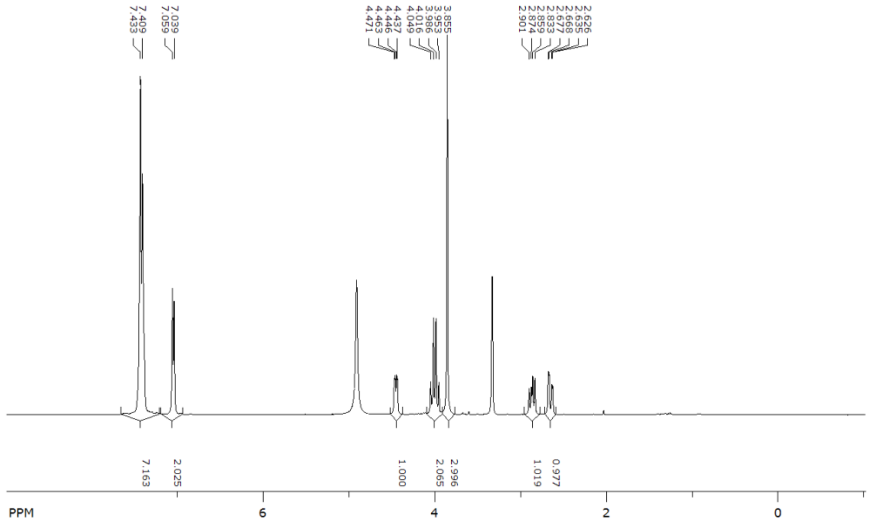

Figure S46. ^{1}H spectrum (400 MHz, CD_3OD) of *2h*

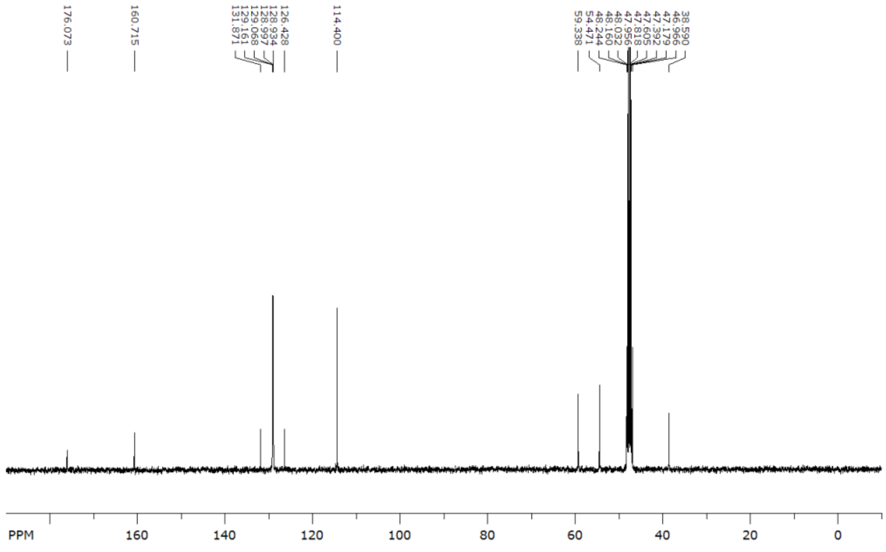

Figure S47. ^{13}C spectrum (100 MHz, CD_3OD) of *2h*

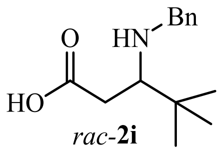

rac-**2i**

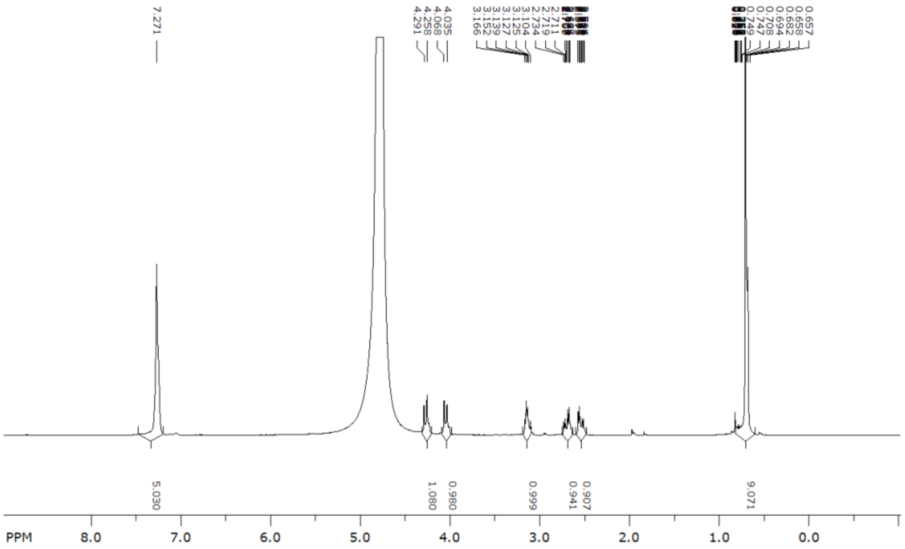

Figure S48. ^{1}H spectrum (400 MHz, D_2O) of *2i*

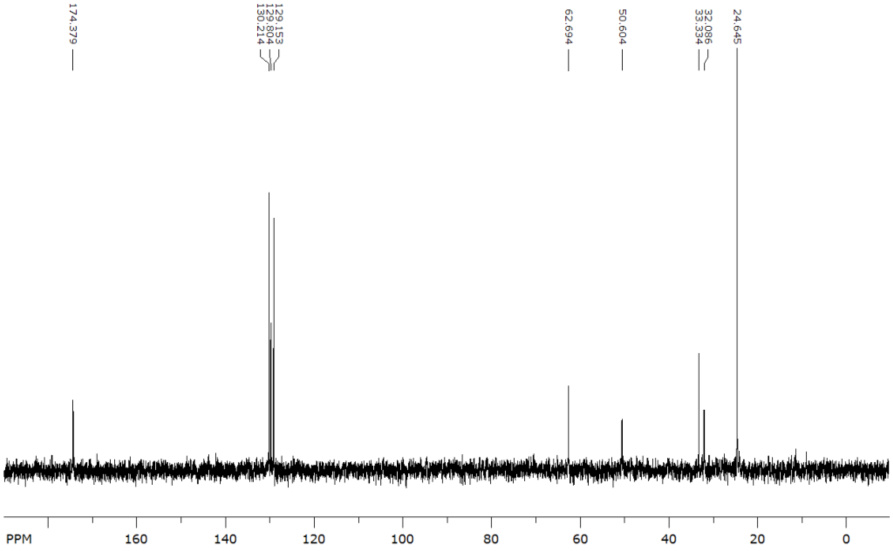

Figure S49. ^{13}C spectrum (100 MHz, D_2O) of *2i*

6) Chromatograms for the determination of enantiomeric excess of raw material and hydrolysis products.

- **Chromatograms for the search of best conditions for the enzymatic hydrolysis resolution under HSBM (Table S1).**

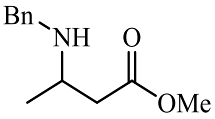

rac-**1a**

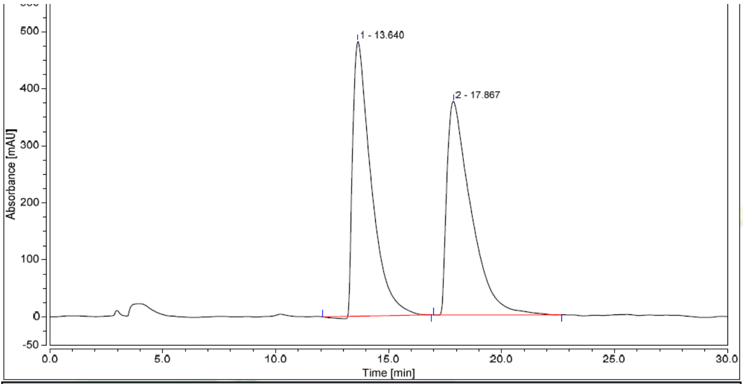

Integration Results							
No.	Peak Name	Retention Time min	Area mAU*min	Height mAU	Relative Area %	Relative Height %	Amount n.a.
1		13.640	455.884	482.090	49.63	56.27	n.a.
2		17.867	462.617	374.599	50.37	43.73	n.a.
Total:			918.501	856.689	100.00	100.00	

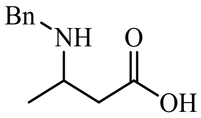

rac-**2a**

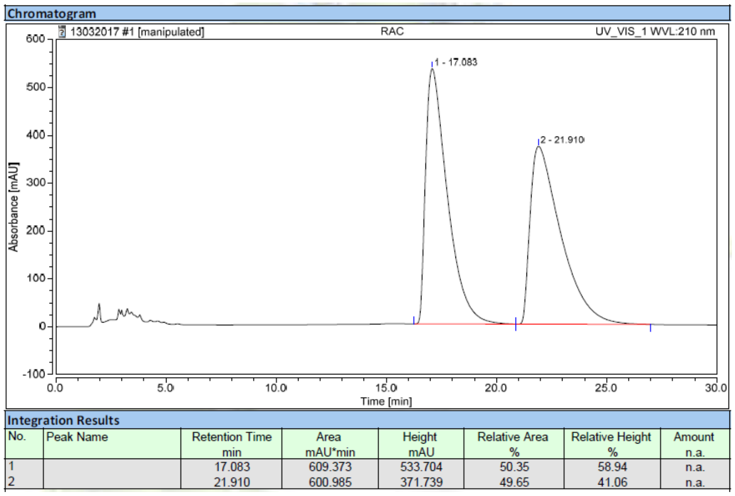

Integration Results							
No.	Peak Name	Retention Time min	Area mAU*min	Height mAU	Relative Area %	Relative Height %	Amount n.a.
1		17.083	609.373	533.704	50.35	58.94	n.a.
2		21.910	600.985	371.739	49.65	41.06	n.a.

For entry 1.

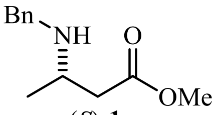

(*S*)-**1a**

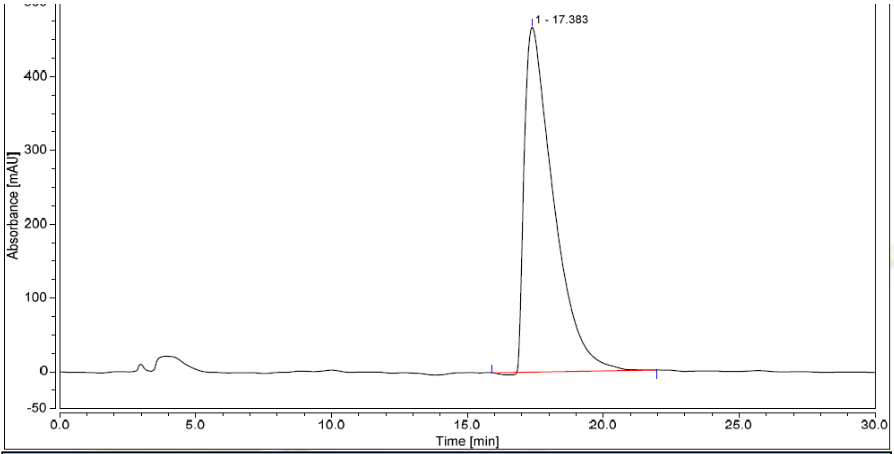

Integration Results							
No.	Peak Name	Retention Time min	Area mAU*min	Height mAU	Relative Area %	Relative Height %	Amount n.a.
1		17.383	579.249	467.640	100.00	100.00	n.a.
**Total:**			**579.249**	**467.640**	**100.00**	**100.00**	

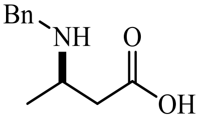

(*R*)-**2a**

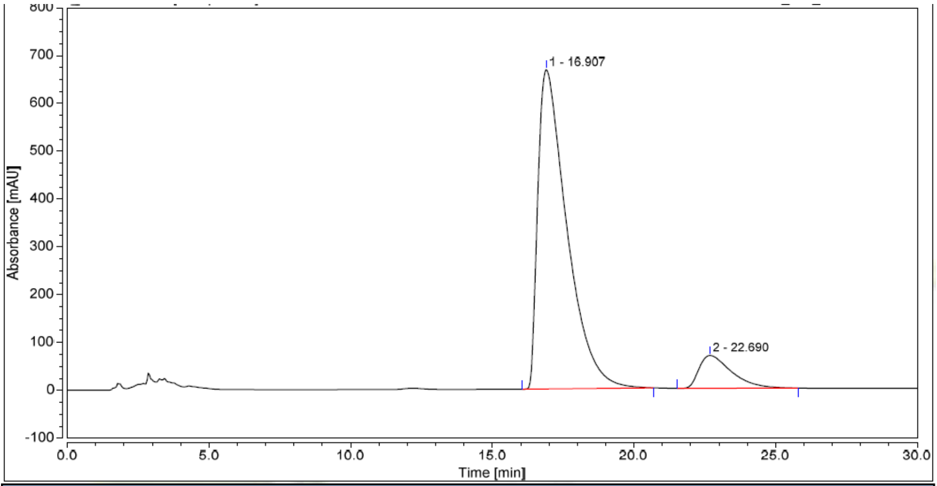

Integration Results							
No.	Peak Name	Retention Time min	Area mAU*min	Height mAU	Relative Area %	Relative Height %	Amount n.a.
1		16.907	792.244	668.044	89.68	90.67	n.a.
2		22.690	91.200	68.721	10.32	9.33	n.a.
**Total:**			**883.444**	**736.764**	**100.00**	**100.00**	

For entry 2.

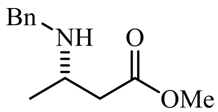

(*S*)-**1a**

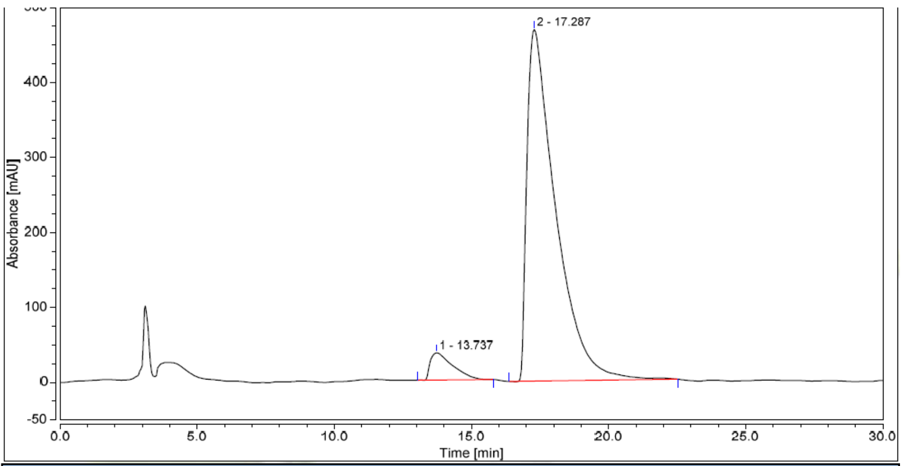

Integration Results							
No.	Peak Name	Retention Time min	Area mAU*min	Height mAU	Relative Area %	Relative Height %	Amount n.a.
1		13.737	34.071	36.090	5.65	7.15	n.a.
2		17.287	569.206	468.866	94.35	92.85	n.a.
**Total:**			**603.276**	**504.957**	**100.00**	**100.00**	

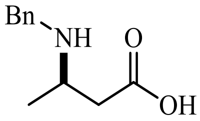

(*R*)-**2a**

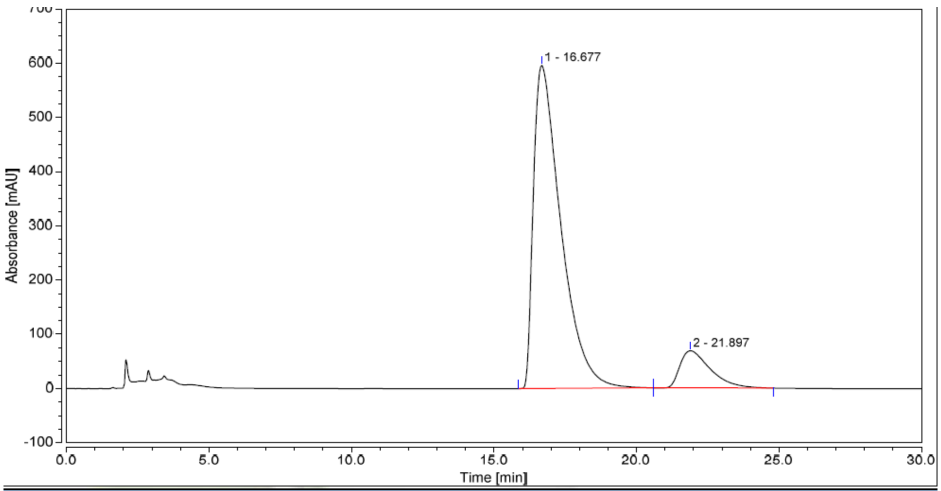

Integration Results							
No.	Peak Name	Retention Time min	Area mAU*min	Height mAU	Relative Area %	Relative Height %	Amount n.a.
1		16.677	664.848	596.004	88.71	89.70	n.a.
2		21.897	84.597	68.427	11.29	10.30	n.a.
**Total:**			**749.446**	**664.430**	**100.00**	**100.00**	

For entry 3.

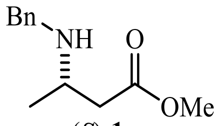

(*S*)-**1a**

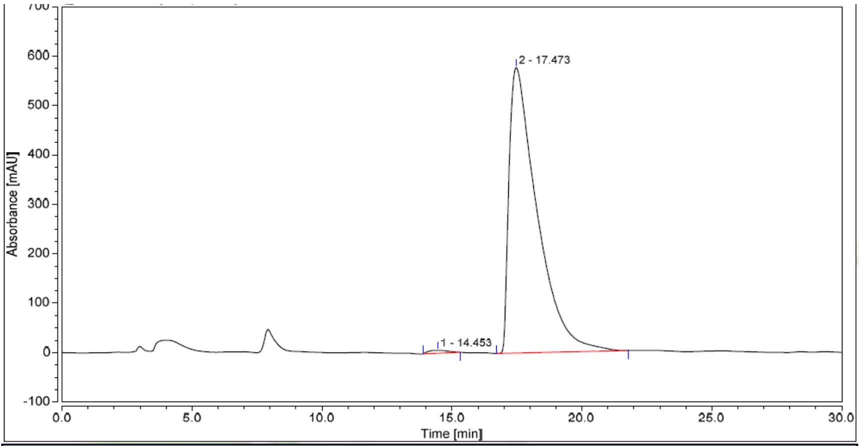

Integration Results							
No.	Peak Name	Retention Time min	Area mAU*min	Height mAU	Relative Area %	Relative Height %	Amount n.a.
1		14.453	5.103	5.936	0.70	1.02	n.a.
2		17.473	721.353	578.212	99.30	98.98	n.a.
Total:			726.456	584.148	100.00	100.00	

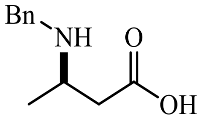

(*R*)-**2a**

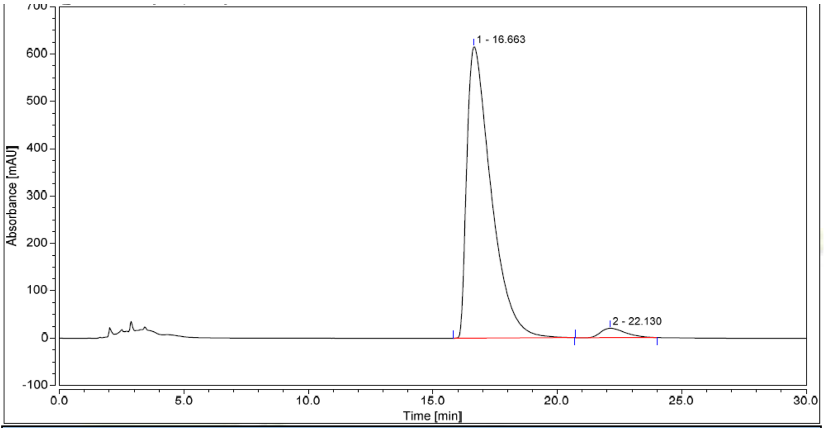

Integration Results							
No.	Peak Name	Retention Time min	Area mAU*min	Height mAU	Relative Area %	Relative Height %	Amount n.a.
1		16.663	693.709	615.390	96.91	96.90	n.a.
2		22.130	22.121	19.698	3.09	3.10	n.a.
Total:			715.830	635.088	100.00	100.00	

For entry 4.

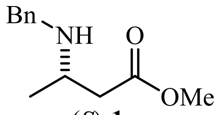

(*S*)-**1a**

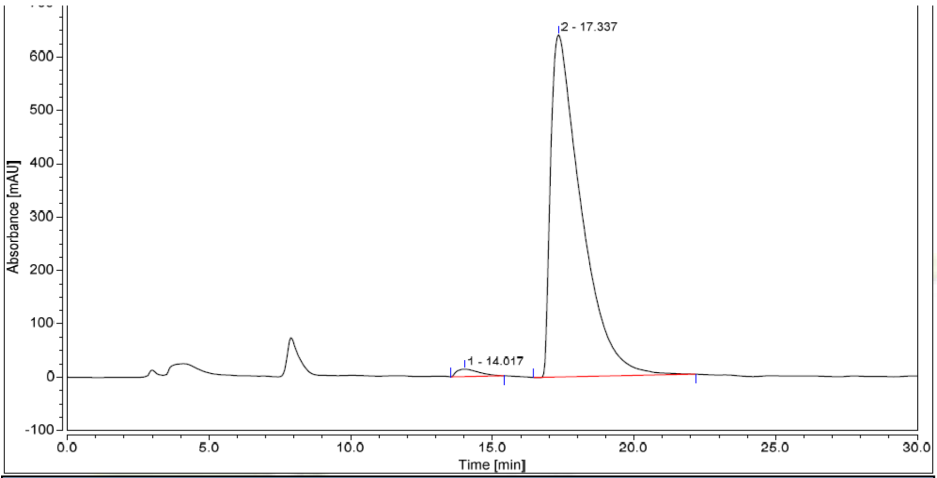

Integration Results							
No.	Peak Name	Retention Time min	Area mAU*min	Height mAU	Relative Area %	Relative Height %	Amount n.a.
1		14.017	12.412	13.802	1.53	2.11	n.a.
2		17.337	799.088	641.507	98.47	97.89	n.a.
Total:			811.499	655.309	100.00	100.00	

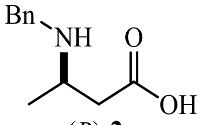

(*R*)-**2a**

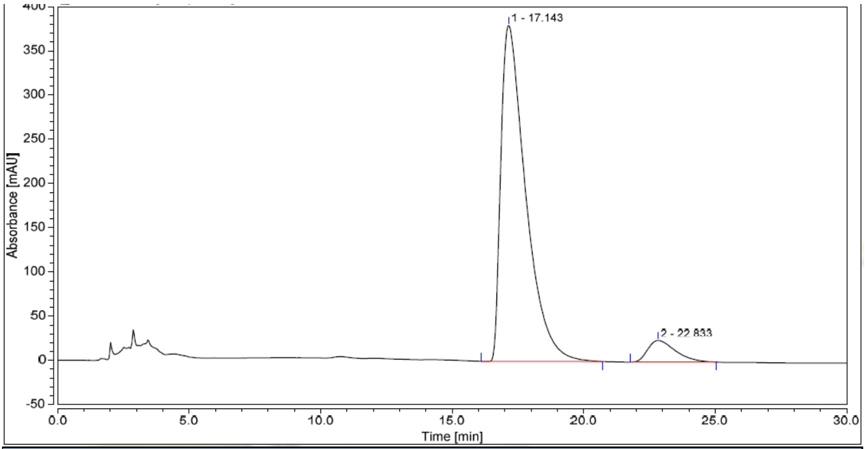

Integration Results							
No.	Peak Name	Retention Time min	Area mAU*min	Height mAU	Relative Area %	Relative Height %	Amount n.a.
1		17.143	415.324	380.072	93.22	93.94	n.a.
2		22.833	30.207	24.535	6.78	6.06	n.a.
Total:			445.531	404.607	100.00	100.00	

For entry 5.

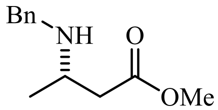

(*S*)-**1a**

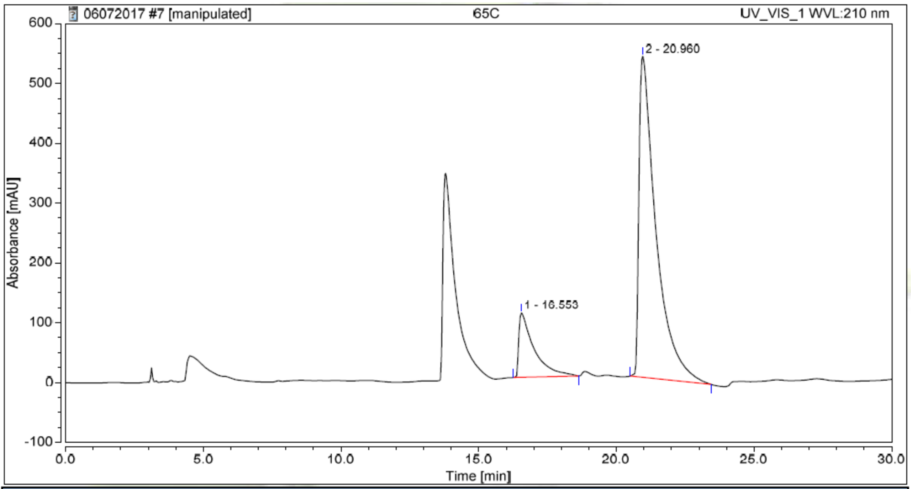

Integration Results							
No.	Peak Name	Retention Time min	Area mAU*min	Height mAU	Relative Area %	Relative Height %	Amount n.a.
1		16.553	69.302	107.805	15.35	16.73	n.a.
2		20.960	382.116	536.487	84.65	83.27	n.a.
Total:			451.419	644.292	100.00	100.00	

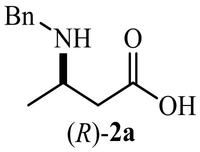

(*R*)-**2a**

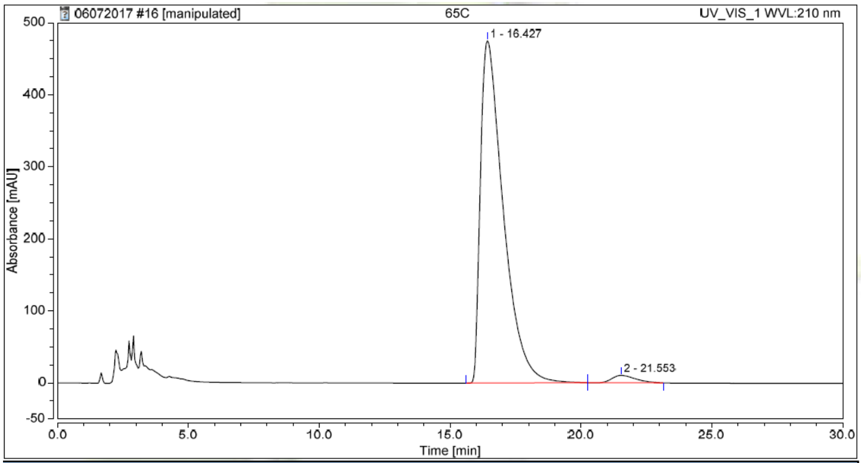

Integration Results							
No.	Peak Name	Retention Time min	Area mAU*min	Height mAU	Relative Area %	Relative Height %	Amount n.a.
1		16.427	480.875	475.227	97.88	97.91	n.a.
2		21.553	10.407	10.129	2.12	2.09	n.a.
Total:			491.282	485.356	100.00	100.00	

For entry 6.

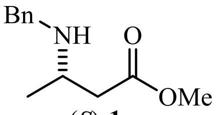

(*S*)-**1a**

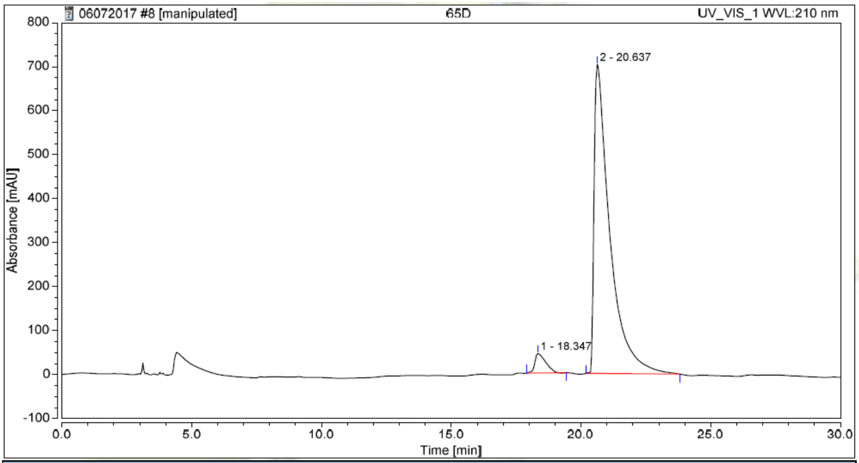

Integration Results							
No.	Peak Name	Retention Time min	Area mAU*min	Height mAU	Relative Area %	Relative Height %	Amount n.a.
1		18.347	21.147	43.883	4.11	5.88	n.a.
2		20.637	493.304	702.389	95.89	94.12	n.a.
**Total:**			**514.451**	**746.271**	**100.00**	**100.00**	

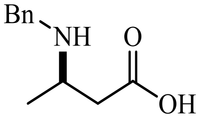

(*R*)-**2a**

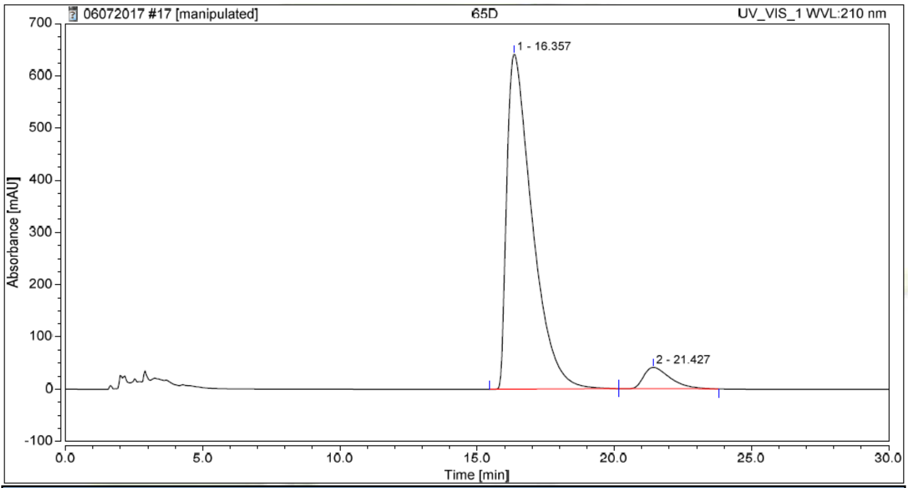

Integration Results							
No.	Peak Name	Retention Time min	Area mAU*min	Height mAU	Relative Area %	Relative Height %	Amount n.a.
1		16.357	686.102	641.786	93.79	94.02	n.a.
2		21.427	45.409	40.830	6.21	5.98	n.a.
**Total:**			**731.510**	**682.616**	**100.00**	**100.00**	

For entry 7.

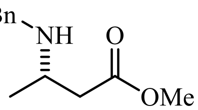

(*S*)-**1a**

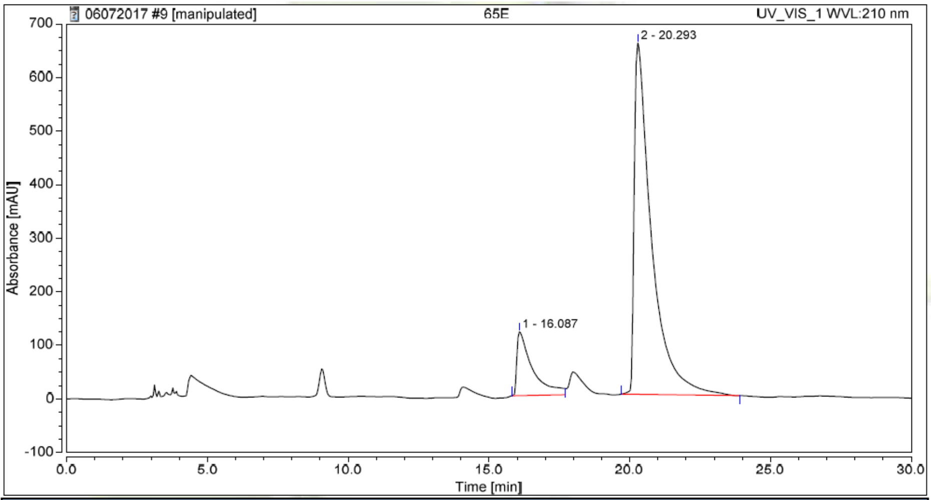

Integration Results							
No.	Peak Name	Retention Time min	Area mAU*min	Height mAU	Relative Area %	Relative Height %	Amount n.a.
1		16.087	76.225	119.348	14.04	15.39	n.a.
2		20.293	466.522	656.049	85.96	84.61	n.a.
Total:			542.746	775.398	100.00	100.00	

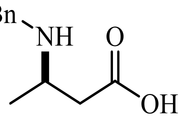

(*R*)-**2a**

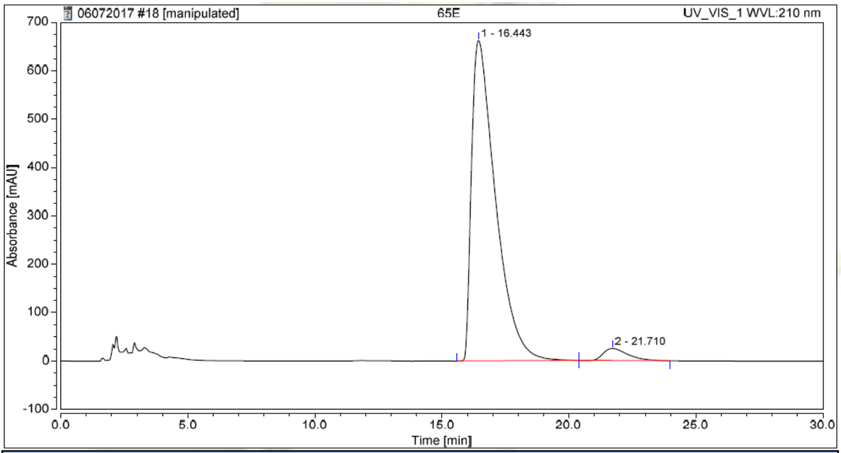

Integration Results							
No.	Peak Name	Retention Time min	Area mAU*min	Height mAU	Relative Area %	Relative Height %	Amount n.a.
1		16.443	724.223	662.647	96.31	96.36	n.a.
2		21.710	27.724	25.002	3.69	3.64	n.a.
Total:			751.947	687.648	100.00	100.00	

For entry 8.

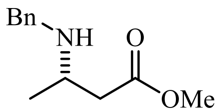

(*S*)-**1a**

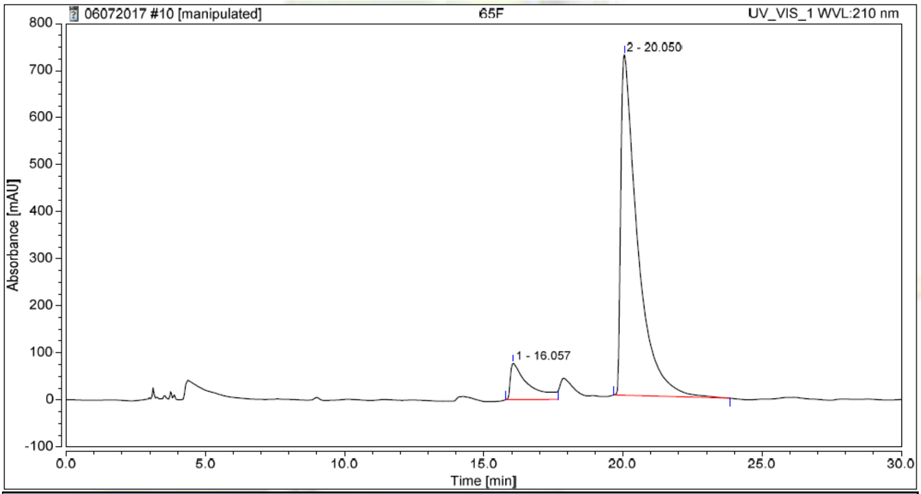

Integration Results							
No.	Peak Name	Retention Time min	Area mAU*min	Height mAU	Relative Area %	Relative Height %	Amount n.a.
1		16.057	57.959	76.901	10.32	9.60	n.a.
2		20.050	503.457	724.282	89.68	90.40	n.a.
**Total:**			**561.415**	**801.182**	**100.00**	**100.00**	

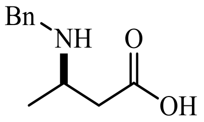

(*R*)-**2a**

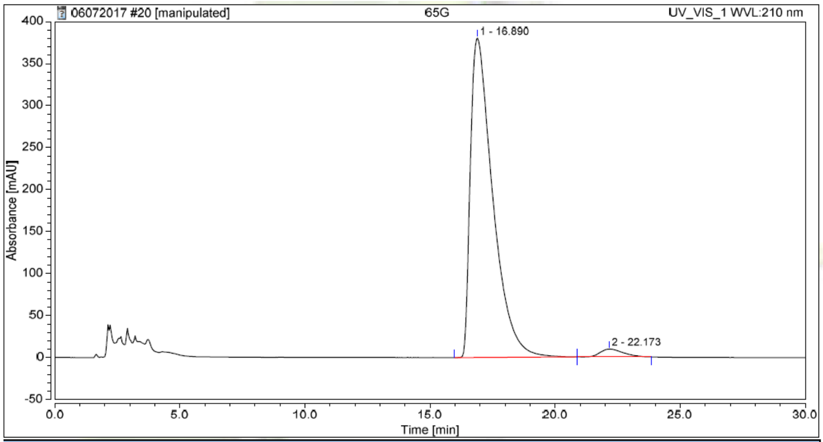

Integration Results							
No.	Peak Name	Retention Time min	Area mAU*min	Height mAU	Relative Area %	Relative Height %	Amount n.a.
1		16.890	394.642	380.555	97.51	97.60	n.a.
2		22.173	10.092	9.368	2.49	2.40	n.a.
**Total:**			**404.734**	**389.923**	**100.00**	**100.00**	

For entry 9.

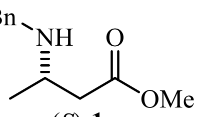

(*S*)-**1a**

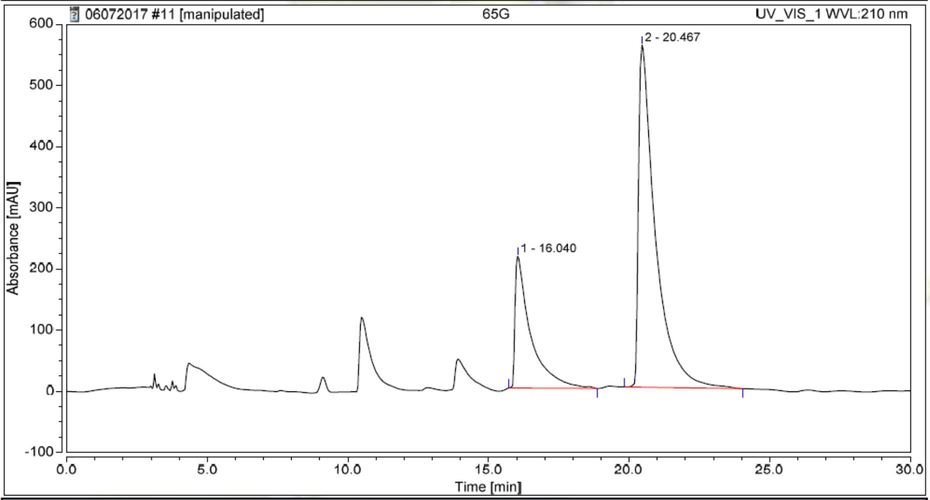

Integration Results							
No.	Peak Name	Retention Time min	Area mAU*min	Height mAU	Relative Area %	Relative Height %	Amount n.a.
1		16.040	137.783	215.602	26.12	27.84	n.a.
2		20.467	389.679	558.936	73.88	72.16	n.a.
**Total:**			**527.463**	**774.538**	**100.00**	**100.00**	

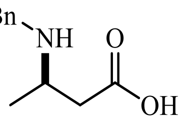

(*R*)-**2a**

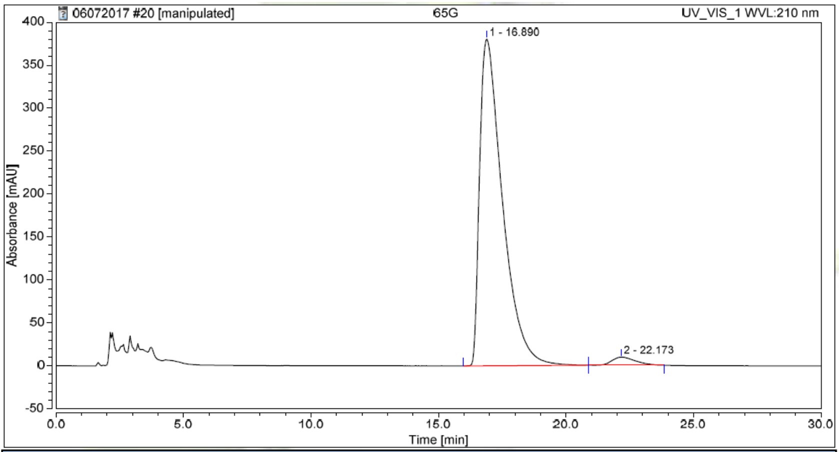

Integration Results							
No.	Peak Name	Retention Time min	Area mAU*min	Height mAU	Relative Area %	Relative Height %	Amount n.a.
1		16.890	394.642	380.555	97.51	97.60	n.a.
2		22.173	10.092	9.368	2.49	2.40	n.a.
**Total:**			**404.734**	**389.923**	**100.00**	**100.00**	

For entry 10.

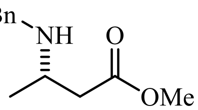

(*S*)-**1a**

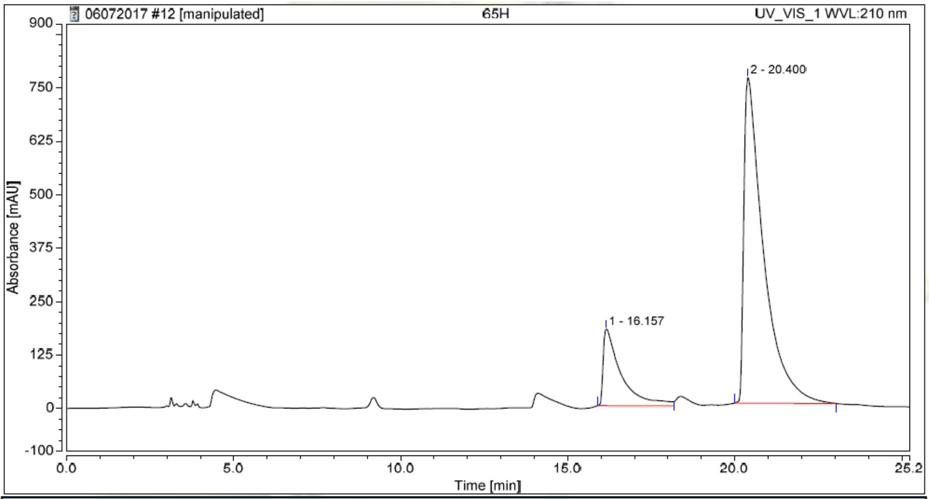

Integration Results							
No.	Peak Name	Retention Time min	Area mAU*min	Height mAU	Relative Area %	Relative Height %	Amount n.a.
1		16.157	111.694	179.934	17.66	19.10	n.a.
2		20.400	520.628	761.956	82.34	80.90	n.a.
**Total:**			**632.323**	**941.890**	**100.00**	**100.00**	

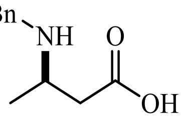

(*R*)-**2a**

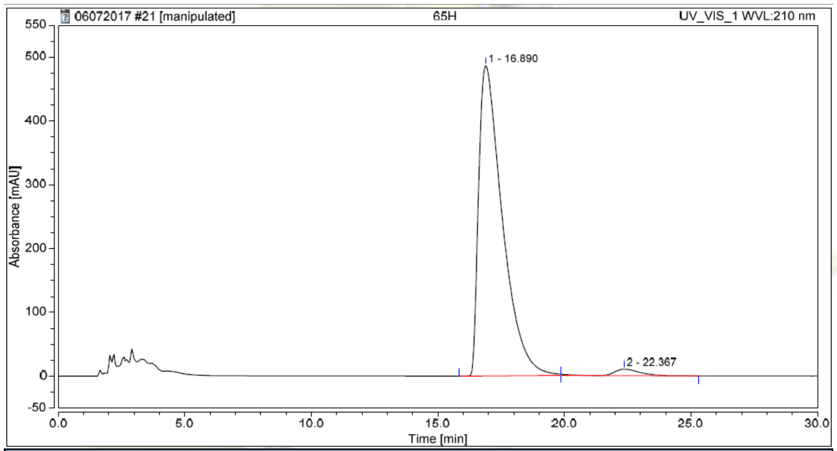

Integration Results							
No.	Peak Name	Retention Time min	Area mAU*min	Height mAU	Relative Area %	Relative Height %	Amount n.a.
1		16.890	527.601	486.451	97.68	97.94	n.a.
2		22.367	12.514	10.218	2.32	2.06	n.a.
**Total:**			**540.114**	**496.669**	**100.00**	**100.00**	

For entry 11.

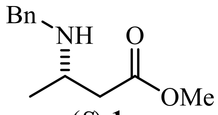

(*S*)-**1a**

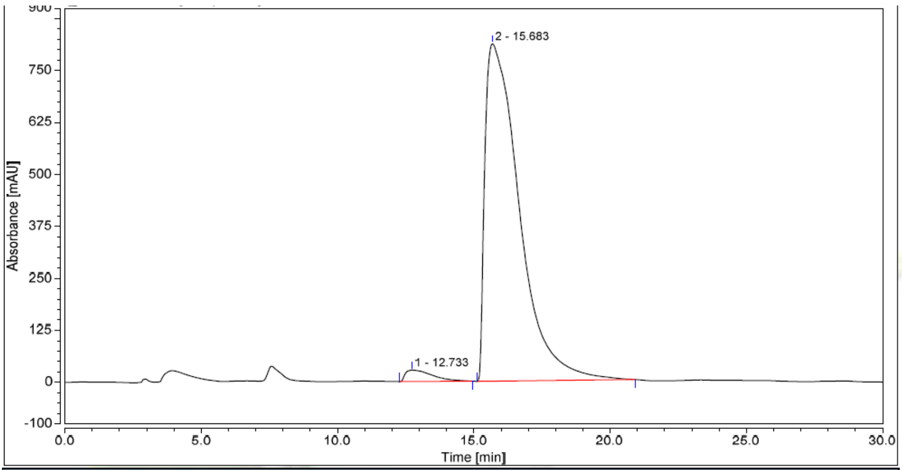

Integration Results

No.	Peak Name	Retention Time min	Area mAU*min	Height mAU	Relative Area %	Relative Height %	Amount n.a.
1		12.733	31.315	27.053	2.57	3.22	n.a.
2		15.683	1185.288	811.859	97.43	96.78	n.a.
**Total:**			**1216.603**	**838.912**	**100.00**	**100.00**	

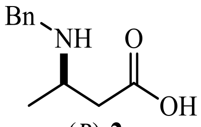

(*R*)-**2a**

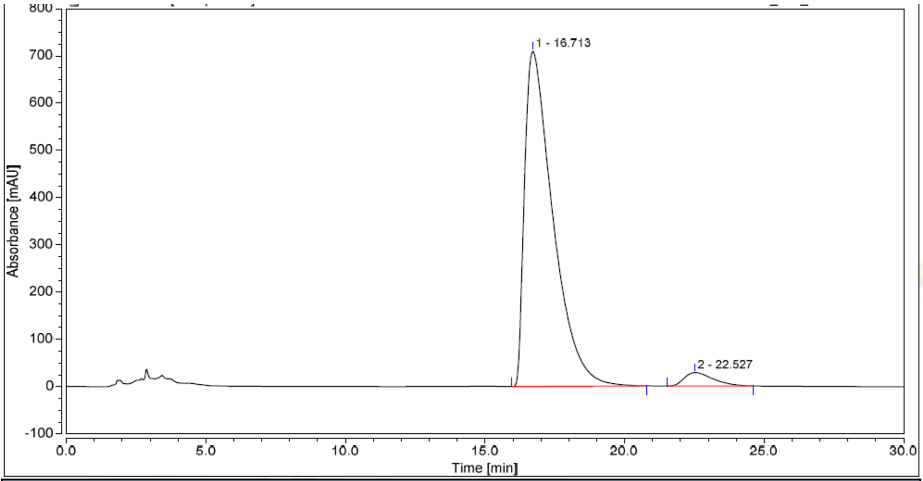

Integration Results

No.	Peak Name	Retention Time min	Area mAU*min	Height mAU	Relative Area %	Relative Height %	Amount n.a.
1		16.713	834.034	710.080	96.02	96.11	n.a.
2		22.527	34.529	28.721	3.98	3.89	n.a.
**Total:**			**868.563**	**738.800**	**100.00**	**100.00**	

For entry 12.

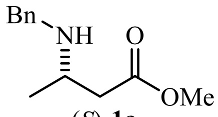

(*S*)-**1a**

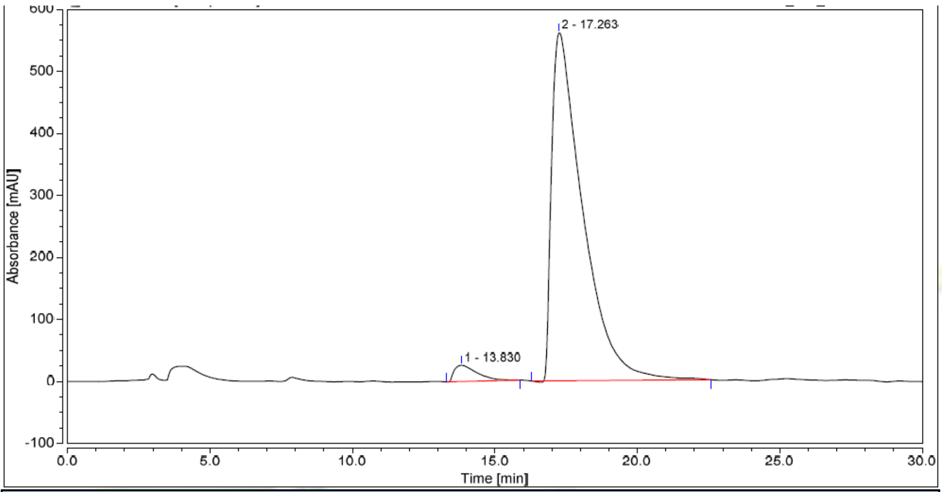

Integration Results							
No.	Peak Name	Retention Time min	Area mAU*min	Height mAU	Relative Area %	Relative Height %	Amount n.a.
1		13.830	24.251	26.194	3.31	4.46	n.a.
2		17.263	708.473	561.512	96.69	95.54	n.a.
**Total:**			**732.724**	**587.706**	**100.00**	**100.00**	

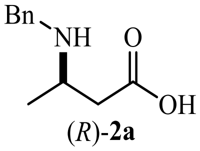

(*R*)-**2a**

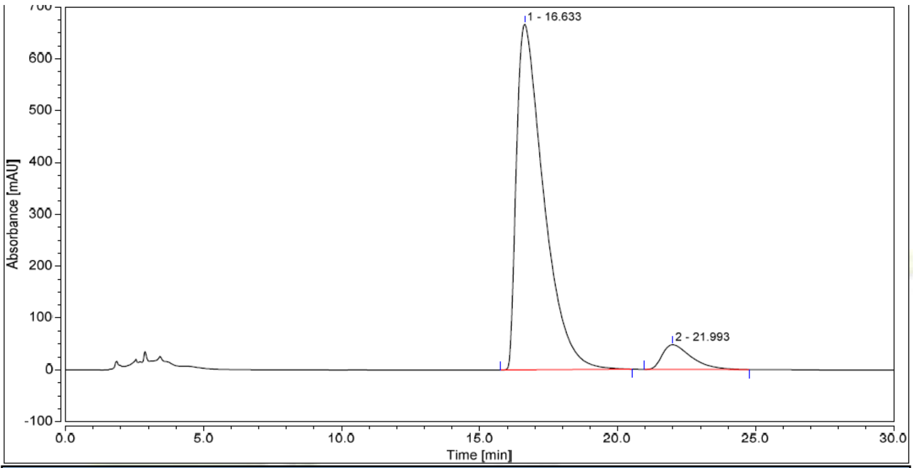

Integration Results							
No.	Peak Name	Retention Time min	Area mAU*min	Height mAU	Relative Area %	Relative Height %	Amount n.a.
1		16.633	759.988	666.344	92.92	93.34	n.a.
2		21.993	57.872	47.564	7.08	6.66	n.a.
**Total:**			**817.860**	**713.909**	**100.00**	**100.00**	

For entry 13.

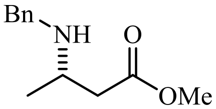

(*S*)-**1a**

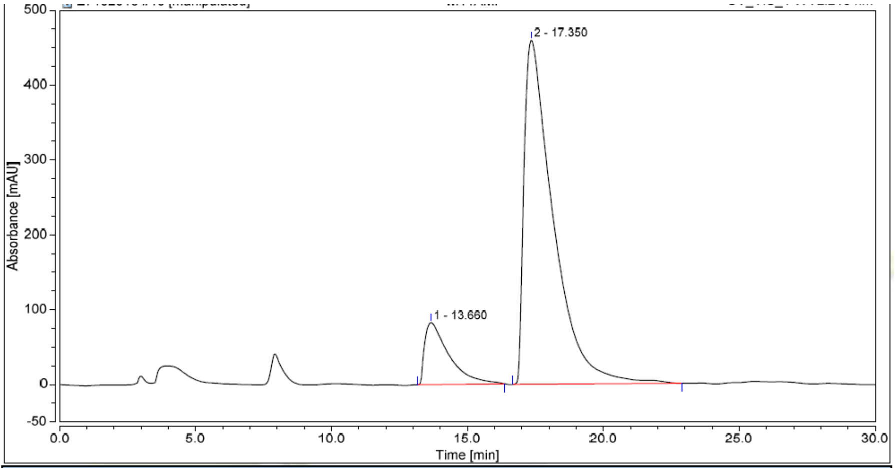

Integration Results							
No.	Peak Name	Retention Time min	Area mAU*min	Height mAU	Relative Area %	Relative Height %	Amount n.a.
1		13.660	85.810	82.777	13.05	15.25	n.a.
2		17.350	571.918	459.992	86.95	84.75	n.a.
Total:			657.728	542.768	100.00	100.00	

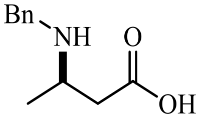

(*R*)-**2a**

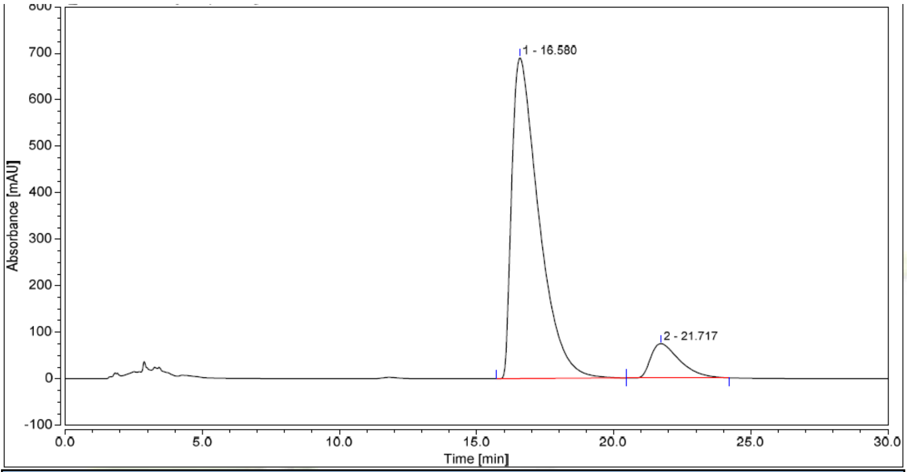

Integration Results							
No.	Peak Name	Retention Time min	Area mAU*min	Height mAU	Relative Area %	Relative Height %	Amount n.a.
1		16.580	787.488	690.073	89.77	90.35	n.a.
2		21.717	89.760	73.686	10.23	9.65	n.a.
Total:			877.247	763.759	100.00	100.00	

- **Chromatograms for substrate scope for the enzymatic resolution of N-benzylated-β^3-amino acid methyl esters (Table S2)**

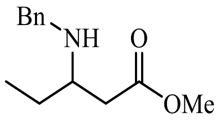

rac-**1b**

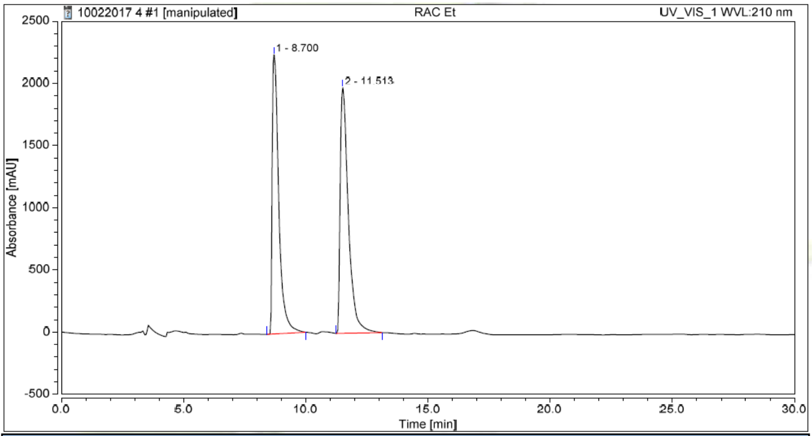

Integration Results

No.	Peak Name	Retention Time min	Area mAU*min	Height mAU	Relative Area %	Relative Height %	Amount n.a.
1		8.700	702.758	2245.976	47.86	53.19	n.a.
2		11.513	765.462	1976.201	52.14	46.81	n.a.
Total:			1468.220	4222.176	100.00	100.00	

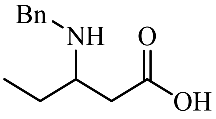

rac-**2b**

Chromatogram

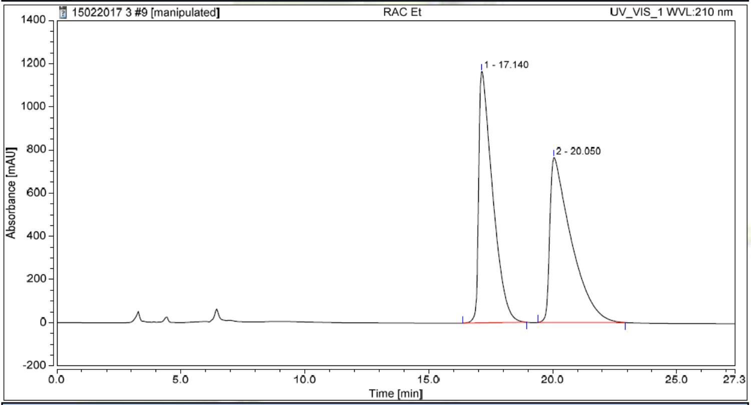

Integration Results

No.	Peak Name	Retention Time min	Area mAU*min	Height mAU	Relative Area %	Relative Height %	Amount n.a.
1		17.140	760.046	1165.262	49.83	60.36	n.a.
2		20.050	765.325	765.205	50.17	39.64	n.a.
Total:			1525.371	1930.467	100.00	100.00	

For entry 1.

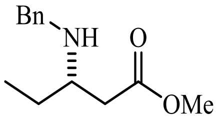

(*S*)-**1b**

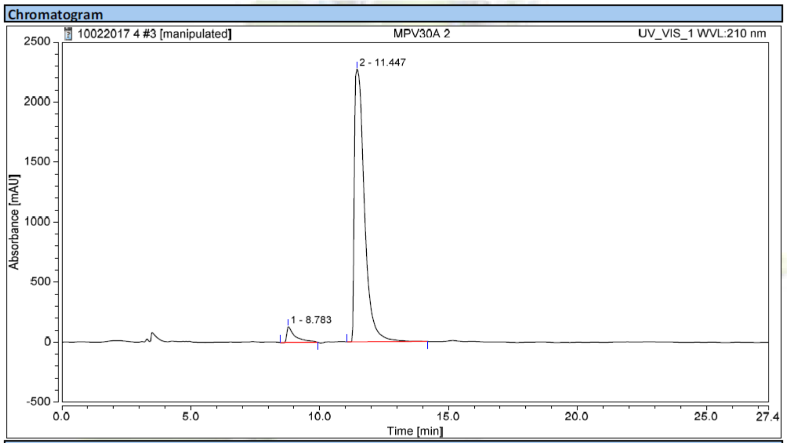

Integration Results

No.	Peak Name	Retention Time min	Area mAU*min	Height mAU	Relative Area %	Relative Height %	Amount n.a.
1		8.783	49.842	133.253	4.63	5.54	n.a.
2		11.447	1026.376	2273.509	95.37	94.46	n.a.
**Total:**			**1076.218**	**2406.762**	**100.00**	**100.00**	

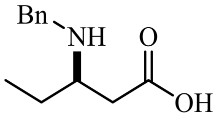

(*R*)-**2b**

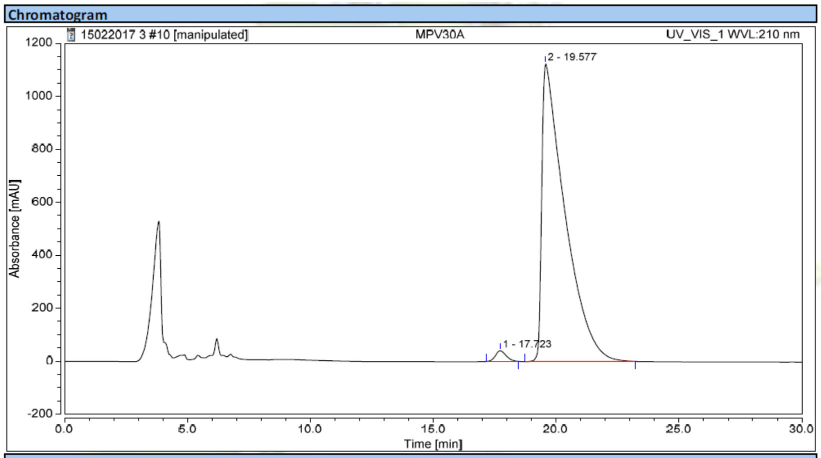

Integration Results

No.	Peak Name	Retention Time min	Area mAU*min	Height mAU	Relative Area %	Relative Height %	Amount n.a.
1		17.723	20.051	40.113	1.61	3.45	n.a.
2		19.577	1222.183	1124.148	98.39	96.55	n.a.
**Total:**			**1242.234**	**1164.262**	**100.00**	**100.00**	

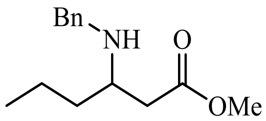

rac-**1c**

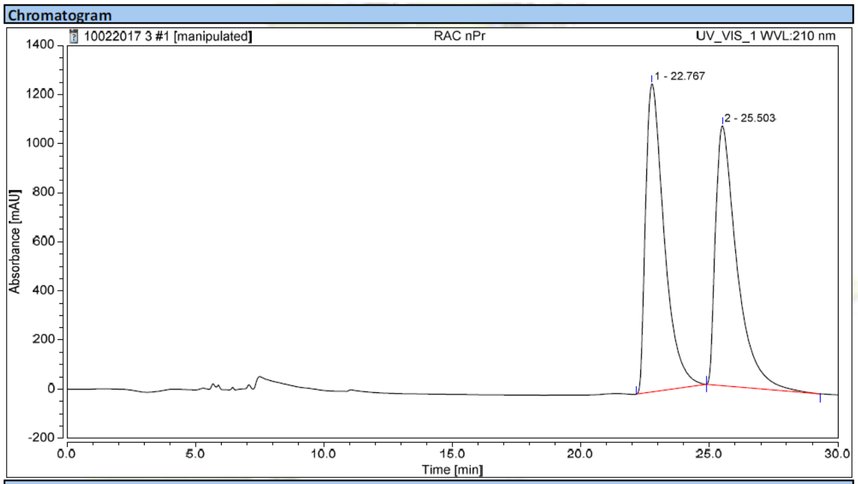

Integration Results

No.	Peak Name	Retention Time min	Area mAU*min	Height mAU	Relative Area %	Relative Height %	Amount n.a.
1		22.767	1052.710	1257.157	50.81	54.25	n.a.
2		25.503	1019.319	1060.358	49.19	45.75	n.a.
**Total:**			**2072.028**	**2317.514**	**100.00**	**100.00**	

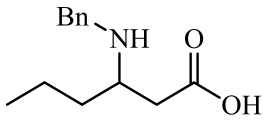

rac-**2c**

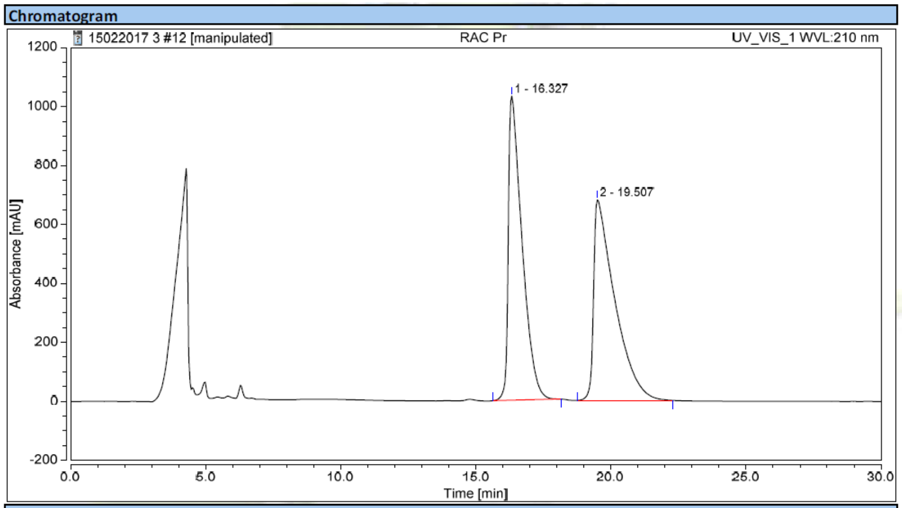

Integration Results

No.	Peak Name	Retention Time min	Area mAU*min	Height mAU	Relative Area %	Relative Height %	Amount n.a.
1		16.327	608.778	1031.470	49.69	60.24	n.a.
2		19.507	616.275	680.864	50.31	39.76	n.a.
**Total:**			**1225.053**	**1712.334**	**100.00**	**100.00**	

For entry 2.

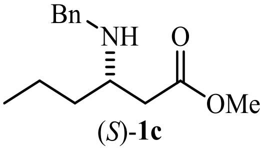

(S)-**1c**

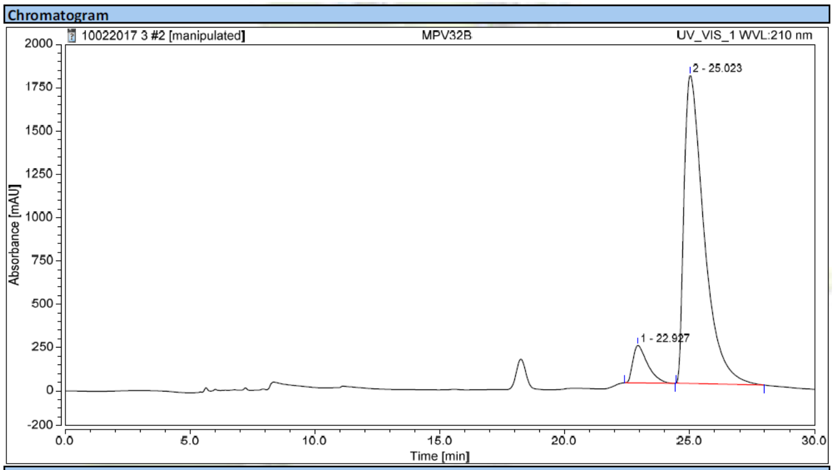

Integration Results

No.	Peak Name	Retention Time min	Area mAU*min	Height mAU	Relative Area %	Relative Height %	Amount n.a.
1		22.927	147.188	217.331	8.17	10.88	n.a.
2		25.023	1654.145	1780.392	91.83	89.12	n.a.
**Total:**			**1801.333**	**1997.723**	**100.00**	**100.00**	

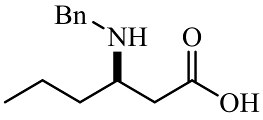

(R)-**2c**

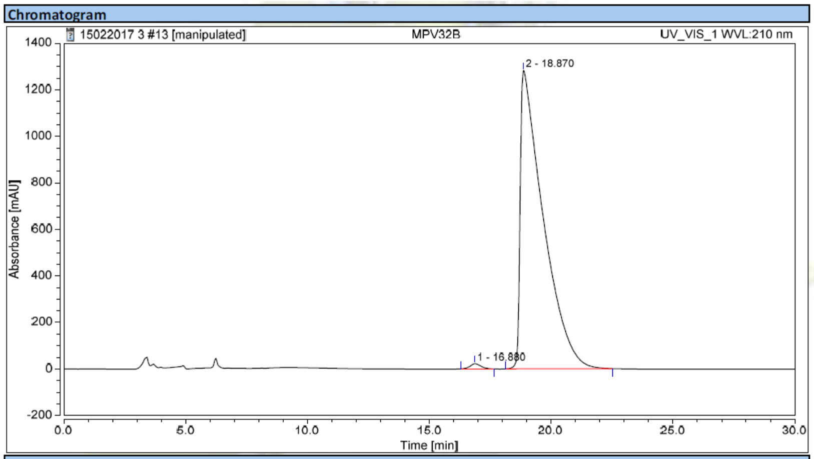

Integration Results

No.	Peak Name	Retention Time min	Area mAU*min	Height mAU	Relative Area %	Relative Height %	Amount n.a.
1		16.880	10.995	21.961	0.78	1.68	n.a.
2		18.870	1396.542	1282.962	99.22	98.32	n.a.
**Total:**			**1407.537**	**1304.924**	**100.00**	**100.00**	

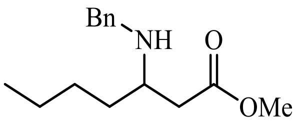

rac-**1d**

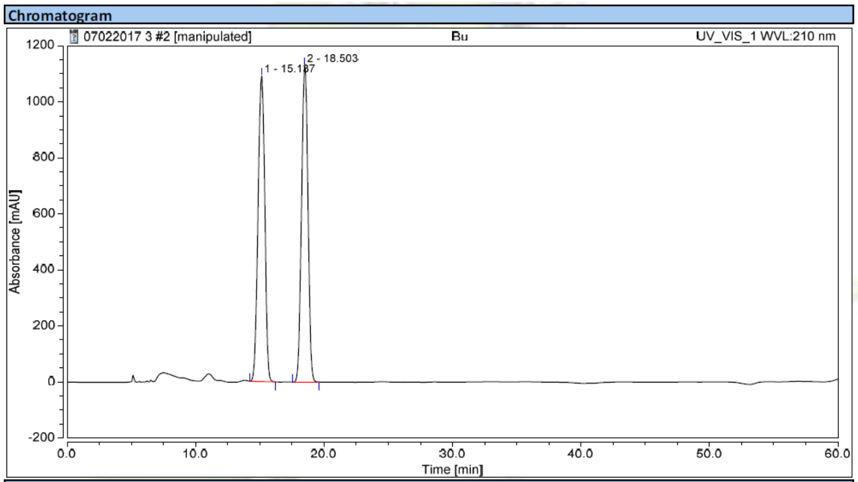

Integration Results

No.	Peak Name	Retention Time min	Area mAU*min	Height mAU	Relative Area %	Relative Height %	Amount n.a.
1		15.137	662.031	1090.083	50.30	49.12	n.a.
2		18.503	654.053	1128.935	49.70	50.88	n.a.
Total:			1316.084	2219.019	100.00	100.00	

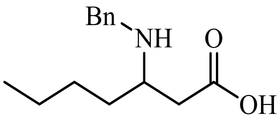

rac-**2d**

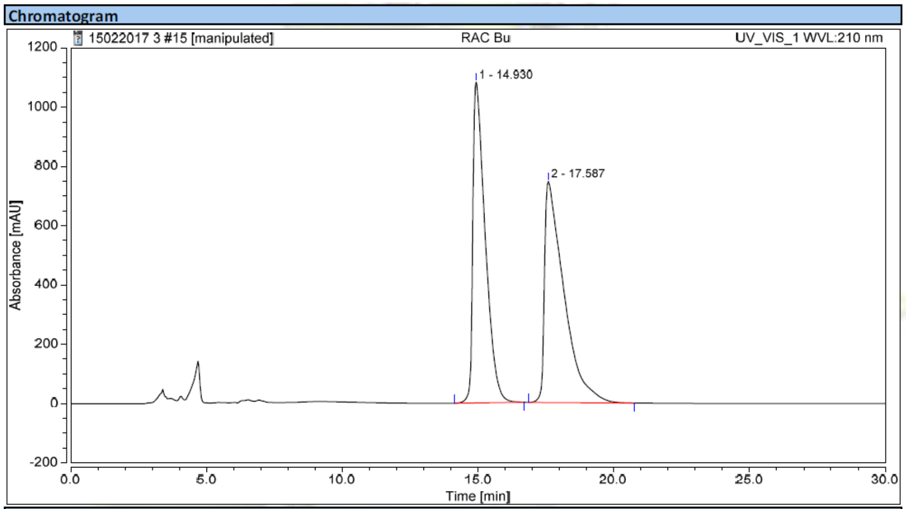

Integration Results

No.	Peak Name	Retention Time min	Area mAU*min	Height mAU	Relative Area %	Relative Height %	Amount n.a.
1		14.930	571.538	1082.133	48.13	59.17	n.a.
2		17.587	615.997	746.785	51.87	40.83	n.a.
Total:			1187.535	1828.918	100.00	100.00	

For entry 3.

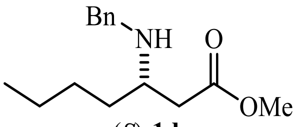

(*S*)-**1d**

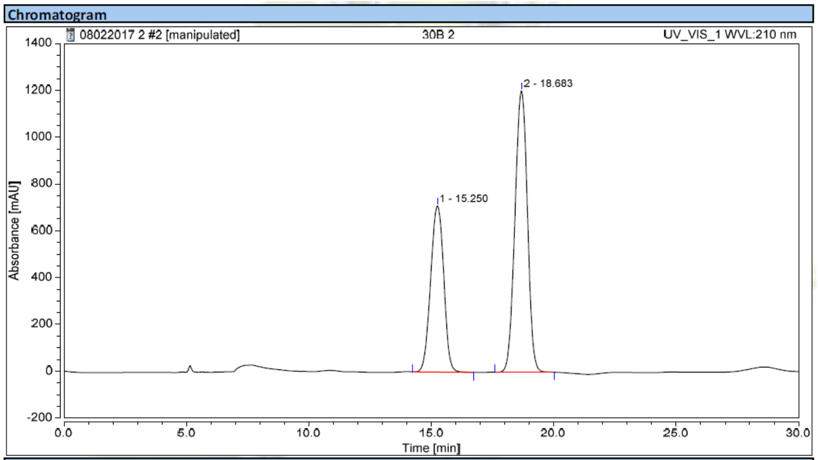

Integration Results

No.	Peak Name	Retention Time min	Area mAU*min	Height mAU	Relative Area %	Relative Height %	Amount n.a.
1		15.250	440.385	711.040	38.30	37.13	n.a.
2		18.683	709.586	1204.201	61.70	62.87	n.a.
**Total:**			**1149.971**	**1915.241**	**100.00**	**100.00**	

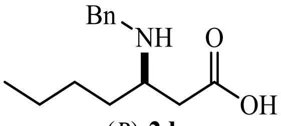

(*R*)-**2d**

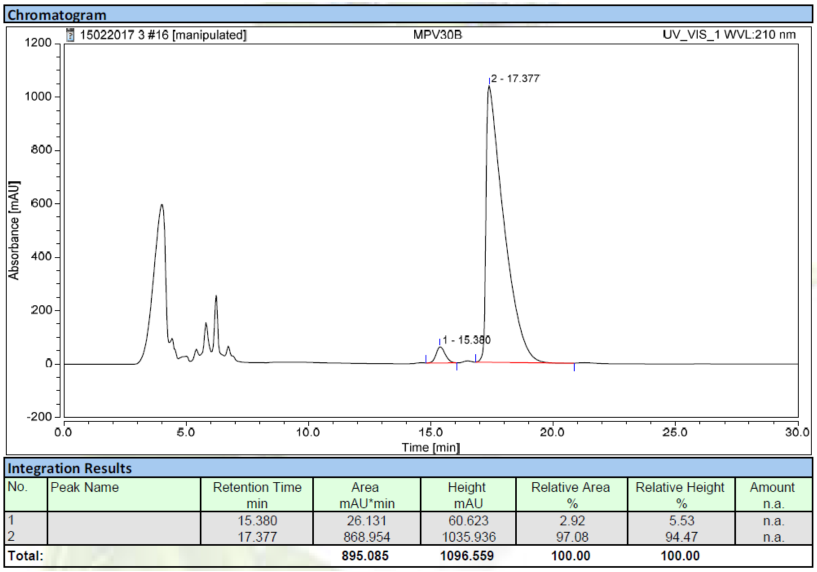

Integration Results

No.	Peak Name	Retention Time min	Area mAU*min	Height mAU	Relative Area %	Relative Height %	Amount n.a.
1		15.380	26.131	60.623	2.92	5.53	n.a.
2		17.377	868.954	1035.936	97.08	94.47	n.a.
**Total:**			**895.085**	**1096.559**	**100.00**	**100.00**	

For entry 4.

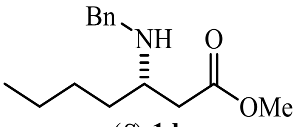

(*S*)-**1d**

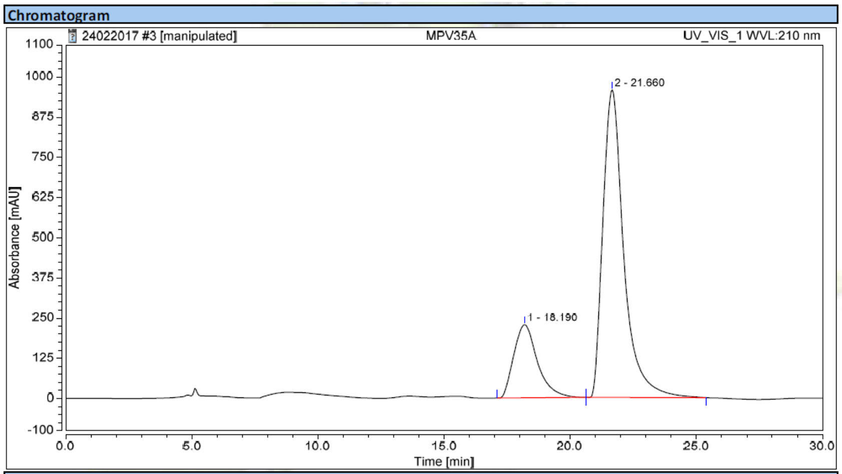

Integration Results

No.	Peak Name	Retention Time min	Area mAU*min	Height mAU	Relative Area %	Relative Height %	Amount n.a.
1		18.190	248.053	227.708	21.28	19.22	n.a.
2		21.660	917.817	956.889	78.72	80.78	n.a.
**Total:**			**1165.870**	**1184.597**	**100.00**	**100.00**	

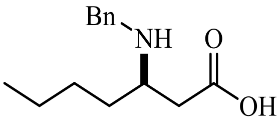

(*R*)-**2d**

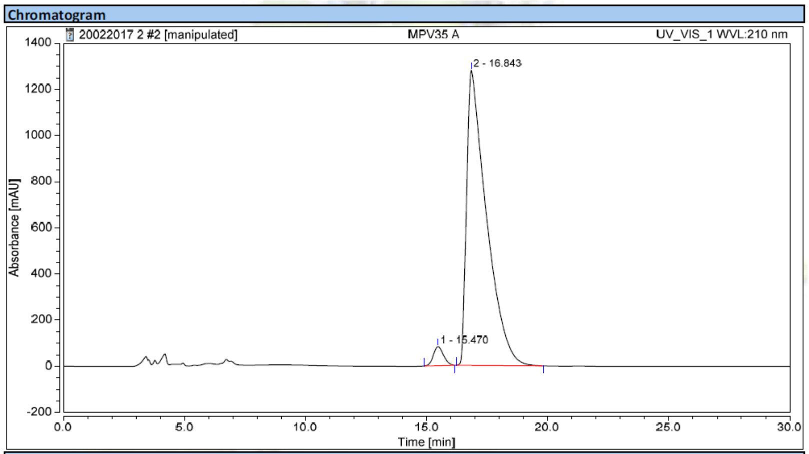

Integration Results

No.	Peak Name	Retention Time min	Area mAU*min	Height mAU	Relative Area %	Relative Height %	Amount n.a.
1		15.470	39.917	82.793	3.19	6.08	n.a.
2		16.843	1210.829	1279.227	96.81	93.92	n.a.
**Total:**			**1250.746**	**1362.020**	**100.00**	**100.00**	

For entry 5.

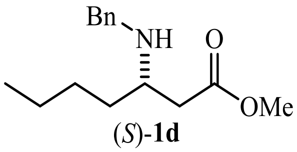

(*S*)-**1d**

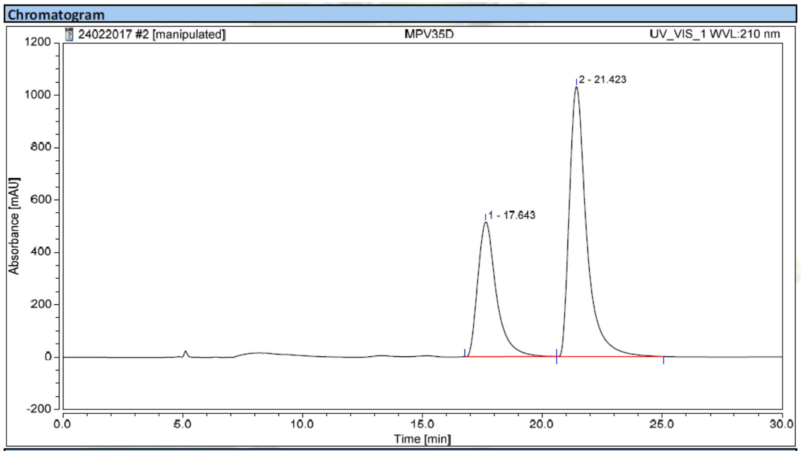

Integration Results

No.	Peak Name	Retention Time min	Area mAU*min	Height mAU	Relative Area %	Relative Height %	Amount n.a.
1		17.643	460.277	514.746	34.93	33.28	n.a.
2		21.423	857.341	1031.838	65.07	66.72	n.a.
**Total:**			**1317.617**	**1546.583**	**100.00**	**100.00**	

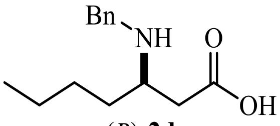

(*R*)-**2d**

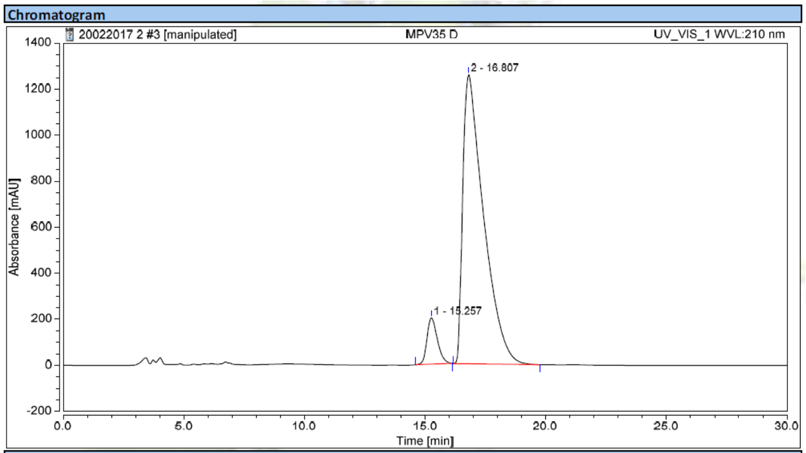

Integration Results

No.	Peak Name	Retention Time min	Area mAU*min	Height mAU	Relative Area %	Relative Height %	Amount n.a.
1		15.257	100.306	201.792	7.42	13.83	n.a.
2		16.807	1252.327	1257.107	92.58	86.17	n.a.
**Total:**			**1352.633**	**1458.899**	**100.00**	**100.00**	

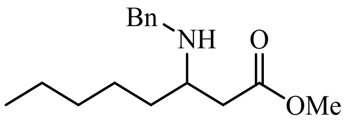

rac-**1e**

Chromatogram

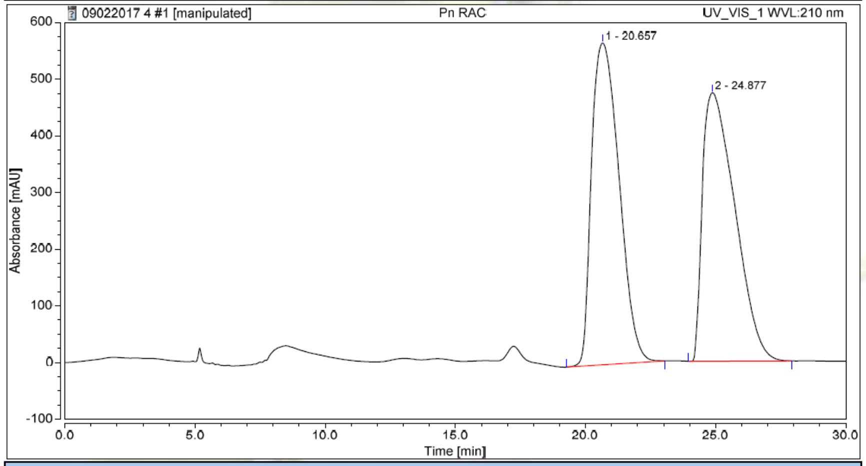

Integration Results

No.	Peak Name	Retention Time min	Area mAU*min	Height mAU	Relative Area %	Relative Height %	Amount n.a.
1		20.657	695.448	567.757	50.37	54.52	n.a.
2		24.877	685.210	473.576	49.63	45.48	n.a.
**Total:**			**1380.657**	**1041.333**	**100.00**	**100.00**	

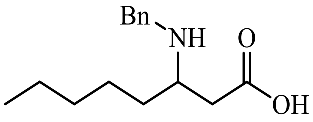

rac-**2e**

Chromatogram

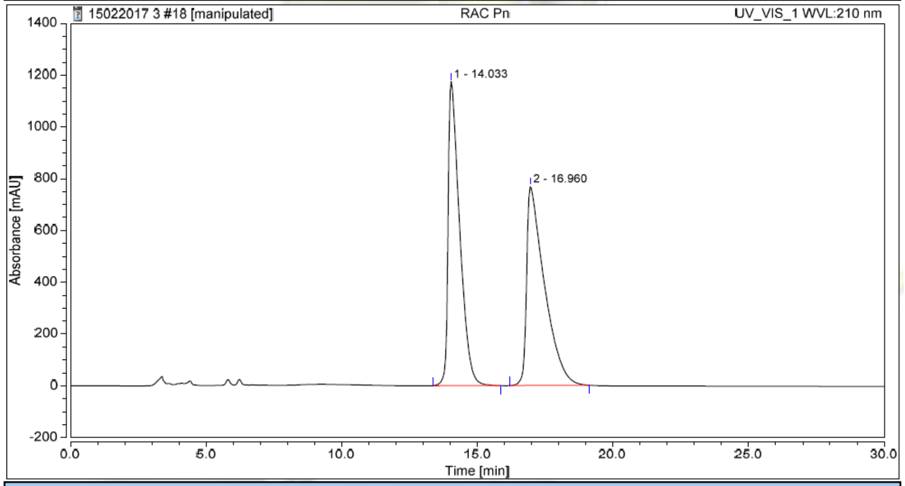

Integration Results

No.	Peak Name	Retention Time min	Area mAU*min	Height mAU	Relative Area %	Relative Height %	Amount n.a.
1		14.033	579.628	1174.789	49.77	60.41	n.a.
2		16.960	584.933	769.925	50.23	39.59	n.a.
**Total:**			**1164.561**	**1944.715**	**100.00**	**100.00**	

For entry 6.

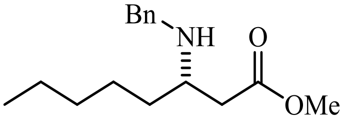

(*S*)-**1e**

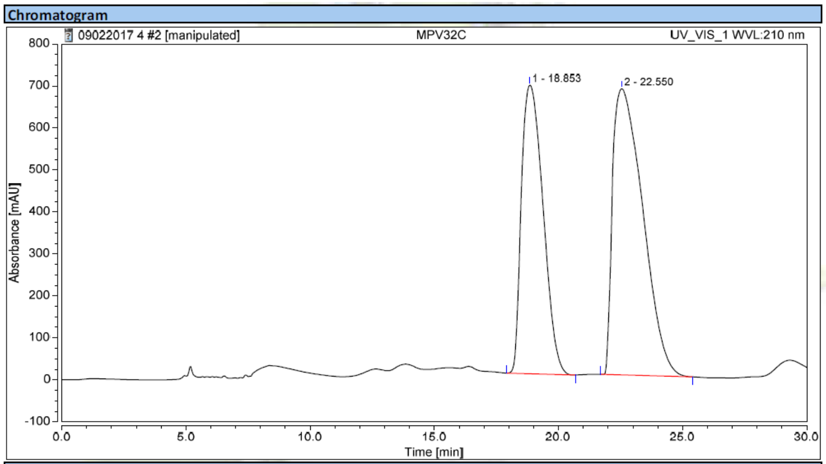

Integration Results

No.	Peak Name	Retention Time min	Area mAU*min	Height mAU	Relative Area %	Relative Height %	Amount n.a.
1		18.853	695.731	688.045	41.96	50.22	n.a.
2		22.550	962.181	681.963	58.04	49.78	n.a.
Total:			1657.912	1370.008	100.00	100.00	

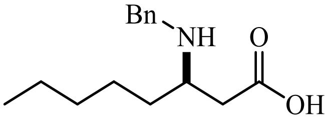

(*R*)-**2e**

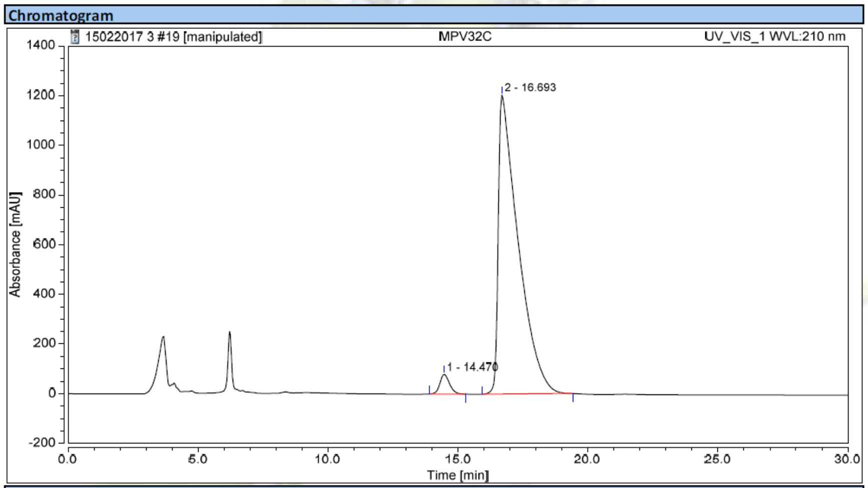

Integration Results

No.	Peak Name	Retention Time min	Area mAU*min	Height mAU	Relative Area %	Relative Height %	Amount n.a.
1		14.470	34.387	79.297	3.13	6.18	n.a.
2		16.693	1065.069	1204.825	96.87	93.82	n.a.
Total:			1099.456	1284.122	100.00	100.00	

For entry 7.

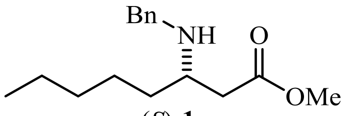

(*S*)-**1e**

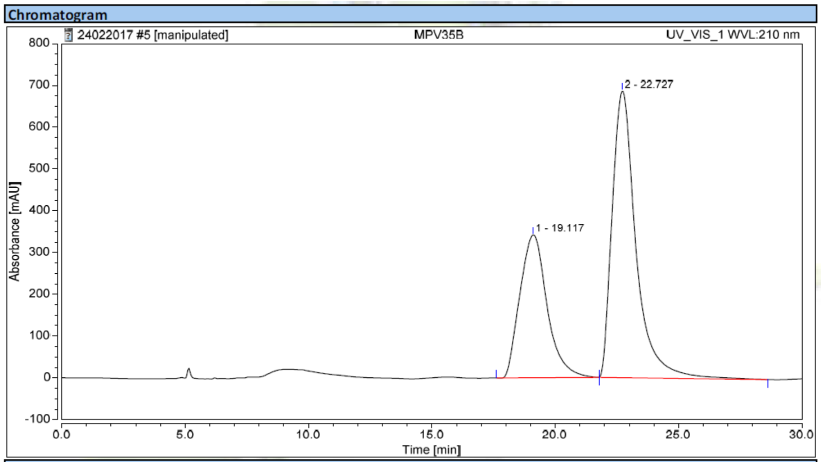

Integration Results

No.	Peak Name	Retention Time min	Area mAU*min	Height mAU	Relative Area %	Relative Height %	Amount n.a.
1		19.117	437.471	342.127	36.34	33.26	n.a.
2		22.727	766.410	686.432	63.66	66.74	n.a.
**Total:**			**1203.881**	**1028.559**	**100.00**	**100.00**	

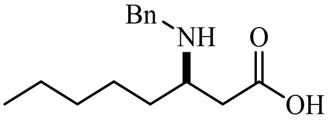

(*R*)-**2e**

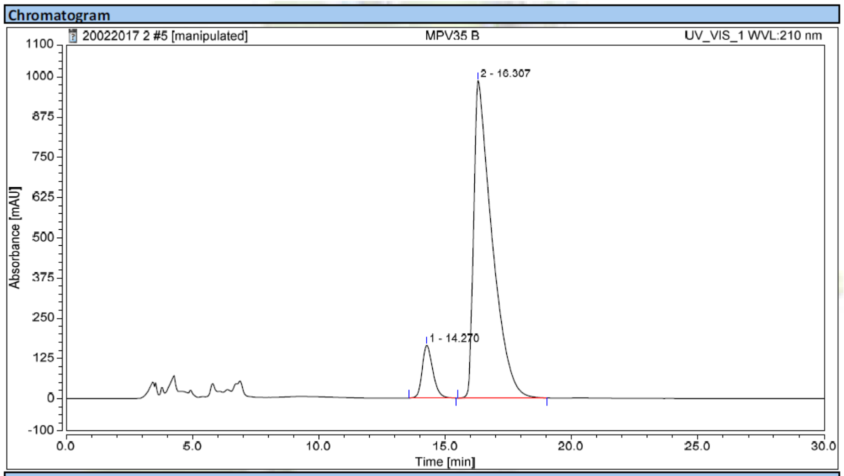

Integration Results

No.	Peak Name	Retention Time min	Area mAU*min	Height mAU	Relative Area %	Relative Height %	Amount n.a.
1		14.270	80.210	164.459	8.84	14.29	n.a.
2		16.307	827.187	986.707	91.16	85.71	n.a.
**Total:**			**907.397**	**1151.166**	**100.00**	**100.00**	

For entry 8.

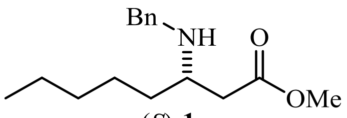

(*S*)-**1e**

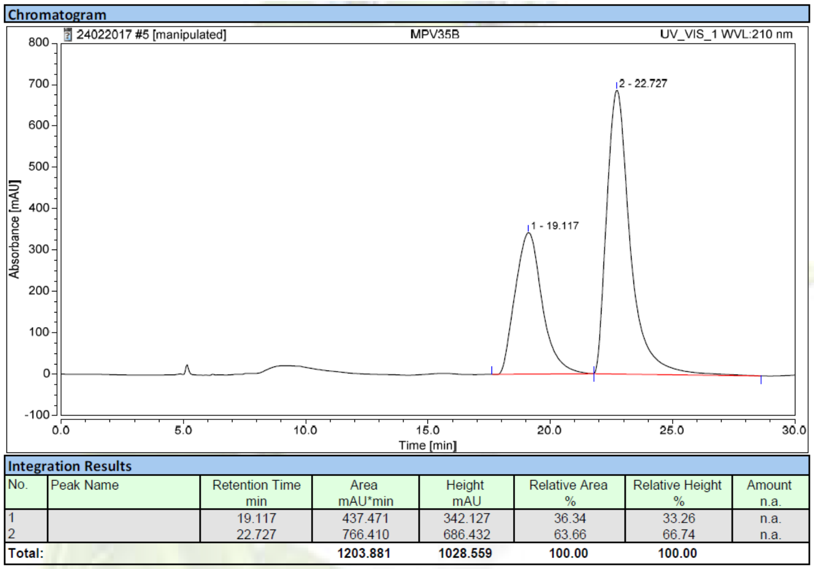

Integration Results

No.	Peak Name	Retention Time min	Area mAU*min	Height mAU	Relative Area %	Relative Height %	Amount n.a.
1		19.117	437.471	342.127	36.34	33.26	n.a.
2		22.727	766.410	686.432	63.66	66.74	n.a.
**Total:**			**1203.881**	**1028.559**	**100.00**	**100.00**	

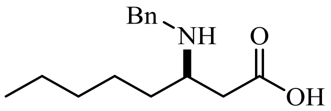

(*R*)-**2e**

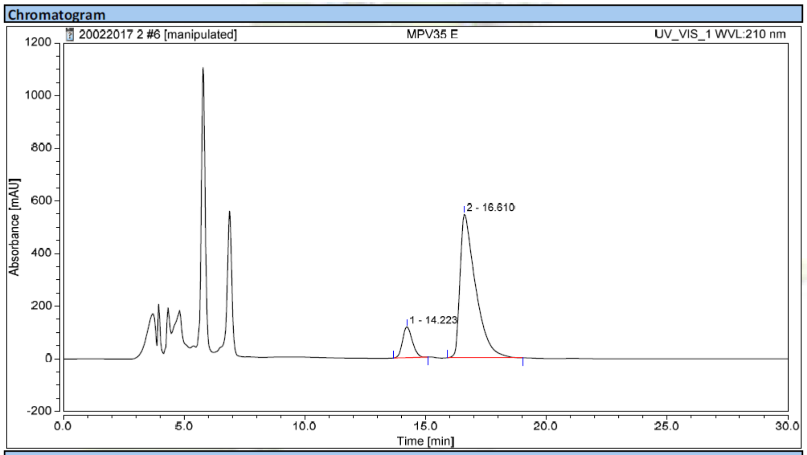

Integration Results

No.	Peak Name	Retention Time min	Area mAU*min	Height mAU	Relative Area %	Relative Height %	Amount n.a.
1		14.223	54.586	117.205	11.79	17.68	n.a.
2		16.610	408.540	545.777	88.21	82.32	n.a.
**Total:**			**463.126**	**662.982**	**100.00**	**100.00**	

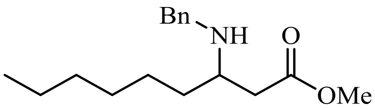

rac-**1f**

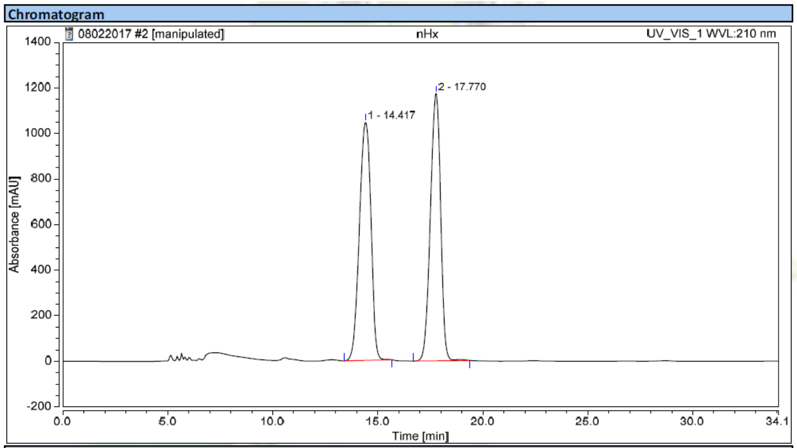

Integration Results

No.	Peak Name	Retention Time min	Area mAU*min	Height mAU	Relative Area %	Relative Height %	Amount n.a.
1		14.417	669.334	1046.779	50.27	47.14	n.a.
2		17.770	662.152	1173.847	49.73	52.86	n.a.
Total:			1331.486	2220.625	100.00	100.00	

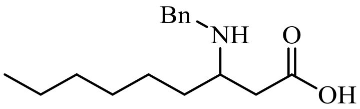

rac-**2f**

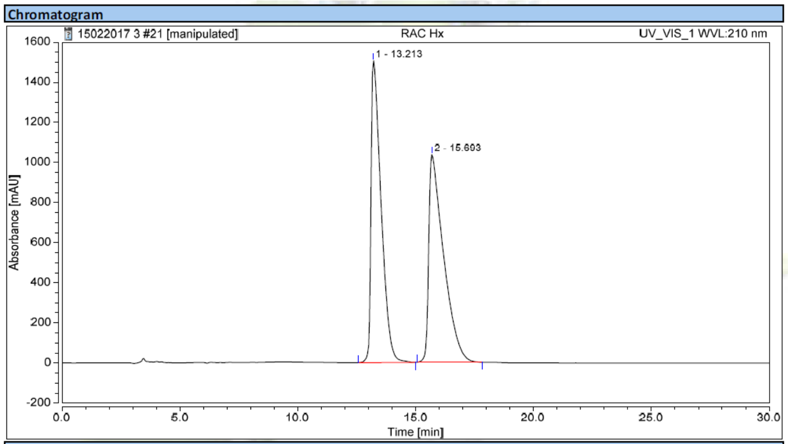

Integration Results

No.	Peak Name	Retention Time min	Area mAU*min	Height mAU	Relative Area %	Relative Height %	Amount n.a.
1		13.213	744.585	1506.831	49.60	59.24	n.a.
2		15.693	756.713	1036.692	50.40	40.76	n.a.
Total:			1501.298	2543.523	100.00	100.00	

For entry 9.

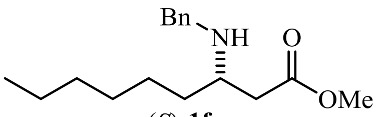

(*S*)-**1f**

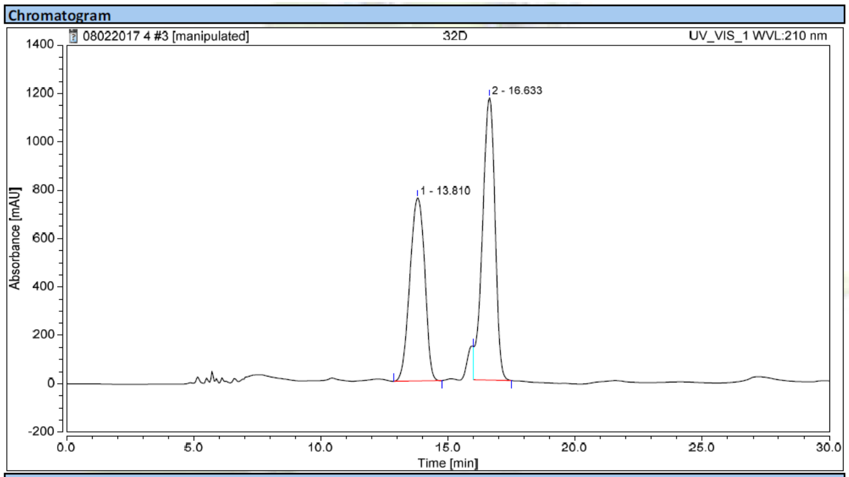

Integration Results

No.	Peak Name	Retention Time min	Area mAU*min	Height mAU	Relative Area %	Relative Height %	Amount n.a.
1		13.810	512.845	757.376	43.45	39.33	n.a.
2		16.633	667.419	1168.452	56.55	60.67	n.a.
**Total:**			**1180.264**	**1925.828**	**100.00**	**100.00**	

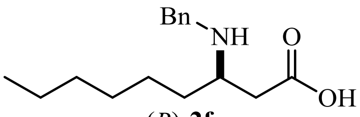

(*R*)-**2f**

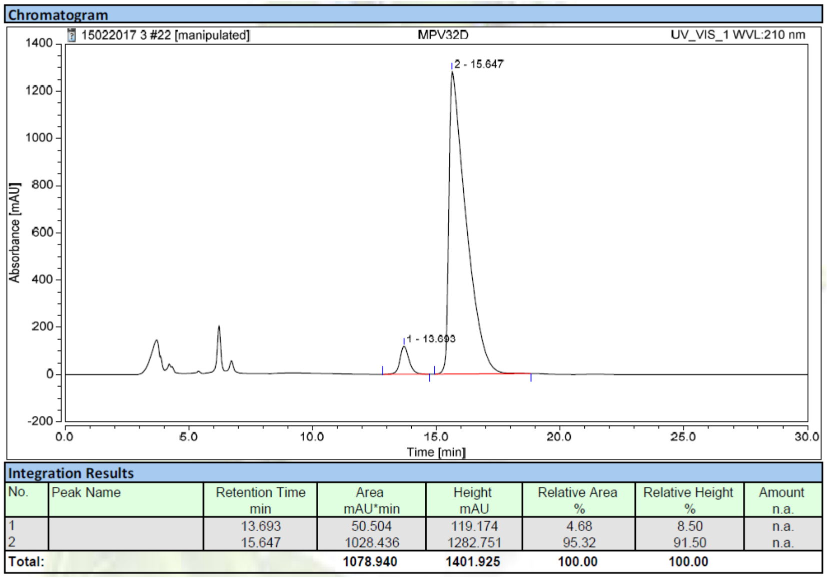

Integration Results

No.	Peak Name	Retention Time min	Area mAU*min	Height mAU	Relative Area %	Relative Height %	Amount n.a.
1		13.693	50.504	119.174	4.68	8.50	n.a.
2		15.647	1028.436	1282.751	95.32	91.50	n.a.
**Total:**			**1078.940**	**1401.925**	**100.00**	**100.00**	

For entry 10.

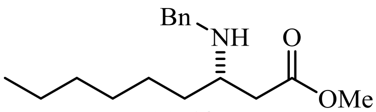

(*S*)-**1f**

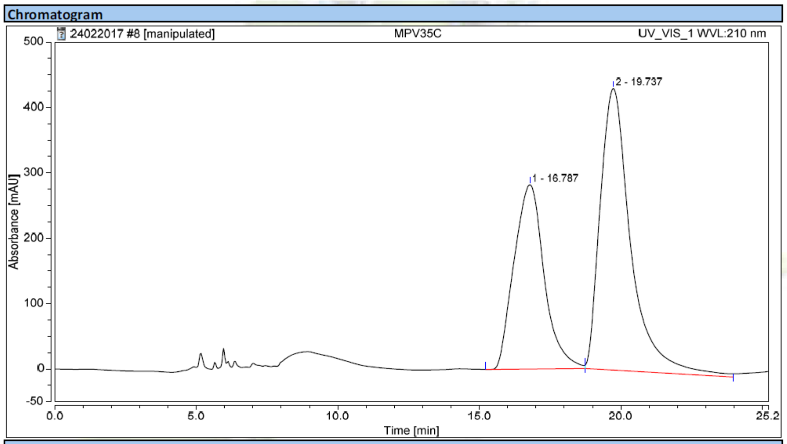

Integration Results

No.	Peak Name	Retention Time<br>min	Area<br>mAU*min	Height<br>mAU	Relative Area<br>%	Relative Height<br>%	Amount<br>n.a.
1		16.787	348.149	281.930	39.29	39.56	n.a.
2		19.737	538.034	430.813	60.71	60.44	n.a.
Total:			886.182	712.743	100.00	100.00	

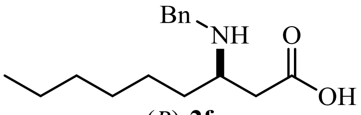

(*R*)-**2f**

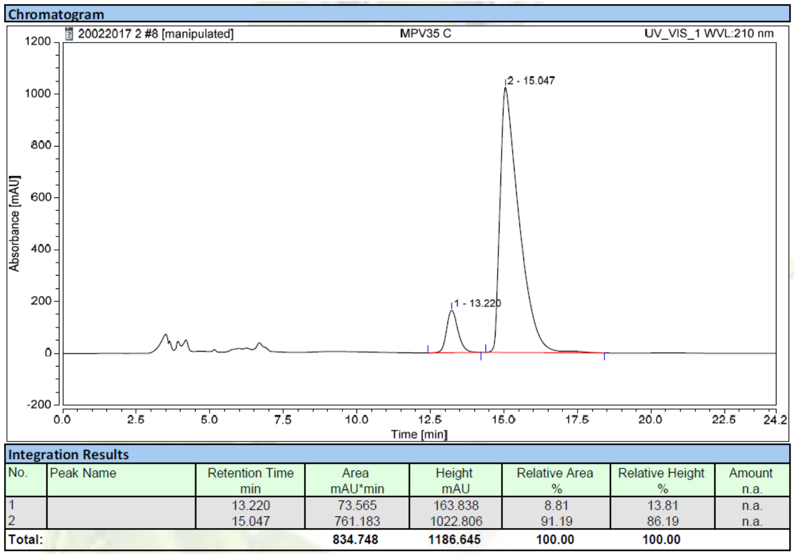

Integration Results

No.	Peak Name	Retention Time<br>min	Area<br>mAU*min	Height<br>mAU	Relative Area<br>%	Relative Height<br>%	Amount<br>n.a.
1		13.220	73.565	163.838	8.81	13.81	n.a.
2		15.047	761.183	1022.806	91.19	86.19	n.a.
Total:			834.748	1186.645	100.00	100.00	

For entry 11.

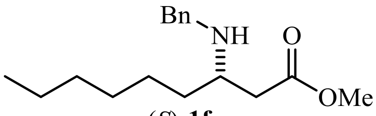

(*S*)-**1f**

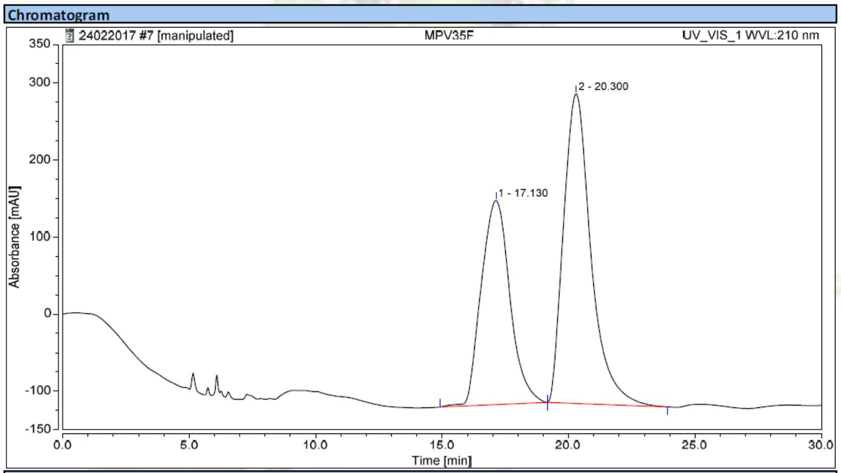

Integration Results

No.	Peak Name	Retention Time min	Area mAU*min	Height mAU	Relative Area %	Relative Height %	Amount n.a.
1		17.130	347.593	264.963	40.81	39.71	n.a.
2		20.300	504.212	402.237	59.19	60.29	n.a.
**Total:**			**851.804**	**667.201**	**100.00**	**100.00**	

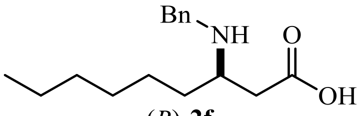

(*R*)-**2f**

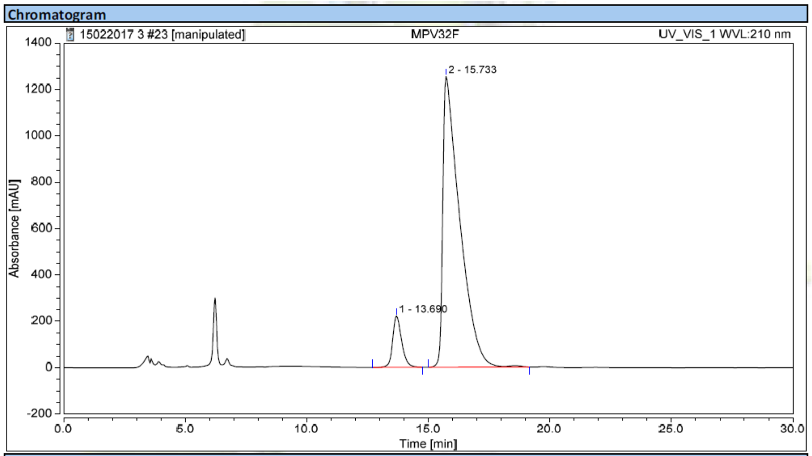

Integration Results

No.	Peak Name	Retention Time min	Area mAU*min	Height mAU	Relative Area %	Relative Height %	Amount n.a.
1		13.690	94.699	221.585	8.55	15.01	n.a.
2		15.733	1012.385	1254.546	91.45	84.99	n.a.
**Total:**			**1107.084**	**1476.131**	**100.00**	**100.00**	

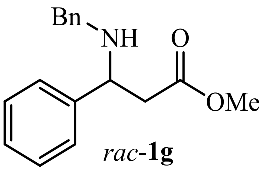

rac-**1g**

Chromatogram

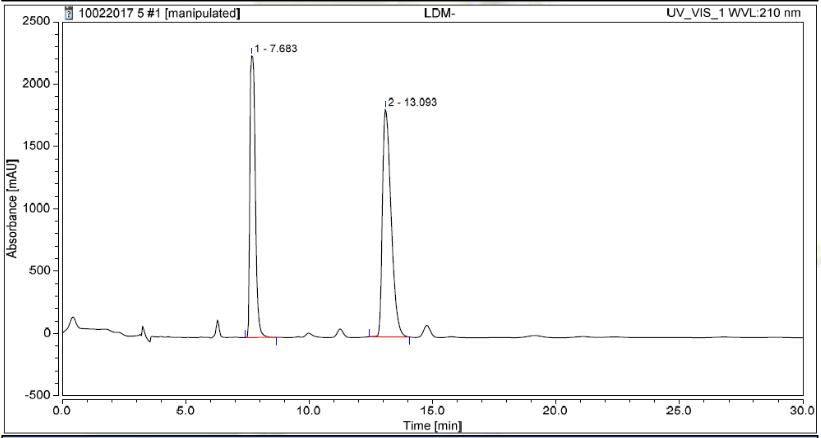

Integration Results

No.	Peak Name	Retention Time min	Area mAU*min	Height mAU	Relative Area %	Relative Height %	Amount n.a.
1		7.683	575.296	2264.166	43.60	55.31	n.a.
2		13.093	744.149	1829.154	56.40	44.69	n.a.
**Total:**			**1319.445**	**4093.320**	**100.00**	**100.00**	

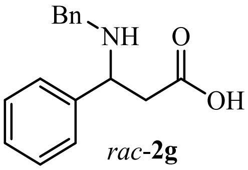

rac-**2g**

Chromatogram

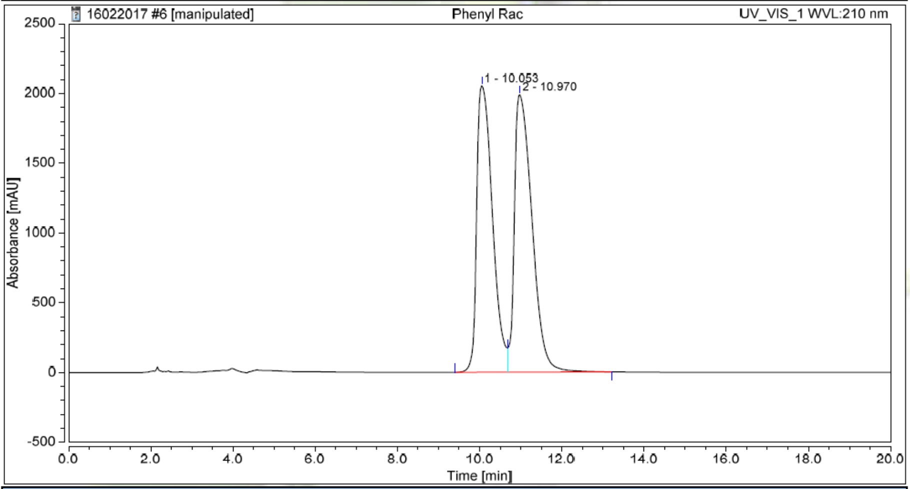

Integration Results

No.	Peak Name	Retention Time min	Area mAU*min	Height mAU	Relative Area %	Relative Height %	Amount n.a.
1		10.053	911.637	2055.268	47.97	50.79	n.a.
2		10.970	988.669	1991.275	52.03	49.21	n.a.
**Total:**			**1900.306**	**4046.544**	**100.00**	**100.00**	

For entry 12.

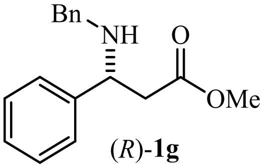

(*R*)-**1g**

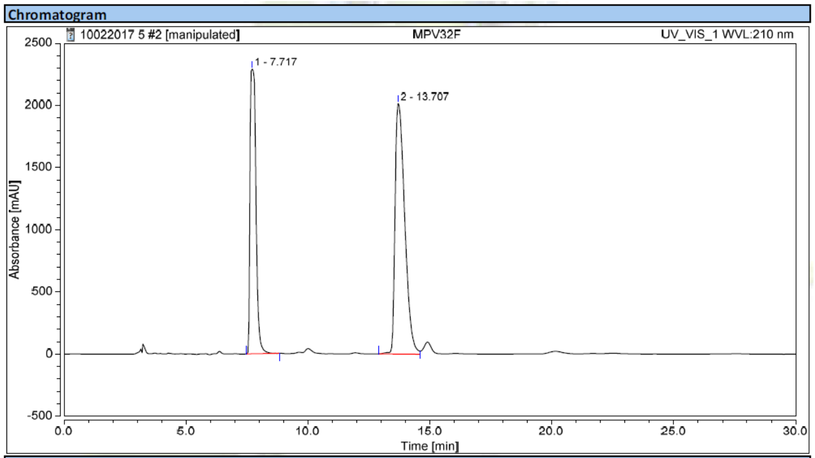

Integration Results

No.	Peak Name	Retention Time min	Area mAU*min	Height mAU	Relative Area %	Relative Height %	Amount n.a.
1		7.717	648.985	2292.370	41.25	53.20	n.a.
2		13.707	924.193	2016.550	58.75	46.80	n.a.
Total:			1573.178	4308.920	100.00	100.00	

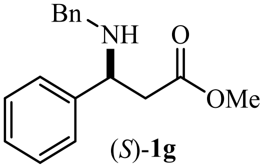

(*S*)-**1g**

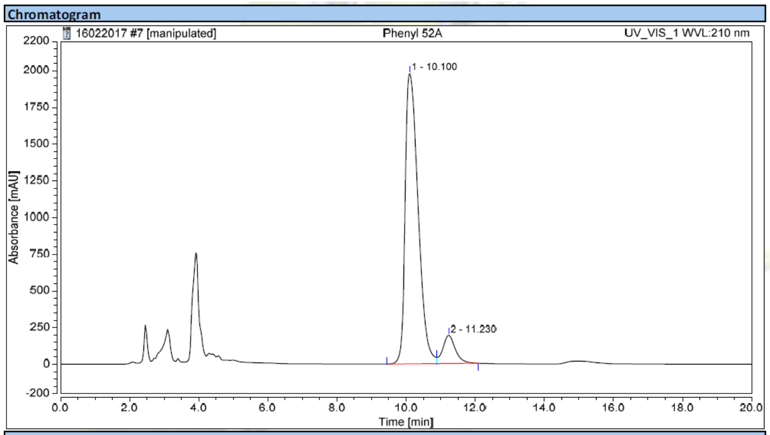

Integration Results

No.	Peak Name	Retention Time min	Area mAU*min	Height mAU	Relative Area %	Relative Height %	Amount n.a.
1		10.100	855.620	1980.535	91.35	91.17	n.a.
2		11.230	80.979	191.903	8.65	8.83	n.a.
Total:			936.599	2172.438	100.00	100.00	

For entry 13.

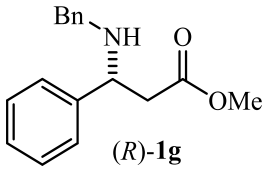

(*R*)-**1g**

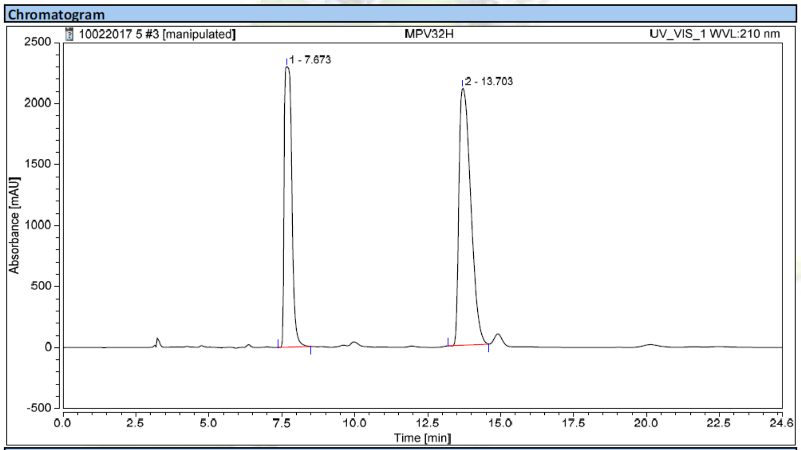

Integration Results

No.	Peak Name	Retention Time min	Area mAU*min	Height mAU	Relative Area %	Relative Height %	Amount n.a.
1		7.673	689.362	2300.475	41.14	52.18	n.a.
2		13.703	986.331	2108.185	58.86	47.82	n.a.
**Total:**			**1675.693**	**4408.660**	**100.00**	**100.00**	

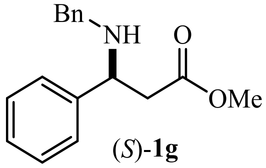

(*S*)-**1g**

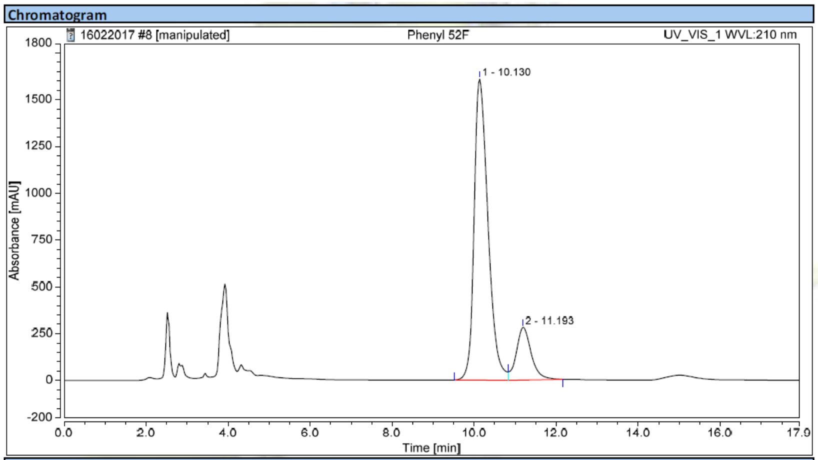

Integration Results

No.	Peak Name	Retention Time min	Area mAU*min	Height mAU	Relative Area %	Relative Height %	Amount n.a.
1		10.130	629.952	1609.806	84.16	85.09	n.a.
2		11.193	118.603	281.989	15.84	14.91	n.a.
**Total:**			**748.555**	**1891.795**	**100.00**	**100.00**	

For entry 14.

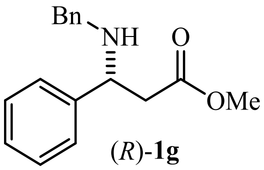

(*R*)-**1g**

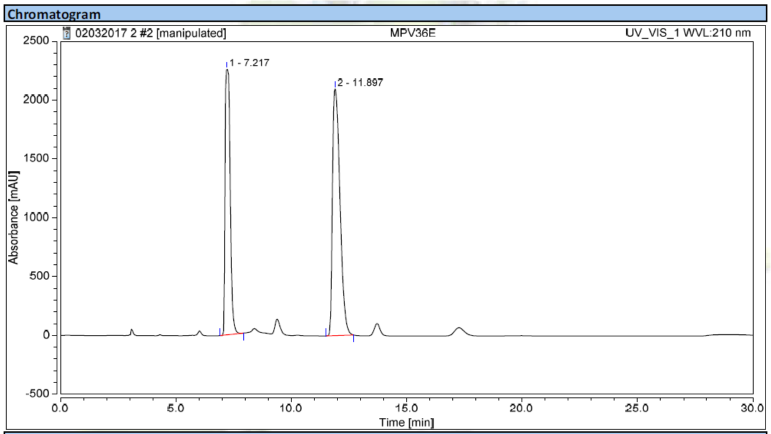

Integration Results

No.	Peak Name	Retention Time min	Area mAU*min	Height mAU	Relative Area %	Relative Height %	Amount n.a.
1		7.217	575.806	2259.430	42.19	51.87	n.a.
2		11.897	789.032	2096.621	57.81	48.13	n.a.
Total:			1364.837	4356.051	100.00	100.00	

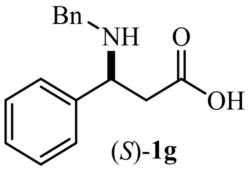

(*S*)-**1g**

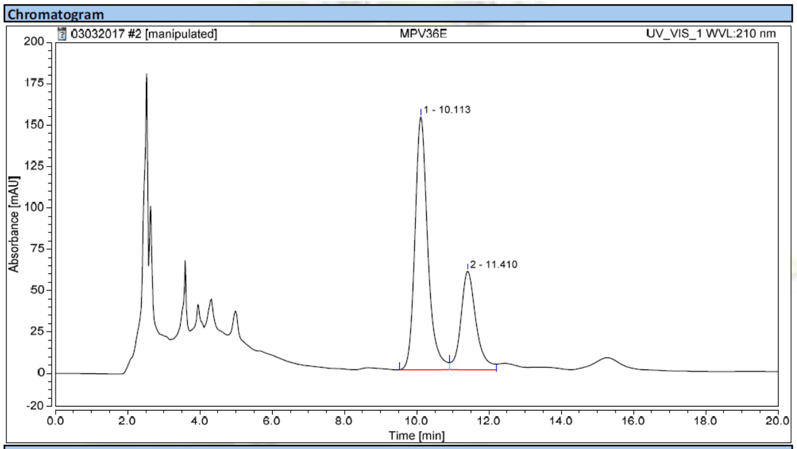

Integration Results

No.	Peak Name	Retention Time min	Area mAU*min	Height mAU	Relative Area %	Relative Height %	Amount n.a.
1		10.113	62.639	152.985	68.40	71.97	n.a.
2		11.410	28.937	59.581	31.60	28.03	n.a.
Total:			91.576	212.565	100.00	100.00	

For entry 15.

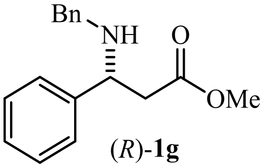

(*R*)-**1g**

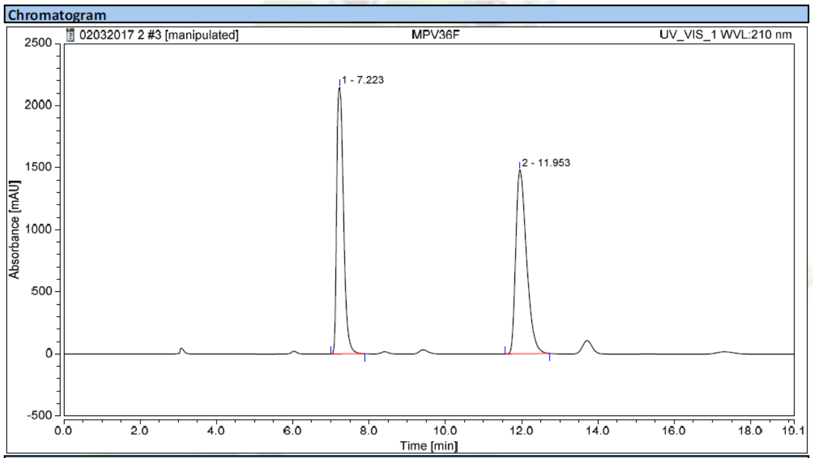

Integration Results

No.	Peak Name	Retention Time min	Area mAU*min	Height mAU	Relative Area %	Relative Height %	Amount n.a.
1		7.223	442.323	2149.784	47.67	59.15	n.a.
2		11.953	485.590	1484.526	52.33	40.85	n.a.
**Total:**			**927.913**	**3634.310**	**100.00**	**100.00**	

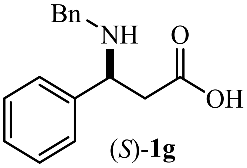

(*S*)-**1g**

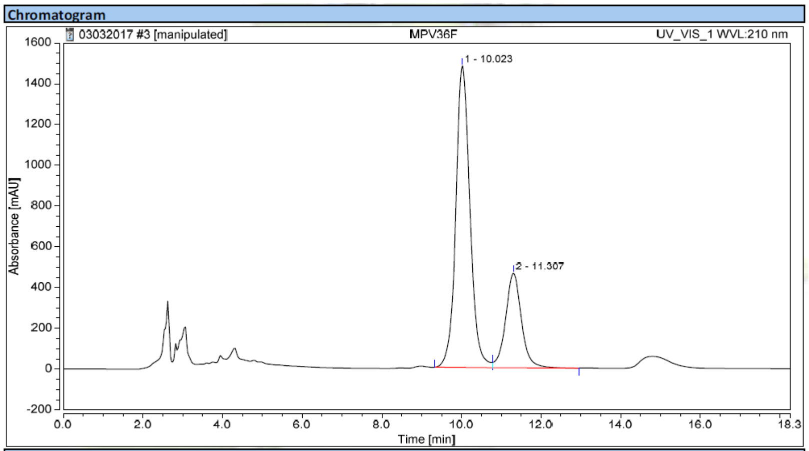

Integration Results

No.	Peak Name	Retention Time min	Area mAU*min	Height mAU	Relative Area %	Relative Height %	Amount n.a.
1		10.023	610.816	1481.644	74.07	76.11	n.a.
2		11.307	213.871	465.079	25.93	23.89	n.a.
**Total:**			**824.686**	**1946.723**	**100.00**	**100.00**	

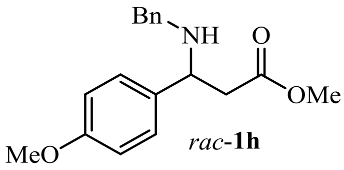

rac-**1h**

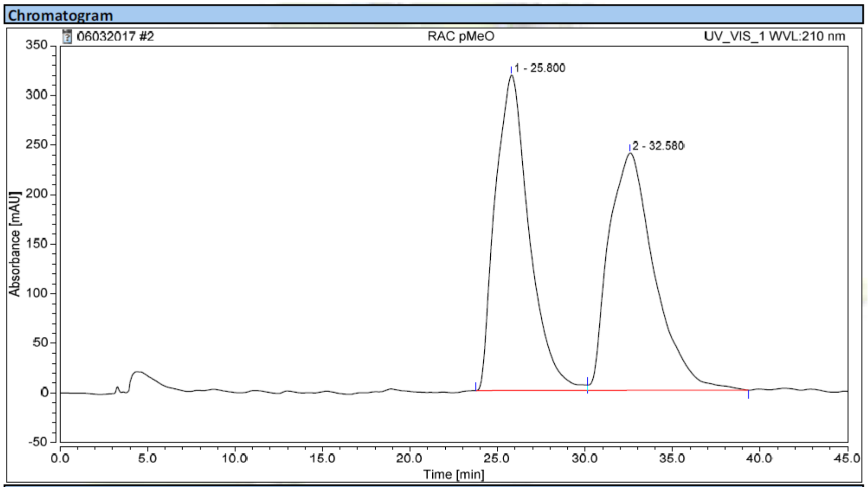

Integration Results

No.	Peak Name	Retention Time min	Area mAU*min	Height mAU	Relative Area %	Relative Height %	Amount n.a.
1		25.800	714.671	318.077	49.67	57.07	n.a.
2		32.580	724.084	239.269	50.33	42.93	n.a.
Total:			1438.754	557.347	100.00	100.00	

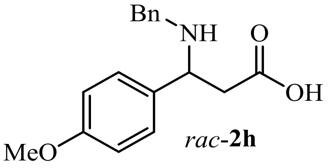

rac-**2h**

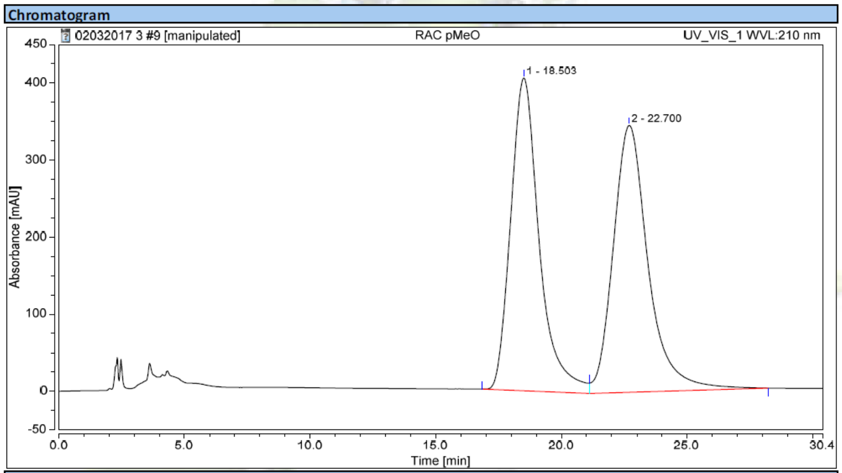

Integration Results

No.	Peak Name	Retention Time min	Area mAU*min	Height mAU	Relative Area %	Relative Height %	Amount n.a.
1		18.503	534.089	406.076	48.90	53.96	n.a.
2		22.700	558.163	346.477	51.10	46.04	n.a.
Total:			1092.252	752.553	100.00	100.00	

For entry 16.

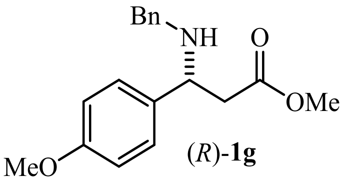

(*R*)-**1g**

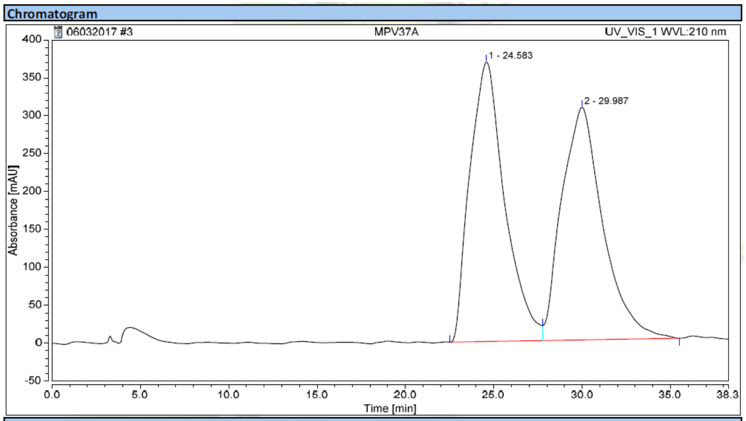

Integration Results

No.	Peak Name	Retention Time min	Area mAU*min	Height mAU	Relative Area %	Relative Height %	Amount n.a.
1		24.583	842.045	368.822	50.44	54.57	n.a.
2		29.987	827.432	307.072	49.56	45.43	n.a.
**Total:**			**1669.477**	**675.895**	**100.00**	**100.00**	

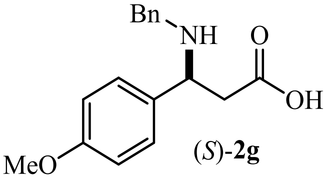

(*S*)-**2g**

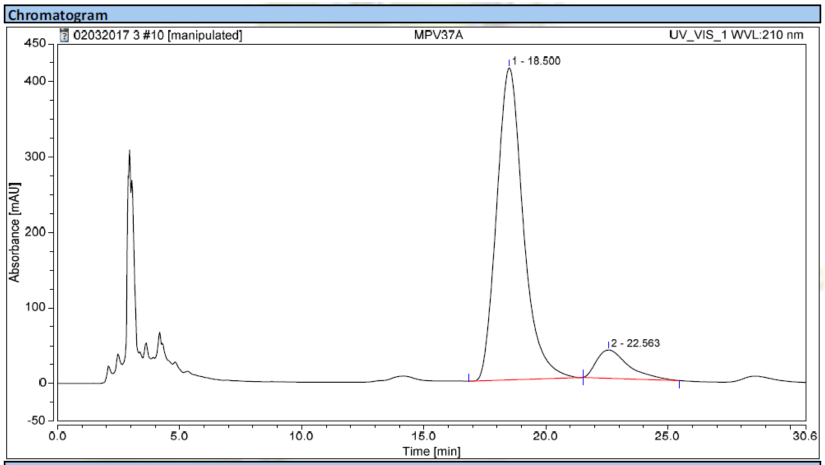

Integration Results

No.	Peak Name	Retention Time min	Area mAU*min	Height mAU	Relative Area %	Relative Height %	Amount n.a.
1		18.500	521.347	413.777	90.19	91.69	n.a.
2		22.563	56.687	37.507	9.81	8.31	n.a.
**Total:**			**578.034**	**451.284**	**100.00**	**100.00**	

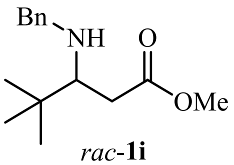

rac-**1i**

Chromatogram

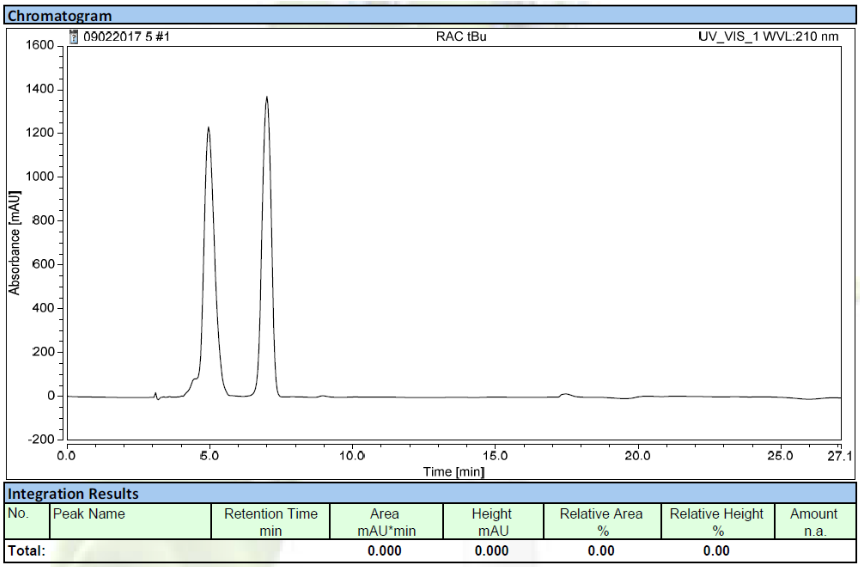

Integration Results

No.	Peak Name	Retention Time min	Area mAU*min	Height mAU	Relative Area %	Relative Height %	Amount n.a.
**Total:**			**0.000**	**0.000**	**0.00**	**0.00**	

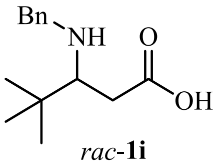

rac-**1i**

Chromatogram

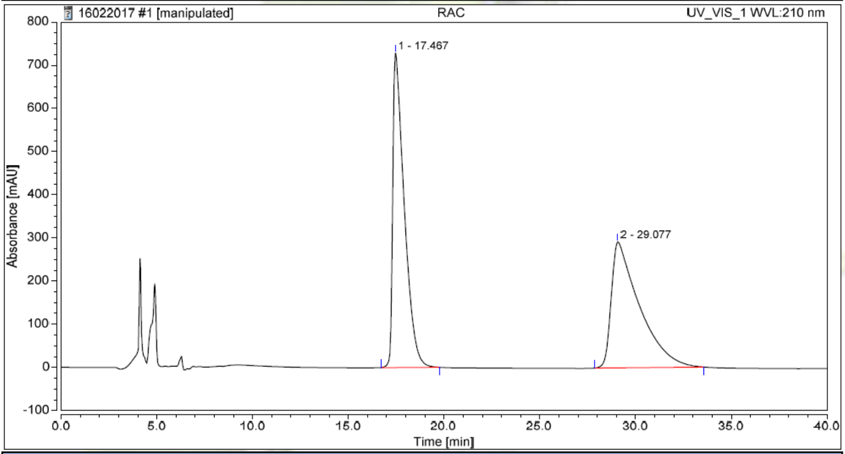

Integration Results

No.	Peak Name	Retention Time min	Area mAU*min	Height mAU	Relative Area %	Relative Height %	Amount n.a.
1		17.467	504.303	728.813	50.45	71.38	n.a.
2		29.077	495.300	292.180	49.55	28.62	n.a.
**Total:**			**999.604**	**1020.993**	**100.00**	**100.00**	

For entry 17.

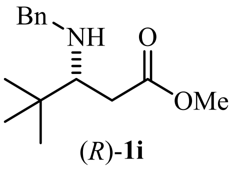

(*R*)-**1i**

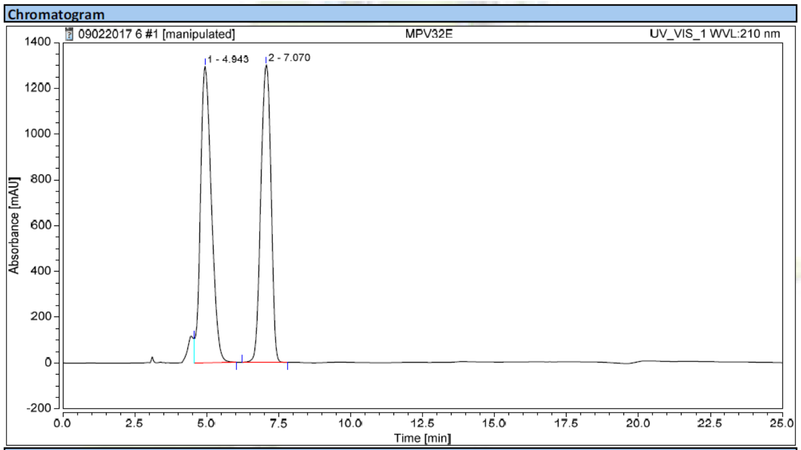

Integration Results

No.	Peak Name	Retention Time min	Area mAU*min	Height mAU	Relative Area %	Relative Height %	Amount n.a.
1		4.943	569.716	1295.633	51.79	49.89	n.a.
2		7.070	530.385	1301.572	48.21	50.11	n.a.
**Total:**			**1100.101**	**2597.206**	**100.00**	**100.00**	

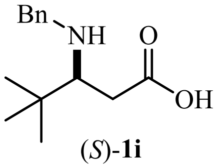

(*S*)-**1i**

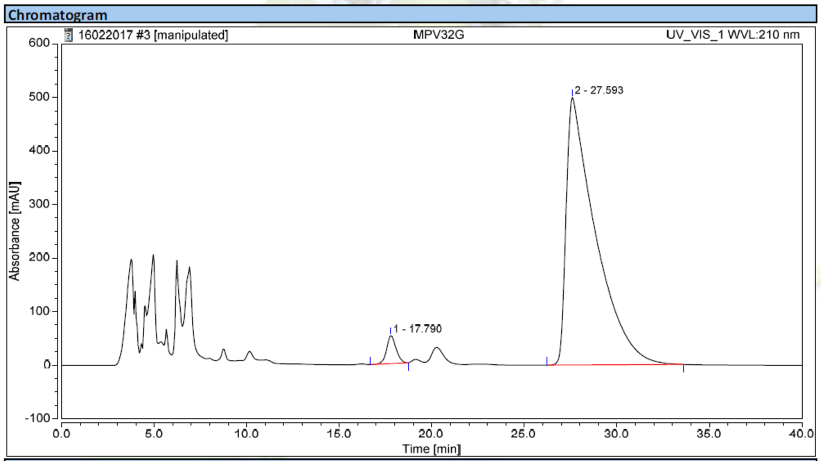

Integration Results

No.	Peak Name	Retention Time min	Area mAU*min	Height mAU	Relative Area %	Relative Height %	Amount n.a.
1		17.790	30.531	51.968	3.23	9.42	n.a.
2		27.593	916.024	499.535	96.77	90.58	n.a.
**Total:**			**946.554**	**551.503**	**100.00**	**100.00**	

For entry 18.

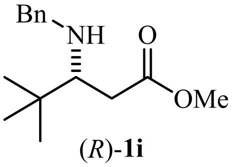

(*R*)-**1i**

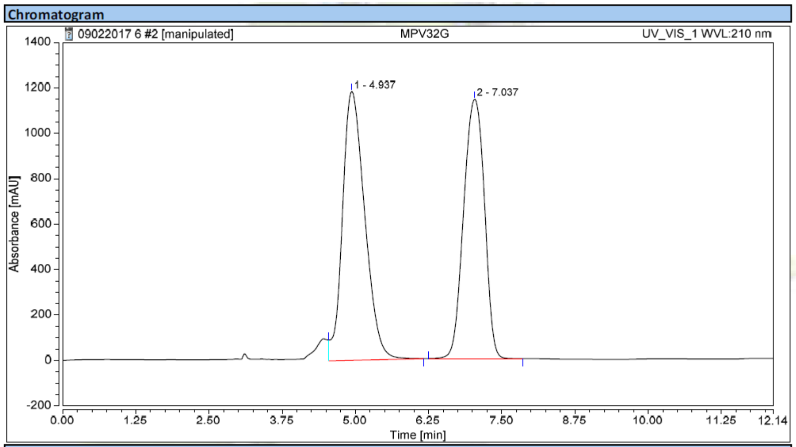

Integration Results

No.	Peak Name	Retention Time min	Area mAU*min	Height mAU	Relative Area %	Relative Height %	Amount n.a.
1		4.937	522.647	1184.487	52.61	50.89	n.a.
2		7.037	470.776	1143.065	47.39	49.11	n.a.
**Total:**			**993.423**	**2327.552**	**100.00**	**100.00**	

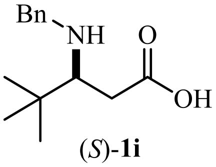

(*S*)-**1i**

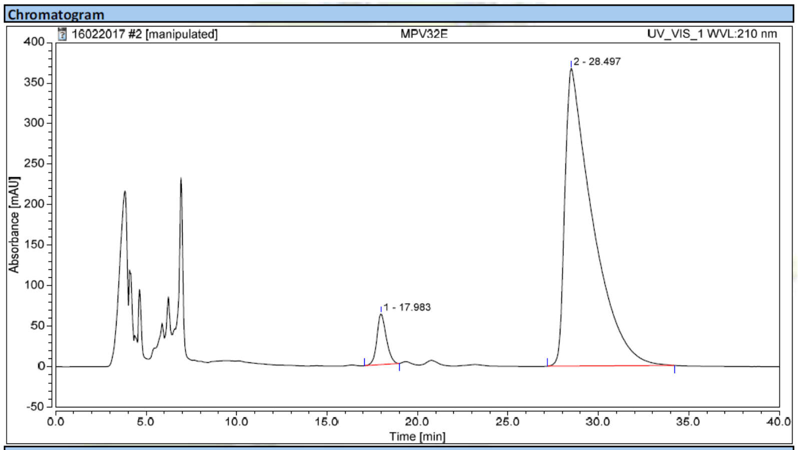

Integration Results

No.	Peak Name	Retention Time min	Area mAU*min	Height mAU	Relative Area %	Relative Height %	Amount n.a.
1		17.983	37.731	62.562	5.47	14.55	n.a.
2		28.497	651.750	367.461	94.53	85.45	n.a.
**Total:**			**689.481**	**430.023**	**100.00**	**100.00**	

- **Chromatograms for the evaluation of recycling capacity of immobilized CALB (Table S3).**

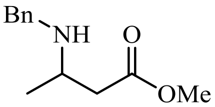

rac-**1a**

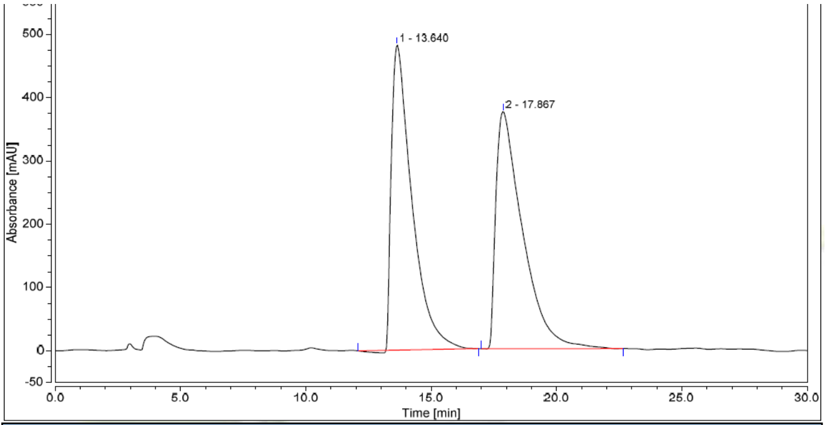

Integration Results							
No.	Peak Name	Retention Time min	Area mAU*min	Height mAU	Relative Area %	Relative Height %	Amount n.a.
1		13.640	455.884	482.090	49.63	56.27	n.a.
2		17.867	462.617	374.599	50.37	43.73	n.a.
Total:			918.501	856.689	100.00	100.00	

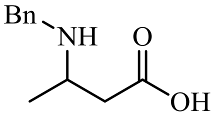

rac-**2a**

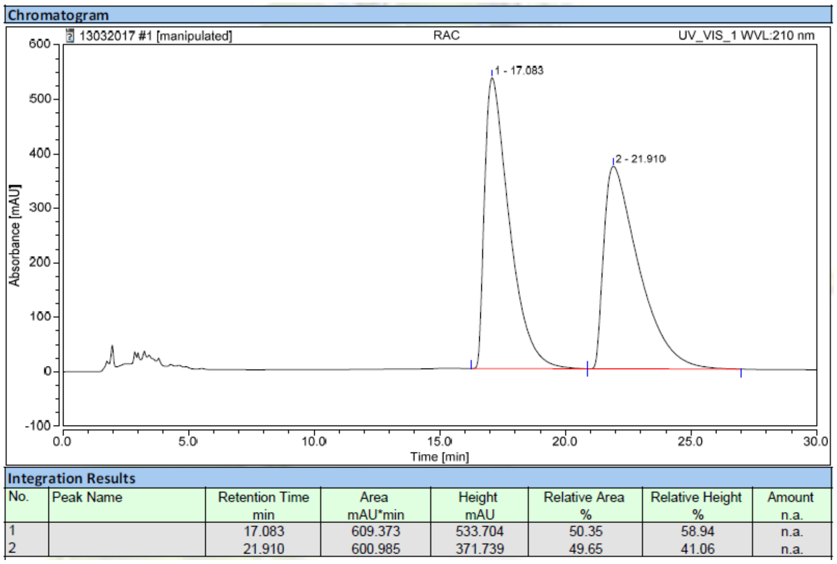

Integration Results							
No.	Peak Name	Retention Time min	Area mAU*min	Height mAU	Relative Area %	Relative Height %	Amount n.a.
1		17.083	609.373	533.704	50.35	58.94	n.a.
2		21.910	600.985	371.739	49.65	41.06	n.a.

For entry 2.

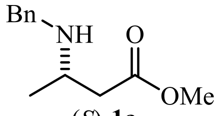

(S)-**1a**

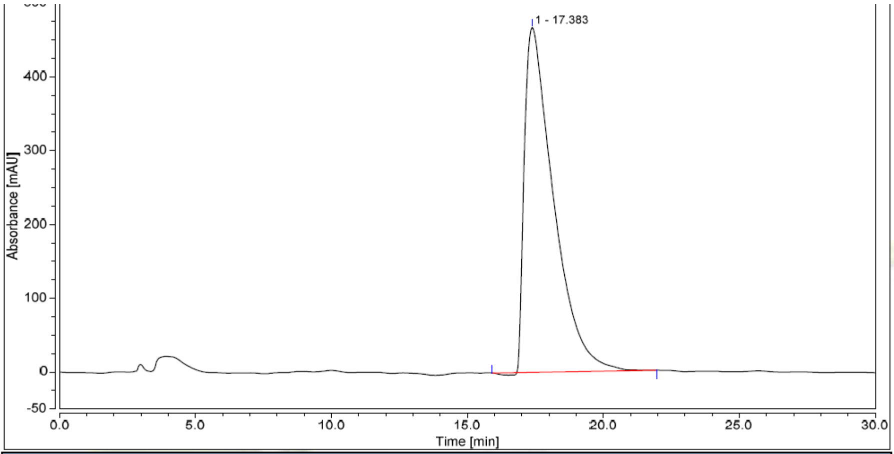

Integration Results							
No.	Peak Name	Retention Time min	Area mAU*min	Height mAU	Relative Area %	Relative Height %	Amount n.a.
1		17.383	579.249	467.640	100.00	100.00	n.a.
**Total:**			**579.249**	**467.640**	**100.00**	**100.00**	

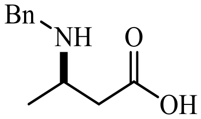

(R)-**2a**

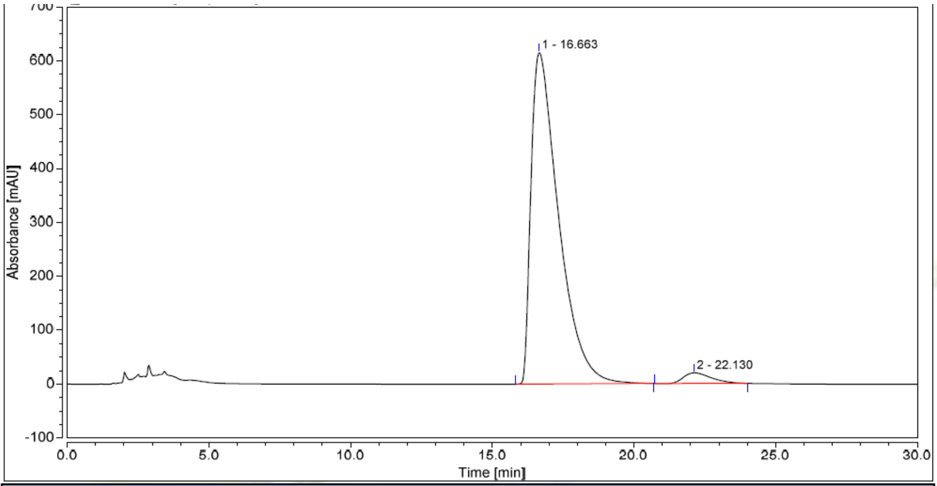

Integration Results							
No.	Peak Name	Retention Time min	Area mAU*min	Height mAU	Relative Area %	Relative Height %	Amount n.a.
1		16.663	693.709	615.390	96.91	96.90	n.a.
2		22.130	22.121	19.698	3.09	3.10	n.a.
**Total:**			**715.830**	**635.088**	**100.00**	**100.00**	

For entry 3.

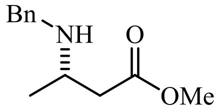

(S)-**1a**

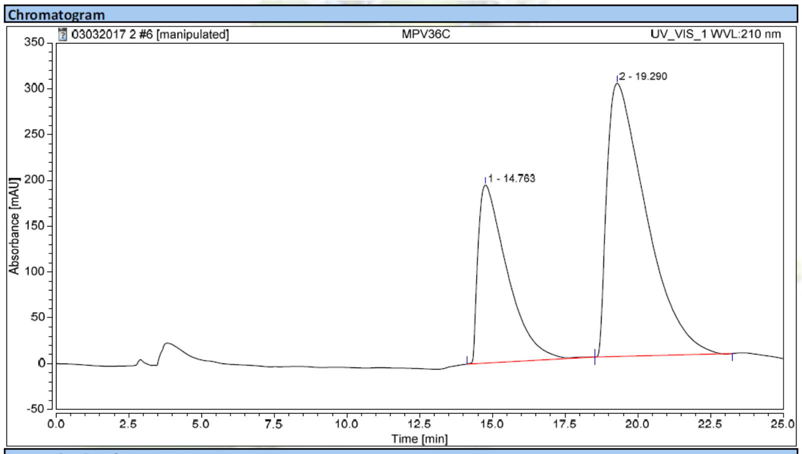

Integration Results

No.	Peak Name	Retention Time min	Area mAU*min	Height mAU	Relative Area %	Relative Height %	Amount n.a.
1		14.763	226.069	194.317	32.61	39.46	n.a.
2		19.290	467.236	298.136	67.39	60.54	n.a.
**Total:**			**693.305**	**492.453**	**100.00**	**100.00**	

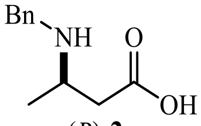

(R)-**2a**

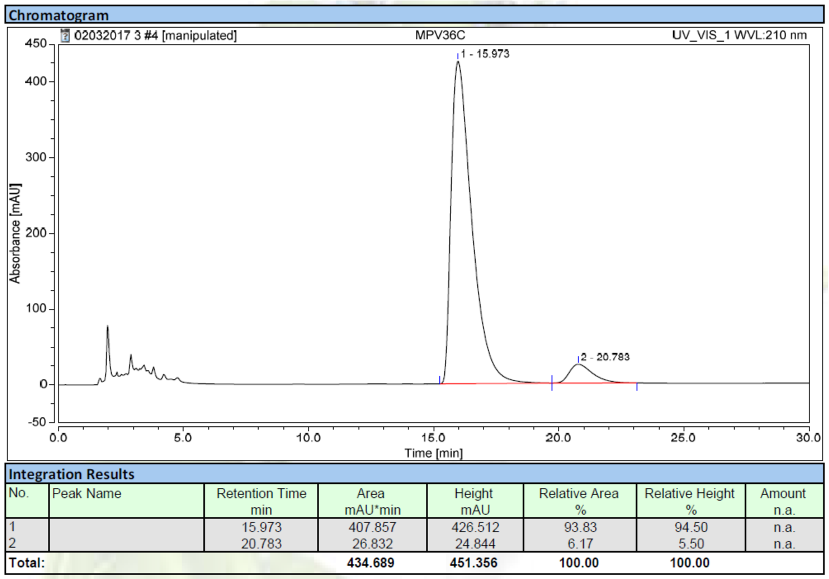

Integration Results

No.	Peak Name	Retention Time min	Area mAU*min	Height mAU	Relative Area %	Relative Height %	Amount n.a.
1		15.973	407.857	426.512	93.83	94.50	n.a.
2		20.783	26.832	24.844	6.17	5.50	n.a.
**Total:**			**434.689**	**451.356**	**100.00**	**100.00**	

For entry 4.

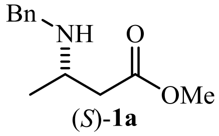

(*S*)-**1a**

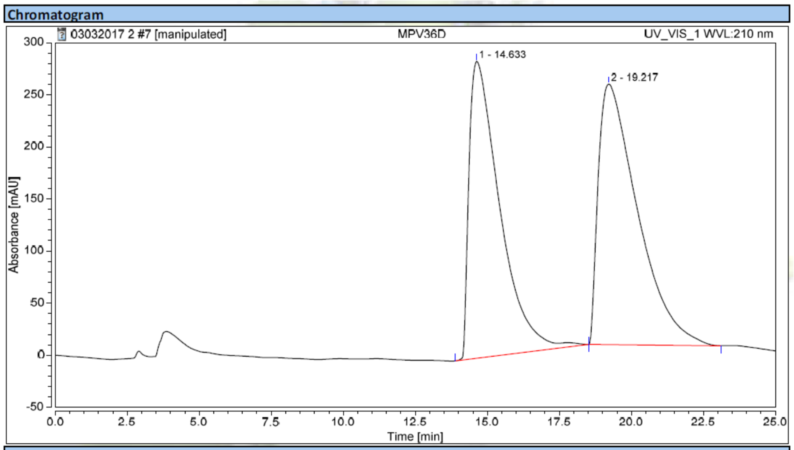

Integration Results

No.	Peak Name	Retention Time min	Area mAU*min	Height mAU	Relative Area %	Relative Height %	Amount n.a.
1		14.633	351.955	285.090	47.12	53.26	n.a.
2		19.217	394.972	250.142	52.88	46.74	n.a.
Total:			746.927	535.233	100.00	100.00	

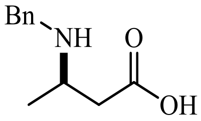

(*R*)-**2a**

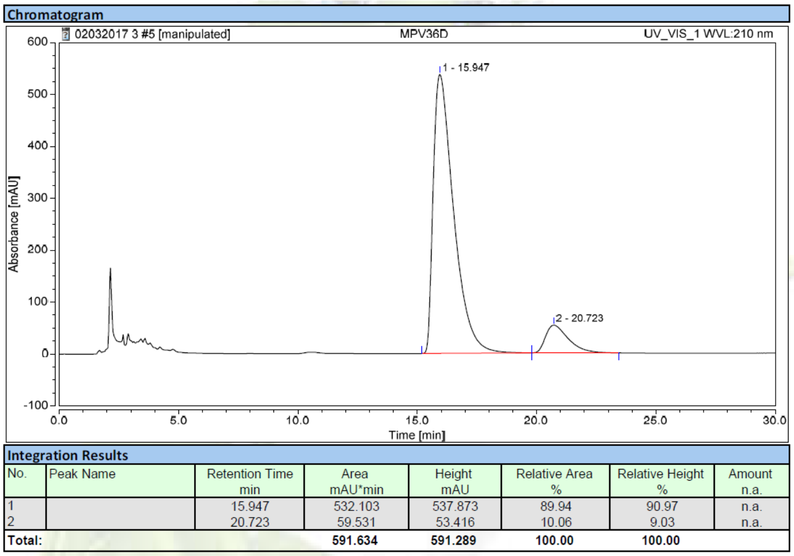

Integration Results

No.	Peak Name	Retention Time min	Area mAU*min	Height mAU	Relative Area %	Relative Height %	Amount n.a.
1		15.947	532.103	537.873	89.94	90.97	n.a.
2		20.723	59.531	53.416	10.06	9.03	n.a.
Total:			591.634	591.289	100.00	100.00	

For entry 5.

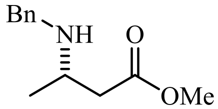

(*S*)-**1a**

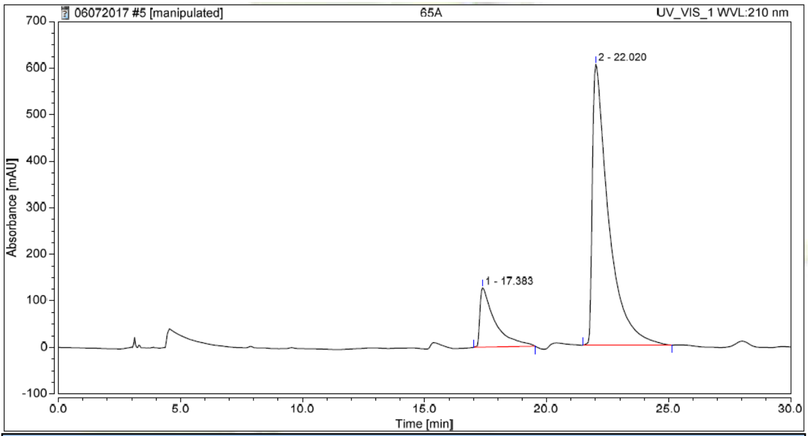

Integration Results							
No.	Peak Name	Retention Time min	Area mAU*min	Height mAU	Relative Area %	Relative Height %	Amount n.a.
1		17.383	91.498	127.524	16.49	17.44	n.a.
2		22.020	463.448	603.731	83.51	82.56	n.a.
Total:			554.946	731.255	100.00	100.00	

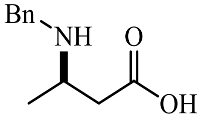

(*R*)-**2a**

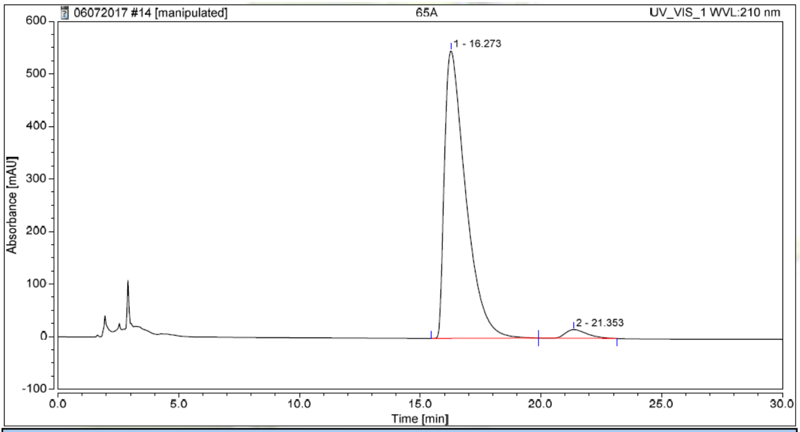

Integration Results							
No.	Peak Name	Retention Time min	Area mAU*min	Height mAU	Relative Area %	Relative Height %	Amount n.a.
1		16.273	556.137	547.420	97.05	97.09	n.a.
2		21.353	16.933	16.431	2.95	2.91	n.a.
Total:			573.070	563.851	100.00	100.00	

For entry 6.

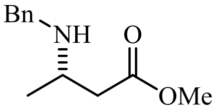

(*S*)-**1a**

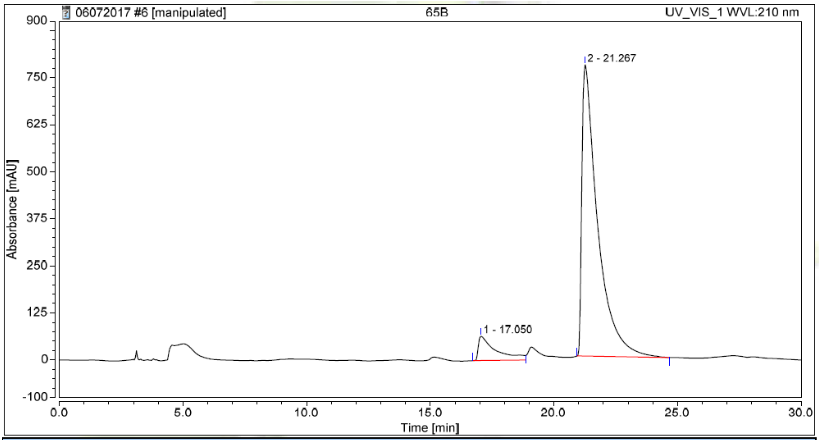

Integration Results							
No.	Peak Name	Retention Time min	Area mAU*min	Height mAU	Relative Area %	Relative Height %	Amount n.a.
1		17.050	53.349	64.519	8.54	7.69	n.a.
2		21.267	571.031	774.103	91.46	92.31	n.a.
Total:			624.381	838.622	100.00	100.00	

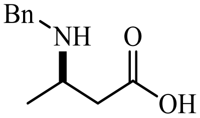

(*R*)-**2a**

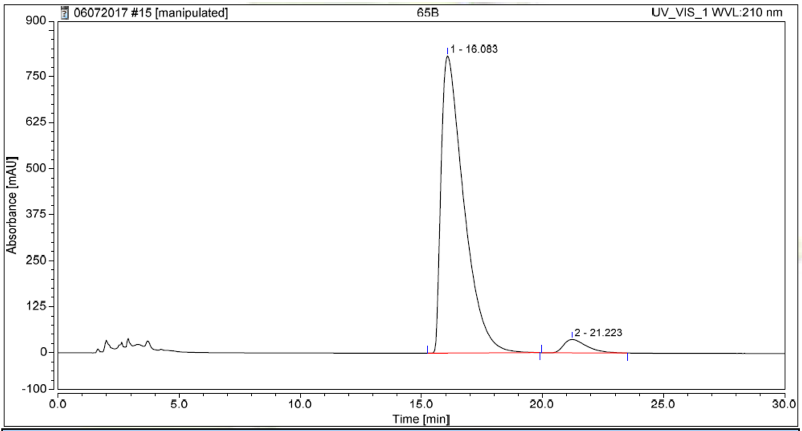

Integration Results							
No.	Peak Name	Retention Time min	Area mAU*min	Height mAU	Relative Area %	Relative Height %	Amount n.a.
1		16.083	876.598	807.399	95.70	95.70	n.a.
2		21.223	39.370	36.295	4.30	4.30	n.a.
Total:			915.968	843.694	100.00	100.00	

- **Chromatograms for scaling-up for the enzymatic hydrolysis using the substrate 1a (Table S4).**

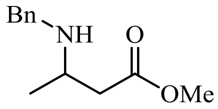

rac-**1a**

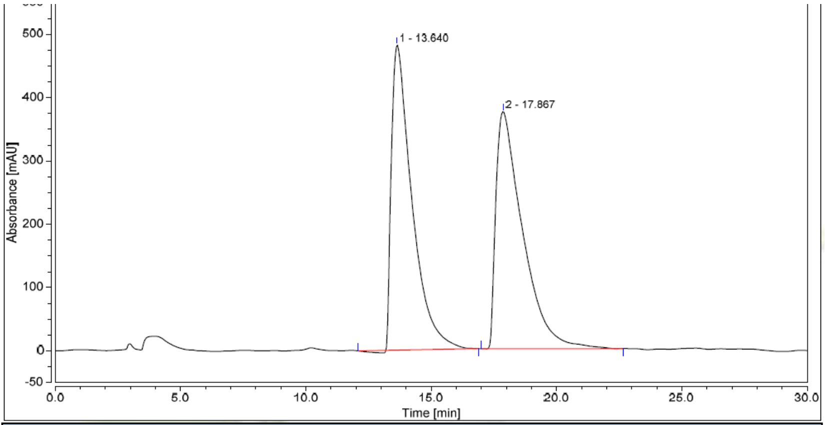

Integration Results							
No.	Peak Name	Retention Time min	Area mAU*min	Height mAU	Relative Area %	Relative Height %	Amount n.a.
1		13.640	455.884	482.090	49.63	56.27	n.a.
2		17.867	462.617	374.599	50.37	43.73	n.a.
**Total:**			**918.501**	**856.689**	**100.00**	**100.00**	

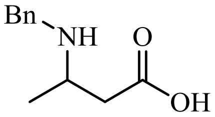

rac-**2a**

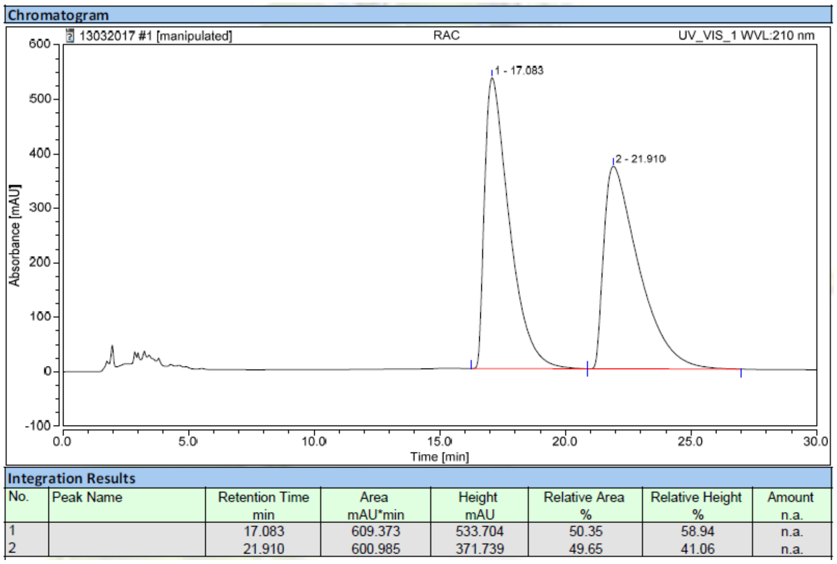

Integration Results							
No.	Peak Name	Retention Time min	Area mAU*min	Height mAU	Relative Area %	Relative Height %	Amount n.a.
1		17.083	609.373	533.704	50.35	58.94	n.a.
2		21.910	600.985	371.739	49.65	41.06	n.a.

For entry 1.

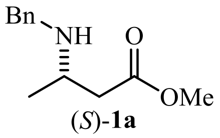

(*S*)-**1a**

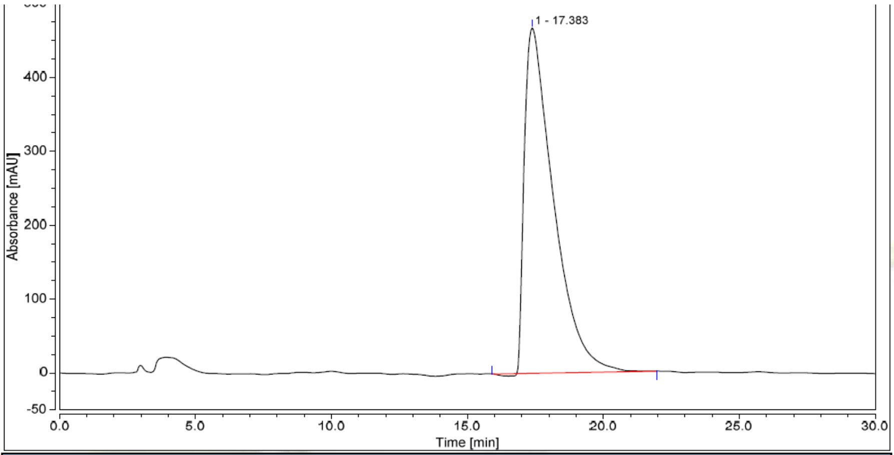

Integration Results							
No.	Peak Name	Retention Time min	Area mAU*min	Height mAU	Relative Area %	Relative Height %	Amount n.a.
1		17.383	579.249	467.640	100.00	100.00	n.a.
**Total:**			**579.249**	**467.640**	**100.00**	**100.00**	

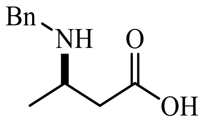

(*R*)-**2a**

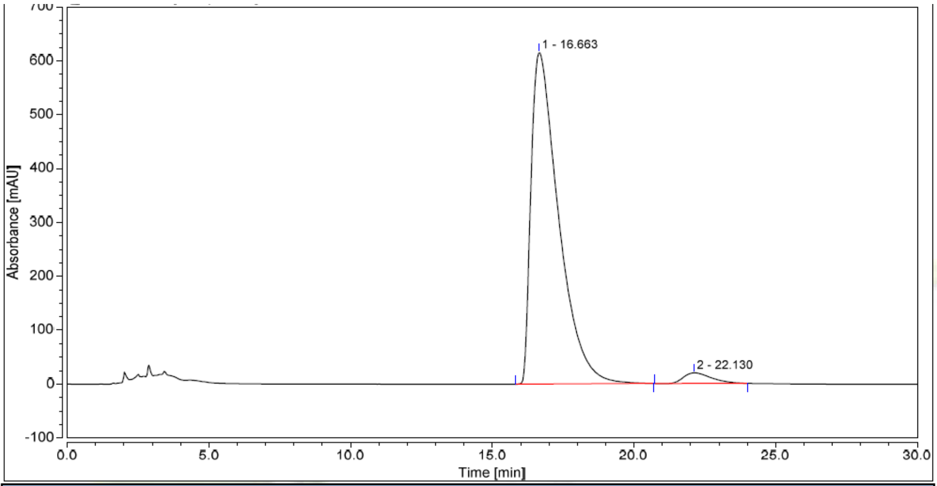

Integration Results							
No.	Peak Name	Retention Time min	Area mAU*min	Height mAU	Relative Area %	Relative Height %	Amount n.a.
1		16.663	693.709	615.390	96.91	96.90	n.a.
2		22.130	22.121	19.698	3.09	3.10	n.a.
**Total:**			**715.830**	**635.088**	**100.00**	**100.00**	

For entry 2.

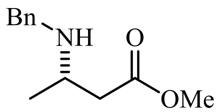

(*S*)-**1a**

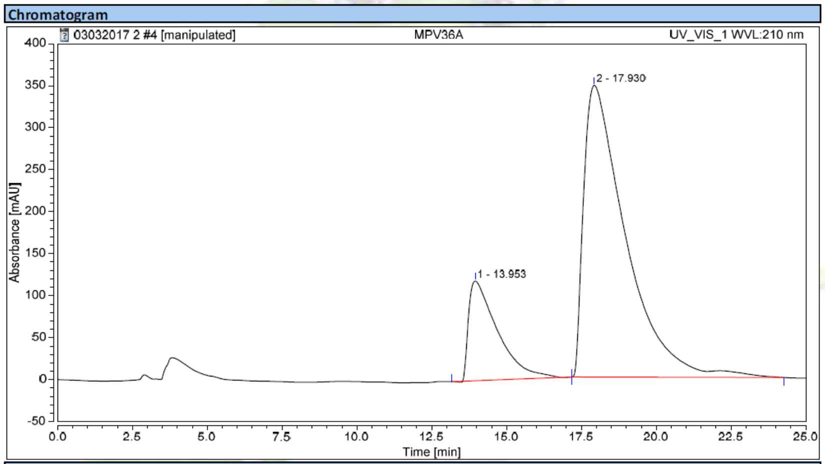

Integration Results

No.	Peak Name	Retention Time min	Area mAU*min	Height mAU	Relative Area %	Relative Height %	Amount n.a.
1		13.953	132.279	118.717	18.94	25.46	n.a.
2		17.930	565.995	347.548	81.06	74.54	n.a.
**Total:**			**698.273**	**466.264**	**100.00**	**100.00**	

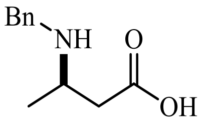

(*R*)-**2a**

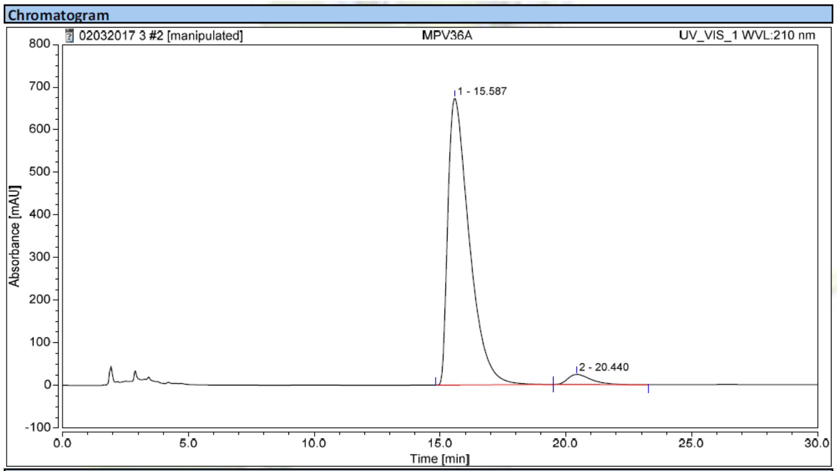

Integration Results

No.	Peak Name	Retention Time min	Area mAU*min	Height mAU	Relative Area %	Relative Height %	Amount n.a.
1		15.587	661.362	673.225	96.35	96.62	n.a.
2		20.440	25.060	23.541	3.65	3.38	n.a.
**Total:**			**686.423**	**696.767**	**100.00**	**100.00**	

For entry 3.

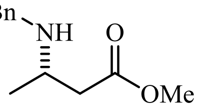

(*S*)-**1a**

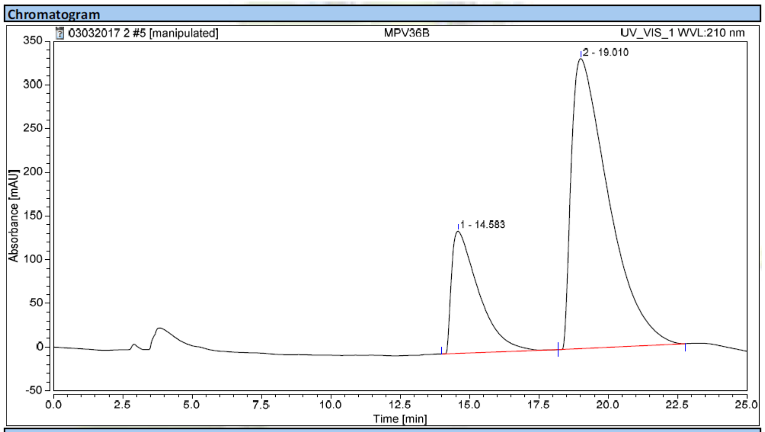

Integration Results

No.	Peak Name	Retention Time min	Area mAU*min	Height mAU	Relative Area %	Relative Height %	Amount n.a.
1		14.583	158.096	139.861	23.40	29.65	n.a.
2		19.010	517.470	331.834	76.60	70.35	n.a.
Total:			675.566	471.695	100.00	100.00	

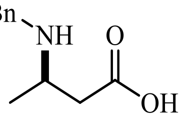

(*R*)-**2a**

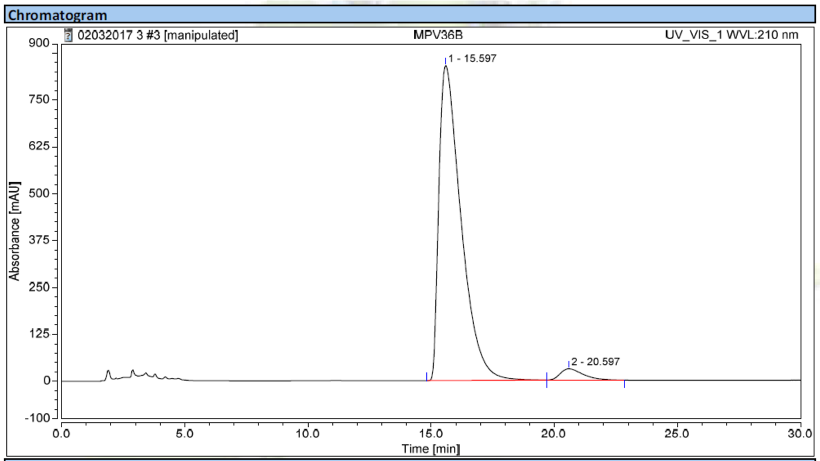

Integration Results

No.	Peak Name	Retention Time min	Area mAU*min	Height mAU	Relative Area %	Relative Height %	Amount n.a.
1		15.597	873.351	840.736	96.46	96.55	n.a.
2		20.597	32.079	30.054	3.54	3.45	n.a.
Total:			905.430	870.790	100.00	100.00	

For entry 4.

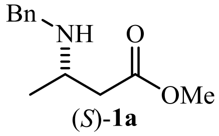

(*S*)-**1a**

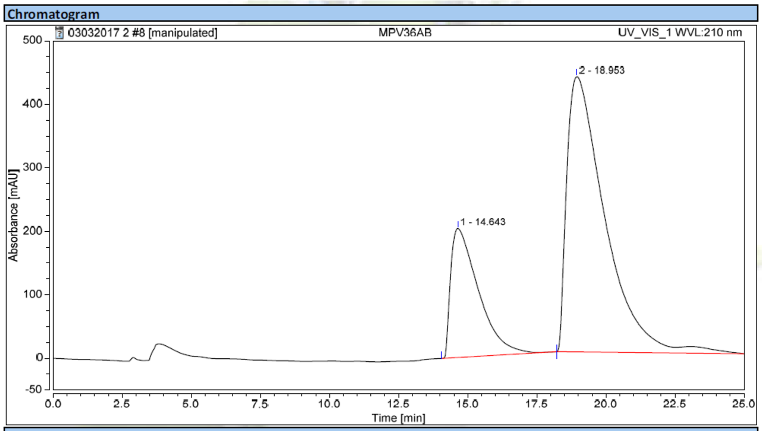

Integration Results

No.	Peak Name	Retention Time min	Area mAU*min	Height mAU	Relative Area %	Relative Height %	Amount n.a.
1		14.643	236.489	203.292	25.35	31.91	n.a.
2		18.953	696.572	433.696	74.65	68.09	n.a.
Total:			933.061	636.988	100.00	100.00	

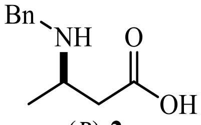

(*R*)-**2a**

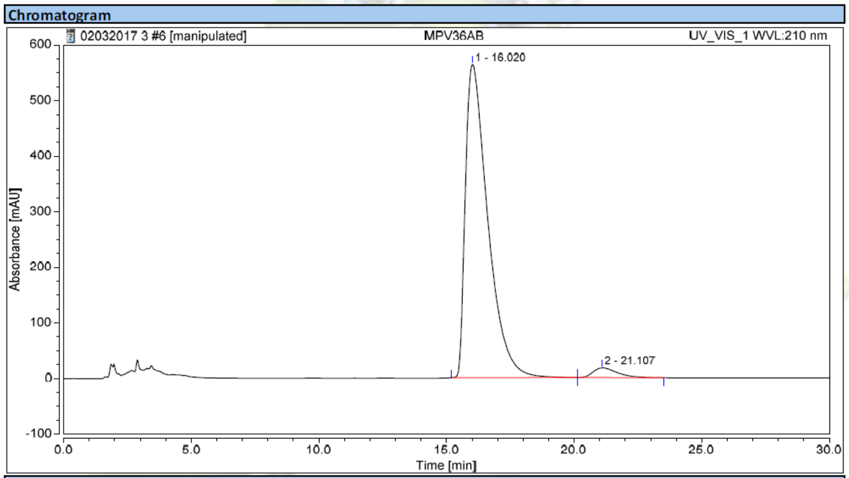

Integration Results

No.	Peak Name	Retention Time min	Area mAU*min	Height mAU	Relative Area %	Relative Height %	Amount n.a.
1		16.020	572.809	563.838	96.73	96.97	n.a.
2		21.107	19.386	17.592	3.27	3.03	n.a.
Total:			592.195	581.431	100.00	100.00	

7) HPLC conditions and retention times for the substrates *rac*-1a to *rac*-1j and hydrolysis products 2a to 2j.

Substrates		Products	
Conditions		Conditions	
	Column, Eluent, Flow(mL/min), Time(min)		Column, Eluent, Flow(mL/min), Time(min)
1a	Chiralpack **OD**, Hex/IPA (99:1), 1, 30	2a	Chirobiotic **TAG**, MeOH, 1, 30
1b	Chiralpack **OD-H**, Hex/IPA (98:2), 1, 30	2b	Chirobiotic **T**, EtOH/H_2O (70:30), 0.5, 30
1c	Chiralpack **OD-H**, Hex/IPA (99:1), 0.6, 30	2c	Chirobiotic **T**, EtOH/H_2O (70:30), 0.5, 30
1d	Chiralpack **AD-H**, Hex/IPA (99:1), 0.6, 30	2d	Chirobiotic **T**, EtOH/H_2O (70:30), 0.5, 30
1e	Chiralpack **AD-H**, Hex/IPA (99:1), 0.6, 30	2e	Chirobiotic **T**, EtOH/H_2O (70:30), 0.5, 30
1f	Chiralpack **AD-H**, Hex/IPA (99:1), 0.6, 30	2f	Chirobiotic **T**, EtOH/H_2O (70:30), 0.5, 30
1g	Chiralpack **OD-H**, Hex/IPA (95:5), 1, 30	2g	Chirobiotic **T**, MeOH/H_2O (95:5), 0.8, 20
1h	Chiralpack **OD**, Hex/IPA (99:1), 0.9, 45	2h	Chirobiotic **TAG**, MeOH, 0.8, 30
1i	Chiralpack **AD-H**, Hex/IPA (99:1), 1, 15	2i	Chirobiotic **T**, EtOH/H_2O (70:30), 0.5, 40

Substrates			Products		
Retention time (min)			Retention time (min)		
	t_R	t_S		t_R	t_S
1a	13.64	17.86	2a	17.08	21.91
1b	8.70	11.51	2b	17.14	20.50
1c	22.76	25.50	2c	16.31	19.50
1d	15.13	18.50	2d	14.93	17.58
1e	20.65	24.87	2e	14.03	16.96
1f	14.41	17.70	2f	13.21	15.69
1g	13.09	7.68	2g	10.97	10.05
1h	24.58	32.58	2h	7.96	7.13
1i	4.93	7.03	2i	17.46	29.07

8) Determinations of the absolute configuration for the products 2a to 2j.

For the determination of the absolute configuration of all products we used different methodologies. A crystallographic report for product (*R*)-**2a** was obtained and the ORTEP diagram are show below, as well, a CIF report is attached to this archive, the absolute configuration for this product was determined as *R,* furthermore, the specific optical rotation was compared with the reported by Escalante [4] (Table S2, entry 1).

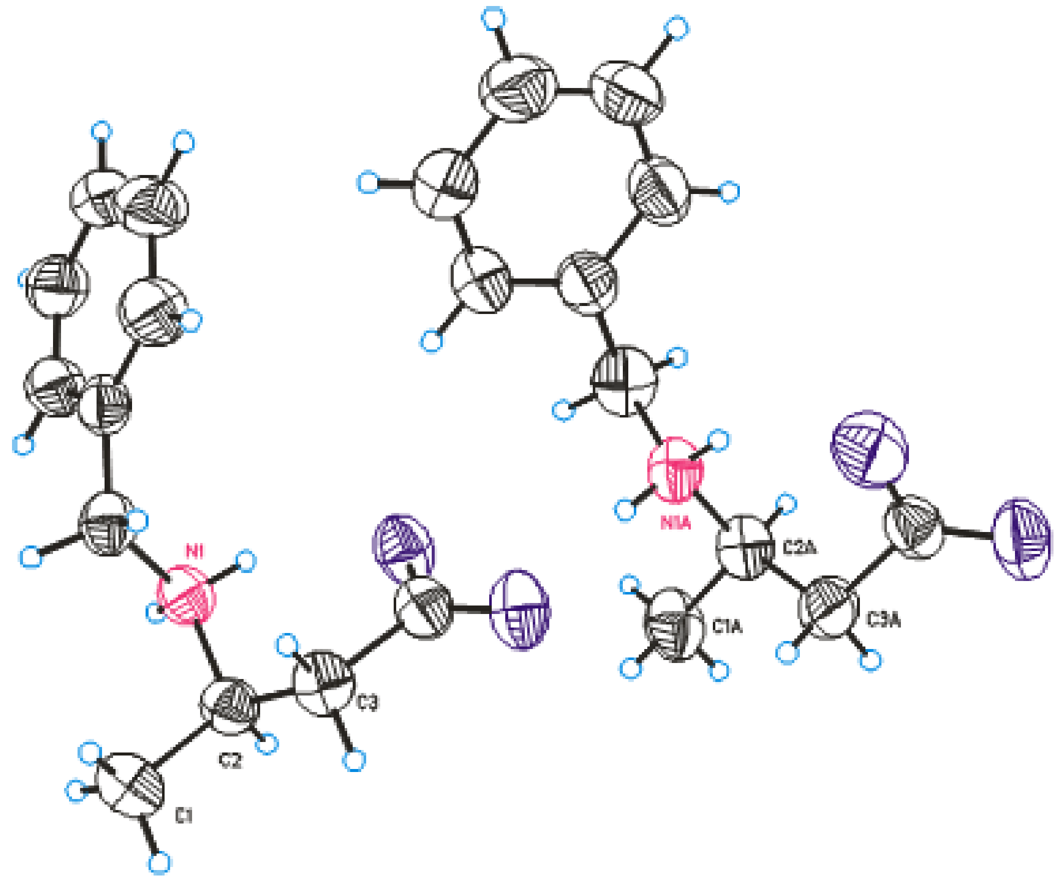

Figure S50. ORTEP diagram for product (*R*)-2a.

The absolute configuration of product **2b** was assigned by chemical comparison with the reported data [17], **reference** (*S*)-**2b** $[\alpha]_D^{25}$ = +32.4 and *ee*>99%**, product** (*R*)-**2b** $[\alpha]_D^{25}$ = -36.5 and *ee*=97%**,** obtaining an *R* configuration. The products **2c** to **2f** were assigned followed an elution sequence general rule already describe [18] using the same column that we used (Chirobiotic T). With the certainty that product **2b** has a well know absolute configuration and that the elution sequence for the majority product is the same for product **2b** to **2f** we assigned the same configuration for all them (*R*).

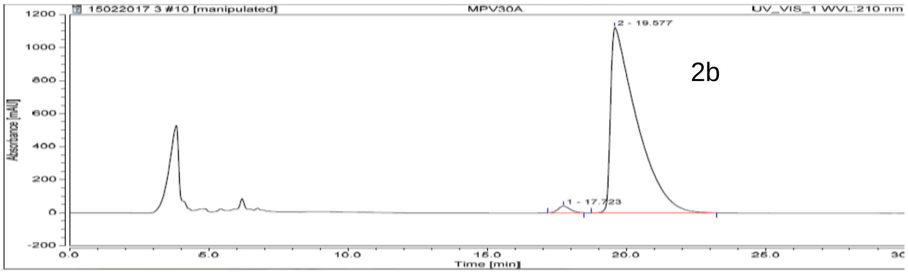

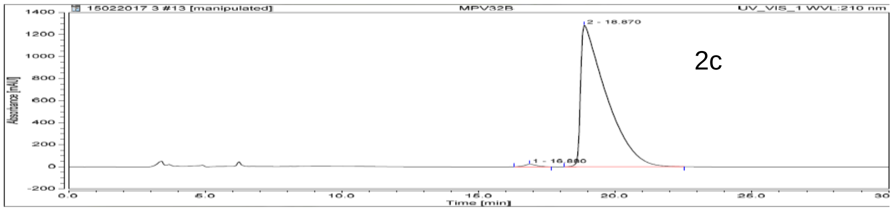

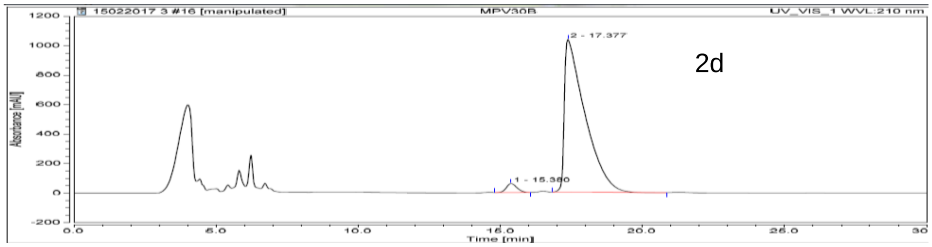

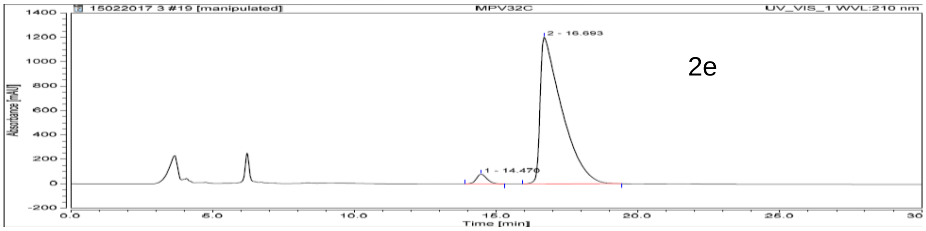

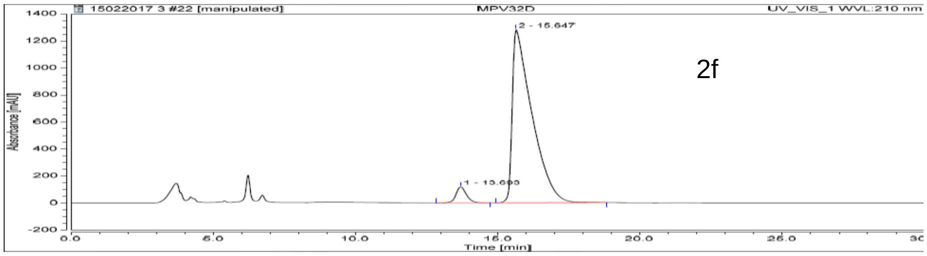

The absolute configuration for products **2g** and **2i** was assigned by chemical comparison [4], **reference** (*S*)-**2g**[4] $[\alpha]_D^{20}$ = -51.9 and *ee*>99%**, product** (*S*)-**2g** $[\alpha]_D^{25}$ = -35.0 and *ee*= 83%**, reference** (*S*)-**2i**[4] $[\alpha]_D^{20}$ = +26 and *ee*>99%**, product** (*S*)-**2i** $[\alpha]_D^{25}$ = +12.8 and *ee*= 94%**,** and for product **2h** an elution sequence general rule was used, compare product **2a** and **2h**, obtaining the *S* configuration for product **2h**.

Reference.

[1] Andrews, I. P.; Know, O. *Organic Synthesis*. **2012**, *12*, 586.

[2] Shearouse, W. C.; Korte, C. M.; Mack, J. *Green Chem*. **2011**, *13*, 598.

[3] Börner, C.; Dennis, M. R.; Sinn, E.; Woodward, S. *Eur. J. Org. Chem*. **2001**, 2435.

[4] Rangel, H.; Morales, M. C.; Galindo, J. M.; Castillo, E.; Zúñiga, A. O.; Juaristi, E.; Escalante, J. *Tetrahedron: Asymmetry*. **2015**, *26*, 325.

[5] Kandula, S. R.; Kumar, P. *Tetrahedron Asymmetry*, **2005**, *16*, 3268-3274.

[6] Alcock, S. G.; Baldwin, J. E.; Bohlmann, R.; Harwood, L. M.; Seeman, J. I. *J. Org. Chem*., **1985**, *50*, 3526-3535.

[7] O´Brien, C. J.; Tellez, J. L.; Nixon, Z. S.; Kang, L. J.; Carter, A. L.; Kunkel, S. R.; Przeworski, K. C.; Chass, G. A. *Angew. Chem. Int. Ed*., **2009**, *48*, 6836-6839.

[8] Malla, R. K.; Ridenour, J. N.; Spilling, C. D. *Beilstein J. Org. Chem*., **2014**, *10*, 1933-1941.

[9] Choudary, B. M.; Mahendar, K.; Kantam, M. L.; Ranganath, K. V. S.; Athar, T. *Adv. Synth. Catal*., **2006**, *348*, 1977-1985.

[10] Asao, N.; Uyehara, T.; Tamamoto, Y. *Tetrahedron,* **1988**, *44*, 4173-4180.

[11] Guizzetti, S.; Benaglia, M.; Bonsignore, M.; Raimondi, L. *Org. Biomol. Chem*., **2011**, *9*, 739-743.

[12] Wang, W.-B.; Roskamp, E. J. *J. Am. Chem. Soc*., **1993**, *115*, 9417, 9420.

[13] Bonsignore, M.; Benaglia, M.; Annunziata, R.; Celentano, G. *SYNLETT*, **2011**, *8*, 1085-1088.

[14] Gedey, S.; Liljeblad, A.; Lázár, L.; Fülöp, F.; Kanerva, L. T. *Tetrahedron: Asymmetry*, **2001**, *12*, 105-110.

[15] Bach, K. K.; El-Seedi, H. R.; Jensen, H. M.; Nielsen, H. B.; Thomsen, I.; Torssell, K. B. G. *Tetrahedron*, **1994**, *50*, 7543-7556.

[16] Blicke, F. F.; Gould, W. A. *J. Org. Chem*. **1985**, *23*, 1102.

[17] Ma, D-Y.; Wang, D.-X.; Pan, J.; Huang, Z-T.; Wang, M.-X. *J. Org. Chem*. **2008**, *73*, 4087.

[18] Péter, A.; Lázár, L.; Fülöp, F.; Armstrong, D.W. *J. Chromatogr. A*. **2001**, *926*, 229.
